# Modulating IDO1 and TDO Inhibition Through Structural Modification of Diaryl Hydroxylamines

**DOI:** 10.1002/cmdc.202501087

**Published:** 2026-04-07

**Authors:** Angeliki S. Foscolos, Alexandros Pappas, Christos N. Petroulias, Anna Kapella, Georgia M. Prifti, Grigoris Zoidis, Minas Papadopoulos, Anastasia Mpakali, Aristeidis Chiotellis

**Affiliations:** ^1^ Institute of Nuclear & Radiological Sciences & Technology, Energy & Safety National Center for Scientific Research “Demokritos” Athens Greece; ^2^ Division of Pharmaceutical Chemistry Department of Pharmacy School of Health Sciences National and Kapodistrian University of Athens Athens Greece

**Keywords:** dioxygenases, hydroxylamines, IDO1, kynurenine, TDO

## Abstract

Diaryl hydroxylamines have emerged as promising scaffolds for targeting the tryptophan‐catabolizing enzymes indoleamine 2,3‐dioxygenase 1 (IDO1) and tryptophan 2,3‐dioxygenase (TDO), key drivers of tumor immune escape. Building on the reported dual IDO1/TDO inhibitory activity of *O*‐((3,5‐difluorophenyl)(phenyl)methyl)hydroxylamine, a series of diaryl hydroxylamines and related analogs were designed and synthesized to probe structure–activity relationships governing dioxygenase inhibition. Structural modifications included variation of the aryl framework toward heteroaromatic and highly electron‐deficient motifs, amide‐linked scaffold elongation, bioisosteric replacement of the hydroxylamine group with thiol or oxime functionalities, and *O*‐benzylhydroxylamine derivatives. Inhibition studies revealed that 1,1′‐diaryl hydroxylamines bearing five‐membered heterocycles and electron‐deficient aryl groups favored micromolar to submicromolar inhibition of both enzymes, whereas scaffold elongation promoted pronounced IDO1 preference and thiol or oxime analogs exhibited diminished potency. Among the compounds evaluated, *O*‐benzylhydroxylamine **28l** (IDO1/TDO IC_50_ = 0.031/2.9 μM) and 1,1′‐diaryl hydroxylamine **16j** (IDO1/TDO IC_50_ = 0.18/5.5 μM) displayed the most favorable activity profiles. This SAR study defines key determinants of IDO1 potency while retaining significant TDO inhibition and identifies fluorinated hydroxylamines as potential starting points for future PET imaging probe development.

## 1 Introduction

Tryptophan (TRP), an essential amino acid, has recently emerged as a crucial player in the field of cancer immunotherapy. Extensive evidence suggests that tryptophan depletion within the tumor microenvironment can significantly impair the function and proliferation of immune cells, particularly T cells, while also promoting the differentiation and activity of immunosuppressive regulatory T cells [1]. This depletion is mainly mediated through activation of the kynurenine pathway (KP), the major pathway of tryptophan metabolism in mammals. The rate‐limiting step in the KP is the enzymatic conversion of TRP to *N*‐formylkynurenine catalyzed by indoleamine‐2,3‐dioxygenase 1 (IDO1), indoleamine‐2,3‐dioxygenase 2 (IDO2) and tryptophan‐2,3‐dioxygenase (TDO). N‐formylkynurenine then spontaneously hydrolyzes to kynurenine (KYN) that can act on aryl hydrocarbon receptors to further dampen antitumor immune responses [2, 3]. Among the many enzymes involved in the KP, the aberrant expression of IDO1/2 and TDO has been identified as the key mediator of KP activation leading to a pathological immune escape in cancer.

The role of IDO1 in tumor immune evasion is well established, with upregulation reported in more than 20 human tumor types and correlated with immunosuppression, increased invasiveness, and poor prognosis [4, 5]. Beyond its classical role in tryptophan depletion, IDO1 is increasingly recognized as a broader regulator of immune oncology, acting through mechanisms both dependent and independent of adaptive immunity, including synergistic expression with immune checkpoint regulators such as PD‐1/PD‐L1 and induction following chemotherapy or radiotherapy, contributing to adaptive resistance [6]. In addition, TDO expression has been reported in several clinically relevant tumors, including brain, lung, and breast cancers [5]. IDO2 expression has also been detected in multiple tumor types, although its precise biological role remains less well defined [7].

Given its central role in shaping an immunosuppressive tumor microenvironment, IDO1 has been intensively explored as a therapeutic target [7]. While IDO1‐selective inhibitors demonstrated encouraging preclinical efficacy and synergy with immunotherapy and conventional treatments, clinical translation has been challenging. Notably, the phase III ECHO‐301/KEYNOTE‐252 trial showed no survival benefit for the IDO1 inhibitor epacadostat in combination with pembrolizumab, underscoring the complexity of kynurenine pathway (KP) biology and compensatory immune mechanisms in humans.

These findings have renewed interest in the contribution of related dioxygenases, particularly TDO. Increasing evidence indicates that IDO1 and TDO are co‐expressed in multiple tumor types, including glioblastoma, lung, breast, pancreatic, and colorectal cancers [8–14]. This redundancy suggests that selective inhibition of a single enzyme may be insufficient, thereby supporting the development of compounds capable of targeting multiple KP enzymes. In this context, dual or pan‐dioxygenase inhibitors have gained attention, with early clinical evaluation of the dual IDO1/TDO inhibitor M4112 demonstrating acceptable safety and preliminary efficacy [15].

**FIGURE 1:**
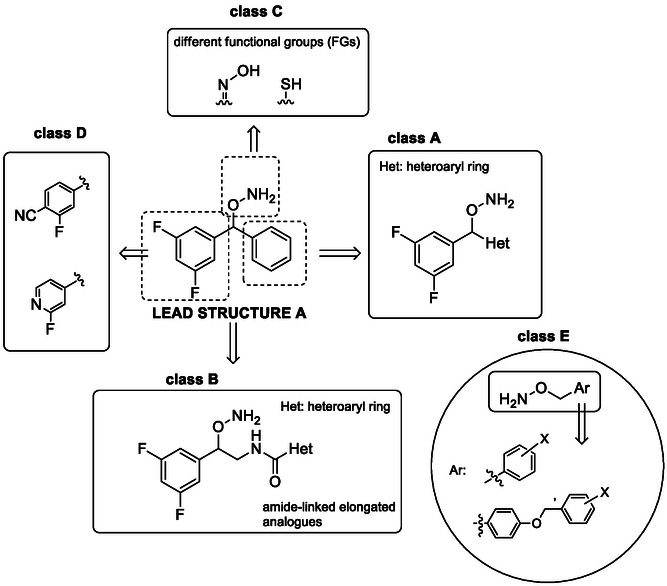
Design strategy and structural classes explored in this study. Starting from the previously identified dual‐active 3,5‐difluorophenyl lead compound A, four modification series were explored (A–D). (A) short 1,1′ diaryl analogs with heteroaryls; (B) elongated (1,4′ diaryl) amide‐linked derivatives with heteroaryls; (C) hydroxylamine bioisosteres incorporating thiol or, oxime iron‐binding groups; (D) electron deficient rings. A series of *O*‐benzylhydroxylamines (E) were also evaluated for their IDO1/TDO inhibitory potential.

Within this context, the hydroxylamine scaffold has emerged as a promising structure for IDO1 inhibition, with both *O‐* substituted [16] and *N*, *O‐*substituted hydroxylamines [17] showing promising in vitro activity. In a recent study [18], the diaryl hydroxylamine scaffold yielded potent pan and dual inhibitors of IDO1, IDO2 and TDO and structure–activity relationships established the importance of extensive ring halogenation for achieving increased potency against these dioxygenase enzymes. Building on these findings, we further investigated the IDO1/TDO inhibitory potential of this interesting scaffold. Compound **A** (Figure 1), which carries a 3,5‐difluorophenyl functionality, was identified as one of the most potent compounds of the diaryl hydroxylamine scaffold for dual and/or pan inhibition, exhibiting an IC_50_ value in the low μM range against IDO1 and TDO (0.357 and 3.48 μM respectively). This compound was therefore selected as the lead compound for the design of four classes of compounds (A–D), as shown in Figure 1.

In classes A–C, the 3,5‐difluorophenyl moiety was retained to ensure enhanced potency demonstrated in the previous study while in class D, this moiety was replaced with a phenyl ring carrying a fluorine substituent in combination with an electron‐withdrawing group on the same ring. Fluorine substituents were preferred over other halogens in all synthesized compounds to facilitate the introduction of an ^18^F radiolabel in future stages of this work. The broader objective of this study was to identify lead compounds with improved IDO1/TDO inhibitory potency, while incorporating structural features that may enable future development into ^18^F‐radiolabeled imaging probes for positron emission tomography (PET). This work therefore focuses on compound design and biological evaluation, providing a foundation for subsequent radiolabeling and imaging studies. The development of a ^18^F‐labeled probe is important because a serious obstacle hampering the unambiguous evaluation of the therapeutic potential of novel dioxygenase inhibitors in a clinical setting is the lack of reliable biomarkers able to routinely detect and monitor the activation of the KP^23^.

Class A comprises 1,1′‐diaryl hydroxylamines in which the phenyl ring of compound A was replaced with various heteroaromatic scaffolds, aiming to explore the influence of ring electronics and heteroatom incorporation on dioxygenase inhibition. Such modifications have been shown in other IDO1 inhibitor classes [19] to modulate potency through altered steric and electronic profiles rather than through a defined binding mode. In class B, a short spacer was introduced between the 3,5‐difluorophenyl ring and the heteroaromatic unit, affording 1,4′‐diarylhydroxylamines. This design maintained a compact molecular architecture, motivated by previous observations that extended spacers can adversely affect TDO inhibition [18]. In this class, the heteroaromatic rings were connected via an amide linkage to probe the impact of increased polarity and hydrogen‐bonding capacity on inhibitory activity. In class C, the hydroxylamine moiety was replaced with alternative iron(II)‐coordinating functional groups, namely thiols [20, 21, 22] and oximes [23, 24] as part of a bioisosteric strategy. Oxime derivatives were synthesized exclusively for the short diaryl hydroxylamine analogs, whereas thiol derivatives were prepared for both short and extended structures. Given prior evidence that hydroxylamines can engage the heme iron of dioxygenases [16], these substitutions were explored to assess whether stronger metal‐binding groups could influence inhibitory potency. Class D retained the phenyl scaffold of compound A while combining fluorine substitution with electron‐withdrawing groups on the opposing ring to modulate electronic properties. Finally, a series of *O*‐benzylhydroxylamines (class E) was evaluated against IDO1 and TDO. While this scaffold has previously been reported as an IDO1 inhibitor [16], its activity against TDO has not been investigated to date.

## 2 Results and Discussion

### 2.1 Chemistry

The synthesis of the 1,1′‐diaryl derivatives commenced with the addition of aryl Grignard reagents to the appropriate heteroaryl‐2‐carbaldehydes, affording the corresponding secondary alcohols in good to excellent yields.

For the hydroxylamine series, the alcohols were converted to the corresponding phthalimides under Mitsunobu conditions, followed by hydrazinolysis to furnish the desired hydroxylamines. Most compounds were isolated as free bases. Derivatives bearing 2‐furanoyl, 2‐pyrrolyl, or 2‐indolyl heteroaromatic rings exhibited pronounced chemical instability and were therefore excluded from biological evaluation.

Oxime derivatives were obtained by oxidation of the alcohols to the corresponding ketones using MnO_2_, followed by condensation with hydroxylamine, affording the oximes typically as E/Z mixtures, which were evaluated without further separation.

**SCHEME 1:**
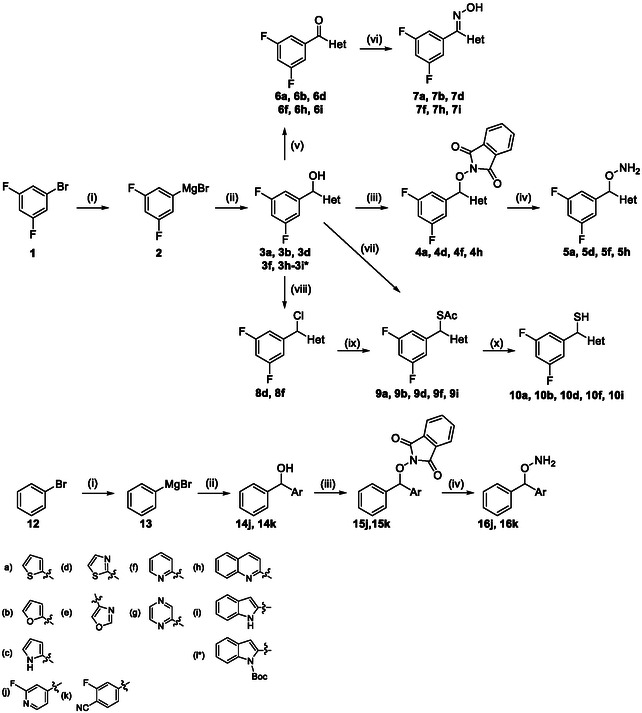
Synthesis of 1,1′‐diaryl compounds. Reagents and conditions: (i) Mg, I_2_ (cat.), THF, reflux, 90 min; (ii) aryl‐carbaldehyde, THF, −78°C, 30 min, 57%–96%; (iii) *N*‐hydroxyphthalimide, DIAD, triphenylphosphine, THF, −10°C to RT, overnight, 67%–88%; (iv) aq*.* NH_2_NH_2_ (55%) EtOH/DCM 1:1, RT, 60–120 min, 73%–91%; (v) activated MnO_2_, DCM, RT, overnight, 93%–99%; (vi) NH_2_OH·HCl, Na_2_CO_3_, EtOH, reflux, 2–48h, 84%–95%; (vii) DIAD, triphenylphosphine, thioacetic acid, THF, 0°C to RT, overnight, 52%–55% (**9a**, **9b** and 9i*); for the Boc‐protected derivative (9i*) subsequent Boc deprotection (TFA/DCM, 0°C ‐ RT, 60 min) afforded **9i**; (viii) SOCl_2_, DMF (cat.), DCM, 0°C to RT, 1–15 h, quantitative yield (**8d** and **8f**) or MsCl, Et_3_N, DCM, 0°C to RT, overnight, quantitative yield (**8d**); (ix) KSAc, DMF, RT, 2 h– overnight, 45%–75% (**9d**, **9f**); (x) NaOH (1M), EtOH, 0°C, 5–60 min then HCl (1M), 0°C to RT, 77%–83%.

Thiol derivatives were synthesized via conversion of the alcohols to the corresponding thioacetates, either through chlorination followed by nucleophilic substitution with potassium thioacetate or directly via Mitsunobu conditions with thioacetic acid. Attempts to access the corresponding mesylate intermediates were unsuccessful, most likely due to the high SN1 reactivity of diaryl mesylates, which favors spontaneous halide substitution under the reaction conditions. Subsequent hydrolysis under basic conditions furnished the free thiols. Short‐chain thiols showed limited stability toward oxidative dimerization, whereas longer‐chain analogs were significantly more stable (Scheme 1).

**SCHEME 2:**
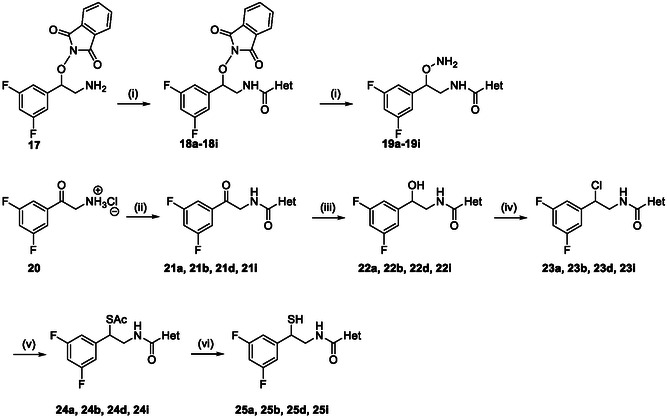
Synthesis of 1,4′‐diaryl compounds. Reagents and conditions: (i) heteroaryl acid, TBTU, DIPEA, DMF, RT, overnight then aq. NH_2_NH_2_ (55%) THF, RT, 60–90 min, or aq. MeNH_2_ (40%), EtOH/THF 3:1, RT, overnight, (34%–89% over two steps); (ii) EDC·HCl, DIPEA, heteroaromatic acid, DCM, 0°C to RT, 2–6 h, 74%–96% or TBTU, DIPEA, DMF, RT, 15% (**21i**); (iii) NaBH_4_, EtOH, 0°C, 2 h to RT, 40‐quant%; (iv) SOCl_2_, DMF (cat.), DCM, 0°C to RT, 1–2 h, quantitative yield (**23a**, **23b** and **23d**) or MsCl, Et_3_N, DCM, 0°C to RT, overnight, quantitative yield (**23a** and **23i**); (v) KSAc, DMF, RT, 2 h– overnight, 25%–64%; (vi) NaOH (1M), EtOH, 0°C, 5–60 min then HCl (1M), 0°C to RT, 53%–77%.

The synthesis of the long hydroxylamine derivatives (Scheme 2) followed previously reported procedures that successfully afforded compounds 18b, 18d, and 18h [25]. The key intermediate 2‐(2‐amino‐1‐(3,5‐difluorophenyl)ethoxy)isoindoline‐1,3‐dione [25] was coupled with the corresponding heteroaryl‐2‐carboxylic acids and subsequently subjected to hydrazinolysis to yield the desired hydroxylamines.

For the long thiol series, the key intermediate 20 was coupled to the appropriate heteroaryl‐2‐carboxylic acids, followed by reduction to the corresponding alcohols and conversion to the thiol functionality via thioacetate intermediates, as previously reported [14]. In contrast to the short‐chain analogs, the long thiols demonstrated significantly greater stability toward oxidative dimerization.

**SCHEME 3:**
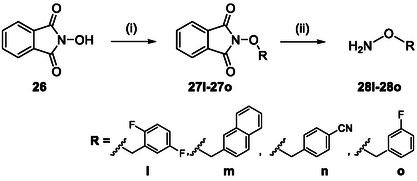
Synthesis of O‐Substituted Hydroxylamines. Reagents and conditions: (i) bromides (**l**‐**o**), NaH, DMF, 0°C to RT, overnight, 54%–99% %; (ii) aq. H_2_NNH_2_, DCM or MeOH, RT, 3 h, 72%–99%.

**SCHEME 4:**
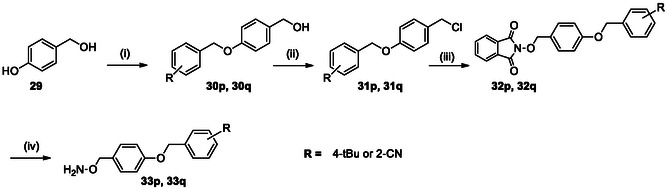
Synthesis of etherified *O*‐Substituted Hydroxylamines. Reagents and conditions: (i) benzyl halides (**p**, **q**), Cs_2_CO_3_, MeCN, reflux, 20 h, 99%; (ii) SOCl_2_, reflux, 1 h, 44%–82%, (iii) NaH, DMF, 0°C to RT, overnight, 46%–66%; (iv) aq. H_2_NNH_2_, DCM, RT, 3 h, 99%.

The *O*‐substituted hydroxylamines were obtained via a two‐step synthetic sequence as previously described [26], involving alkylation of N‐hydroxyphthalimide followed by hydrazinolysis (Scheme 3). Etherified hydroxylamines were synthesized using a reported four‐step protocol based on Williamson ether formation, chlorination, phthalimide substitution, and final hydrazinolysis [26] (Scheme 4). Full experimental details are provided in the Supporting Information.

### 2.2 Biological Activity

#### 2.2.1 IDO1 and TDO Enzymatic Inhibition Assays

All compounds were tested as racemic mixtures or E/Z mixtures (for oximes), so one isomer could be the preferred, more potent species. Enzymatic inhibition was evaluated using human IDO1 and TDO recombinant enzymes, and the resulting IC_50_ values are summarized in Table 1.

**TABLE 1:** Enzymatic and cellular IC_50_ values for IDO1 and TDO.

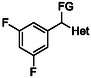						
Compound	Heterocycle	**Functional** **group**	IDO1 enzymatic assay (μM)	TDO enzymatic assay (μM)	IDO1 cell assay (μM)	TDO cell assay (μM)
**5a**	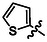	–ONH_2_	0.09	>10	>10	>10
**5d**	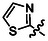	–ONH_2_	∼0.5^†^	>10	3.0	3.7
**5f**	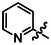	–ONH_2_	<1^†^	>10	9.9	>10
**5h**	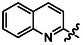	–ONH_2_	<1^†^	>10	>10	–a
**16j**	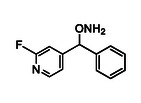	–ONH_2_	0.18	10.9	1.7	5.5
**16k**	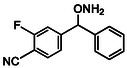	–ONH_2_	0.13	∼10	5.7	>10
**10a**	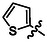	–SH	>10^†^	>10^†^	>10^†^	>10^†^
**10b**	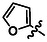	–SH	>10^†^	>10^†^	>10^†^	∼10^†^
**10d**	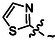 ∼	–SH	>10^†^	>10^†^	>10^†^	>10^†^
**10i**	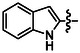	‐SH	>10^†^	>10^†^	>10^†^	>10^†^
**7a**	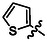	=N–OH	>10^†^	>10^†^	>10^†^	>10^†^
**7b**	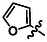	=N–OH	>10^†^	5.46	>10^†^	>10^†^
**7d**	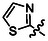	=N–OH	>10^†^	>10^†^	<10	—
**7f**	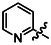	=N–OH	>10^†^	>10^†^	>10^†^	—
**7h**	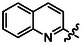	=N–OH	>10^†^	>10^†^	>10^†^	—
**7i**	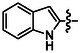	=N–OH	>10^†^	>10^†^	>10^†^	—

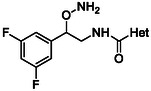						
Compound	Heterocycle	**Functional** **group**	IDO1 enzymatic assay (μM)	TDO enzymatic assay (μM)	IDO1 cell assay (μM)	TDO cell assay (μM)
**19a**	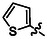	–ONH_2_	∼3^†^	>10^†^	>10	—
**19b**	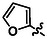	–ONH_2_	6.9	>10	>10	>10^†^
**19c**	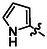	–ONH_2_	>10^†^	>10^†^	>10	—
**19d**	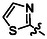	–ONH_2_	∼5^†^	>10^†^	>10^†^	—
**19e**	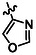	–ONH_2_	>10^†^	>10^†^	>10^†^	—
**19f**	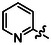	–ONH_2_	∼5^†^	>10^†^	<10^†^	—
**19g**	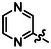	–ONH_2_	3.8	>10	>10	>10
**19h**	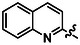	–ONH_2_	>10^†^	>10^†^	–	>10
**19i**	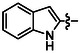	–ONH_2_	5–10^†^	>10^†^	2.82	—
**25a**	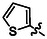	–SH	∼10^†^	>10^†^	>10^†^	>10^†^
**25b**	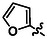	–SH	>10^†^	>10^†^	>10^†^	>10^†^
**25d**	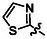	–SH	>10^†^	>10^†^	∼10^†^	>10^†^
**25i**	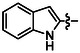	–SH	>10^†^	>10^†^	>10^†^	>10^†^

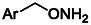					
Compound	Ar	IDO1 enzymatic assay (μM	TDO enzymatic assay (μM)	IDO1 cell assay (μM)	TDO cell assay (μM)
**28l**	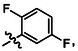	0.031	2.9	0.7	4.8
**28m**	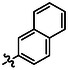	>10^†^	>10^†^	>10^†^	—
**28n**	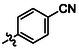	>10^†^	>10^†^	>10^†^	—
**28o**	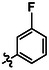	>10^†^	>10^†^	>10^†^	—
**33p**	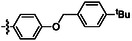	>10^†^	>10^†^	>10^†^	—
**33q**	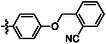	>10^†^	>10^†^	>10^†^	—

*Notes:* IC_50_ values were obtained by nonlinear regression analysis of dose–response data using a four‐parameter nonlinear regression logistic model. For selected representative compounds, IC_50_ values were determined from full dose–response curves generated using an 11‐point, half‐log serial dilution series (1 nM–100 μM).

† Estimated IC_50_ values derived from 3‐point concentration profiling (0.1, 1, and 10 μM; technical duplicates), reported for comparative SAR ranking. Compounds that did not reach 50% inhibition at 10 μM are reported as >10 μM.

a Not tested.

For the 1,1′‐diaryl hydroxylamines (class A), five‐membered ring heterocycles (**5a**, **5d**) generally conferred measurable inhibition for both IDO1 and TDO in the low micromolar range. For example, compound **5d** (2‐thiazolyl) inhibited IDO1 with an IC_50_ value of 0.5 μM, while TDO inhibition was observed only at concentrations >10 μM. Similarly, **16j** and **16k**, bearing fluorine‐substituted electron‐deficient rings, showed enzyme IC_50_ values of 0.13–0.18 μM for IDO1 and ∼10 μM for TDO. These results indicate that a highly electron‐deficient aryl ring may be an important determinant for achieving potent IDO1/TDO inhibition. Replacing the five‐membered ring with six‐membered or larger heterocycles shifted selectivity toward IDO1 only. In particular, compounds **5f** and **5h** lost significant TDO inhibition (>10 μM in enzymatic assays) while maintaining relatively potent IDO1 activity (<1 μM). Additional analogs of this series (including 2‐furanoyl, 2‐pyrrolyl, and 2‐indolyl derivatives) were synthesized but could not be biologically evaluated due to poor chemical stability.

Class B comprised elongated hydroxylamines (1,4′‐diaryl derivatives), which were systematically investigated due to their increased chemical stability relative to the corresponding 1,1′‐diaryl analogs. These compounds were generally selective for IDO1, displaying enzymatic IC_50_ values ranging from 3.8 to 6.9 μM (e.g., **19b**, **19g**), while TDO inhibition remained weak or absent (IC_50_ > 10 μM). Overall, the introduction of an amide linker or various heteroaryl groups did not significantly enhance IDO1 potency nor contribute to TDO inhibition. These observations suggest that even modest elongation of the inter‐aryl chain constrains TDO binding, possibly due to sterically hindered access to the narrower TDO pocket. Additionally, the combination of an amide linker with heteroaryl moieties does not necessarily translate into stronger IDO1 binding, potentially due to suboptimal geometry or solvation effects.

Oxime substitution (class C; **7a**–**7i**) abolished inhibitory activity against both enzymes, indicating that replacement of the hydroxylamine group with an oxime metal‐binding motif does not improve target engagement. For this reason, the synthesis and evaluation of 1,4′‐diaryl oxime derivatives were not pursued.

Thiol derivatives **(**class C**)** were also evaluated in enzymatic assays as analogs of both the 1,1′‐diaryl (**10a**, **10b**, **10d**, **10i**) and elongated hydroxylamine (**25a**, **25b**, **25d**, **25i**) scaffolds. In biochemical assays against recombinant human IDO1 and TDO, short‐chain thiols did not exhibit measurable inhibition at concentrations up to 10 μM, whereas elongated thiol analogs showed modest inhibition at this concentration, with a preference toward IDO1 over TDO. Overall, thiol derivatives were markedly less potent than hydroxylamines in enzymatic assays, underscoring the importance of the hydroxylamine group for productive heme coordination.

**FIGURE 2:**
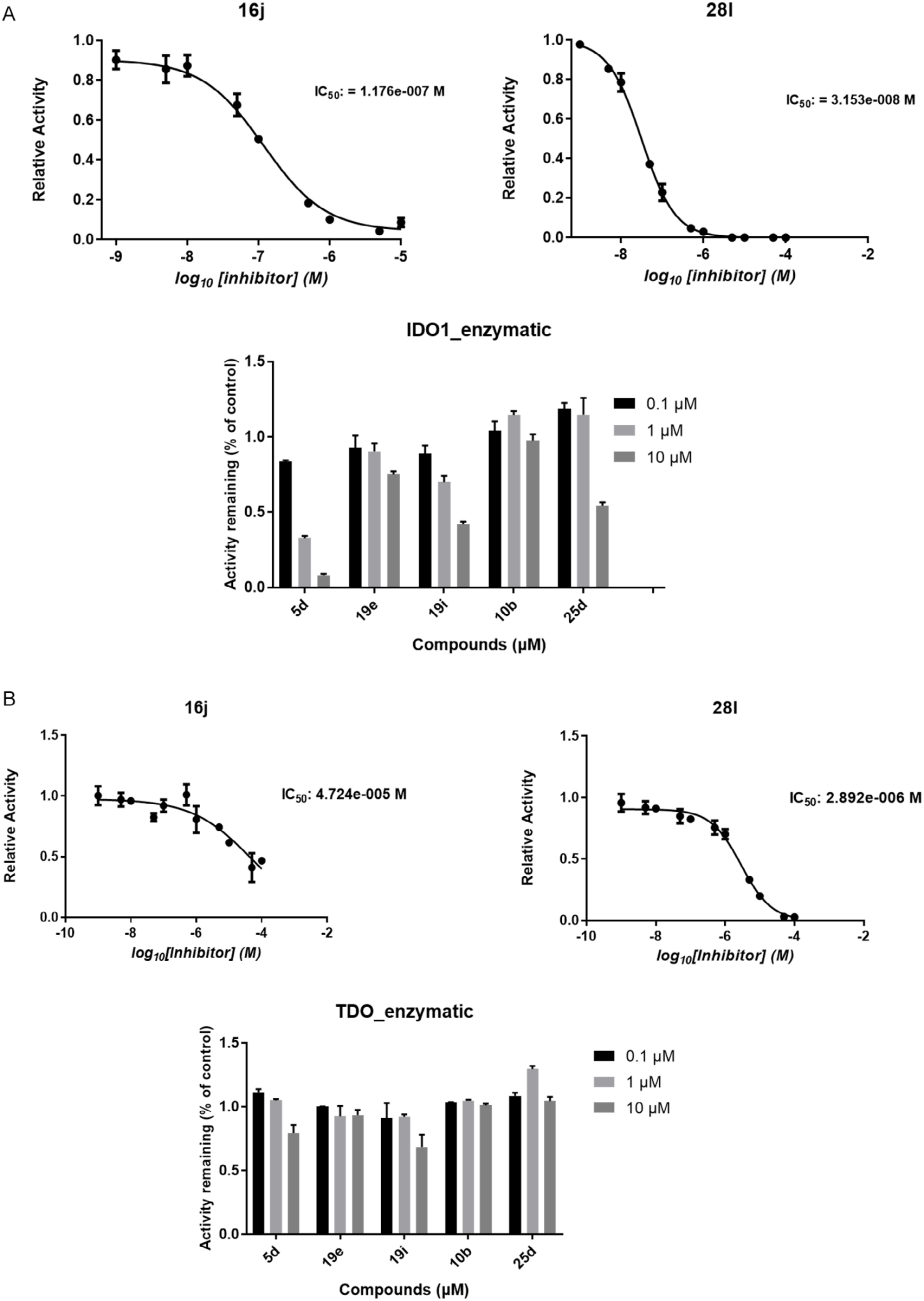
Enzymatic inhibition of recombinant human IDO1 and TDO by selected diaryl hydroxylamine derivatives (16j, 28l, 5d, 19e, 19i, 10b, and 25d). (A) IDO1 inhibition and (B) TDO inhibition. For the compounds, initial SAR profiling was performed at three fixed concentrations (0.1, 1, and 10 μM), showing residual enzymatic activity relative to vehicle control. In addition, full concentration–response curves were generated for compounds 16j and 28l using an 11‐point, half‐log serial dilution series (1 nM–100 μM). IC_50_ values were determined by nonlinear regression using a four‐parameter logistic model. Enzymatic activity was quantified by kynurenine formation using the p‐DMAB colorimetric assay. Data represent technical replicates.

For direct comparison across structural classes, the most representative compounds (**16j, 28l, 5d, 19e, 19i, 10b,** and **25d)** were evaluated in concentration‐dependent inhibition assays using recombinant human IDO1 and TDO (Figure 2). These compounds were selected because they exemplify the key structural determinants identified in this study, including short versus elongated scaffolds, heterocycle polarity, and iron‐binding functionality. The resulting curves highlight distinct potency and selectivity profiles, demonstrating how aromatic electron density, linker length, and pharmacophore identity collectively affect IDO1/TDO inhibition.

#### 2.2.2 Cell‐Based IDO1 and TDO Activity Assays

In general, most compounds displayed reduced potency in cellular assays compared with recombinant enzyme assays, consistent with the influence of cellular factors such as membrane permeability, subcellular localization, and metabolic stability. Among the class A compounds, compound **5d** retained measurable intracellular activity against both enzymes, exhibiting cellular IC_50_ values of 3 μM (IDO1) and 3.7 μM (TDO), while compounds **16j** and **16k** showed cellular IC_50_ values of 1.7–5.7 μM for IDO1 and 5.5 μM or > 10 μM for TDO.

Elongated hydroxylamines of class B largely failed to translate their enzymatic IDO1 activity into the cellular context, with most compounds exhibiting cellular IC_50_ values >10 μM. Notably, compound **19i** (2‐indolyl) represented an exception, displaying comparable activity in enzymatic and cellular IDO1 assays (∼5 μM and 2.8 μM, respectively).

Thiol derivatives showed limited cellular activity. In IFNγ‐stimulated HeLa cells, compound **25d** produced the highest reduction of kynurenine accumulation (∼10 μM), whereas compound **10b** exhibited the most pronounced effect in the TDO‐dependent A172 assay (∼10 μM). Overall, the reduced cellular activity of thiols likely reflects instability, oxidation, or limited cell permeability, further underscoring the importance of the hydroxylamine group for effective intracellular target engagement.

Finally, *O*‐benzylhydroxylamines exhibited strong enzymatic inhibition of IDO1 but generally weaker activity in cell‐based assays. An important exception was compound **28l**, which demonstrated potent inhibition in both enzymatic and cellular IDO1 assays (IC_50_ = 0.031 μM and 0.7 μM, respectively), while retaining measurable TDO inhibition (IC_50_ = 2.9 μM and 4.8 μM, respectively). These results identify the *O*‐benzylhydroxylamine scaffold as a promising framework for achieving robust IDO1 inhibition with retained TDO intracellular activity.

To evaluate intracellular target engagement and potency under physiologically relevant conditions, cellular IC_50_ values for **16j, 28l, 5d, 19e, 19i, 10b, and 25d** were determined in IFNγ‐stimulated HeLa cells (IDO1) and A172 glioblastoma cells (TDO). The resulting dose–response curves are depicted in Figure 3. The remaining compounds evaluated either by full dose–response analysis or by activity assessment at three fixed concentrations are provided in the Supporting Information (Figures S1–S4).

FIGURE 3Cellular inhibition of endogenous IDO1 and TDO by selected diaryl hydroxylamine derivatives (16j, 5d, 19e, 19i, 28l, 10b, and 25d). For all compounds, initial screening was performed at three fixed concentrations (0.1, 1, and 10 μM), and residual enzymatic activity relative to vehicle control is shown as bar plots. In addition, full concentration–response curves were generated for selected representative compounds to enable robust IC_50_ determination. Dose–response curves are shown for (A) IFNγ‐stimulated HeLa cells (IDO1) and (B) A172 glioblastoma cells (TDO; compounds 16j, 5d, 28l, and 10b). Cells were treated for 48 h with increasing concentrations of each inhibitor. Kynurenine accumulation in conditioned medium was quantified following p‐DMAB derivatization. IC_50_ values were determined by nonlinear regression using a four‐parameter logistic model. Data represent technical replicates.

**Figure:**
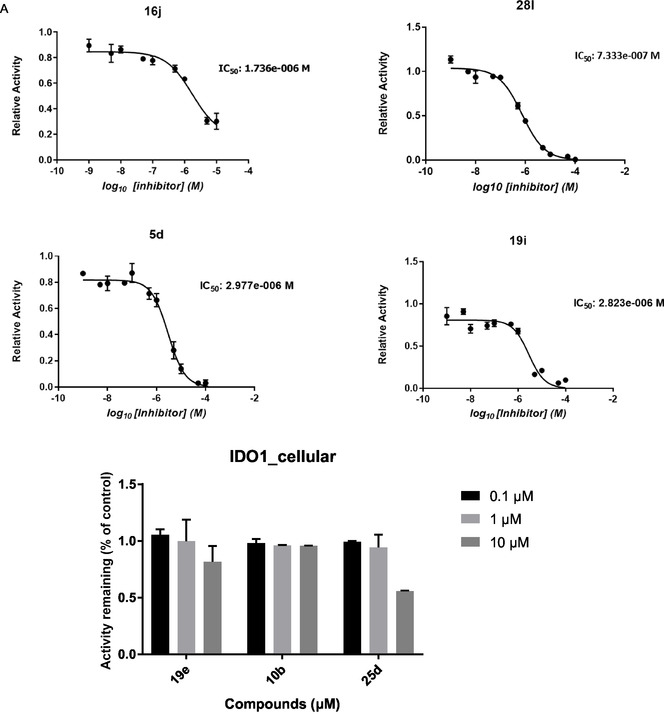

**Figure:**
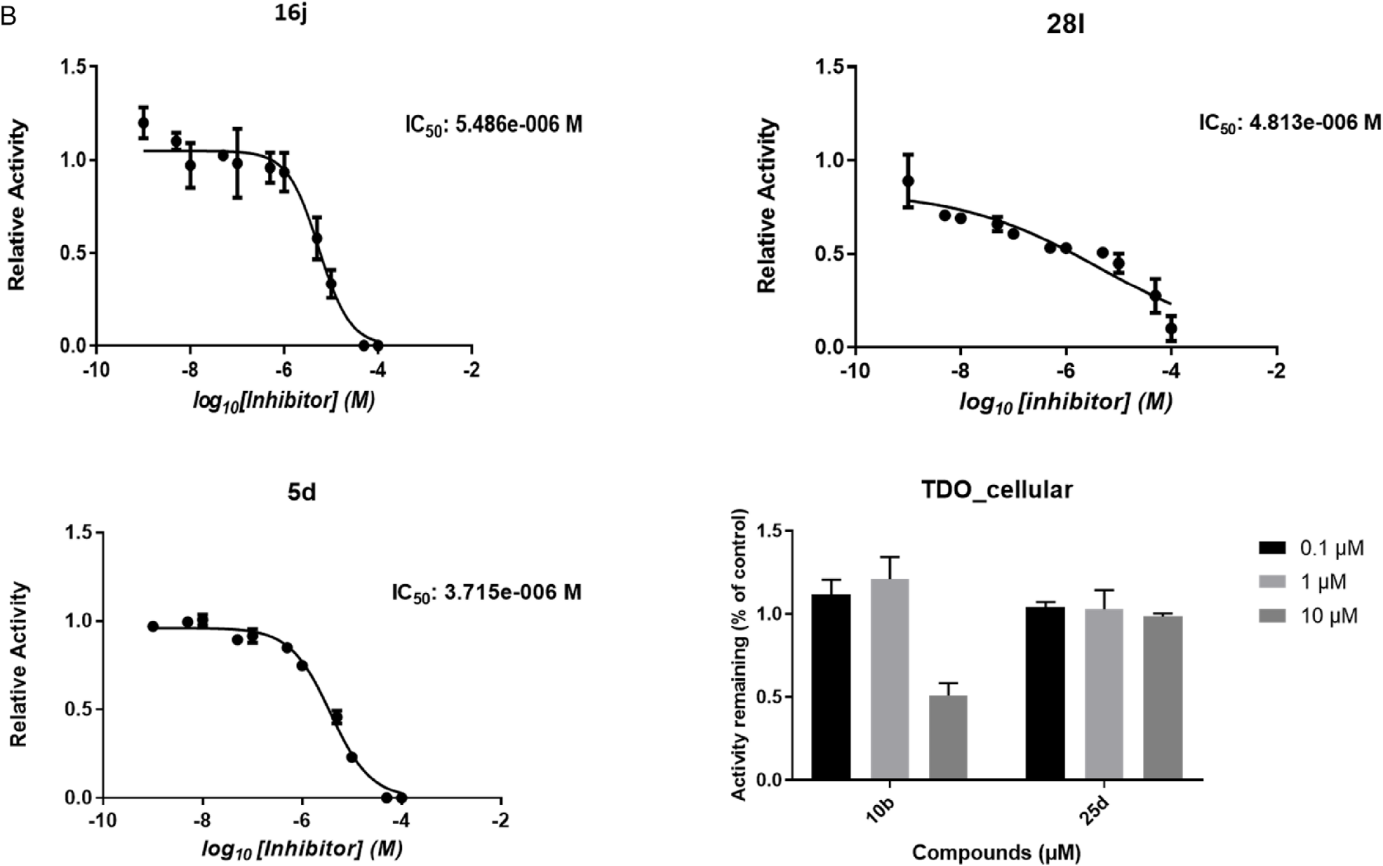

## 3 Conclusion

This study systematically explored key structural determinants of the diarylhydroxylamine scaffold aiming at potentiating its inhibitory capacity toward the IDO1 and TDO enzymes. Starting from a 3,5‐difluorophenyl lead compound previously identified as a potent dual inhibitor, we designed and synthesized different structural series exploring variations in the aryl framework, linker length, and the nature of the iron‐binding pharmacophore. The results demonstrate that maintaining a highly electron‐deficient aromatic framework is crucial for achieving potent IDO1/TDO inhibition, whereas even modest elongation of the inter‐aryl spacer promotes IDO1 selectivity. Bioisosteric replacement of the hydroxylamine group with alternative iron‐binding functionalities (oxime, thiol) did not improve potency, underscoring the essential role of the hydroxylamine moiety in effective heme coordination. Among all tested compounds, *O*‐benzylhydroxylamine **28l** and 1,1′‐diarylhydroxylamine **16j** displayed the most potent dioxygenase inhibitory profiles and represent promising lead candidates for further optimization.

Collectively, these findings provide new insights into the structure–activity relationships governing IDO1/TDO inhibition and establish a foundation for the rational design of multifunctional modulators based on the diarylhydroxylamine scaffold. In addition to their therapeutic relevance, selected compounds identified in this study may serve as suitable starting points for the future development of ^18^F‐radiolabeled derivatives to enable in vivo investigation of kynurenine pathway activity. Future work will focus on potentiating IDO1/TDO inhibition through computational and structural approaches, as well as exploring the PET imaging potential of optimized derivatives.

## 4 Experimental

### 4.1 Materials and Methods

All reactions were carried out with magnetic stirring, and if moisture or air‐sensitive, under an argon atmosphere using standard Schlenk techniques in oven‐dried (120°C oven temperature) or flame‐dried glassware. All reagents and starting materials were purchased from commercial suppliers (Fluorochem, Alfa Aesar, Sigma‐Aldrich, Merck, etc.) and used without further purification. Anhydrous CH_2_Cl_2_ was obtained by distillation from calcium hydride under argon. Anhydrous THF was freshly distilled from Na and benzophenone ketyl. All nonaqueous reactions were performed under an inert atmosphere of argon. Concentrated to dryness refers to the removal of solvent with a rotary evaporator at normal water aspirator pressure, followed by further evacuation on a high‐vacuum line. Thin‐layer chromatography was performed using silica gel 60 Å precoated aluminum or glass‐backed plates (0.25 mm thickness) with fluorescent indicators. Additional plates included precoated normal‐phase silica gel aluminum sheets (Silica gel 60 F254, Merck, 0.2 mm) and precoated aluminum oxide plates (TLC Aluminium oxide 60 F254, neutral). Developed TLC plates were visualized with UV light (254 nm), iodine vapors, or anisaldehyde staining solution. The chromatographic purification of the products was carried out using Fluka silica gel 60 for preparative column chromatography (particle size 40–63 μm).

NMR spectra were obtained in CDCl_3_, DMSO‐*d*_6_, MeOD, or D_2_O at 25°C on using Bruker Avance DRX 250, 400, 500 MHz spectrometers, and Bruker Ultrashield™ Plus Avance III 600 MHz. The measured chemical shifts are reported in δ (ppm), and the residual solvent signal was used as the internal calibration standard (CDCl_3_): ^1^H = 7.26 ppm, ^13^C = 77.18 ppm); (DMSO‐*d*_6_): ^1^H = 2.50 ppm, ^13^C = 39.51 ppm; (MeOD): ^1^H = 3.31 ppm, ^13^C = 49.00 ppm; (D_2_O): ^1^H = 4.79 ppm, ^13^C = 39.51 ppm with tetramethylsilane or solvent as internal standard. ^13^C‐NMR spectra were obtained with complete proton decoupling. Data of NMR spectra were recorded as follows: s = singlet, d = doublet, t = triplet, q = quartet, dd = doublet of doublets, dt = doublet of triplets td = triplet of doublets, tt = triplet of triplets, ddd = doublet of doublet of doublets, m = multiplet and brs = broad signal. Coupling constants (*J*) are reported in Hertz (Hz). Two‐dimensional experiments ^1^H–^1^H (COSY) and ^1^H^13^C (HSQC, HMBC) were performed for peak assignment. Data processing, including Fourier transformation, baseline correction, phasing, peak picking, and integrations, was performed using MestReNova software (v.12.0.0 and v.15.0.0) and TopSpin 4.5.0. Elemental analyses (C, H, N) were performed at the NCSR Demokritos, Greece with deviations ≤ ±0.4% from theoretical values, confirming ≥95% purity of the compounds.

### 4.2 Chemistry

The final step of the synthesis of the most potent compounds from each class is presented below. Compound 28l was synthesized as already reported [26].

#### 4.2.1 General Procedure for the Synthesis of Hydroxylamines 5d, 16j, 19e, 19i

Hydrazine monohydrate 55% aq. solution (2 eq.) or aqueous methylamine 40% (10 eq.) were added dropwise in a solution (0.1 M) of the corresponding phthalimide (1 eq.) in the appropriate mixture of solvents (see SI) at room temperature. For **5d** and **16j**, after stirring for 60–120 min, ether was added and the reaction was left in the freezer for 30 min. The white precipitate was filtered off, washed with cold EtOH, and the filtrate was evaporated to dryness. For **19e** and **19i,** the reaction was stirred until completion as confirmed by TLC, and the volatiles were removed under reduced pressure with the addition of ethanol. The crude hydroxylamines were purified by FCC using an appropriate system of hexanes/AcOEt with 1% Et_3_N.

##### 4.2.1.1 *O*‐((3,5‐difluorophenyl)(thiazol‐2‐yl)methyl)hydroxylamine (5d)

was synthesized from **4d** (0.1 g, 0.27 mmol). Purified by FCC using hexanes/AcOEt/Et_3_N 8:2:0.1; Light yellow oil (0.059 g, 91%); R_*f*_ = 0.3 (hexanes/AcOEt 7:3); ^1^H NMR (500 MHz, CDCl_3_) δ: 7.77 (d, ^3^ *J*_H−H_ = 3.2 Hz, 1H, thiazoleH9), 7.36 (d, ^3^ *J*_H−H_ = 3.3 Hz, 1H, thiazoelH10), 6.98 (d, ^3^ *J*_H−F_ = 5.9 Hz, 2H, ArH2/H6), 6.76 (tt, ^3^ *J*_H−F_ = 9.0, ^4^ *J*_H−H_ = 2.4 Hz, 1H, ArH4), 5.93 (s, 1H, H*7*), 5.74 (brs, 2H, –ONH_2_); ^13^C NMR (126 MHz, CDCl_3_) δ: 169.73 (thiazoleC8), 163.22 (dd, ^1^ *J*_C−F_ = 249.7, ^3^ *J*_C−F_ = 12.6 Hz, ArC3/C5), 142.99 (thiazoleC9), 142.86 (t, ^3^ *J*_C−F_ = 8.4 Hz, ArC1), 120.01 (thiazoleC10), 110.29, (m, AXX’‐system, ArC2/C6), 104.03 (t, ^2^ *J*_C−F_ = 25.3 Hz, ArC4), 84.90 (C7); Elemental analysis calcd. (%) for C_10_H_8_F_2_N_2_OS:C, 49.58; H, 3.33; N, 11.56; found:C, 49.47; H, 3.48; N, 11.29

##### 4.2.1.2 O‐((2‐fluoropyridin‐4‐yl)(phenyl)methyl)hydroxylamine HCl salt (16j)

was synthesized from **15j** (98 mg, 0.28 mmol). Purified by FCC using hexanes/AcOEt/Et_3_N 8:2:0.1. The free base was dissolved in dry Et_2_O (50 mL) and the resulting solution was cooled to −30°C. A solution of hydrogen chloride in Et_2_O (1.0 M) was added dropwise under stirring until the pH of the mixture reached ≈2. A precipitate formed upon acidification, and the reaction flask was stored in a freezer for 30 min to ensure complete precipitation. The resulting solid was collected by vacuum filtration, washed thoroughly with cold dry Et_2_O (3 × 10 mL), and dried under high vacuum for 3 h to afford the hydrochloride salt as an off‐white solid (56 mg, 78%); R_*f*_ = 0.3 (free‐base hexanes/AcOEt 7:3); ^1^H NMR (500 MHz, D_2_O) δ: 8.21 (d, ^3^ *J*_H−H_ = 5.4 Hz, 1H, ArH6), 7.51 (q, *J* = 2.3 Hz, 5H, PhH), 7.38 (d, ^3^ *J*_H−F_ = 5.4 Hz, 1H, ArH3), 7.20 (s, 1H, ArH5), 6.23 (brs, 1H, H7); ^13^C NMR (63 MHz, D_2_O) δ: 163.68 (d, ^1^ *J*_C−F_ = 239.9 Hz, ArC2), 153.62 (d, ^3^ *J*_C−F_ = 7.6 Hz, ArC6), 147.50 (d, ^3^ *J*_C−F_ = 13.1 Hz, ArC4), 135.38 (PhC8), 130.24 (PhC11), 129.44 (PhC10/12), 128.07 (PhC9/13), 119.66 (d, ^4^ *J*_C−F_ = 3.9 Hz, ArC6), 107.58 (d, ^2^ *J*_C−F_ = 37.5 Hz, ArC4), 85.56 (d, ^4^ *J*_C−F_ = 3.0 Hz, C7); Elemental analysis calcd. (%) for C_12_H_12_ClFN_2_O:C, 56.59; H, 4.75; N, 11.00; found:C, 56.31; H, 5.01; N, 10.73.

##### 4.2.1.3 *N*‐(2‐(aminooxy)‐2‐(3,5‐difluorophenyl)ethyl)oxazole‐4‐carboxamide (19e)

was synthesized from **18e** (90 mg, 0.22 mmol); off‐yellow solid (33 mg, 46%); R_*f*_ = 0.3 (hexanes/AcOEt 5:5); ^1^H NMR (250 MHz, DMSO‐d_6_,) δ: 8.61 (s, 1H, oxazoleH11), 8.51 (s, 1H, oxazoleH10), 8.20 (t, ^3^ *J*_H−H_ = 5.78 Hz, NH), 7.15–6.99 (m, 3H, ArH2/H4/H6), 6.21 (s, 2H, O–NH_2_), 4.70 (t, ^3^ *J*_H−H_ *=* 5.7 Hz, 1H, H7), 3.49 (dd, ^3^ *J*_H−H_ = ^3^ *J*_H−H_ 5.68 Hz, 2H, H8); ^13^C NMR (63 MHz, DMSO‐d_6_) δ: 162.24 (dd, ^1^ *J*_C−F_ = 245.3 Hz, ^3^ *J*_C−F_ = 12.72 Hz, ArC3/C5), 159.81(C = O), 152.28 (oxazoleC11), 145.39 (ArC1), 141.88 (oxazoleC10), 135.38 (oxazoleC9), 109.83 (m, AXX’‐system, ArC2/C6), 102.73 (t, ^2^ *J*_C−F_ = 25.96 Hz, ArC1), 82.71 (C7), 42.40 (C8); Elemental analysis calcd. (%) for C_12_H_11_F_2_N_3_O_3_: C, 50.89; H, 3.91; N, 14.84; found:C, 51.16; H, 4.09; N, 15.13.

##### 4.2.1.4 *N*‐(2‐(aminooxy)‐2‐(3,5‐difluorophenyl)ethyl)‐1*H‐*indole‐2‐carboxamide (19i)

was synthesized from **18i** (99 mg, 0.22 mmol); pink solid (41 mg, 58%); R_*f*_ = 0.4 (hexanes/AcOEt 6:4); ^1^H NMR (250 MHz, DMSO‐d_6_) δ: 11.57 (s, 1H, indoleN*H*), 8.55 (t, ^3^ *J*_H−H_ = 5.8 Hz, 1H, NH), 7.60 (d, ^3^ *J*_H−H_ = 7.9 Hz, 1H, indoleH12), 7.41 (d, ^3^ *J*_H−H_ = 8.2 Hz, 1H, indole*H15*), 7.23–6.97 (m, 6H, indoleH10/H13/H14, ArH1/H3/H5), 6.22 (s, 2H, O–NH_2_), 4.71 (t, ^3^ *J*_H−H_ = 5.8 Hz, 1H, H7), 3.54 (dd, ^3^ *J*_H−H_ =^3^ *J*_H−H_ = 5.8, 2H, H8); ^13^C NMR (63 MHz, DMSO‐d_6_) δ: 162.28 (dd, ^1^ *J*_C−F_ = 245.8 Hz, ^3^ *J*_C−F_ = 13.0 Hz, ArC3/C5), 161.16 (C = O), 145.67 (t, ^3^ *J*_C−F_ = 8.5 Hz, ArC1), 136.42 (indoleC), 131.51 (indoleC), 127.05 (indoleC), 123.31 (indoleC), 121.51 (indole*C12*), 119.72 (indoleC), 112.30 (indole*C15*), 109.86 (m, AXX’‐system, ArC2/C6), 102.74 (t, ^2^ *J*_C−F_ = 25.8 Hz, ArC4), 102.64 (indoleC), 83.24 (C7), 42.97 (C8); Elemental analysis calcd. (%) for C17H15F2N3O2:C, 61.63; H, 4.56; N, 12.68; found:C, 61.49; H, 4.71; N, 12.53

#### 4.2.2 General Procedure for the Synthesis of Thiols 10b, 25d

The corresponding thioester was dissolved in abs. EtOH (0.12 M) and the solution was degassed by applying the freeze‐pump‐thaw technique. A solution of NaOH 1 M (1.2 eq) was then added dropwise at 0°C, and the reaction was allowed to stir at that temperature until TLC confirmed full consumption of the SM (usually 5–60 min). The solution was then neutralized by the dropwise addition of HCl 1 M (1.25 eq) at 0°C, and the reaction was allowed to reach room temperature. The volatiles were removed under reduced pressure with the addition of ethanol and the crude mixture was purified by FCC using the appropriate hexanes/AcOEt system.

##### 4.2.2.1 (3,5‐difluorophenyl)(furan‐2‐yl)methanethiol (10b)

was synthesized from **9b** (0.126 g, 0.47 mmol) according to the general procedure I. Purified by FCC using hexanes/AcOEt 100:0 to 98:2; Pale yellow oil (82 mg, 77%); R_*f*_ = 0.2 (hexanes/AcOEt 98:2; ^1^H NMR (500 MHz, CDCl_3_) δ: 7.39 (s, 1H, furaneH11), 6.96–6.92 (m, 2H, ArH2/H6), 6.74–6.70 (m, 1H, ArH4), 6.34–6.33 (brs, 1H, furaneH10), 6.26–6.6.25 (m, 1H, furanH9), 5.26 (d, *J* = 6.1 Hz, 1H, H7), 2.49 (d, *J* = 6.1 Hz, 1H, –SH); ^13^C NMR (126 MHz, CDCl_3_) δ: 163.16 (dd, ^1^ *J*_C−F_ = 249.1 Hz, ^3^ *J*_C−F_ = 12.5 Hz, ArC3/C5), 153.98 (furaneC8), 145.29 (t, ^3^ *J*_C−F_ = 8.9 Hz, ArC1), 142.90 (furaneC11), 110.91 (m, AXX’‐system, ArC2/C6), 110.65 (furaneC10), 107.93 (furaneC9), 103.34 (t, ^2^ *J*_C−F_ = 25.4 Hz, ArC4), 44.49 (C7); Elemental analysis calcd. (%) for C_11_H_8_F_2_OS:C, 58.40; H, 3.56; found:C, 58.23; H, 3.81.

##### 4.2.2.2 *N*‐(2‐(3,5‐difluorophenyl)‐2‐mercaptoethyl)thiazole‐2‐carboxamide (25d)

was synthesized from **24d** (0.106 g, 0.31 mmol) according to the general procedure I. The product was purified by FCC using hexanes/AcOEt 7:3; white solid (49.2 mg, 53%); R_*f*_ = 0.4 (hexanes/AcOEt 7:3); ^1^H NMR (500 MHz, CDCl_3_) δ: 7.85 (d, *J* = 3.0 Hz, 1H, thiazoleH12), 7.59 (d, *J* = 3.0 Hz, 1H, thiazoleH11), 7.55 (brs, 1H, –NH), 6.95–6.89 (m, 2H, ArH2/H6), 6.74 (tt, ^3^ *J*_H−F_ = 8.8 Hz, ^4^ *J*_H−H_ = 2.3 Hz, 1H, ArH4), 4.29–4.22 (m, 1H, H7), 3.93–3.86 (m, 1H, H8a), 3.82–3.74 (m, 1H, H8b), 2.08 (d, *J *= 6.6 Hz, 1H, –SH); ^13^C NMR (126 MHz, CDCl_3_) δ: 163.35 (dd, ^1^ *J*_C−F_ = 249.6 Hz, ^3^ *J*_C−F_ = 12.4 Hz, ArC3/C5), 163.12 (C9), 159.78 (thiazoleC10), 145.10 (t, ^3^ *J*_C−F_ = 8.5 Hz, ArC1), 143.79 (thiazoleC12), 125.08 (thiazoleC11), 110.52 (m, AXX’‐system, ArC2/C6), 103.69 (t, ^2^ *J*_C−F_ = 24.9 Hz, ArC4), 47.27 (C7), 43.03 (C8); Elemental analysis calcd. (%) for C_12_H_10_F_2_N_2_OS_2_:C, 47.99; H, 3.36; N, 9.33; found:C, 47.86; H, 3.57; N, 9.17.

##### 4.2.2.3 Expression & purification of recombinant human dioxygenases IDO1 and TDO

N‐terminal His_6_‐tagged human IDO1 and TDO constructs in pET‐28a(+) vector were transformed and expressed in *E. coli* BL21(DE3) competent cells. Cultures were grown in kanamycin‐supplemented medium at 37°C to OD_600_ ∼ 0.5, supplemented with δ‐aminolevulinic acid (5‐ALA, 0.5 mM) to support heme incorporation, induced with IPTG (0.5 mM), shifted to 20°C, and expressed overnight. Cells were harvested by centrifugation at 4,000 rpm at 4°C, resuspended in Lysis buffer (25 mM Tris‐HCl pH 8.0, 300 mM NaCl, 10 mM *β*‐mercaptoethanol, lysozyme, and EDTA‐free protease inhibitors), incubated for 30 min on ice, and lysed by sonication. Lysates were clarified by centrifugation at 20,000 × g for 1 h (4°C). The supernatant was adjusted to 20 mM imidazole and batch‐bound to Ni^2+‐^NTA agarose pre‐equilibrated in binding buffer (25 mM Tris‐HCl pH 8.0, 300 mM NaCl, 20 mM imidazole) for 2 h at 4°C with gentle rotation. The resin was transferred to a gravity column, washed (5 bed volumes) with wash buffer (25 mM Tris‐HCl pH 8.0, 300 mM NaCl, 40 mM imidazole), and protein was eluted with elution buffer (25 mM Tris‐HCl pH 8.0, 300 mM NaCl, 300 mM imidazole) in  × 1 mL fractions. Fractions were collected and analyzed for purity by SDS‐PAGE. Protein concentrations were determined by A_280_ (NanoDrop) using the theoretical extinction coefficients calculated with ProtParam. Purified proteins were supplemented with 10% (v/v) glycerol, aliquoted and stored at −80°C until use.

##### 4.2.2.4 IDO1 enzymatic inhibition assay

[27] Reactions were assembled in clear, flat‐bottom 96‐well plates at 37°C in assay buffer composed of 50 mM potassium phosphate, pH 6.5, 10 μM methylene blue, 20 mM ascorbic acid, catalase 100 μg mL^−1^, and 0.01% (v/v) Triton X‐100. Recombinant human IDO1 (50 nM) and test compounds were preincubated for 15 min at room temperature, and reactions were initiated with the addition of L‐tryptophan 150 μM and run for 30 min under initial‐rate conditions. For the thiol series, DTT (10 μM) was included in all treatment and matched‐vehicle control wells.

Initial SAR profiling was performed at three fixed concentrations (0.1, 1, and 10 μM), and these data are presented as bar plots for comparative analysis. Full dose–response curves (11‐point, half‐log serial dilution) were generated for selected representative compounds to enable robust IC_50_ determination by nonlinear regression.

Reactions were quenched by adding 30 μL of 30% (w/v) trichloroacetic acid (TCA; final volume of reaction 150 μL), followed by incubation at 60°C for 30 min to convert N‐formyl‐kynurenine to kynurenine and precipitate protein. After clarification, 100 μL supernatant was transferred to a clear flat‐bottom plate and mixed 1:1 with 2% (w/v) *p*‐DMAB in glacial acetic acid. Reactions were incubated at room temperature for 15 min and absorbance was read at 490 nm on a Tecan infinite M200 microplate reader. Data were fitted by nonlinear regression using a four‐parameter logistic model in GraphPad Prism 8.0; initial data handling was performed in Excel.

##### 4.2.2.5 TDO enzymatic inhibition assay [28]

Conditions were identical to the IDO1 assay except for enzyme/substrate loads and hydrolysis time: recombinant TDO (200 nM) with inhibitors in the same buffer system was preincubated for 15 min at room temperature and initiated with L‐tryptophan 300 μM. For initial SAR screening across the full compound set, compounds were evaluated at three fixed concentrations (0.1, 1, and 10 μM; vehicle 0.1% DMSO).

Initial SAR profiling was performed at three fixed concentrations (0.1, 1, and 10 μM), and these data are presented as bar plots for comparative analysis. Full dose–response curves (11‐point, half‐log serial dilution) were generated for selected representative compounds to enable robust IC_50_ determination by nonlinear regression.

Reactions were quenched by adding 30 μL of 30% (w/v) trichloroacetic acid TCA (final volume of reaction 150 μL), followed by incubation at 60°C for 90 min to convert N‐formyl‐kynurenine to kynurenine and precipitate protein. After clarification, 100 μL supernatant was transferred to a clear flat‐bottom plate and mixed 1:1 with 2% (w/v) p‐DMAB in glacial acetic acid. Reactions were incubated at room temperature for 15 min and absorbance was read at 490 nm on a Tecan infinite M200 microplate reader, as above. Data were fitted by nonlinear regression using a four‐parameter logistic model in GraphPad Prism 8.0; initial data handling was performed in Excel.

##### 4.2.2.6 Cell lines and culture conditions

HeLa (ATCC CCL‐2) and A172 glioblastoma (ATCC CRL‐1620) cells were maintained at 37°C in a humidified 5% CO_2_ incubator in DMEM medium supplemented with 10% fetal bovine serum (FBS) and 2 mM L‐glutamine. Cells were passaged at 70%–80% confluence. For all plate‐based assays, phenol red‐free DMEM was used to avoid interference with absorbance readings. IDO1 expression in IFNγ‐stimulated HeLa cells and TDO expression in A172 cells were verified by Western blot analysis before performing the cellular IC_50_ assays to ensure robust enzyme levels under the conditions used.

##### 4.2.2.7 Cellular IDO1 activity assay [27, 28]

IDO1 activity was quantified as Kyn accumulation in conditioned medium of HeLa cells. 1 day prior to treatment, 0.8 × 10^4^ cells per well were seeded into 96‐well plates and allowed to adhere overnight. Cells were then stimulated with recombinant human IFNγ (25 ng/mL) to induce IDO1, and co‐treated for 48 h with test compounds

Initial SAR profiling was performed at three fixed concentrations (0.1, 1, and 10 μM), and these data are presented as bar plots for comparative analysis. Full dose–response curves (11‐point, half‐log serial dilution) were generated for selected representative compounds to enable robust IC_50_ determination by nonlinear regression.

For the thiol series, DTT (10 μM) was included in all treatments and matched vehicle wells to minimize the risk of oxidation. The assay was performed in complete phenol red‐free DMEM supplemented with L‐tryptophan adjusted to 100 μM to avoid potential high‐Trp substrate inhibition of IDO1. Medium‐only (no cells) controls were included to correct for background absorbance. After 48 h, 150 μL of supernatant from each well (of the 200 μL total) was collected, transferred to a new plate and mixed with 45 μL of 30% (w/v) trichloroacetic acid (TCA). Samples were incubated for 40 min at 60°C to convert residual N‐formyl‐kynurenine to Kynurenine and to precipitate proteins, and centrifuged for 10 min at 3,000 × g. Then, 100 μL of the clarified supernatant was transferred to a fresh flat‐bottom plate, combined 1:1 with 2% (w/v) p‐DMAB in glacial acetic acid (freshly prepared), and incubated for 30 min at room temperature protected from light. Absorbance was read at 490 nm on a Tecan infinite M200 microplate reader. Each condition was run in technical replicates. Data were fitted with a four‐parameter model in GraphPad Prism 8.0; initial data handling was performed in Excel.

##### 4.2.2.8 Cellular TDO activity assay [29]

TDO activity was quantified as Kyn accumulation in conditioned medium of A172 cells. 1 day prior to treatment, 1.5 × 10^4^ cells per well were seeded into 96‐well plates and allowed to adhere overnight. Cells were then treated for 48 h with test compounds.

Initial SAR profiling was performed at three fixed concentrations (0.1, 1, and 10 μM), and these data are presented as bar plots for comparative analysis. Full dose–response curves (11‐point, half‐log serial dilution) were generated for selected representative compounds to enable robust IC_50_ determination by nonlinear regression.

For the thiol series, DTT (10 μM) was included in all treatments and matched vehicle wells. The assay was performed in complete phenol red‐free DMEM supplemented with L‐tryptophan adjusted to 400 μM. Medium‐only (no cells) controls were included to correct for background absorbance. After 48 h, 150 μL of supernatant from each well (of the 200 μL total) was transferred to a new plate and mixed with 45 μL of 30% (w/v) TCA. Samples were incubated for 40 min at 60°C to convert residual N‐formyl‐kynurenine to Kynurenine and to precipitate proteins, and centrifuged for 10 min at 3,000 × g. Then, 100 μL of the clarified supernatant was transferred to a fresh flat‐bottom plate, combined 1:1 with 2% (w/v) p‐DMAB in glacial acetic acid (freshly prepared), and incubated for 30 min at room temperature protected from light. Absorbance was read at 490 nm on a Tecan infinite M200 microplate reader. Each condition was run in technical replicates. Data were fitted with a four‐parameter model in GraphPad Prism 8.0; initial data handling was performed in Excel.

## Supporting Information

Additional supporting information can be found online in the Supporting Information section. **Supporting** **Figure S1.** Enzymatic inhibition of recombinant human IDO1 by additional diaryl hydroxylamine derivatives. Compounds are grouped into classes A–E as defined in the main manuscript; class C is further subdivided into C‐1 (oximes and short thiols) and C‐2 (long thiols). IDO1 activity was assessed under initial‐rate conditions by quantifying kynurenine formation using the p‐dimethylaminobenzaldehyde (p‐DMAB) colorimetric assay. For selected compounds, full concentration–response curves were generated using serial dilutions and used for IC_50_ determination by nonlinear regression (four‐parameter logistic model). In parallel, the broader compound set was evaluated as part of initial SAR screening at three fixed concentrations (0.1, 1, and 10 µM), and results are presented as bar plots showing relative activity remaining compared to vehicle‐treated controls. Data are normalized to vehicle control (control = 1) and expressed as fraction of control. Data represent independent experiments performed in technical replicates. **Supporting** **Figure S2.** Enzymatic inhibition of recombinant human TDO by additional diaryl hydroxylamine derivatives: Compounds are grouped into classes A–E as defined in the main manuscript; class C is further subdivided into C‐1 (oximes and short thiols) and C‐2 (long thiols). Enzymatic inhibition of recombinant human TDO was assessed under initial‐rate conditions by quantifying kynurenine formation using the p‐dimethylaminobenzaldehyde (p‐DMAB) colorimetric assay. For selected compounds, full concentration–response curves were generated using serial dilutions and used for IC_50_ determination by nonlinear regression (four‐parameter logistic model). In parallel, additional compounds listed in Table 1 were evaluated as part of initial SAR screening at three fixed concentrations (0.1, 1, and 10 µM), and results are presented in bar plots as relative activity remaining compared to vehicle‐treated controls. Experimental conditions are as described for Figure S1. Data are normalized to vehicle control (control = 1) and expressed as fraction of control. Data represent independent experiments performed in technical replicates. **Supporting** **Figure S3.** Cellular inhibition of endogenous IDO1 activity in IFNγ‐stimulated HeLa cells. Compounds are grouped into classes A–E as defined in the main manuscript; class C is further subdivided into C‐1 (oximes and short thiols) and C‐2 (long thiols). Endogenous IDO1 activity was evaluated by quantifying kynurenine accumulation in conditioned medium after 48 h of treatment using the p‐dimethylaminobenzaldehyde (p‐DMAB) derivatization assay. For selected compounds, full cellular concentration–response curves were generated using serial dilutions and used for IC_50_ determination by nonlinear regression (four‐parameter logistic model). In parallel, initial SAR screening across the broader compound set was performed at three fixed concentrations (0.1, 1, and 10 µM) and is presented as bar plots for comparative profiling. Data are normalized to vehicle control (control = 1) and expressed as fraction of control. Data represent technical replicates from independent experiments. **Supporting** **Figure S4.** Cellular inhibition of endogenous TDO activity in A172 glioblastoma cells: Compounds are grouped into classes A–E as defined in the main manuscript; class C is further subdivided into C‐1 (oximes and short thiols) and C‐2 (long thiols). Endogenous TDO activity was evaluated by quantifying kynurenine accumulation in conditioned medium after 48 h of treatment using the p‐dimethylaminobenzaldehyde (p‐DMAB) derivatization assay. Compounds were evaluated at three fixed concentrations (0.1, 1, and 10 µM) as part of initial SAR screening and are presented as bar plots for comparative profiling. Experimental conditions were identical to those described in Figure S3. Data represent technical replicates from independent experiments. Data are normalized to vehicle control (control = 1) and expressed as fraction of control.

## Funding

Part of this work was supported by the European Social Fund (ESF) through the Operational Programme “Human Resources Development, Education and Lifelong Learning 2014–2020” in the context of the project “Targeting the kynurenine pathway for tumor imaging and characterization by single‐photon emission computed tomography (SPECT) and positron emission tomography (PET)” (MIS 5047830). Prof. G. Zoidis would like to thank Gilead ASKLEPIOS Grants Program for the financial support.

## Conflicts of Interest

The authors declare no conflicts of interest.

## Supporting information

Supplementary Material

## Data Availability

The data that support the findings of this study are available from the corresponding author upon reasonable request.
