# Supplementary material for: Modulating IDO1 and TDO Inhibition Through Structural Modification of Diaryl Hydroxylamines
Source: ChemMedChem. 2026 Apr 7;21(7):e202501087. doi: 10.1002/cmdc.202501087 (PMC13056353; doi:10.1002/cmdc.202501087)
Supplement: Supplementary file 1 — Supplementary Material [file CMDC-21-e202501087-s001.pdf]

Supporting Information

Modulating IDO1 and TDO Inhibition Through Structural Modification of Diaryl Hydroxylamines

Angeliki S. Foscolos, Alexandros Pappas, Christos N. Petroulias, Anna Kapella, Georgia M. Prifti, Grigoris Zoidis, Minas Papadopoulos, Anastasia Mpakali* and Aristeidis Chiotellis*

A. S. Foscolos, A. Pappas, C. Petroulias, A. Kapella, M. Papadopoulos, A. Mpakali, A. Chiotellis
Institute of Nuclear & Radiological Sciences & Technology, Energy & Safety
National Center for Scientific Research "Demokritos"
Patriarchou Grigoriou & Neapoleos 27, 15310, Athens, Greece

G. M Prifti, G. Zoidis
Division of Pharmaceutical Chemistry, Department of Pharmacy, School of Health Sciences
National and Kapodistrian University of Athens
Zografou University campus, 15771 Athens, Greece

Corresponding author:
*Aristeidis Chiotellis
email: achiotel@rrp.demokritos.gr
* Anastasia Mpakali
email: ampakali@gmail.com

Table of Contents

Chemistry 2
Screening-Level Inhibition Profiles and Concentration–Response Curves 38
A. Enzymatic Data 38
B. Cellular Data 45
^{1}H and ^{13}C NMR spectra 49
References 78

Chemistry

Materials and Methods

All reactions were carried out with magnetic stirring, and if moisture or air sensitive, under argon atmosphere using standard Schlenk techniques in oven-dried (120 °C oven temperature) or flame-dried glassware. All reagents and starting materials were purchased from commercial suppliers (Alfa Aesar, Sigma-Aldrich, Merck) and used without further purification. Anhydrous CH_2Cl_2 was obtained by distillation from calcium hydride under argon. Anhydrous THF was freshly distilled from Na and benzophenone ketyl. All non-aqueous reactions were performed under an inert atmosphere of argon. Concentrated to dryness refers to the removal of solvent with a rotary evaporator at normal water aspirator pressure, followed by further evacuation on a high-vacuum line. Thin-layer chromatography was performed using silica gel 60 Å precoated aluminum or glass-backed plates (0.25 mm thickness) with fluorescent indicators. Additional plates included precoated normal-phase silica gel aluminum sheets (Silica gel 60 F254, Merck, 0.2 mm), and precoated aluminum oxide plates (TLC Aluminium oxide 60 F254, neutral). Developed TLC plates were visualized with UV light (254 nm), iodine vapors, or anisaldehyde staining solution. The chromatographic purification of the products was carried out using Fluka silica gel 60 for preparative column chromatography (particle size 40–63 μm).

NMR spectra were obtained in $CDCl_3$, DMSO-d_6, MeOD or D_2O at 25 °C on using Bruker Avance DRX 250, 400, 500 MHz spectrometers, and Bruker Ultrashield™ Plus Avance III 600 MHz. The measured chemical shifts are reported in δ (ppm), and the residual solvent signal was used as the internal calibration standard ($CDCl_3$): ^{1}H = 7.26 ppm, ^{13}C = 77.18 ppm); (DMSO-d_6): ^{1}H = 2.50 ppm, ^{13}C = 39.51 ppm; (MeOD): ^{1}H = 3.31 ppm, ^{13}C = 49.00 ppm; (D_2O): ^{1}H = 4.79 ppm, ^{13}C = 39.51 ppm with tetramethylsilane or solvent as internal standar. ^{13}C-NMR spectra were obtained with complete proton decoupling. Data of NMR spectra were recorded as follows: s = singlet, d = doublet, t = triplet, q = quartet, dd = doublet of doublets, dt = doublet of triplets td = triplet of doublets, tt = triplet of triplets, ddd = doublet of doublet of doublets, m = multiplet and brs = broad singlet. Coupling constants (*J*) are reported in Hertz (Hz). Two-dimensional experiments ^{1}H-^{1}H (COSY) and ^{1}H-^{13}C (HSQC, HMBC) were performed for peak assignment. Data processing, including Fourier transformation, baseline correction, phasing, peak picking, and integrations, was performed using MestReNova software (v.12.0.0 and v.15.0.0) and TopSpin 4.5.0. Elemental analyses (C, H, N) were carried out at the NCSR Demokritos, Greece, and Service Central de Microanalyse, CNRS, France, with deviations ≤ ±0.4% from theoretical values, confirming ≥95% purity of the compounds.

Additional Synthetic Details and Observations

The aryl Grignard reagents were prepared from the corresponding aryl bromides (phenyl bromide or 3,5-difluorophenyl bromide) in anhydrous THF following standard procedures and were reacted with the appropriate heteroaryl-2-carbaldehydes to afford the corresponding secondary alcohols in good to excellent yields. Alcohols 14j and 14k were obtained by reacting phenylmagnesium bromide with 2-fluoroisonicotinaldehyde and 2-fluoro-4-formylbenzonitrile, respectively.

For the hydroxylamine series, the secondary alcohols were subjected to Mitsunobu conditions using N-hydroxysuccinimide, affording the corresponding *N*-hydroxyphthalimide intermediates in good yields. In several cases, the hydrazine-1,2-dicarboxylate Mitsunobu reagent byproduct generated during the reaction co-eluted with the desired phthalimide during flash column chromatography and could not be fully separated at this stage. In a limited number of cases, recrystallization from ethanol afforded analytically pure phthalimides; however, in most instances this impurity was efficiently removed during the subsequent hydrazinolysis step. Hydrazinolysis of the phthalimides furnished the final hydroxylamines in good yields.

Most hydroxylamines were isolated and stored as free bases at −25 °C. Attempts to generate hydrochloride salts using HCl (1 M in ether) occasionally resulted in partial decomposition of the compounds, although several derivatives could be isolated as stable hydrochloride salts, as detailed in the Experimental Section. It should be noted that hydroxylamines bearing 2-furanoyl, 2-pyrrolyl, or 2-indolyl heteroaromatic rings were found to be highly unstable and could not be reliably handled or evaluated in vitro.

For the oxime series, oxidation of the secondary alcohols to the corresponding ketones was performed using MnO_2 in dichloromethane, affording the ketones in excellent to quantitative yields. Condensation with hydroxylamine in refluxing ethanol yielded the corresponding oximes, typically obtained as E/Z mixtures. While separation of the isomers by flash column chromatography was feasible in some cases, the mixtures were generally used directly for biological evaluation.

In the thiol series, initial attempts to convert the alcohols into mesylate intermediates using methanesulfonyl chloride and triethylamine in dichloromethane at 0 °C were unsuccessful, resulting predominantly in formation of the corresponding chlorides. This behavior is attributed to the high SN1 reactivity of diaryl mesylates, which likely undergo rapid solvolysis and halide substitution in the presence of chloride ions. Consequently, chlorides were generated either directly during these reactions or alternatively using thionyl chloride in dichloromethane. The resulting chlorides were generally of sufficient purity to be used directly in subsequent steps without further purification.

Conversion of the chlorides to the corresponding thioacetates was achieved by reaction with potassium thioacetate in DMF, affording the thioacetate intermediates in moderate to good yields (45–75%). For alcohols that proved unstable under chlorination conditions, thioacetates were instead prepared via Mitsunobu reaction using thioacetic acid. Hydrolysis of the thioacetates under basic conditions (NaOH) afforded the free thiols, which were purified by flash column chromatography.

Short-chain thiols were prone to oxidative dimerization to the corresponding disulfides and were therefore stored under an inert atmosphere at −25 °C. Compound 10f underwent rapid dimerization under the conditions of the in vitro assays and was therefore excluded from biological testing. In contrast, long-chain thiol derivatives displayed significantly improved stability toward oxidative dimerization.

The synthesis of long-chain hydroxylamine derivatives followed previously reported procedures that successfully afforded compounds 18b, 18d, and 18h[1]. The key intermediate, 2-(2-amino-1-(3,5-difluorophenyl)ethoxy)isoindoline-1,3-dione, was prepared as described and coupled with the appropriate heteroaryl-2-carboxylic acids using HBTU/DIPEA. The resulting phthalimides were subjected directly to hydrazinolysis with either aqueous hydrazine or methylamine to afford the desired hydroxylamines in good yields.

For the long thiol series, key intermediate 20[1] was coupled with the appropriate heteroaryl-2-carboxylic acids using EDC/DIPEA in anhydrous dichloromethane, except for compound 21i, which required TBTU/DIPEA in DMF due to limited solubility of the substrate acid in dichloromethane. Reduction of the resulting ketones to the corresponding alcohols using $NaBH_4$ in ethanol proceeded in nearly quantitative yields. These alcohols were converted to the corresponding chlorides using $SOCl_2$/DMF or MsCl/Et_3N, followed by reaction with potassium thioacetate and subsequent basic hydrolysis to afford the free thiols.

O-substituted hydroxylamines were prepared following previously reported two-step protocols[2], involving alkylation of N-hydroxyphthalimide with the appropriate benzyl bromides in the presence of sodium hydride in DMF, followed by hydrazinolysis. Etherified hydroxylamines were synthesized via a reported four-step sequence [15], consisting of Williamson ether formation from 4-hydroxybenzyl alcohol, chlorination with thionyl chloride under solvent-free conditions, substitution with N-hydroxyphthalimide, and final hydrazinolysis to afford the target hydroxylamines.

Grignard reagents preparations

Preparation of (3,5-difluorophenyl)magnesium bromide (2)

Mg turnings (2.92 g, 0.12 mol, 1.2 eq.) and a few crystals of I_2 were ground together in a mortar, transferred to a 2-neck 250 ml round-bottom flask and were suspended in THF (70 mL). Then, a few ml of a solution of 1-bromo-3,5-difluorobenzene (19.3 g, 11.51 ml, 0.1 mol, 1 eq.) in THF (18.5 mL) were added in the mixture which was gently heated with a Bunsen flame until initiation of the reaction (color of I_2 disappears). The reaction was kept under gentle reflux by carefully adding the rest of the bromide solution in a continuous way. After the addition was complete, the reaction was heated to reflux for an additional 90 min and was allowed to reach room temperature. The stock solution was kept in the refrigerator under Argon and it was stable for more than a month to be used for Grignard reactions. *Note* that the solution was not titrated and for the subsequent reactions was treated as a 1 M solution of (3,5-difluorophenyl)magnesium bromide based on the amounts used and assuming full conversion of the reagents.

Preparation of phenylmagnesium bromide 1M (13)

Mg turnings (1.35 g, 0.056 mol 1.12 eq.) and a few crystals of I_2 were ground together in a mortar, transferred to a 2-neck 100 ml round-bottom flask and were suspended in anh. THF (30 mL). Then, a few ml of a solution of bromobenzene (5.25 ml, 0.05 mol, 1 eq.) in anh. THF (20 mL) were added in the mixture which was gently heated with a Bunsen flame until initiation of the reaction (color of I_2 disappears). The reaction was kept under gentle reflux by carefully adding the rest of the bromide solution in a continuous way. After the addition was complete, the reaction was heated to reflux for an additional 90 min and was allowed to reach room temperature. The stock solution was kept in the refrigerator under Argon and it was stable for more than a month to be used for Grignard reactions. *Note* that the solution was not titrated and for the subsequent reactions was treated as a 1 M solution of phenylmagnesium bromide based on the amounts used and assuming full conversion of the reagents.

General procedure for the synthesis of alcohols 3a, 3b, 3d, 3f, 3h-3i* and 14j, 14k (A)

To a solution of the corresponding aldehyde (1 eq.) in anhydrous THF (0.2 M) at -78°C was added dropwise a solution of the appropriate Grignard reagent (1.3 eq, 1 M) in THF prepared as described above. The reaction was allowed to stir at -78°C until completion by TLC (usually complete within 30 min). The reaction was quenched at -78° C by the dropwise addition of aq. NH_4Cl (sat.) and the mixture was allowed to reach room temperature before being transferred to a separatory funnel with ether. The organic layer was washed with water (2x) and brine (1x), dried over Na_2SO_4, filtered and concentrated to dryness. The crude alcohol was purified by flash column chromatography (FCC) using the appropriate system consisting of hexanes/AcOEt, when necessary.

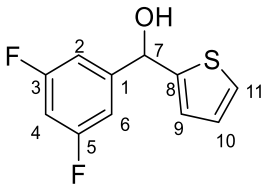

(3,5-difluorophenyl)(thiophen-2-yl)methanol (3a) was synthesized from thiophene-2-carbaldehyde (0.5 g, 4.46 mmol) and (3,5-difluorophenyl)magnesium bromide (1M) according to the general procedure A. Light yellow oil (0.94 g, 94 %). R_f = 0.5 (hexanes/AcOEt 8:2); ^{1}H NMR (250 MHz, $CDCl_3$) δ: 7.30 (dd, $^3J_{H\text{-}H}$ = 4.9 Hz, $^4J_{H\text{-}H}$ = 0.7 Hz, 1H, thiopheneH11), 7.02 – 6.92 (m, 4H, ArH2/H6, thiopheneH10/H11), 6.73 (tt, $^3J_{H\text{-}F}$ = 8.9 Hz, $^4J_{H\text{-}H}$ = 2.3 Hz, 1H, ArH4), 6.02 (s, 1H, H7), 2.54 (brs, 1H, -O*H*); ^{13}C NMR (63 MHz, $CDCl_3$)

δ: 163.18 (dd, $^{1}J_{C\text{-}F}$ = 249.3 Hz, $^{3}J_{C\text{-}F}$ = 12.6 Hz, ArC3/C5), 147.07 (t, $^{3}J_{C\text{-}F}$ = 8.5 Hz, ArC1), 146.77 (thiopheneC8), 126.98 (thiopheneC10), 126.26 (thiopheneC9), 125.56 (thiopheneC11), 109.28 (m, AXX'-system, ArC2/C6), 103.34 (t, $^{2}J_{C\text{-}F}$ = 25.5 Hz, ArC4), 71.35 (C7); Elemental analysis calcd. (%) for $C_{11}H_8F_2OS$: C, 58.40; H, 3.56; found: C, 58.62; H, 3.43.

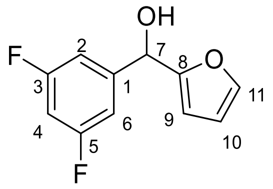

(3,5-difluorophenyl)(furan-2-yl)methanol (3b) was synthesized from furfural (0.5 g, 5.2 mmol) and (3,5-difluorophenyl)magnesium bromide (1M) according to the general procedure A. Purified by FCC using hexanes/AcOEt 9:1; Light yellow oil (1.05 g, 96%); R_f = 0.4 (hexanes/AcOEt 8:2); ^{1}H NMR (500 MHz, $CDCl_3$) δ: 7.43 – 7.38 (m, 1H, furaneH11), 7.01 – 6.94 (m, 2H, ArH2/H6), 6.75 (tt, $^{3}J_{H\text{-}F}$ = 8.9 Hz, $^{4}J_{H\text{-}H}$ = 2 Hz, ArH4), 6.37 – 6.31 (m, 1H, furaneH10), 6.19 – 6.16 (m 1H, furaneH9), 5.80 (s, 1H, H7), 2.47 (brs, 1H, -O*H*); ^{13}C (63 MHz, $CDCl_3$) δ: 163.13 (dd, $^{1}J_{C\text{-}F}$ = 249.1 Hz, $^{3}J_{C\text{-}F}$ = 12.6 Hz, ArC3/C5), 157.41 (furanC8), 144.79 (t, $^{3}J_{C\text{-}F}$ = 8.7 Hz, ArC1), 143.08 (furaneC11), 110.52 (furaneC10), 109.57 (m, AXX'-system, ArC2/C6), 108.03 (furaneC9), 103.4 (t, $^{2}J_{C\text{-}F}$ = 25.5 Hz, ArC4), 69.10 (C7); Elemental analysis calcd. (%) for $C_{11}H_8F_2O_2$: C, 62.86; H, 3.84; found: C, 62.63; H, 4.02.

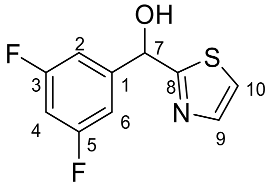

(3,5-difluorophenyl)(thiazol-2-yl)methanol (3d) was synthesized from thiazole-2-carbaldehyde (0.5 g, 4.42 mmol) and (3,5-difluorophenyl)magnesium bromide (1M) according to the general procedure A. Purified by FCC using hexanes/AcOEt 8:2; White solid (0.95 g, 95%); R_f = 0.1 (hexanes/AcOEt 8:2); ^{1}H NMR (500 MHz, $CDCl_3$) δ: 7.59 (d, *J* = 3.2 Hz, 1H, thiazoleH9), 7.29 (d, *J* = 3.2 Hz, 1H, thiazoleH10), 7.02 – 6.95 (m, 2H, ArH2/H6), 6.72 (tt, $^{3}J_{H\text{-}F}$ = 8.8 Hz, $^{4}J_{H\text{-}H}$ = 2.2 Hz, 1H, ArH4), 6.01 (s, 1H, H7), 5.26 (brs, 1H, -O*H*); ^{13}C NMR (63 MHz, $CDCl_3$) δ: 173.70 (thiazoleC8), 163.28 (dd, $^{1}J_{C\text{-}F}$ = 249.9 Hz, $^{3}J_{C\text{-}F}$ = 12.6 Hz, ArC3/C5), 144.91 (t, $^{3}J_{C\text{-}F}$ = 8.7 Hz, ArC1), 141.63 (thiazoleC9), 120.50 (thiazoleC10), 109.64 (m, AXX'-system, ArC2/C6), 104.05 (t, $^{2}J_{C\text{-}F}$ = 25.3 Hz, ArC4), 72.43 (t, $^{4}J_{C\text{-}F}$ = 2.2 Hz, C7); Elemental analysis calcd. (%) for $C_{10}H_7F_2NOS$: C, 52.86; H, 3.11; N, 6.16; found: C, 52.58; H, 3.29; N, 5.82.

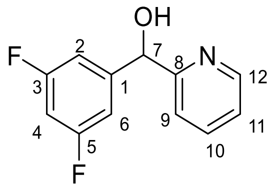

(3,5-difluorophenyl)(pyridin-2-yl)methanol (3f) was synthesized from pyridine-2-carbaldehyde (0.5 g, 4.67 mmol) and (3,5-difluorophenyl)magnesium bromide (1M) according to the general procedure A. Purified by FCC using hexanes/AcOEt 8:2; Light yellow oil (0.67 g, 65%); R_f = 0.35 (hexanes/AcOEt 7:3); ^{1}H NMR (500 MHz, $CDCl_3$) δ: 8.45 (d, $^{3}J_{H\text{-}H}$ = 4.4 Hz, 1H, pyridineH12), 7.62 (dt, $^{3}J_{H\text{-}H}$ = 7.7 Hz, $^{4}J_{H\text{-}H}$ = 1.7

Hz, 1H, pyridineH10), 7.23 (d, $^3J_{H\text{-}H}$ = 8.1 Hz, 1 H, pyridineH9), 7.19 – 7.14 (m, 1H, pyridineH11), 6.97 – 6.87 (m, 2H, ArH2/H6), 6.65 (tt, $^3J_{H\text{-}F}$ = 9 Hz, $^4J_{H\text{-}H}$ = 2.1 Hz, 1H, ArH4), 5.72 (s, 1H, H*7*); ^{13}C NMR (126 MHz, $CDCl_3$) *δ*: 163.06 (dd, $^1J_{C\text{-}F}$ = 249.7 Hz, $^3J_{C\text{-}F}$ = 12.6 Hz, ArC3/C5), 160.28 (pyridineC8), 148.17 (pyridineC12), 147.33 ($^3J_{C\text{-}F}$ = 8.3 Hz, ArC1), 137.29 (pyridineC10), 122.94 (pyridineC11), 121.2 (pyridineC9), 109.65 (m, AXX'-system, ArC2/C6), 102.97 (t, $^2J_{C\text{-}F}$ = 25.2 Hz, ArC4), 74.36 (C7); Elemental analysis calcd. (%) for $C_{12}H_9F_2NO$: C, 65.16; H, 4.10; N, 6.33; found: C, 65.07; H, 4.11; N, 6.25.

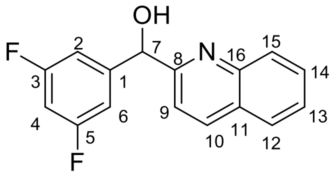

(3,5-difluorophenyl)(quinolin-2-yl)methanol (3h) was synthesized from quinoline-2-carbaldehyde (0.51 g, 3.24 mmol) and (3,5-difluorophenyl)magnesium bromide (1M) according to the general procedure A. Purified by FCC using hexanes/AcOEt 9:1 to 8:2; Yellow solid (0.77 g, 87%); R_f = 0.5 (hexanes/AcOEt 7:3); ^{1}H NMR (500 MHz, $CDCl_3$) *δ*: 8.18 – 8.08 (m, 2H, quinolineH), 7.83 (d, *J* = 8.1 Hz, 1H, quinolineH), 7.80 – 7.74 (m, 1H, quinolineH), 7.61 – 7.54 (m, 1H, quinolineH), 7.22 (d, *J* = 8.4 Hz, 1H, quinolineH), 7.02 – 6.94 (m, 2H, ArH2/H6), 6.75 – 6.67 (m, 1H, ArH4), 6.10 (brs, 1H, -O*H*), 5.84 (s, 1H, H7); ^{13}C NMR (63 MHz, $CDCl_3$) *δ*: 163.28 (dd, $^1J_{C\text{-}F}$ = 249.2, $^3J_{C\text{-}F}$ = 12.3 Hz, ArC3/C5), 159.32, (quinolineC8), 146.62 (t, $^3J_{C\text{-}F}$ = 8.2 Hz, ArC1), 145.43(quinolineC16), 138.40 (quinolineC10), 130.77 (quinolineC), 129.08 (quinolineC11),128.33 (quinolineC), 127.87 (quinolineC), 127.83 (quinolineC), 127.40 (quinolineC), 119.06 (quinC9), 110.25 (dd, $^2J_{C\text{-}F}$ = 15.4 Hz, $^4J_{C\text{-}F}$ = 8.6 Hz, ArC2/C6), 103.96, (t, $^2J_{C\text{-}F}$ = 25.4 Hz, ArC1), 74.04 (C7); Elemental analysis calcd. (%) for $C_{16}H_{11}F_2NO$: C, 70.84; H, 4.09; N, 5.16; found: C, 70.68; H, 4.22; N, 4.97.

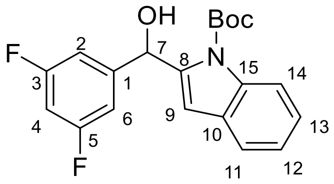

***tert*-butyl 2-((3,5-difluorophenyl)(hydroxy)methyl)-1*H*-indole-1-carboxylate (3i*)** was synthesized from tert-butyl 2-formyl-1*H*-indole-1-carboxylate[3] (1.9 g, 7.7 mmol) and (3,5-difluorophenyl)magnesium bromide (1M) according to the general procedure A. Purified by FCC using hexanes/AcOEt 9:1; white solid (2.3 g, 83%); R_f = 0.4 (hexanes/AcOEt 8:2); ^{1}H NMR (250 MHz, $CDCl_3$) *δ*: 7. 97 (d, *J* = 8.3 Hz, 1H, indoleH), 7.50 (d, *J* = 7.6 Hz, 1H, indoleH), 7.38 – 7.20 (m, 2H, indoleH), 7.07 – 6.92 (m, 2H, ArH2/H6), 6.76 (tt, $^3J_{H\text{-}F}$ = 8.9 Hz, $^4J_{H\text{-}H}$ = 2.3 Hz, 1H, ArH4), 6.33 (s, 1H, indoleH9), 6.12 (s, 1H, H7), 4.94 (brs, 1H, -O*H*), 1.68 (s, 9H, -N(CO)OCC*H*$_3$); ^{13}C NMR (63 MHz, $CDCl_3$) *δ*: 163.02 (dd, $^1J_{C\text{-}F}$ = 247.6 Hz, $^3J_{C\text{-}F}$ = 12.6 Hz, ArC3/C5), 151.82 (-N(*C*O)OCCH$_3$), 145.90 (t, $^3J_{C\text{-}F}$ = 8.5 Hz, ArC1), 141.48 (indoleC), 136.53 (indoleC), 128.66 (indoleC), 125.12 (indoleC), 123.48 (indoleC), 121.37 (indoleC), 115.83 (indoleC), 111.62 (indoleC), 109.81 (m, AXX'-system, ArC2/C6), 102.86 (t, $^2J_{C\text{-}F}$ = 25.0Hz, ArC4), 85.91 (C7), 69.44 (-N(CO)O*C*CH$_3$), 28.26 (-N(*C*O)OC*C*H$_3$); Elemental analysis calcd. (%) for $C_{20}H_{19}F_2NO_3$: C, 66.84; H, 5.33; N, 3.90; found: C, 67.06; H, 5.57; N, 4.08.

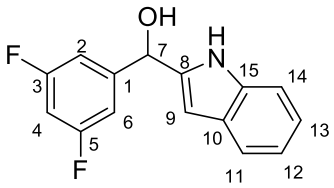

(3,5-difluorophenyl)(1H-indol-2-yl)methanol (3i) was synthesized from indole-2-carbaldehyde (0.5 g, 5.2 mmol) and (3,5-difluorophenyl)magnesium bromide (1M) according to the general procedure A (*note that 2.6 equiv of the Grignard reagent was used in this case to make up for the deprotonation of the indoleNH*). Purified by FCC using hexanes/AcOEt 8:2; olive green oil (0.506 g, 57%); R_f = 0.5 (hexanes/AcOEt 7:3); ^{1}H NMR (500 MHz, $CDCl_3$) δ: 8.20 (s, 1H, indoleN*H*), 7.60 (d, *J* = 7.9 Hz, 1H, indoleH), 7.28 (d, *J* = 8 Hz, 1H, indoleH), 7.25 – 7.19 (m, 1 H, indoleH), 7.18 – 7.12 (m, 1H, indoleH), 6.98 – 6.91 (m, 2H, ArH2/H6), 6.78 (tt, $^3J_{H\text{-}F}$ = 8.8 Hz, $^4J_{H\text{-}H}$ = 2.3 Hz, 1H, ArH4), 6.32 (s, 1H, indoleH9), 5.83 (s, 1H, H7), 2.99 (s, 1H, -O*H*); ^{13}C NMR (126 MHz, $CDCl_3$) δ: 163.18 (dd, $^1J_{C\text{-}F}$ = 249.6 Hz, $^3J_{C\text{-}F}$ = 12.7 Hz, ArC3/C5), 145.56 (t, $^3J_{C\text{-}F}$ = 8.4 Hz, ArC1), 138.76 (indoleC), 136.40 (indoleC), 127.91 (indoleC), 122.73 (indoleC), 120.94 (indoleC), 120.32 (indoleC), 111.27 (indoleC), 109.41 (m, AXX'-system, ArC2/C6), 103.45 (t, $^2J_{C\text{-}F}$ = 25.4 Hz, ArC4), 101.52 (indoleC9), 69.76 (C7); Elemental analysis calcd. (%) for $C_{15}H_{11}F_2NO$: C, 69.49; H, 4.28; N, 5.40; found: C, 69.54; H, 4.36; N, 5.47.

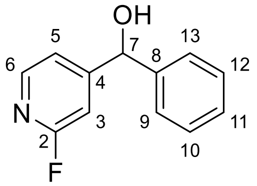

(2-fluoropyridin-4-yl)(phenyl)methanol (14j) was synthesized from 2-fluoroisonicotinaldehyde (710 mg, 5.7 mmol) and phenylmagnesium bromide 1M according to the general procedure A. Off-white viscous oil (1.03 g, 89 %). R_f = 0.3 (hexanes/AcOEt 8:2); ^{1}H NMR (500 MHz, $CDCl_3$) δ: 8.12 (d, $^3J_{H\text{-}H}$ = 5.3 Hz, 1H, ArH6), 7.41 – 7.31 (m, 5H, PhH), 7.16 (d, $^3J_{H\text{-}F}$ = 5.2, 1H, ArH3), 7.03 (s, 1H, ArH5), 5.81 (s, 1H, H*7*), 2.60 (brs, 1H, -O*H*); ^{13}C NMR (63 MHz, $CDCl_3$) δ: 164.04 (d, $^1J_{C\text{-}F}$ = 239.7 Hz, ArC2), 159.22 (d, $^3J_{C\text{-}F}$ = 7.6 Hz, Ar*C*4), 147.21 (d, $^3J_{C\text{-}F}$ = 14.4 Hz, ArC6), 142.27 (PhC8), 128.99 (PhC10/12), 128.54 (PhC11), 126.91 (PhC9/13), 119.26 (d, $^4J_{C\text{-}F}$ = 3.9 Hz, ArC5), 106.97 (d, $^2J_{C\text{-}F}$ = 37.6 Hz, ArC3), 74.59 (d, $^4J_{C\text{-}F}$ = 2.9 Hz, C7); Elemental analysis calcd. (%) for $C_{12}H_{10}FNO$: C, 70.93; H, 4.96; N, 6.89; found: C, 70.68; H, 5.12; N, 6.71.

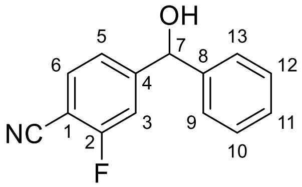

2-fluoro-4-(hydroxy(phenyl)methyl)benzonitrile (14k) was synthesized from 2-fluoro-4-formylbenzonitrile (1 g, 6.7 mmol) and phenylmagnesium bromide 1M according to the general procedure A; off-white solid (1.47 g, 96 %). R_f = 0.25 (hexanes/AcOEt 8:2); ^{1}H NMR (500 MHz, DMSO-d_6) δ: 7.85 (t, $^3J_{H\text{-}H}$ = 7.5 Hz, 1H, ArH6), 7.53 (d, $^3J_{H\text{-}F}$ = 12.0 Hz, 1H, ArH3), 7.43 – 7.38 (m, 3H, ArH5, PhH), 7.32 (t, *J* = 7.7 Hz, 2H, PhH), 7.24 (t, *J* = 7.2 Hz, 1H, PhH), 6.28 (d, $^2J_{H\text{-}OH}$ = 3.9 Hz, 1H, H7), 5.81 (d, *J* = 4.2 Hz, 1H, -O*H*); ^{13}C NMR (63 MHz, $CDCl_3$) δ: 163.30 (d, $^1J_{C\text{-}F}$ = 259.1 Hz, ArC2), 152.60 (d, $^3J_{C\text{-}F}$ = 7.2 Hz, ArC4), 142.35(PhC8), 133.38 (ArC6), 129.01 (PhC10/12), 128.54 (PhC11), 126.78 (PhC9/13), 122.76 (d, $^4J_{C\text{-}F}$ = 3.4 Hz, Ar*C6*), 114.22 (d, $^2J_{C\text{-}F}$ = 20.6 Hz, ArC3), 114.1 (*C*≡N), 99.79 (d, $^2J_{C\text{-}F}$ = 15.9 Hz, ArC1), 75.12 (d,

$^4J_{C-F}$ = 1.9 Hz, C*7*); Elemental analysis calcd. (%) for $C_{14}H_{10}FNO$: C, 74.00; H, 4.44; N, 6.16; found: C, 73.81; H, 4.62; N, 5.97.

General procedure for the synthesis of phthalimides 4a, 4d, 4f, 4h and 15j, 15k (B)

A solution of DIAD (1.4 eq) in THF (1 M) was added dropwise to a mixture of the corresponding alcohol (1 eq), N-hydroxyphthalimide (1.4 eq) and triphenylphoshine (1.4 eq) in THF (0.25 M) at -10 °C. During the addition, the color of the solution turned abruptly to deep orange/red. After the addition was complete, the reaction was stirred overnight allowing it gradually to reach room temperature resulting to an almost colorless solution. The reaction was then diluted with ethyl acetate and washed with sat. $NaHCO_3$ solution (x2), water (x1) and brine (x1). The organic layer was dried over sodium sulfate, filtered and evaporated to dryness. The crude phthalimide was purified by FCC using an appropriate system of hexanes/AcOEt.

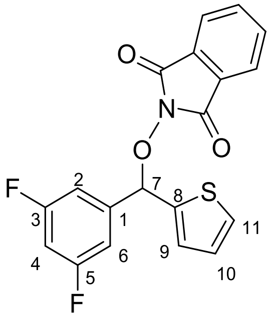

2-((3,5-difluorophenyl)(thiophen-2-yl)methoxy)isoindoline-1,3-dione (4a) was synthesized from **3a** (0.331 g, 1.46 mmol) according to the general procedure B. Purified by FCC using hexanes/AcOEt 9:1; Light yellow solid (0.48 g, 88%); R_f = 0.5 (hexanes/AcOEt 7:3); ^{1}H NMR (250 MHz, $CDCl_3$) *δ*: 7.84 – 7.68 (m, 4H, N*Phth*), 7.40 (dd, $^3J_{H-H}$ = 5.05 Hz, $^4J_{H-H}$ = 1.06 Hz, 1H, thiopheneH11), 7.24 – 7.18 (m, 2H, ArH2/H6), 7.12 – 7.08 (m, 1H, thiopheneH9), 6.97 (dd, $^3J_{H-H}$ = 5.05 Hz, $^3J_{H-H}$ = 3.7 Hz, 1H, thiopheneH10), 6.81 (tt, $^3J_{H-F}$ = 8.6 Hz, $^4J_{H-H}$ = 2.4 Hz, 1H, ArH4), 6.61 (s, 1H, H7); Elemental analysis calcd. (%) for $C_{19}H_{11}F_2NO_3S$: C, 61.45; H, 2.99; N, 3.77 found: C, 61.31; H, 3.04; N, 4.01

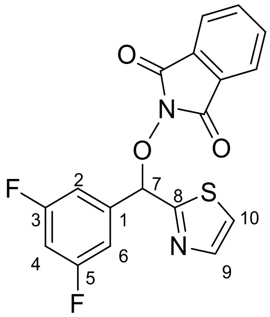

2-((3,5-difluorophenyl)(thiazol-2-yl)methoxy)isoindoline-1,3-dione (4d) was synthesized from **3d** (0.2 g, 0.88 mmol) according to the general procedure B. Purified by FCC using hexanes/AcOEt 8:2 and then recrystallized from EtOH to afford a white solid (0.22 g, 67%); R_f = 0.5 (hexanes/AcOEt 7:3 ran x2); ^{1}H NMR (500 MHz, $CDCl_3$) *δ*: 7.81 – 7.77 (m, 2H, N*Phth*), 7.76 (d, $^3J_{H-H}$ = 3.2 Hz, 1H, thiazoleH9), 7.75 – 7.71 (m, 2H, N*Phth*), 7.49 (d, $^3J_{H-H}$ = 3.2 Hz, 1H, thiazoleH10), 7.24 – 7.18 (m, 2H, ArH2/H6), 6.83 (tt, $^3J_{H-F}$ = 8.8 Hz, $^4J_{H-H}$ = 2.1 Hz, 1H ArH4), 6.62 (s, 1H, H7); Elemental analysis calcd. (%) for $C_{18}H_{10}F_2N_2O_3S$: C, 58.06; H, 2.71; N, 10.20 found: C, 57.81; H, 3.01; N, 9.87

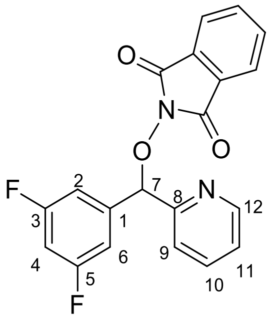

2-((3,5-difluorophenyl)(pyridin-2-yl)methoxy)isoindoline-1,3-dione (4f) was synthesized from **3f** (0.17 g, 0.77 mmol) according to the general procedure B. Purified by FCC using hexanes/AcOEt 8:2 to 7:3 and then recrystallized from EtOH to afford a light yellow solid (0.2 g, 71%); R_f = 0.3 (hexanes/AcOEt 7:3); ^{1}H NMR (500 MHz, $CDCl_3$) δ: 8.54 (d, $^3J_{H-H}$ = 4.7 Hz, 1H, pyridineH12), 7.86 (d, $^3J_{H-H}$ = 7.9 Hz, 1H, pyridineH9), 7.81 (dd, $^3J_{H-H}$ = 7.9 Hz, $^4J_{H-H}$ = 1.7 Hz, 1H, pyridineH10), 7.79 – 7.75 (m, 2H, N*Phth*), 7.73 – 7.69 (m, 2H, N*Phth*), 7.29 – 7.25 (m, 1H, pyridineH11), 7.15 – 7.09 (m, 2H, ArH2/H6), 6.75 (tt, $^3J_{H-F}$ = 8.8 Hz, $^4J_{H-H}$ = 2.3 Hz, 1H, ArH4), 6.46 (s, 1H, H7); Elemental analysis calcd. (%) for $C_{20}H_{12}F_2N_2O_3$: C, 65.58; H, 3.30; N, 10.37; found C, 65.82; H, 3.41; N, 10.51.

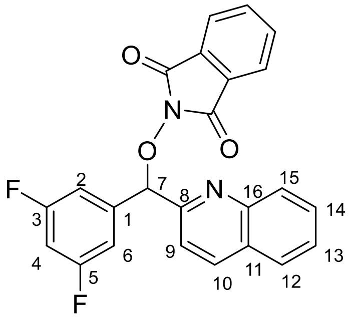

2-((3,5-difluorophenyl)(quinolin-2-yl)methoxy)isoindoline-1,3-dione (4h) was synthesized from **3h** (0.05 g, 0.184 mmol) according to the general procedure B. Purified by FCC using hexanes/AcOEt 8:2 and then recrystallized from EtOH to afford a light yellow solid (58 mg, 76%); R_f = 0.4 (hexanes/AcOEt 8:2 ran two times); ^{1}H NMR (500 MHz, $CDCl_3$) δ: 8.27 (d, *J* = 8.6 Hz, 1H, quinolineH), 8.01 (d, *J* = 8.4 Hz, 1H, quinolineH), 7.96 (d, *J* = 8.6 Hz, 1H, quinolineH), 7.82 (d, *J* = 8 Hz, 1H, quinolineH) 7.76 – 7.63 (m, 5H, N*Phth* overlapping with quinolineH), 7.58 – 7.50 (m, 1H, quinolineH), 7.20 – 7.11 (m, 2H, ArH2/H6), 6.75 (tt, $^3J_{H-F}$ = 8.8 Hz, $^4J_{H-H}$ = 2.3 Hz, 1H, H4), 6.61 (s, 1H, H7); Elemental analysis calcd. (%) for $C_{24}H_{14}F_2N_2O_3$: C, 69.23; H, 3.39; N, 6.73; found: C, 69.11; H, 3.43; N, 6.59.

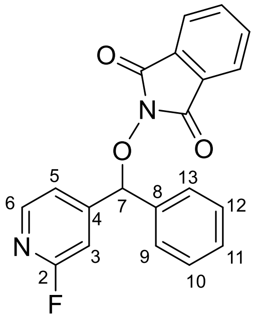

2-((2-fluoropyridin-4-yl)(phenyl)methoxy)isoindoline-1,3-dione (15j) was synthesized from **14j** (0.48 g, 2.36 mmol) according to the general procedure B. Purified by FCC using hexanes gradually to hexanes/AcOEt 9:1 and then recrystallized from EtOH to afford a white solid (0.72 g, 86%); R_f = 0.4 (hexanes/AcOEt 7:3); ^{1}H NMR (250 MHz, $CDCl_3$) *δ*: 8.22 (d, $^3J_{H\text{-}H}$ = 5.3 Hz, 1H, ArH6), 7.79-7.69 (m, 4H, *Phth*), 7.51 – 7.43 (m, 2H, ArH3, PhH), 7.37 (t, *J* = 3.3 Hz, 4H, PhH), 7.16 (s, 1H, ArH5), 6.47 (s, 1H, H*7*); Elemental analysis calcd. (%) for $C_{20}H_{13}FN_2O_3$: C, 68.96; H, 3.76; N, 8.04; found: C, 69.13; H, 3.91; N, 8.21.

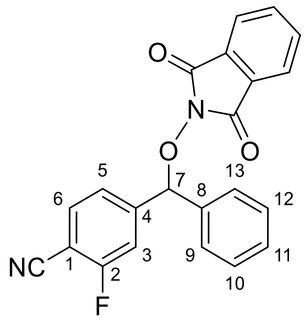

4-(((1,3-dioxoisoindolin-2-yl)oxy)(phenyl)methyl)-2-fluorobenzonitrile (15k) was synthesized from **14k** (0.7 g, 3.1 mmol) according to the general procedure B. Purified by FCC using hexanes/AcOEt 8:2 to 7:3 and then recrystallized from EtOH to afford a white solid (0.98 g, 85%); R_f = 0.4 (hexanes/AcOEt 7:3); ^{1}H NMR (250 MHz, $CDCl_3$) *δ*: 7.79-7.70 (m, 4H, H*Phth*), 7.63 (dd, $^3J_{H\text{-}H}$ = 8.0, $^4J_{H\text{-}F}$ = 6.4 Hz, 1H, ArH6), 7.54 – 7.42 (m, 4H, PhH), 7.37 (m, 3H, ArH3/H5, Ph*H*), 6.48 (s, 1H, H*7*); Elemental analysis calcd. (%) for $C_{22}H_{13}FN2O_3$: C, 70.96; H, 3.52; N, 7.52; found: C, 71.19; H, 3.73; N, 7.79.

General procedure for the synthesis of hydroxylamines 5a, 5d, 5f, 5h and 16j, 16k (C)

Hydrazine monohydrate 55% aq. solution (2 eq) were added dropwise in a solution (0.1 M) of the corresponding phthalimide (1 eq.) in EtOH/DCM (1:1) at room temperature. After stirring for 60 – 120 min, ether was added and the reaction was left in the freezer for 30 min. The white precipitate was filtered off, washed with cold EtOH and the filtrate was evaporated to dryness. The crude hydroxylamine was purified by FCC using an appropriate system of hexanes/AcOEt with 1% Et_3N.

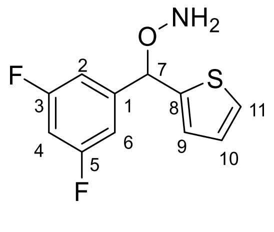

***O*-((3,5-difluorophenyl)(thiophen-2-yl)methyl)hydroxylamine (5a)** was synthesized from **4a** (0.05 g, 0.134 mmol) according to the general procedure C. Purified by FCC using hexanes/AcOEt/Et_3N 9:1:0.1; colorless oil (0.024 g, 74%); R_f = 0.5 (hexanes/AcOEt 7:3); ^{1}H NMR (250 MHz, $CDCl_3$) *δ*: 7.32 (dd, $^3J_{H\text{-}H}$ = 5.1 Hz, $^4J_{H\text{-}H}$ = 1.3 Hz, 1H, thiopheneH11), 7.00 – 6.93 (m, 3H, ArH2/H6 overlapping with thiopheneH9), 6.91 – 6.86 (m, 1H, thiopheneH10), 6.76 (tt, $^3J_{H\text{-}F}$ = 8.8 Hz, $^4J_{H\text{-}H}$ = 2.4 Hz, 1H, ArH4), 5.81 (s, 1H, H7), 5.58 (brs, 2H, -O*NH$_2$*); ^{13}C NMR (63 MHz, $CDCl_3$) *δ*: 163.20 (dd, $^1J_{C\text{-}F}$ = 250 Hz, $^3J_{C\text{-}F}$ = 12.5 Hz, ArC3/C5), 144.93 (t, $^3J_{C\text{-}F}$ = 8.4 Hz, ArC1), 143.02 (thiopheneC8), 126.80 (thiopheneC10), 126.67 (thiopheneC9), 126.50 (thiopheneC11), 109.95 (m, AXX'-system, ArC2/C6), 103.54 (t, $^2J_{C\text{-}F}$ = 25.4 Hz, ArC4), 83.26 (t, $^4J_{C\text{-}F}$ = 2 Hz, C7); Elemental analysis calcd. (%) for $C_{11}H_9F_2NOS$: C, 54.76; H, 3.76; N, 5.81; found: C, 54.39; H, 3.85; N, 6.04.

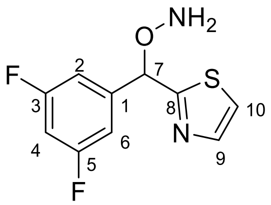

***O*-((3,5-difluorophenyl)(thiazol-2-yl)methyl)hydroxylamine (5d)** was synthesized from **4d** (0.1 g, 0.27 mmol) according to the general procedure C. Purified by FCC using hexanes/AcOEt/Et_3N 8:2:0.1; Light yellow oil (0.059 g, 91%); R_f = 0.3 (hexanes/AcOEt 7:3); ^1H NMR (500 MHz, $CDCl_3$) δ: 7.77 (d, $^3J_{H\text{-}H}$ = 3.2 Hz, 1H, thiazoleH9), 7.36 (d, $^3J_{H\text{-}H}$ = 3.3 Hz, 1H, thiazoelH10), 6.98 (d, $^3J_{H\text{-}F}$ = 5.9 Hz, 2H, ArH2/H6), 6.76 (tt, $^3J_{H\text{-}F}$ = 9.0, $^4J_{H\text{-}H}$ = 2.4 Hz, 1H, ArH4), 5.93 (s, 1H, H*7*), 5.74 (brs, 2H, -O*NH₂*); ^{13}C NMR (126 MHz, $CDCl_3$) δ: 169.73 (thiazoleC8), 163.22 (dd, $^1J_{C\text{-}F}$ = 249.7, $^3J_{C\text{-}F}$ = 12.6 Hz, ArC3/C5), 142.99 (thiazoleC9), 142.86 (t, $^3J_{C\text{-}F}$ = 8.4 Hz, ArC1), 120.01 (thiazoleC10), 110.29, (m, AXX'-system, ArC2/C6), 104.03 (t, $^2J_{C\text{-}F}$ = 25.3 Hz, ArC4), 84.90 (C7); Elemental analysis calcd. (%) for $C_{10}H_8F_2N_2OS$: C, 49.58; H, 3.33; N, 11.56; found: C, 49.47; H, 3.48; N, 11.29.

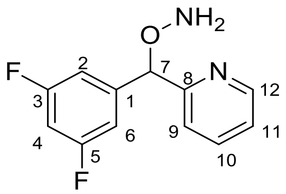

***O*-((3,5-difluorophenyl)(pyridin-2-yl)methyl)hydroxylamine (5f)** was synthesized from **4f** (0.05 g, 0.12 mmol) according to the general procedure C. Purified by FCC using hexanes/AcOEt/Et_3N 8:2:0.1; Light yellow oil (0.029 g, 83%); R_f = 0.2 (hexanes/AcOEt 7:3); ^1H NMR (250 MHz, $CDCl_3$) δ: 8.57 (d, $^3J_{H\text{-}H}$ = 5.0 Hz, 1H, pyridineH12), 7.72 – 7.65 (m, 1H, pyridineH), 7.36 (d, *J* = 7.8 Hz, 1H, pyridineH), 7.22 – 7.17 (m, 1H, pyridineH), 6.98 – 6.90 (m, 2H, ArH2/H6), 6.68 (tt, $^3J_{H\text{-}F}$ = 8.9 Hz, $^4J_{H\text{-}H}$ = 2.3 Hz, ArH4), 5.70 (brs, 3H, -ONH_2 overlapping with H7); ^{13}C NMR (63 MHz, $CDCl_3$) δ: 163.04 (dd, $^1J_{C\text{-}F}$ = 249.6 Hz, $^3J_{C\text{-}F}$ = 12.5 Hz, ArC3/C5), 159.50 (pyridineC8), 149.57 (pyridineC12), 144.09 (t, $^3J_{C\text{-}F}$ = 8.9 Hz, ArC1), 137.04 (pyridineC10), 123.05 (pyridineC11), 121.10 (pyridineC9), 110.13 (m, AXX'-system, ArC2/C6), 103.32 (t, $^2J_{C\text{-}F}$ = 25.4 Hz, ArC4), 88.11 (t, $^4J_{C\text{-}F}$ = 2 Hz, ArC7); Elemental analysis calcd. (%) for $C_{12}H_{10}F_2N_2O$: C, 61.02; H, 4.27; N, 11.86; found: C, 61.09; H, 4.33; N, 11.79.

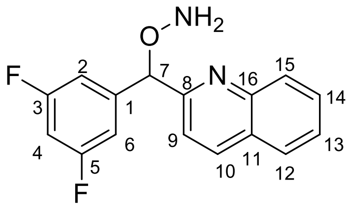

***O*-((3,5-difluorophenyl)(quinolin-2-yl)methyl)hydroxylamine (5h)** was synthesized from **4h** (0.05 g, 0.12 mmol) according to the general procedure C. Purified by FCC using hexanes/AcOEt/Et_3N 8:2:0.1; Light yellow oil (0.029 g, 83%); R_f = 0.2 (hexanes/AcOEt 7:3); ^1H NMR (500 MHz, $CDCl_3$) δ: 8.18 – 8.12 (m, 2H, quinolineH), 7.80 (d, *J* = 8.2 Hz, 1H, quinolineH), 7.72 (t, *J* = 7.7 Hz, 1H, quinolineH), 7.54 (t, *J* = 7.7 Hz, 1H, quinolineH), 7.47 (d, *J* = 8.2 Hz, 1H, quinolineH), 7.06 – 6.99 (m, 2H, ArH2/H6), 6.69 (tt, $^3J_{H\text{-}F}$ = 8.8 Hz, $^4J_{H\text{-}H}$ = 2.2 Hz, ArH4), 5.91 (s, H7), 5.73 (brs, 2H, -ONH_2); ^{13}C NMR (126 MHz, $CDCl_3$) δ: 163.11 (dd, $^1J_{C\text{-}F}$ = 248.2 Hz, $^3J_{C\text{-}F}$ = 12.4 Hz, ArC3/C5), 159.91 (quinolineC8), 147.75 (quinolineC16), 143.79 (t, $^3J_{C\text{-}F}$ = 8.7 Hz, ArC1), 137.33 (quinolineC), 129.91 (quinolineC), 129.57 (quinolineC), 127.77 (quinolineC11), 127.71

(quinolineC), 126.89 (quinolineC), 118.68 (quinolineC), 110.07 (m, AXX'-system, ArC2/C6), 103.35 (t, $^{2}J_{C-F}$ = 25.6 Hz, ArC4), 88.68 (C7); Elemental analysis calcd. (%) for $C_{16}H_{12}F_2N_2O$: C, 67.13; H, 4.23; N, 9.79; found: 67.32; H, 4.47; N, 9.94.

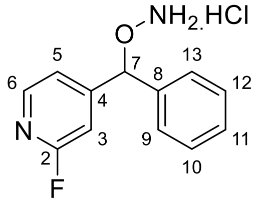

***O*-((2-fluoropyridin-4-yl)(phenyl)methyl)hydroxylamine HCl salt (16j)** was synthesized from **15j** (98 mg, 0.28 mmol) according to the general procedure C. Purified by FCC using hexanes/AcOEt/Et_3N 8:2:0.1. The free base was dissolved in dry Et_2O (50 mL) and the resulting solution was cooled to –30 °C. A solution of hydrogen chloride in Et_2O (1.0 M) was added dropwise under stirring until the pH of the mixture reached approximately 2. A precipitate formed upon acidification, and the reaction flask was stored in a freezer for 30 minutes to ensure complete precipitation. The resulting solid was collected by vacuum filtration, washed thoroughly with cold dry Et_2O (3 × 10 mL), and dried under high vacuum for 3 h to afford the hydrochloride salt as an off-white solid (56 mg, 79%); R_f = 0.3 (free -base hexanes/AcOEt 7:3); ^{1}H NMR (500 MHz, D_2O) δ: 8.21 (d, $^{3}J_{H-H}$ = 5.4 Hz, 1H, ArH6), 7.51 (q, J = 2.3 Hz, 5H, PhH), 7.38 (d, $^{3}J_{H-F}$ = 5.4 Hz, 1H, ArH3), 7.20 (s, 1H, ArH5), 6.23 (brs, 1H, H7); ^{13}C NMR (63 MHz, D_2O) δ: 163.68 (d, $^{1}J_{C-F}$ = 239.9 Hz, ArC2), 153.62 (d, $^{3}J_{C-F}$ = 7.6 Hz, ArC6), 147.50 (d, $^{3}J_{C-F}$ = 13.1 Hz, ArC4), 135.38 (PhC8), 130.24 (PhC11), 129.44 (PhC10/12), 128.07 (PhC9/13), 119.66 (d, $^{4}J_{C-F}$ = 3.9 Hz, ArC6), 107.58 (d, $^{2}J_{C-F}$ = 37.5 Hz, ArC4), 85.56 (d, $^{4}J_{C-F}$ = 3.0 Hz, C7); Elemental analysis calcd. (%) for $C_{12}H_{12}ClFN_2O$: C, 56.59; H, 4.75; N, 11.00; found: C, 56.31; H, 5.01; N, 10.73.

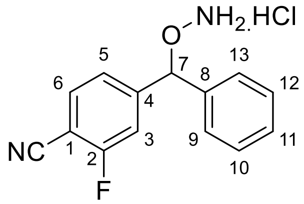

4-((aminooxy)(phenyl)methyl)-2-fluorobenzonitrile HCl salt (16k) was synthesized from **15k** (0.25 g, 0.67 mmol) according to the general procedure C. Purified by FCC using hexanes/AcOEt/Et_3N 8:2:0.1. The free base was dissolved in dry Et_2O (50 mL) and the resulting solution was cooled to –30 °C. A solution of hydrogen chloride in Et_2O (1.0 M) was added dropwise under stirring until the pH of the mixture reached approximately 2. A precipitate formed upon acidification, and the reaction flask was stored in a freezer for 30 minutes to ensure complete precipitation. The resulting solid was collected by vacuum filtration, washed thoroughly with cold dry Et_2O (3 × 10 mL), and dried under high vacuum for 3 h to afford the hydrochloride salt as an off-white solid (0.137 g, 73%); R_f = 0.3 (free base - hexanes/AcOEt 7:3); ^{1}H NMR (250 MHz, MeOD) δ: 7.83 (dd, $^{3}J_{H-H}$ = 8.0, $^{4}J_{H-F}$ = 6.6 Hz, 1H, ArH6), 7.50 – 7.41 (m, 6H, ArH3, PhH), 7.39 (m, ArH5), 6.21 (d, J = 4.4 Hz, 1H, H7); ^{13}C NMR (63 MHz, MeOD) δ: 164.53 (d, $^{1}J_{C-F}$ = 258.0 Hz, ArC2), 147.50 (d, $^{3}J_{C-F}$ = 7.7 Hz, ArC4), 137.17 (PhC8), 135.52 (ArC6), 131.12 (PhC11), 130.49 (PhC10/12), 128.86 (PhC9/13), 124.88 (d, $^{4}J_{C-F}$ = 3.6 Hz, ArC1), 115.99 (d, $^{2}J_{C-F}$ = 21.6 Hz, ArC3), 114.28 (C≡N), 87.68 (d, $^{4}J_{C-F}$ = 1.8 Hz, C7); Elemental analysis calcd. (%) for $C_{14}H_{12}ClFN_2O$: C, 60.33; H, 4.34; N, 10.05; found: C, 59.99; H, 4.61; N, 9.87.

General procedure for the synthesis of alcohols 6a, 6b, 6d, 6f, 6h and 6i (D)

Activated MnO_2 (10 equiv.) was added in one portion to a solution of the corresponding alcohol (1 equiv.) in dry DCM (0.1 M) under argon. The reaction mixture was stirred at room temperature overnight, resulting in a black suspension. Upon completion (TLC monitoring, anisaldehyde stain), the reaction was filtered through a pad of Celite, and the filtrate was concentrated under reduced pressure. The residue was dried under high vacuum to afford the crude ketone, which was used without further purification.

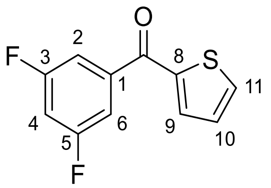

(3,5-difluorophenyl)(thiophen-2-yl)methanone (6a) was synthesized from **3a** (0.35 g, 1.55 mmol) according to the general procedure D to afford an off-white solid (0.34 g, 99%); R_f = 0.8 (hexanes/AcOEt 8:2); ^{1}H NMR (250 MHz, $CDCl_3$) δ: 7.77 (d, $^3J_{H\text{-}H}$ = 4.9 Hz, 1H, thiopheneH11), 7.65 (dd, $^3J_{H\text{-}H}$ = 3.8, $^3J_{H\text{-}H}$ = 1.1 Hz, 1H, thiopheneH9), 7.40 – 7.33 (m, 2H, ArH2/H6), 7.18 (dd, $^3J_{H\text{-}H}$ = 4.9, $^3J_{H\text{-}H}$ = 3.8 Hz, 1H, thiopheneH10), 7.03 (tt, $^3J_{H\text{-}F}$ = 8.6, $^4J_{H\text{-}H}$ = 2.4 Hz, 1H, ArH4); ^{13}C NMR (63 MHz, $CDCl_3$) δ: 185.42 (C=O), 162.80 (dd, $^1J_{C\text{-}F}$ = 251.3, $^2J_{C\text{-}F}$ = 11.8 Hz, ArC3/C5), 142.48 (thiophenC8), 140.97 (t, $^3J_{C\text{-}F}$ = 8.0 Hz, ArC1), 135.40 (thiophenC9), 135.29 (thiophenC11), 128.38 (thiophenC10), 112.28 (m, AXX'-system, ArC2/C6), 107.64 (t, $^2J_{C\text{-}F}$ = 25.3 Hz, ArC4); Elemental Analysis calcd. (%) for $C_{11}H_6F_2OS$: C, 58.92; H, 2.70; found: C, 58.83; H, 2.77.

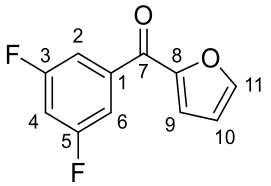

(3,5-difluorophenyl)(furan-2-yl)methanone (6b) was synthesized from **3b** (0.10 g, 0.48 mmol) according to the general procedure D to afford a yellow oil (98 mg, 99%); R_f = 0.5 (hexanes/AcOEt 8:2); ^{1}H NMR (250 MHz, $CDCl_3$) δ: 7.76 – 7.70 (m, 1H, furaneH12), 7.59 – 7.47 (m, 2H, ArH2/H6), 7.32 (d, $^3J_{H\text{-}H}$ = 3.7 Hz, 1H, furaneH10), 7.03 (tt, $^3J_{H\text{-}F}$ = 8.5, $^4J_{H\text{-}H}$ = 2.4 Hz, 1H, ArH4), 6.63 (dd, $^3J_{H\text{-}H}$ = 3.6, $^3J_{H\text{-}H}$ = 1.7 Hz, 1H, furaneH11); ^{13}C NMR (63 MHz, $CDCl_3$) δ: 179.46 (C=O), 162.87 (dd, $^1J_{C\text{-}F}$ = 250.6, $^2J_{C\text{-}F}$ = 11.7 Hz, ArC3/C5), 151.85 (furaneC9), 147.75 (furaneC12), 139.87 (t, $^3J_{C\text{-}F}$ = 8.1 Hz, ArC1), 121.28 (furaneC10), 112.75 (furaneC11, overlapping with ArC2/C6), 112.55 (m, AXX'-system, ArC2/C6), 108.03 (t, $^2J_{C\text{-}F}$ = 25.4 Hz, ArC4); Elemental analysis calcd. (%) for $C_{11}H_6F_2O_2$: C, 63.47; H, 2.91; found: C, 63.21; H, 3.13.

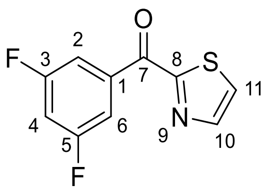

(3,5-difluorophenyl)(thiazol-2-yl)methanone (6d) was synthesized from **3d** (0.05 g, 0.22 mmol) according to the general procedure D to afford a white solid (48 mg, 97%); R_f = 0.55 (hexanes/AcOEt 9:1); ^{1}H NMR (500 MHz, $CDCl_3$) δ: 8.12 – 8.09 (m, 3H, thiazoleH10, ArH2/H6), 7.77 (d, $^3J_{H\text{-}H}$ = 3.1 Hz, 1, thiazoleH11), 7.08 (tt, $^3J_{H\text{-}F}$ = 8.4, $^4J_{H\text{-}H}$ = 2.4 Hz, 1H, ArH4); ^{13}C NMR (126 MHz, $CDCl_3$) δ: 181.34 (C=O), 166.90 (thiazoleC8), 162.81 (dd, $^1J_{C\text{-}F}$ = 250.0, $^3J_{C\text{-}F}$ = 12.0 Hz, ArC3/C5), 145.26 (thiazoleC10), 137.81 (t,

$^3J_{C-F}$ = 8.6 Hz, ArC1), 127.16 (thiazoleC11), 114.30 (m, AXX'-system, ArC2/C6), 109.03 (t, $^2J_{C-F}$ = 25.4 Hz, ArC4); Elemental analysis calcd. (%) for $C_{10}H_5F_2NOS$: C, 53.33; H, 2.24; N, 6.22; found: C, 53.37; H, 2.26; N, 6.19.

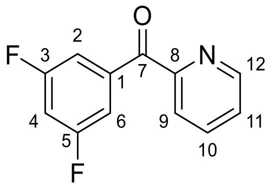

(3,5-difluorophenyl)(pyridin-2-yl)methanone (6f) was synthesized from **3f** (0.25 g, 1.13 mmol) according to the general procedure D to afford a beige solid (0.24 g, 97%); R_f = 0.65 (hexanes/AcOEt 8:2); ^{1}H NMR (250 MHz, $CDCl_3$) δ: 8.72 (dd, $^3J_{H-H}$ = 4.9, $^4J_{H-H}$ = 1.3 Hz, 1H, pyridineH12), 8.08 (dt, $^3J_{H-H}$ = 7.9, $^4J_{H-H}$ = 1.2 Hz, 1H, pyridineH10), 7.92 (td, $^3J_{H-H}$ = 7.8, $^4J_{H-H}$ = 1.7 Hz, 1H, pyridineH9), 7.74 –7.64 (m, 2H, ArH2/H6), 7.52 (ddd, $^3J_{H-H}$ = 7.6, $^3J_{H-H}$ = 4.8, $^4J_{H-H}$ = 1.3 Hz, 1H, pyridineH11), 7.03 (tt, $^3J_{H-F}$ = 8.5, $^4J_{H-H}$ = 2.4 Hz, 1H, ArH4); ^{13}C NMR (63 MHz, $CDCl_3$) δ: 190.82 (C=O), 162.62 (dd, $^1J_{C-F}$ = 249.7, $^2J_{C-F}$ = 11.7 Hz, ArC3/C5), 154.04 (pyridineC8), 148.73 (pyridineC12), 139.12 (t, $^3J_{C-F}$ = 8.3 Hz, ArC1), 137.46 (pyridineC10), 126.94 (pyridineC11), 124.93 (pyridineC9), 114.20 (m, AXX'-system, ArC2/C6), 108.23 (t, $^2J_{C-F}$ = 25.4 Hz, ArC4); Elemental analysis calcd. (%) for $C_{12}H_7F_2NO$: C, 65.76; H, 3.22; N, 6.39; found: C, 65.59; H, 3.49; N, 6.26.

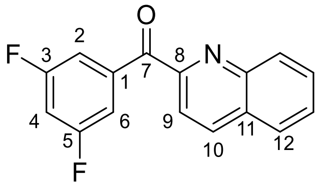

(3,5-difluorophenyl)(quinolin-2-yl)methanone (6h) was synthesized from **3h** (0.15 g, 0.55 mmol) according to the general procedure D to afford a white solid (0.14 g, 93%); R_f = 0.7 (hexanes/AcOEt 8:2); ^{1}H NMR (500 MHz, $CDCl_3$) δ: 8.36 (d, $^3J_{H-H}$ = 8.5 Hz, 1H, quinolineH10), 8.21 (d, $^3J_{H-H}$ = 8.5 Hz, 1H, quinolineH15), 8.15 (d, $^3J_{H-H}$ = 8.5 Hz, 1H, quinolineH9), 7.91 (dd, $^3J_{H-H}$ = 8.3, $^4J_{H-H}$ = 1.5 Hz, 1H, quinolineH12), 7.89 – 7.86 (m, 2H, ArH2/H6), 7.81 (ddd, J_1 = 8.5, J_2 = 6.9, J_3 = 1.5 Hz, 1H, quinolineH14), 7.69 (ddd, $^3J_{H-H}$ = 8.1, $^3J_{H-H}$ = 6.9, $^4J_{H-H}$ = 1.2 Hz, 1H, quinolineH13), 7.07 (tt, $^3J_{H-F}$ = 8.5, $^4J_{H-H}$ = 2.4 Hz, 1H, ArH4); ^{13}C NMR (126 MHz, $CDCl_3$) δ: 190.72 (C=O), 162.67 (dd, $^1J_{C-F}$ = 249.6, $^2J_{C-F}$ = 11.8 Hz, ArC3/C5), 153.60 (quinolineC8), 146.80 (quinolineC16), 139.12 (t, $^3J_{C-F}$ = 8.3 Hz, ArC1), 137.53 (quinolineC10), 130.75 (quinolineC14), 130.50 (quinolineC15), 129.26 (quinolineC11), 129.06 (quinolineC12), 127.82 (quinolineC13), 120.77 (quinolineC9), 114.60 (m, AXX'-system, ArC2/C6), 108.31 (t, $^2J_{C-F}$ = 25.4 Hz, ArC4); Elemental analysis calcd. (%) for $C_{16}H_9F_2NO$: C, 71.37; H, 3.37; N, 5.20; found: C, 71.12; H, 3.46; N, 5.03.

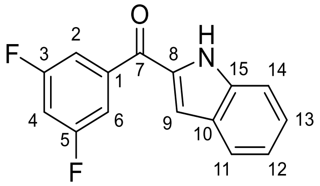

(3,5-difluorophenyl)(1*H*-indol-2-yl)methanone (6i) was synthesized from **3i** (0.05 g, 0.19 mmol) according to the general procedure D to afford a brown solid (48 mg, 97%); R_f = 0.8 (hexanes/AcOEt 8:2); ^{1}H NMR (250 MHz, $CDCl_3$) δ: 9.64 (s, 1H, indoleN*H*), 7.74 (d, $^3J_{H-H}$ = 8.1 Hz, 1H, indoleH11), 7.55 – 7.46 (m, 3H, ArH2/H6, indoleH), 7.40 (t, J = 7.7 Hz, 1H, indoleH), 7.23 – 7.15 (m, 2H, indoleH), 7.09 – 7.04 (tt,

$^{3}J_{H-F}$ = 8.6, $^{4}J_{H-H}$ = 2.4 Hz, 1H, ArH4); ^{13}C NMR (63 MHz, CDCl3) δ: 184.51 (C=O), 162.90 (dd, $^{1}J_{C-F}$ = 251.0, $^{3}J_{C-F}$ 12.0 Hz, ArC3/C5), 140.85 (t, $^{3}J_{C-F}$ = 8.2 Hz, ArC1), 138.14 (indoleC15), 133.48 (indoleC8), 127.71 (indoleC10), 127.34 (indoleC), 123.55 (indoleC), 121.53 (indoleC), 113.62 (indoleC), 112.60 (indoleC), 112.40 (m, AXX'-system, ArC2/C6), 107.74 (t, $^{2}J_{C-F}$ = 25.2 Hz, ArC4); Elemental analysis calcd. (%) for $C_{15}H_{9}F_{2}NO$: C, 70.04; H, 3.53; N, 5.45; found: C, 69.83; H, 3.75; N, 5.21.

General procedure for the synthesis of oximes 7a, 7b, 7d, 7f, 7h and 7i (E)

In a round-bottom flask, the corresponding ketone (1 eq) was added, followed by EtOH to adjust the reaction concentration to 0.38 M, hydroxylamine hydrochloride (3 eq), and sodium carbonate (3 eq). The reaction mixture was stirred in an oil bath at 85 °C (reflux) for the appropriate time. After completion, the mixture was cooled to room temperature and filtered through a paper filter to remove white solids. The filter was washed with EtOH, and the filtrate was concentrated under reduced pressure. The resulting solid was dissolved in ethyl acetate and filtered again to remove any remaining white solids. The organic phase was concentrated under reduced pressure and dried under high vacuum to afford the desired oxime.

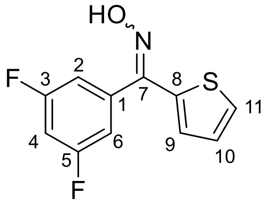

(3,5-difluorophenyl)(thiophen-2-yl)methanone oxime (7a) was synthesized from **6a** (0.05 g, 0.22 mmol) according to the general procedure E. The reaction was stirred at reflux for 48 h, with 1 additional equivalent of both reagents (hydroxylamine hydrochloride and sodium carbonate) added during the process, affording the product as a white solid (45 mg, 84%); R_f = 0.35 (hexanes/AcOEt 9:1). The product is a mixture of ***E*** and ***Z*** conformers. Major/Minor: 1.3/1

Major Isomer

^{1}H NMR (500 MHz, $CDCl_3$) δ: 9.13 (brs, 1H, -O*H*), 7.56 (d, $^{3}J_{H-H}$ = 5.1 Hz, 1H, thiopheneH11), 7.15 (d, $^{3}J_{H-H}$ = 3.8 Hz, 1H, thiopheneH9), 7.01 – 6.99 (m, 3H, ArH2/H6, thiopheneH10), 6.87 – 6.74 (m, 1H, ArH4); ^{13}C NMR (126 MHz, $CDCl_3$) δ: 162.91 (dd, $^{1}J_{C-F}$ = 250.1, $^{3}J_{C-F}$ 12.4 Hz, ArC3/C5), 150.37 (C*7*), 139.04 (t, $^{3}J_{C-F}$ =9.0 Hz, ArC1), 138.64 (thiopheneC8), 132.94 (thiopheneC11), 132.10 (thiopheneC10),126.01 (thiopheneC9), 112.73 (m, AXX'-system, ArC2/C6), 105.07 (t, $^{2}J_{C-F}$ = 25.2 Hz, ArC4).

Minor Isomer

^{1}H NMR (500 MHz, $CDCl_3$) δ: 9.13 (brs, 1H, -O*H*), 7.27 (d, $^{3}J_{H-H}$ = 5.1 Hz, 1H, thiopheneH11), 6.93 – 6.90 (m, 3H, ArH2/H6, thiopheneH9),-6.87 – 6.74 (m, 2H, ArH4, thiopheneH10); ^{13}C NMR (126 MHz, $CDCl_3$) δ: 162.91 (dd, $^{1}J_{C-F}$ = 250.1, $^{3}J_{C-F}$ 12.4 Hz, Ar*C3*/*C5*), 151.78 (C*7*), 138.64 (thiopheneC8), 134.83 (t, $^{3}J_{C-F}$ =9.0 Hz, ArC1), 129.79 (thiopheneC11), 128.13 (thiopheneC10), 127.48 (thiopheneC9), 112.36 (m, AXX'-system, ArC2/C6), 105.07 (t, $^{2}J_{C-F}$ = 25.2 Hz, ArC4).

Elemental analysis calcd. (%) for $C_{11}H_{7}F_{2}NOS$: C, 55.23; H, 2.95; N, 5.85; found: C, 55.11; H, 3.12; N, 6.02.

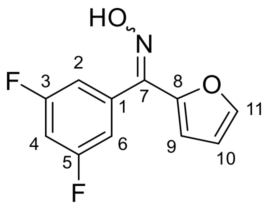

(3,5-difluorophenyl)(furan-2-yl)methanone oxime (7b) was synthesized from **6b** (0.05 g, 0.24 mmol) according to the general procedure X. After stirring at reflux for 2 h, the reaction mixture was left to stir at room temperature for an additional 16 h. The crude product was purified by FCC using hexanes/AcOEt 9:1; white solid (51 mg, 95%); R_f = 0.5 (hexanes/AcOEt 8:2). The product is a mixture of ***E*** and ***Z*** conformers. Major/Minor ~ 2:1

Major Isomer:

^{1}H NMR (500 MHz, $CDCl_3$) δ: 8.63 (brs, 1H, -O*H*), 7.54-7.53 (m, 1H, furanH11), 7.51 (~dd, J_1 = 1.6Hz, J_2 = 0.6 Hz, 1H, furanH), 7.15 – 7.11 (m, 2H, ArH2/H6), 6.91 (qt, $^3J_{H-F}$ = 8.9, $^4J_{H-H}$ = 2.3 Hz, 1H, ArH4); 6.62 (dd, *J* = 3.6, 1.7 Hz, 1H, furanH); ^{13}C NMR (126 MHz, $CDCl_3$) δ:162.75 (dd, $^1J_{C-F}$ = 248.4, $^3J_{C-F}$ 12.4 Hz, ArC3/C5), 145.76 (C7), 144.77 (furanC8), 143.47 (furanC11), 137.43 (t, $^3J_{C-F}$ =9.0 Hz, ArC1), 119.72 (furanC), 112.36 (dd, $^2J_{C-F}$ = 16.5, $^4J_{C-F}$ = 8.8 Hz, ArC2/C6), 112.31 (furan*C*, overlapping with ArC2/C6), 104.85 (t, $^2J_{C-F}$ = 25.2 Hz, ArC4).

Minor Isomer:

^{1}H NMR (500 MHz, $CDCl_3$) δ: 8.63 (s, 1H, -O*H*), 7.54 (d, $^3J_{H-H}$ = 0.8 Hz, 1H, furaneH11), 7.05 – 7.02 (m, 2H, ArH2/H6), 6.91 (qt, J_1 = 8.9, J_2 = 2.3 Hz, 1H, ArH4) 6.45 (dd, J_1 = 3.5, J_2 = 1.8 Hz, 1H, furaneH), 6.37 (dd, J_1= 3.3, J_2 = 0.66 Hz, 1H, furaneH); ^{13}C NMR (126 MHz, $CDCl_3$) δ: 162.75 (dd, $^1J_{C-F}$ = 248.4, $^3J_{C-F}$ 12.4 Hz, ArC3/C5), 146.66(C7), 143.72 (furaneC8), 143.47 (furaneC11), 137.43 (t, $^3J_{C-F}$ =9.0 Hz, ArC1),123.16 (furaneC10), 112.36 (dd, $^2J_{C-F}$ = 16.5, $^4J_{C-F}$ = 8.8 Hz, ArC2/C6), 111.68 (furaneC9), 104.85 (t, $^2J_{C-F}$ = 25.2 Hz, ArC4).

Elemental analysis calcd. (%) for $C_{11}H_7F_2NO_2$: C, 59.20; H, 3.16; N, 6.28; found: C, 59.02; H, 3.39; N, 6.11.

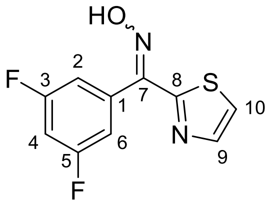

(3,5-difluorophenyl)(thiazol-2-yl)methanone oxime (7d) was synthesized from **6d** (0.1 g, 0.44 mmol) according to the general procedure X. The reaction was stirred at reflux for 16 h, brought to room temperature, and concentrated under reduced pressure. The afforded solid was dissolved in AcOEt and washed with water. The organic phase was concentrated under reduced pressure and the crude product was purified by FCC using hexanes/AcOEt 9:1; white solid (93 mg, 87%); R_f = 0.35 (hexanes/AcOEt 9:1). The product is a mixture of ***E*** and ***Z*** conformers. Major/Minor: 1.2/1

Major Isomer:

^{1}H NMR (250 MHz, $CDCl_3$) δ: 8.76 (brs, 1H, -O*H*), 8.06 (d, $^3J_{H-H}$ = 3.2 Hz, 1H, thiazoleH9), 7.67 (d, $^3J_{H-H}$ = 3.2 Hz, 1H, thiazoleH10), 7.28 – 7.19 (m, 2H, ArH2/H6), 6.95 – 6.85 (m ,1H, ArH4); ^{13}C NMR (63 MHz,

$CDCl_3$) δ: 162.79 (dd, $^1J_{C-F}$ = 248.1, $^3J_{C-F}$ = 11.6 Hz, ArC3/C5), 151.17 (thiazoleC8), 143.74 (C7), 142.52 (thiazoleC9), 137.67 (t, $^3J_{C-F}$ = 8.3 Hz, ArC1), 123.27 (thiazoleC10), 112.83 (m, AXX'-system, ArC2/C6, two isomers overlapping), 106.64 – 103.04 (m, ArC4, two isomers overlapping).

Minor Isomer:

^{1}H NMR (250 MHz, $CDCl_3$) δ: 8.76 (brs, 1H, -O*H*), 7.89 (d, $^3J_{H-H}$ = 3.0 Hz, 1H, thiazoleH9), 7.40 (d, $^3J_{H-H}$ = 3.0 Hz, 1H, thiazoleH10), 7.10 – 7.06 (m, 2H, ArH2/H6), 6.95 – 6.85 (m 1H, ArH4); ^{13}C NMR (63 MHz, $CDCl_3$) δ: 162.79 (dd, $^1J_{C-F}$ = 248.1, $^3J_{C-F}$ = 11.6 Hz, ArC3/C5), 151.17 (thiazoleC8), 143.74 (C7), 142.52 (thiazoleC9), 137.67 (t, $^3J_{C-F}$ = 8.3 Hz, ArC1), 123.27 (thiazoleC10), 112.83 (m, AXX'-system, ArC3/C5, two isomers overlapping), 106.64 – 103.04 (m, ArC4, two isomers overlapping).

Elemental analysis calcd. (%) for $C_{10}H_6F_2N_2OS$: C, 50.00; H, 2.52; N, 11.66; found: C, 49.84; H, 2.58; N, 11.55.

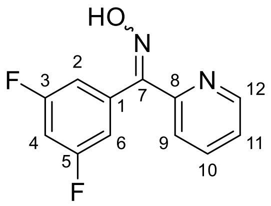

(3,5-difluorophenyl)(pyridin-2-yl)methanone oxime (7f) was synthesized from **6f** (0.185 g, 0.84 mmol) according to the general procedure E. The reaction was stirred at reflux for 2 h and afforded the product as a red solid (177 mg, 90%); R_f = 0.4 (hexanes/AcOEt 7:3). The product is a mixture of ***E*** and ***Z*** conformers. Major/Minor ~ 3.5:1

Major Isomer

^{1}H NMR (500 MHz, $CDCl_3$) δ: 9.95 (brs, 1H, -O*H*), 8.63 (dd, $^3J_{H-H}$ = 4.9, $^4J_{H-H}$ = 1.7 Hz, 1H, pyridineH12), 7.74 (t, $^3J_{H-H}$ = 7.8 Hz, 1H, pyridineH10), 7.67 (d, $^3J_{H-H}$ = 8.0 Hz, 1H, pyridineH9), 7.32 (t, $^3J_{H-H}$ = 5.0 Hz, 1H, pyridineH11), 6.99 – 6.92 (m, 2H, ArH2/H6), 6.85 (tt, *J* $^3J_{H-F}$ = 9.0, $^4J_{H-H}$ = 2.2 Hz, 1H, ArH4); ^{13}C NMR (126 MHz, $CDCl_3$) δ: 162.91 (dd, $^1J_{C-F}$ = 249.01, $^3J_{C-F}$ = 11.7 Hz, ArC3/C5), 153.68 (C7), 149.48 (pyridineC8), 147.96 (pyridineC12), 137.11 (pyridineC10), 134.57 (t, $^3J_{C-F}$ = 8.6 Hz, ArC1), 124.19 (pyridineC11), 122.82 (pyridineC9), 112.91 (m, AXX'-system, ArC2/C6),104.75 (t, $^2J_{C-F}$ = 25.4 Hz, ArC4).

Minor Isomer

^{1}H NMR (500 MHz, $CDCl_3$) δ: 9.95 (brs, 1H, -O*H*), 8.70 (dd, $^3J_{H-H}$ = 4.9, $^4J_{H-H}$ = 1.7 Hz, 1H, pyridineH12), 7.88 (td, $^3J_{H-H}$ = 7.8 Hz, $^4J_{H-H}$ = 1.7 Hz, 1H, pyridineH10), 7.46 (dd, $^3J_{H-H}$ = 7.7, $^4J_{H-H}$ = 0.9 Hz, 1H, pyridineH9), 7.40 – 7.38 (m, 1H, pyridineH11), 7.05 – 7.00 (m, 2H, ArH3/H5), 6.85 (tt, *J* $^3J_{H-F}$ = 9.0, $^4J_{H-H}$ = 2.2 Hz, 1H, ArH4); ^{13}C NMR (126 MHz, $CDCl_3$) δ: 162.91 (dd, $^1J_{C-F}$ = 249.01, $^3J_{C-F}$ = 11.7 Hz, ArC3/C5), 154.53 (C7), 149.64 (pyridineC8), 148.04 (pyridineC12), 137.78 (pyridineC10), 134.57 (t, $^3J_{C-F}$ = 8.6 Hz, ArC1), 124.89 (pyridineC11), 124.86 (pyridineC9), 111.90 (m, AXX'-system, ArC2/C6), 104.75 (t, $^2J_{C-F}$ = 25.4 Hz, ArC4).

Elemental analysis calcd. (%) for C12H8F2N2O: C, 61.54; H, 3.44; N, 11.96; found: C, 61.21; H, 3.57; N, 11.88.

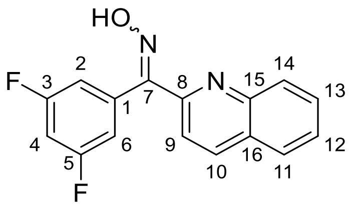

(3,5-difluorophenyl)(quinolin-2-yl)methanone oxime (7h) was synthesized from **6h** (0.05 g, 0.19 mmol) according to the general procedure E. The reaction was stirred at reflux for 2 h and afforded the product as a white solid (47 mg, 89%); R_f = 0.45 (hexanes/AcOEt 8:2). A single isomer was observed in the ^{1}H NMR spectrum.

^{1}H NMR (250 MHz, $CDCl_3$) *δ*: 9.77 (brs, 1H, -O*H*), 8.34 (d, $^{3}J_{H\text{-}H}$ = 8.6 Hz, 1H, quinolineH10), 8.15 (d, $^{3}J_{H\text{-}H}$ = 8.5 Hz, 1H, quinolineH14), 7.98 – 7.79 (m, 2H, quinolineH11/H13), 7.71 (d, $^{3}J_{H\text{-}H}$ = 7.6 Hz, 1H, quinolineH9), 7.40 (d, $^{3}J_{H\text{-}H}$ = 8.6 Hz, 1H, quinolineH12), 7.10 – 7.06 (m, 2H, ArH2/H6), 6.92 (tt, *J* $^{3}J_{H\text{-}F}$ = 9.0, $^{4}J_{H\text{-}H}$ = 2.3 Hz, 1H, ArH4); ^{13}C NMR (63 MHz, $CDCl_3$) *δ*: 163.08 (dd, $^{1}J_{C\text{-}F}$ = 249.4, $^{3}J_{C\text{-}F}$ = 12.7 Hz, ArC3/C6), 151.19 (*C7*), 145.31 (quinolineC8/C15), 138.57 (t, $^{3}J_{C\text{-}F}$ = 8.3 Hz, ArC1), 138.29 (quinolineC10), 131.30 (quinolineC13), 128.62 (quinolineC11/C14), 128.07 (quinolineC16), 127.89 (quinolineC12), 121.28 (quinolineC9), 112.09 (m, AXX'-system, ArC3/C5), 104.84 (t, $^{2}J_{C\text{-}F}$ = 25.3 Hz, ArC4); Elemental analysis calcd. (%) for $C_{16}H_{10}F_2N_2O$: C, 67.60; H, 3.55; N, 9.85; found: C, 67.72; H, 3.61; N, 10.02.

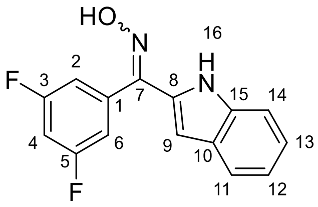

(3,5-difluorophenyl)(1H-indol-2-yl)methanone oxime (7i) was synthesized from **6i** (0.05 g, 0.19 mmol) according to the general procedure E. The reaction was stirred at reflux for 48 h, with 1 additional equivalent of both reagents (hydroxylamine hydrochloride and sodium carbonate) added during the process, affording the product as an orange-brown solid (47 mg, 89%); R_f = 0.4 (hexanes/AcOEt 8:2). The product is a mixture of ***E*** and ***Z*** conformers. Major/Minor: 1.2/1

Major Isomer:

^{1}H NMR (500 MHz, $CDCl_3$) *δ*: 8.86 (s, 1H, indoleN*H*), 7.47 (d, $^{3}J_{H\text{-}H}$ = 7.7 Hz, 1H, indoleH11), 7.26 – 7.24 (m, 1H, indoleH), 7.18 – 7.15 (m, 1H, indoleH), 7.05 – 6.95 (m, 3H, ArH2/H6, indoleH), 6.91 – 6.85 (m,1H, ArH4), 6.30 (s, 1H, indoleH9); ^{13}C NMR (126 MHz, $CDCl_3$) *δ*: 162.85 (dd, $^{1}J_{C\text{-}F}$ = 249.5, $^{3}J_{C\text{-}F}$ = 12.6 Hz, ArC3/C5), 149.78 (C*7*), 138.51(t, $^{3}J_{C\text{-}F}$ = 9.5 Hz, ArC1), 137.12 (indoleC8/C15),127.99 (indoleC10), 124.61 (indoleC), 121.57 (indoleC), 120.54 (indoleC), 112.58 (m, AXX'-system, ArC2/C6), 111.23 (indoleC), 107.81 (indoleC9), 105.13 (t, $^{2}J_{C\text{-}F}$ = 25.3, ArC4).

Minor Isomer:

^{1}H NMR (500 MHz, $CDCl_3$) *δ*: 10.14 (s, 1H, indoleN*H*), 7.55 (d, $^{3}J_{H\text{-}H}$ = 8.0 Hz, 1H, indoleH11), 7.38 (d, *J* = 8.2 Hz, 1H, indoleH), 7.26 – 7.24 (m, 1H, indoleH), 7.14 – 7.05 (m, 3H, ArH2/H6, indoleH), 6.91 – 6.85 (m,1H, ArH4), 6.52 (s, 1H, indoleH9); ^{13}C NMR (126 MHz, $CDCl_3$) *δ*: 162.85 (dd, $^{1}J_{C\text{-}F}$ = 249.5, $^{3}J_{C\text{-}F}$ = 12.6 Hz, ArC3/C5), 149.05 (C*7*), 134.10 (t, $^{3}J_{C\text{-}F}$ = 9.5 Hz, ArC1), 132.41 (indoleC), 128.92 (indoleC*)*, 126.27

(indoleC), 125.19 (indoleC), 122.02 (indoleC), 120.86 (indoleC), 112.90 (m, AXX'-system, ArC2/C6), 111.86 (indoleC), 110.46 (indoleC9), 105.13 (t, $^{2}J_{C\text{-}F}$ = 25.3, ArC4).

Elemental analysis calcd. (%) for $C_{15}H_{10}F_2N_2O$: C, 66.18; H, 3.70; N, 10.29; found: C, 65.92; H, 3.81; N, 10.15.

General procedure for the synthesis of chlorides 8d,8f *via* MsCl or $SOCl_2$ (F)

- MsCl (1.2 eq) was added dropwise to an ice-cold solution of the corresponding alcohol (1 eq) in DCM (0.25 M) and Et_3N (1.6 eq) and the reaction was slowly allowed to reach room temperature leaving it to stir overnight. The mixture was transferred to a separatory funnel with DCM and washed with aq. $NaHCO_3$ (sat) solution (x1), water (x1) and brine (x1), dried over $MgSO_4$, filtered and evaporated to dryness. The crude chloride was sufficiently pure by TLC and used directly for the formation of the corresponding thioester.

- $SOCl_2$ (10 eq.) was added dropwise to an ice-cold solution of the corresponding alcohol (1 eq) in DCM (0.2 M). After the addition was complete, DMF (0.65 eq.) were added and the reaction was allowed to reach room temperature. The reaction was monitored with TLC and when the SM was completely consumed (1 – 15 h) the reaction was transferred to a separatory funnel with DCM and was successively washed with sat. $NaHCO_3$ (x1), water (x1) and brine (x1). The organic layer was dried over Na_2SO_4, filtered and evaporated to dryness. The crude chloride was sufficiently pure by TLC and ^{1}H-NMR and used directly for the formation of the corresponding thioester.

The chlorides were found to be sufficiently pure according to ^{1}H NMR analysis and were therefore used without further purification. A representative ^{1}H NMR spectrum is provided for compound **8f**.

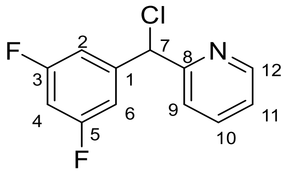

2-(chloro(3,5-difluorophenyl)methyl)pyridine (8f) was synthesized from **3f** (0.5 g, 2.26 mmol) according to the general procedure F ($SOCl_2$). No purification needed; Blue-green oil (0.54 g, quant); R_f = 0.45 (hexanes/AcOEt 8:2); ^{1}H NMR (500 MHz, $CDCl_3$) δ: 8.59 (d, J = 4.3 Hz, 1H, pyridineH12), 7.76 (dt, J_1 = 7.8 Hz, J_2 = 1.7 Hz, 1H, pyridineH10), 7.55 (d, J = 7.8 Hz, 1 H, pyridineH9), 7.26 (overlap with $CDCl_3$ peak, 1H, pyridineH11), 7.06 – 6.96 (m, 2H, ArH2/H6), 6.74 (tt, $^{3}J_{H\text{-}F}$ = 8.8 Hz, $^{4}J_{H\text{-}H}$ = 2.2 Hz, 1H, ArH4), 6.08 (s, 1H, H*7*).

General procedure for the synthesis of thioester 9d,9f (G)

Potassium thioacetate (2 eq) was added to a solution of the corresponding chloride (1 eq) in DMF (0.22 M) at room temperature and the reaction was allowed to stir, while monitoring with TLC. After full consumption of the SM (2h to overnight), the reaction was poured to water and extracted with ethyl acetate or ether (3x). The combined organic layers were dried over sodium sulfate, filtered and evaporated to dryness. The crude thioester was purified by FCC using the appropriate hexanes/AcOEt solvent system.

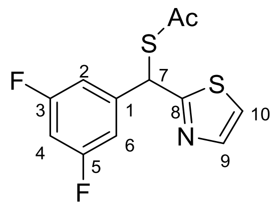

S-((3,5-difluorophenyl)(thiazol-2-yl)methyl) ethanethioate (9d) was synthesized from the corresponding crude chloride **8d** (0.21 g, 0.85 mmol) according to the general procedure G. Purified by FCC using hexanes/AcOEt 98:2 to 9:1; Brown-red oil (0.182 g, 75%); R_f = 0.35 (hexanes/AcOEt 8:2; ^{1}H NMR (500 MHz, $CDCl_3$) δ: 7.78 (d, J = 3.3 Hz, 1H, thiazoleH9), 7.31 (d, J = 3.3 Hz, 1H, thiazoleH10), 7.02 – 6.98 (m, 2H, ArH2/H6), 6.73 (tt, $^3J_{H\text{-}F}$ = 8.9 Hz, $^4J_{H\text{-}H}$ = 2.2 Hz, 1H, ArH4), 6.12 (s, 1H, H7), 2.39 (s, 3H, -SC(=O)CH_3); ^{13}C NMR (126 MHz, $CDCl_3$) δ: 192.28 (-S(C=O)CH_3), 168.25 (thiazoleC8), 163.11 (dd, $^1J_{C\text{-}F}$ = 249.3 Hz, $^3J_{C\text{-}F}$ = 12.5 Hz, ArC3/C5), 143.33 (thiazoleC9), 142.84 (t, $^3J_{C\text{-}F}$ = 8.7 Hz, ArC1), 120.37 (thiazoleC10), 111.76 (m, AXX'-system, ArC2/C6), 103.94 (t, $^2J_{C\text{-}F}$ = 25.3 Hz, ArC4), 48.3 (t, $^4J_{C\text{-}F}$ = 2 Hz, C7), 29.9 (-S(C=O)CH_3); Elemental analysis calcd. (%) for $C_{12}H_9F_2NOS_2$: C, 50.51; H, 3.18; N, 4.91; found: C, 50.76; H, 3.37; N, 5.07.

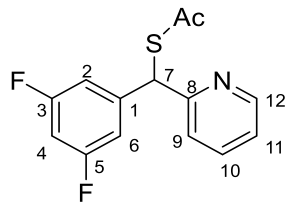

S-((3,5-difluorophenyl)(pyridin-2-yl)methyl) ethanethioate (9f) was synthesized from **8f** (0.54 g, 2.25 mmol) according to the general procedure G. Purified by FCC using hexanes/AcOEt 9:1; orange solid (0.284 g, 45%); R_f = 0.45 (hexanes/AcOEt 8:2); ^{1}H NMR (500 MHz, $CDCl_3$) δ: 8.63 (d, J = 4.7 Hz, 1H, pyridineH12), 7.66 (dt, J_1 = 7.7 Hz, J_2 = 1.9 Hz, 1H, pyridine10), 7.31 (d, J = 7.7 Hz, 1 H, pyridineH9), 7.21 (dd, J_1 = 7.7 Hz, J_2 = 4.7 Hz, 1H, pyridineH11), 7.02 – 6.97 (m, 2H, ArH2/H6), 6.67 (tt, $^3J_{H\text{-}F}$ = 8.8 Hz, $^4J_{H\text{-}H}$ = 2.4 Hz, 1H, ArH4), 5.92 (s, 1H, H7), 2.36 (s, 3H, -SCOCH_3); ^{13}C NMR (126 MHz, $CDCl_3$) δ: 194.00 (-SCOCH_3) 163.02 (dd, $^1J_{C\text{-}F}$ = 250 Hz, $^3J_{C\text{-}F}$ = 12.8 Hz, ArC3/C5), 158.48 (pyridineC8), 150.02 (pyridineC12), 144.39 (t, $^3J_{C\text{-}F}$ = 9.2 Hz, ArC1), 137.23 (pyridineC10), 122.99 (pyridineC), 122.86 (pyridineC), 111.68 (m, AXX'-system, ArC2/C6), 103.20 (t, $^2J_{C\text{-}F}$ = 25.3 Hz, ArC4), 52.84 (C7), 30.36 (-SCOCH_3); Elemental analysis calcd. (%) for $C_{14}H_{11}F_2NOS$: C, 60.20; H, 3.97; N, 5.01; found: C, 60.45; H, 4.21; N, 5.13.

General procedure for the synthesis of thioesters 9a, 9b, 9i* and 9i *via* Mistunobu reaction conditions (H)

DIAD (2 eq) was added slowly to an ice-cold solution of triphenylphoshine (2 eq) in THF (0.35 M) and was left to stir for 30 min. Then, a mixture of the corresponding alcohol (1eq) and thioacetic acid (2 eq) dissolved in THF (0.17 M) was added dropwise to the white suspension and the reaction was allowed to reach room temperature slowly and stirred overnight. The mixture was transferred to a separatory funnel with ether and washed with sat. $NaHCO_3$ (x2), water (x1) and brine (x1), dried over sodium sulfate, filtered and evaporated to dryness. The crude mixture was purified by FCC using the appropriate hexanes/AcOEt solvent system.

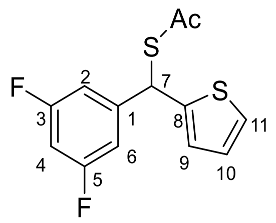

S-((3,5-difluorophenyl)(thiophen-2-yl)methyl) ethanethioate (9a) was synthesized from **3a** (0.5 g, 2.21 mmol) according to the general procedure H. Purified by FCC using hexanes/AcOEt 100:0 to 97:3; brown oil (0.326 g, 52%); R_f = 0.6 (hexanes/AcOEt 9:1); ^{1}H NMR (250 MHz, $CDCl_3$) *δ*: 7.25 (m, 1H, thiopheneH11 – partially overlapping with $CDCl_3$ peak), 6.98 – 6.88 (m, 4H, ArH2/H6 and thiopheneH9/H10), 6.72 (tt, $^3J_{H\text{-}F}$ = 8.8 Hz, $^4J_{H\text{-}H}$ = 2.2 Hz, ArH4), 6.05 (s, 1H, H7), 2.37 (s, 3H, -SCOC*H*$_3$); ^{13}C NMR (63 MHz, $CDCl_3$) *δ*: 193.24 (-S*C*OCH$_3$), 163.07 (dd, $^1J_{C\text{-}F}$ = 249 Hz, $^3J_{C\text{-}F}$ = 12.8 Hz, ArC3/C5), 145.03 (t, $^3J_{C\text{-}F}$ = 8.9 Hz, ArC1), 143.03 (thioheneC8), 127.14 (thiopheneC), 126.81 (thiopheneC), 125.99 (thiopheneC), 111.39 (m, AXX'-system, ArC2/C6), 103.44 (t, $^2J_{C\text{-}F}$ = 25.4 Hz, ArC4), 46.83 (t, $^4J_{C\text{-}F}$ = 2.1 Hz, C7), 30.35 (-SCO*C*H$_3$); Elemental analysis calcd. (%) for $C_{13}H_{10}F_2O_2S_2$: C, 54.91; H, 3.55; found: C, 55.16; H, 3.81.

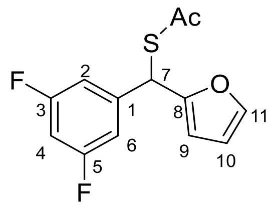

S-((3,5-difluorophenyl)(furan-2-yl)methyl) ethanethioate (9b) was synthesized from **3b** (0.4 g, 1.9 mmol) according to the general procedure H. Purified by FCC using hexanes/AcOEt 100:0 to 95:5; Light brown oil (0.279 g, 55%); R_f = 0.4 (hexanes/AcOEt 9:1); ^{1}H NMR (250 MHz, $CDCl_3$) *δ*: 7.41 – 7.37 (m, 1H, furaneH11), 7.00– 6.87 (m, 2H, ArH2/H6), 6.72 (tt, tt, $^3J_{H\text{-}F}$ = 8.8 Hz, $^4J_{H\text{-}H}$ = 2.2 Hz, ArH4), 6.37 – 6.29 (m, 1H, furaneH9), 6.27 – 6.18 (m 1H, furaneH10), 5.88 (s, 1H, H7), 2.36 (s, 3H, -SCOC*H*$_3$); ^{13}C NMR (63 MHz, $CDCl_3$) *δ*: 193.19 (-S*C*OCH$_3$), 163.03 (dd, $^1J_{C\text{-}F}$ = 248.9 Hz, $^3J_{C\text{-}F}$ = 12.8 Hz, ArC3/C5), 151.41 (furaneC8), 143.20 (furaneC11), 143.01 (t, $^3J_{C\text{-}F}$ = 9.2 Hz, ArC1 – partially overlapping with furaneC11), 111.46 (m, AXX'-system, ArC2/C6), 110.68 (furaneC), 108.65 (furaneC), 103.42 (t, $^2J_{C\text{-}F}$ = 25.4 Hz, ArC4), 44.68 (t, $^4J_{C\text{-}F}$ = 2.2 Hz, C7), 30.34 (s, -SCO*C*H$_3$); Elemental analysis calcd. (%) for $C_{13}H_{10}F_2O_2S$: C, 58.20; H, 3.76; found: C, 57.94; H, 4.01.

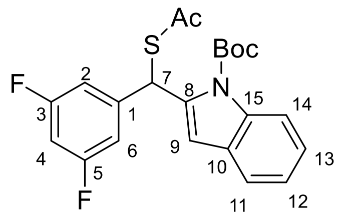

tert*-butyl 2-((acetylthio)(3,5-difluorophenyl)methyl)-1*H*-indole-1-carboxylate (9i*)** was synthesized from **3i (100 mg, 0.28 mmol) according to the general procedure H. Purified by FCC using hexanes/AcOEt 98:2 to 85:15; Light yellow oil (58 mg, 50%); R_f = 0.4 (hexanes/AcOEt 95:5); ^{1}H NMR (500 MHz, $CDCl_3$) *δ*: 8.03 (d, *J* = 8.2 Hz, 1H, indoleH), 7.52 (d, *J* = 7.5 Hz, 1H, indoleH), 7.34 – 7.29 (m, 1 H, indoleH), 7.26 – 7.22 (m, 1H, indoleH, partially overlapping with $CDCl_3$ peak), 6.89 – 6.78 (m, 2H, ArH2/H6), 6.77 – 6.69 (m, 2H, H7 and indoleH9), 6.69 (1H, ArH4, partially overlapping with previous multiplet), 2.42 (s, 3H, -S(CO)C*H*$_3$), 1.57 (s, 9H, -N(CO)OCC*H*$_3$); ^{13}C NMR (126 MHz, $CDCl_3$) *δ*: 194.98 (-S(*C*O)CH$_3$), 164.24 (dd,

$^1J_{C-F}$ = 249.7 Hz, $^3J_{C-F}$ = 12.5 Hz, ArC3/C5), 142.51 (t, $^3J_{C-F}$ = 8.7 Hz, ArC1), 136.63 (indoleC), 136.44 (indoleC), 128.0 (indoleC), 122.83 (indoleC), 120.81 (indoleC), 120.43 (indoleC), 111.77 (m, AXX'-system, ArC2/C6), 111.06 (indoleC), 103.80 (t, $^2J_{C-F}$ = 25.0Hz, ArC4), 103.03 (indoleC9), 45.65 (C7), 30.52 (-S(CO)*C*H3); Elemental analysis calcd. (%) for $C_{22}H_{21}F_2NO_3S$: C, 63.30; H, 5.07; N, 3.36; found: C, 63.12; H, 5.26; N, 3.49.

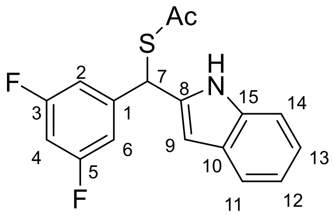

S-((3,5-difluorophenyl)(1H-indol-2-yl)methyl) ethanethioate (9i) TFA (24.5 mmol, 2.79 g, 1.87 ml, 70 eq.) was added dropwise to an ice-cold solution of **9i** (0.15 g, 3.5 mmol, 1 eq.) in DCM (10 ml). After 10 min of stirring, the reaction was allowed to reach room temperature and was stirred for 1h and volatiles were removed under reduced pressure. The residue was azeotroped twice with acetonitrile and the crude mixture was purified by flash column chromatography using hexanes/AcOEt (98:2 to 95:5) to afford the title compound as a brown oil (0.071 g, 48%); R_f = 0.4 (hexanes/AcOEt 8:2); ^{1}H NMR (500 MHz, $CDCl_3$) δ: 8.28 (s, 1H, indoleN*H*), 7. 52 (d, *J* = 7.9 Hz, 1H, indoleH), 7.32 (d, *J* = 8.1 Hz, 1H, indoleH), 7.20 – 7.16 (m, 1 H, indoleH), 7.11 – 7.06 (m, 1H, indoleH), 7.01 – 6.95 (m, 2H, H2/H6), 6.79 (tt, $^3J_{H-F}$ = 8.7 Hz, $^4J_{H-H}$ = 2.1 Hz, 1H, H4), 6.24 (s, 1H, indoleH9), 6.00 (s, 1H, H7), 2.42 (s, 3H, -S(CO)C*H*3); ^{13}C NMR (126 MHz, $CDCl_3$) δ: 194.98 (-S(*C*O)CH3), 164.24 (dd, $^1J_{C-F}$ = 249.7 Hz, $^3J_{C-F}$ = 12.5 Hz, ArC3/C5), 142.51 (t, $^3J_{C-F}$ = 8.7 Hz, ArC1), 136.63 (indoleC), 136.44 (indoleC), 128.0 (indoleC), 122.83 (indoleC), 120.81 (indoleC), 120.43 (indoleC), 111.77 (m, AXX'-system, ArC2/C6), 111.06 (indoleC), 103.80 (t, $^2J_{C-F}$ = 25.0Hz, ArC4), 103.03 (indoleC9), 45.65 (C7), 30.52 (-S(CO)*C*H3); Elemental analysis calcd. (%) for $C_{17}H_{13}F_2NOS$: C, 64.34; H, 4.13; N, 4.41; found: C, 64.09; H, 4.41; N, 4.17.

General procedure for the synthesis of thiols 10a, 10b, 10d, 10f and 10i (I)

The corresponding thioester was dissolved in abs. EtOH (0.12 M) and the solution was degassed by applying the freeze-pump-thaw technique. A solution of NaOH 1M (1.2 eq) was then added dropwise at 0 °C and the reaction was allowed to stir at that temperature until TLC confirmed full consumption of the SM (usually 5-60 min). The solution was then neutralized by the dropwise addition of HCl 1M (1.25 eq) at 0 °C and the reaction was allowed to reach room temperature. The volatiles were removed under reduced pressure and the residue was dissolved in ether or AcOEt, washed with water (x2) and brine (x1), dried over $MgSO_4$, filtered and evaporated to dryness. The crude thiol was purified by FCC using the appropriate hexanes/AcOEt system.

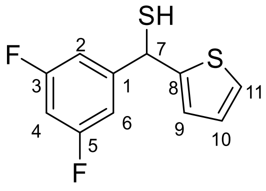

(3,5-difluorophenyl)(thiophen-2-yl)methanethiol (10a) was synthesized from **9a** (50 mg, 0.18 mmol) according to the general procedure I. Purified by FCC using hexanes; Pale green oil (17 mg, 40%); R_f = 0.45 (hexanes/AcOEt 98:2; ^{1}H NMR (500 MHz, $CDCl_3$) δ: 7.26 – 7.24 (m, 1H, thiopheneH11), 7.03 – 6.93 (m, 4H, thiopheneH9/H10, ArH2/H6), 6.72 (tt, $^3J_{H-F}$ = 8.8 Hz, $^4J_{H-H}$ = 2.3 Hz, 1H, ArH4), 5.54 (d, *J* = 5.4 Hz,

1H, H7), 2.59 (d, *J* = 5.4 Hz, 1H, -S*H*); ^{13}C NMR (126 MHz, $CDCl_3$) *δ*: 163.16 (dd, $^{1}J_{C\text{-}F}$ = 250 Hz, $^{3}J_{C\text{-}F}$ = 12.5 Hz, ArC3/C5), 147.51 (t, $^{3}J_{C\text{-}F}$ = 8.4 Hz, ArC1), 146.42 (thiopheneC8), 127.04 (thiopheneC11), 126.07 (thiopheneC), 125.82 (thiopheneC), 110.76 (m, AXX'-system, ArC2/C6), 103.31 (t, $^{2}J_{C\text{-}F}$ = 25.4 Hz, ArC4), 42.67 (C7); Elemental analysis calcd. (%) for $C_{11}H_8F_2S_2$: C, 54.52; H, 3.33; found: C, 54.27; H, 3.61.

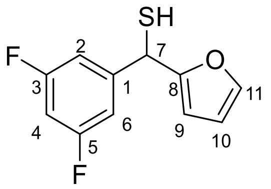

(3,5-difluorophenyl)(furan-2-yl)methanethiol (10b) was synthesized from **9b** (0.126 g, 0.47 mmol) according to the general procedure I. Purified by FCC using hexanes/AcOEt 100:0 to 98:2; Pale yellow oil (82 mg, 77%); R_f = 0.2 (hexanes/AcOEt 98:2; ^{1}H NMR (500 MHz, $CDCl_3$) *δ*: 7.39 (s, 1H, furaneH11), 6.96 – 6.92 (m, 2H, ArH2/H6), 6.74 – 6.70 (m, 1H, ArH4), 6.34 – 6.33 (brs, 1H, furaneH10), 6.26 – 6.6.25 (m, 1H, furanH9), 5.26 (d, *J* = 6.1 Hz, 1H, H7), 2.49 (d, *J* = 6.1 Hz, 1H, -S*H*); ^{13}C NMR (126 MHz, $CDCl_3$) *δ*: 163.16 (dd, $^{1}J_{C\text{-}F}$ = 249.1 Hz, $^{3}J_{C\text{-}F}$ = 12.5 Hz, ArC3/C5), 153.98 (furaneC8), 145.29 (t, $^{3}J_{C\text{-}F}$ = 8.9 Hz, ArC1), 142.90 (furaneC11), 110.91 (m, AXX'-system, ArC2/C6), 110.65 (furaneC10), 107.93 (furaneC9), 103.34 (t, $^{2}J_{C\text{-}F}$ = 25.4 Hz, ArC4), 44.49 (C7); Elemental analysis calcd. (%) for $C_{11}H_8F_2OS$: C, 58.40; H, 3.56; found: C, 58.23; H, 3.81.

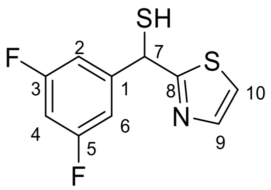

(3,5-difluorophenyl)(thiazol-2-yl)methanethiol (10d) was synthesized from **9d** (0.052 g, 0.18 mmol) according to the general procedure I. Purified by FCC using hexanes/AcOEt 10:0 to 9:1; Amber colored oil (13.2 mg, 30%); R_f = 0.35 (hexanes/AcOEt 8:2; ^{1}H NMR (500 MHz, $CDCl_3$) *δ*: 7.78 (brs, 1H, thiazoleH9), 7.34 (d, *J* = 3.2 Hz, 1H, thiazoleH10), 7.01 – 6.99 (m, 2H, ArH2/H6), 6.75 (tt, $^{3}J_{H\text{-}F}$ = 8.8 Hz, $^{4}J_{H\text{-}H}$ = 2.3 Hz, 1H, ArH4), 5.59 (d, *J* = 6 Hz, 1H, H7), 2.90 (d, *J* = 6 Hz, 3H, -S*H*); ^{13}C NMR (63 MHz, $CDCl_3$) *δ*: 172.05 (thiazoleC8), 163.57 (dd, $^{1}J_{C\text{-}F}$ = 250.2 Hz, $^{3}J_{C\text{-}F}$ = 12.7 Hz, ArC3/C5), 143.58 (broad, ArC1), 143.16 (thiazoleC9), 120.60 (thiazoleC10), 111.10 (m, AXX'-system, ArC2/C6), 103.85 (t, $^{2}J_{C\text{-}F}$ = 25.3 Hz, ArC4), 44.49 (s, C7); Elemental analysis calcd. (%) for $C_{10}H_7F_2NS_2$: C, 49.37; H, 2.90; N, 5.76; found: C, 49.09; H, 3.14; N, 5.91.

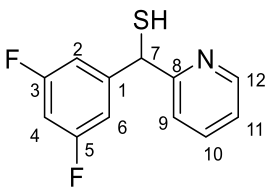

(3,5-difluorophenyl)(pyridin-2-yl)methanethiol (10f) was synthesized from **9f** (50 mg, 0.17 mmol) according to the general procedure I. Purified by FCC using hexanes/AcOEt 8:2; light yellow oil (17 mg, 41%); R_f = 0.25 (hexanes/AcOEt 8:2; ^{1}H NMR (500 MHz, $CDCl_3$) *δ*: 8.60 (d, *J* = 4.4 Hz, 1H, pyridineH12), 7.68 (dt, $^{3}J_{H\text{-}H}$ = 7.8 Hz, $^{4}J_{H\text{-}H}$ = 1.7 Hz, 1H, pyridineH10), 7.33 (d, *J* = 7.8 Hz, 1 H, pyridineH9), 7.22 (dd, J_1 = 7.8 Hz, J_2 = 4.9 Hz, 1H, pyridineH11), 7.04 – 6.96 (m, 2H, ArH2/H6), 6.68 (tt, $^{3}J_{H\text{-}F}$ = 8.8 Hz, $^{4}J_{H\text{-}H}$ = 2.2

Hz, 1H, ArH4), 5.39 (s, 1H, H*7*), 2.79 (brs, 1H, -S*H*); ^{13}C NMR (126 MHz, $CDCl_3$) *δ:* 163.11 (dd, $^{1}J_{C\text{-}F}$ = 247.23 Hz, $^{3}J_{C\text{-}F}$ = 12.6 Hz, ArC3/C5), 160.44 (pyridineC8), 149.42 (pyridineC12), 146.51 (t, $^{3}J_{C\text{-}F}$ = 8.7 Hz, ArC1), 137.51 (pyridineC10), 122.77 (pyridineC), 122.36 (pyridineC), 111.20 (m, AXX'-system, ArC2/C6), 103.08 (t, $^{2}J_{C\text{-}F}$ = 25.2 Hz, ArC4), 48.65 (C7); Elemental analysis calcd. (%) for $C_{12}H_9F_2NS$: C, 60.75; H, 3.82; N, 5.90; found: C, 60.49; H, 4.07; N, 6.11.

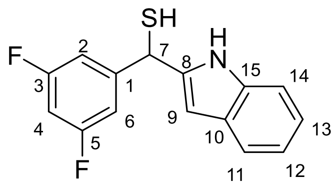

(3,5-difluorophenyl)(1*H*-indol-2-yl)methanethiol (10i) was synthesized from **9i** (71 mg, 0.19 mmol) according to the general procedure **I**. Purified by FCC using hexanes-AcOEt 8:2 to 7:3; light yellow oil (21 mg, 41%); R_f = 0.5 (hexanes/AcOEt 6:4); ^{1}H NMR (500 MHz, $CDCl_3$) *δ*: 8.21 (s, 1H, indoleN*H*), 7. 58 (d, *J* = 7.7 Hz, 1H, indoleH), 7.35 (d, *J* = 8.1 Hz, 1H, indoleH), 7.25 – 7.20 (m, 1 H, indoleH), 7.17 – 7.12 (m, 1H, indoleH), 7.03 – 6.95 (m, 2H, ArH2/H6), 6.77 (tt, $^{3}J_{H\text{-}F}$ = 8.7 Hz, $^{4}J_{H\text{-}H}$ = 2.2 Hz, 1H, ArH4), 6.37 (s, 1H, indoleH9), 5,46 (d, *J* = 6 Hz, 1H, H7), 2.47 (d, *J* = 6 Hz, 1H, -S*H*); ^{13}C NMR (126 MHz, $CDCl_3$) *δ*: 163.23 (dd, $^{1}J_{C\text{-}F}$ = 249.6 Hz, $^{3}J_{C\text{-}F}$ = 12.8 Hz, ArC3/C5), 145.82 (t, $^{3}J_{C\text{-}F}$ = 8.5 Hz, ArC1), 138.06 (indoleC), 136.55 (indoleC), 128.22 (indoleC), 122.74 (indoleC), 120.87 (indoleC), 120.49 (indoleC), 111.09 (indoleC), 110.99 (m, AXX'-system, partially overlapping with indoleC, ArC2/C6), 103.50 (t, $^{2}J_{C\text{-}F}$ = 25.5 Hz, ArC4), 102.32 (indoleC9), 41.15 (C7); Elemental analysis calcd. (%) for $C_{15}H_{11}F_2NS$: C, 65.44; H, 4.03; N, 5.09; found: C, 65.28; H, 4.19; N, 4.91.

General Procedure for the synthesis of amides 18a–18i (J)

To a solution of the corresponding carboxylic acid (1.2 eq) in DMF (0.2–0.3 M) was added TBTU (1.2 eq) followed by dropwise addition of DIPEA (2.4 eq) to a solution of **17**[2] (1 eq). The reaction mixture was stirred overnight at room temperature. The reaction was poured into ice-cold saturated $NaHCO_3$ solution, and the precipitate was filtered, washed with cold water, and dried.

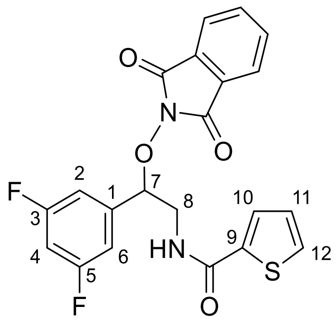

***N*-(2-(3,5-difluorophenyl)-2-((1,3-dioxoisoindolin-2-yl)oxy)ethyl)thiophene-2-carboxamide (18a)** was synthesized from **17** (0.35 g, 1.0 mmol) according to the general procedure J. Purified by FCC using hexanes/AcOEt 8:2 gradually to 7:3 to afford a pink salmon-colored solid (0.42 g, 99%); R_f = 0.5 (hexanes/AcOEt 6:4); ^{1}H NMR (500 MHz, $CDCl_3$) *δ*: 7.85 – 7.83 (m, 2H, N*Phth*), 7.79 – 7.77 (m, 2H, N*Phth*), 7.75 (d, $^{3}J_{H\text{-}H}$ = 3.9 Hz, 1H, thiopheneH10), 7.62 (t, $^{3}J_{H\text{-}H}$ = 5.6 Hz, 1H, N*H*), 7.52 (d, $^{3}J_{H\text{-}H}$ = 5.1 Hz, 1H, thiopheneH12), 7.14 (t, $^{3}J_{H\text{-}H}$ = 4.4 Hz, 1H, thiopheneH11), 7.11 (d, $^{3}J_{H\text{-}F}$ = 6.3 Hz, 2H, ArH2/H6), 6.82 (tt, $^{3}J_{H\text{-}F}$ = 8.8, $^{4}J_{H\text{-}H}$ = 2.3 Hz, 1H, Ar*H4*), 5.23 (dd, $^{3}J_{H\text{-}H}$ = 8.3, $^{4}J_{H\text{-}H}$ = 3.2 Hz, 1H, H7), 4.01 (ddd, $^{2}J_{H\text{-}H}$ = 14.9,

$^{3}J_{H-H}$ = 6.6, $^{3}J_{H-H}$ = 2.9 Hz, 1H, H8a), 3.83 (ddd, $^{2}J_{H-H}$ = 15.2, $^{3}J_{H-H}$ = 8.3, $^{3}J_{H-H}$ = 4.5 Hz, 1H, H8b); Elemental analysis calcd. (%) for $C_{21}H_{14}F_2N_2O_4S$: C, 58.88; H, 3.29; N, 6.54; found: C, 59.04; H, 3.52; N, 6.76.

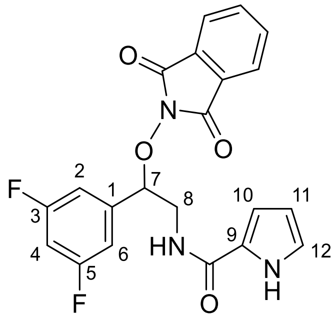

***N*-(2-(3,5-difluorophenyl)-2-((1,3-dioxoisoindolin-2-yl)oxy)ethyl)-1*H*-pyrrole-2-carboxamide (18c)** was synthesized from **17** (0.35 g, 1.0 mmol) according to the general procedure J. Purified by FCC using hexanes/AcOEt 7:3 gradually to 6:4 and 5:5 to afford a white solid (0.1 g, 43%); R_f = 0.4 (hexanes/AcOEt 6:4); ^{1}H NMR (500 MHz, $CDCl_3$) δ: 9.32 (s, 1H, pyrroleN*H*)), 7.85 – 7.83 (m, 2H, N*Phth*), 7.79 – 7.76 (m, 2H, N*Phth*), 7.46 (brs, t, $^{3}J_{H-H}$ = 5.6 Hz, 1H, N*H*), 7.11 (m, ArH2/H6), 6.95 – 6.91 (m, 2H, pyrroleH10/H12), 6.92 – 6.91 (m, 1H, pyrroleH10), 6.81 (tt, $^{3}J_{H-F}$ = 8.8, $^{4}J_{H-H}$ = 2.4 Hz, 1H, ArH4), 6.30 (dd, $^{3}J_{H-H}$ = 6.1 Hz, $^{3}J_{H-H}$ = 2.7 Hz, 1H, pyrroleH11), 5.21 (dd, $^{3}J_{H-H}$ = 8.2, $^{3}J_{H-H}$ = 3.1 Hz, 1H, H7), 3.99 (ddd, $^{2}J_{H-H}$ = 15.2, $^{3}J_{H-H}$ = 6.7, $^{3}J_{H-H}$ = 3.1 Hz, 1H, H8a), 3.82 (ddd, $^{2}J_{H-H}$ = 14.9, $^{3}J_{H-H}$ = 8.2, $^{3}J_{H-H}$ = 4.5 Hz, 1H, H8b); Elemental analysis calcd. (%) for $C_{21}H_{15}F_2N_3O_4$: C, 61.32; H, 3.68; N, 10.22; found: C, 61.17; H, 3.91; N, 10.36.

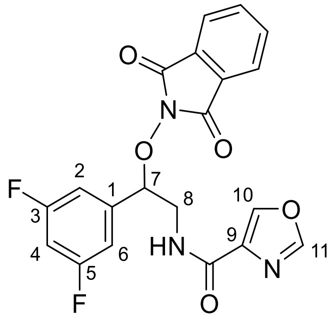

***N*-(2-(3,5-difluorophenyl)-2-((1,3-dioxoisoindolin-2-yl)oxy)ethyl)oxazole-4-carboxamide (18d)** was synthesized from **17** (0.15 g, 0.42 mmol) according to the general procedure J to afford a pale yellow solid (0.16 g, 92%); R_f = 0.4 (hexanes/AcOEt 5:5); ^{1}H NMR (500 MHz, $CDCl_3$) δ: 8.24 (s, 1H, oxazoleH11), 7.98 (brs 1H, N*H*), 7.92 (s, 1H, oxazoleH10), 7.82 – 7.80 (m, 2H, N*Phth*), 7.75 – 7.74 (m, 2H, N*Phth*), 7.12 (d, $^{3}J_{H-F}$ = 6.3 Hz, 2H, ArH2/H6), 6.79 (tt, $^{3}J_{H-F}$ = 8.8, $^{4}J_{H-H}$ = 2.3 Hz, 1H, ArH4), 5.35 (dd, $^{3}J_{H-H}$ = 7.5, $^{4}J_{H-H}$ = 3.5 Hz, 1H, H7), 3.96 (ddd, $^{2}J_{H-H}$ = 14.9, $^{3}J_{H-H}$ = 6.5, $^{3}J_{H-H}$ = 3.5 Hz, 1H, H8a), 3.89 (ddd, $^{2}J_{H-H}$ = 14.9, $^{3}J_{H-H}$ = 7.5, $^{3}J_{H-H}$ = 5.7 Hz, 1H, H8b); Elemental analysis calcd. (%) for $C_{20}H_{13}F_2N_3O_5$: C, 58.12; H, 3.17; N, 10.17; found: C, 57.96; H, 3.45; N, 9.92.

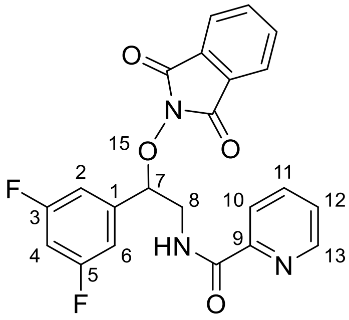

***N*-(2-(3,5-difluorophenyl)-2-((1,3-dioxoisoindolin-2-yl)oxy)ethyl)picolinamide (18e)** was synthesized from **17** (0.35 g, 0.99 mmol) according to the general procedure J. Purified by FCC using hexanes/AcOEt 7:3 gradually to 6:4 to afford a salmon-colored solid (0.18 g, 47%); R_f = 0.4 (hexanes/AcOEt 6:4); ^{1}H NMR (500 MHz, $CDCl_3$) δ: 8.86 (brs, 1H, N*H*), 8.62 (d, J = 4.6 Hz, 1H, pyridineH13), 8.19 (d, J = 7.6 Hz, 1H, pyridineH10), 7.87 (t, J = 7.7 Hz, 1H, pyridineH11), 7.81 – 7.78 (m, 2H, N*Phth*), 7.74 – 7.73 (m, 2H, N*Phth*), 7.57 – 7.41 (m, 2H, pyridineH12), 7.15 (d, $^3J_{H\text{-}F}$ = 6.3 Hz, 2H, ArH2/H6), 6.79 (tt, $^3J_{H\text{-}F}$ = 8.8, $^4J_{H\text{-}H}$ = 2.3 Hz, 1H, ArH4), 5.45 (dd, $^3J_{H\text{-}H}$ = 7.3, $^4J_{H\text{-}H}$ = 3.9 Hz, 1H, H7), 4.07 – 3.92 (m, 2H, H8); Elemental analysis calcd. (%) for $C_{22}H_{15}F_2N_3O_4$: C, 62.41; H, 3.57; N, 9.93; found: C, 62.27; H, 3.82; N, 10.14.

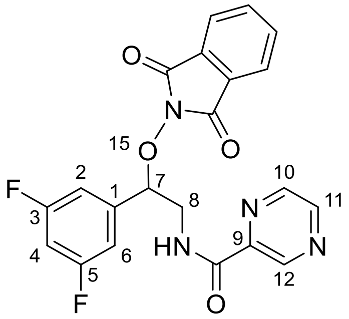

***N*-(2-(3,5-difluorophenyl)-2-((1,3-dioxoisoindolin-2-yl)oxy)ethyl)pyrazine-2-carboxamide (18g)** was synthesized from **17** (0.35 g, 0.99 mmol) according to the general procedure J. The resulting white precipitate was dissolved in $CHCl_3$ and concentrated under reduced pressure. The residue was dried under high vacuum to afford a salmon-colored solid (0.4 g, 95%); R_f = 0.5 (hexanes/AcOEt 5:5); ^{1}H NMR (500 MHz, $CDCl_3$) δ: 9.40 (brs, 1H, pyrazineH12), 8.78 (d, J = 2.7 Hz, 1H, pyrazineH11), 8.71 (brs, 1H, N*H*), 8.62 (brs, 1H, pyrazineH10), 7.81 – 7.79 (m, 2H, N*Phth*), 7.77 – 7.73 (m, 2H, N*Phth*), 7.14 (d, $^3J_{H\text{-}F}$ = 5.9 Hz, 2H, ArH2/H6), 6.80 (tt, $^3J_{H\text{-}F}$ = 9.0, $^4J_{H\text{-}H}$ = 2.4 Hz, 1H, ArH4), 5.42 (dd, $^3J_{H\text{-}H}$ = 7.5, $^4J_{H\text{-}H}$ = 3.5 Hz, 1H, H7), 4.03 (ddd, $^2J_{H\text{-}H}$ = 14.9, $^3J_{H\text{-}H}$ = 6.6, $^3J_{H\text{-}H}$ = 3.4 Hz, 1H, H8a), 3.95 (ddd, $^2J_{H\text{-}H}$ =14.9, $^3J_{H\text{-}H}$ = 7.2, $^3J_{H\text{-}H}$ = 5.7 Hz, 1H, H8b); Elemental analysis calcd. (%) for $C_{21}H_{14}F_2N_4O_4$: C, 59.44; H, 3.33; N, 13.20; found: C, 59.68; H, 3.51; N, 13.37.

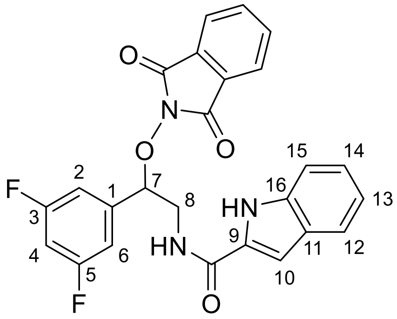

***N*-(2-(3,5-difluorophenyl)-2-((1,3-dioxoisoindolin-2-yl)oxy)ethyl)-1*H*-indole-2-carboxamide (18i)** was synthesized from **17** (0.3 g, 0.85 mmol) according to the general procedure J. The resulting yellow precipitate was recrystallized from $CHCl_3$ to afford a yellow solid (0.29 g, 73%); R_f = 0.3 (hexanes/AcOEt 7:3); ^{1}H NMR (500 MHz, DMSO-d$_6$) *δ*: 11.59 (s, 1H, indoleN*H*), 8.72 (t, $^3J_{H-H}$ = 6.0 Hz, 1H, N*H*), 7.84 (brs, 4H, N*Phth*), 7.62 (d, $^3J_{H-H}$ = 7.8 Hz, 1H, indoleH12), 7.42 (d, $^3J_{H-H}$ = 8.3 Hz, 1H, indoleH), 7.31 – 7.30 (m, 2H, ArH2/H6*)* 7.23 (t, $^3J_{H-F}$ = 9.1 Hz, 1H, ArH4), 7.18 (t, *J* = 7.7 Hz, 1H, indoleH), 7.11 (s, 1H, indoleH10), 7.04 (t, *J* = 7.5 Hz, 1H, indoleH), 5.48 (t, $^3J_{H-H}$ = 6.2 Hz, 1H, H7), 3.93 (ddd, $^2J_{H-H}$ = 14.3, $^3J_{H-H}$ = 6.5, $^3J_{H-H}$ = 3.2 Hz, 1H, H8a), 3.83 (ddd, $^2J_{H-H}$ = 14.3, $^3J_{H-H}$ = 7.6, $^3J_{H-H}$ = 5.3 Hz, 1H, H8b)

General procedure for the synthesis of hydroxylamines 19a–19i (K)

The crude amide was treated with the appropriate reagent under the following conditions: aqueous methylamine (40%, 10 eq, 0.075 M in EtOH/THF, 2:1) or aqueous hydrazine hydrate (55%, 2 eq, 0.2 M in THF). The reaction mixture was stirred at room temperature until completion as monitored by TLC. The volatiles were removed under reduced pressure, and the residue was purified by flash column chromatography using hexanes/AcOEt 1:1 containing 1% Et_3N to afford the desired aminooxy carboxamide.

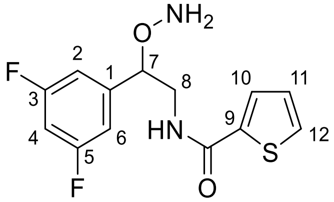

***N*-(2-(aminooxy)-2-(3,5-difluorophenyl)ethyl)thiophene-2-carboxamide hydrochloride (19a)** was synthesized from **18a** (0.37 g, 0.86 mmol) according to the general procedure K using aqueous methylamine 40%; white oil (0.25 g, 96%); R_f = 0.4 (free-base hexanes/AcOEt 6:4); ^{1}H NMR (DMSO-d$_6$, 500 MHz) *δ*: 8.59 (t, *J* = 6.0 Hz, 1H, N*H*), 7.74 (d, *J* = 4.6 Hz, 2H, thiopheneH10/12), 7.15 – 7.11 (m, 2H, ArH4, thiopheneH11), 7.02 – 7.11 (m, 2H, ArH2/H6), 6.20 (s, 1H, O-N*H*$_2$), 4.68 (t, $^3J_{H-H}$ = 5.8 Hz, 1H, H7), 3.57 – 3.30 (m, 2H, H8); ^{13}C NMR (DMSO-d$_6$, 126 MHz) *δ*: 162.45 (dd, $^1J_{C-F}$ = 246.18 Hz, $^3J_{C-F}$ =14.31 Hz, 2C, ArC3/C5), 161.59 (C=O), 141.10 (t, $^3J_{C-F}$ = 8.92 Hz, ArC1), 139.24 (thiopheneC9), 131.08 (thiopheneC12), 128.58 (thiopheneC11), 127.92 (thiopheneC10) 110.59 (m, AXX'-system, ArC2/C6), 104.22 (t, $^2J_{C-F}$ =25.83 Hz, ArC4), 82.98 (C7), 42.82 (C8); Elemental analysis calcd. (%) for $C_{13}H_{12}F_2N_2O_2S$: C, 52.34; H, 4.05; N, 9.39; found: C, 52.61; H, 4.21; N, 9.47.

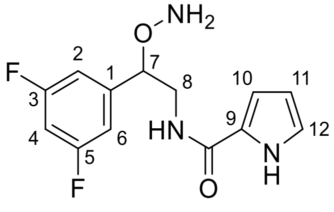

***N*-(2-(aminooxy)-2-(3,5-difluorophenyl)ethyl)-1*H*-pyrrole-2-carboxamide (19c)** was synthesized from **18c** (50 mg, 0.12 mmol) according to the general procedure J using aqueous hydrazine hydrate 55%; slightly yellow oil (27 mg, 82%); R_f = 0.35 (hexanes/AcOEt 5:5); ^{1}H NMR (250MHz, DMSO-d$_6$) *δ*: 11.43 (s, 1H, pyrroleN*H*), 8.05 (t, 1H, $^3J_{H-H}$ = 5.54 Hz, N*H*), 7.12 (tt, $^3J_{H-F}$ = 9.27 Hz, $^4J_{H-H}$ = 4.73 Hz, 1H, ArH4), 7.05-6.92(m, 2H, ArH2/H6), 6.83 (brs, 1H, pyrroleH12), 6.75 (brs, 1H, pyrroleH10), 6.18 (s, 2H, O-N*H*$_2$), 6.06 (dd, $^3J_{H-H}$ = 6.7 Hz, $^3J_{H-H}$ = 2.4 Hz, 1H, pyrroleH11), 4.64 (t, $^3J_{H-H}$ = 5.7 Hz, 1H, H7), 3.54-3.40(m, 2H overlap with DMSO-d$_6$, H8); ^{13}C NMR (63MHz, DMSO-d$_6$) *δ*: 170.37 (C=O), 162.50 (dd, $^1J_{C-F}$ = 247 Hz, $^3J_{C-F}$ = 13.5

Hz, ArC3/C5), 145.83 (t, $^{3}J_{C\text{-}F}$ = 8.53 Hz, ArC1), 126.08 (pyrroleC9), 121.35 (pyrroleC12), 110.10 (pyrroleC10), 109.82 (m, AXX'-system, ArC2/C6), 108.53 (pyrroleC11), 102.67 (t, $^{2}J_{C\text{-}F}$ =25.93 Hz, ArC4), 83.44 (C7), 42.75 (C8); Elemental analysis calcd. (%) for $C_{13}H_{13}F_2N_3O_2$: C, 55.51; H, 4.66; N, 14.94; found: C, 55.27; H, 4.81; N, 14.73.

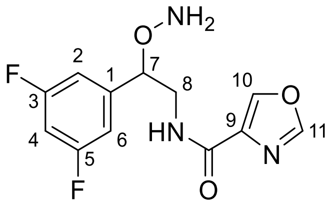

***N*-(2-(aminooxy)-2-(3,5-difluorophenyl)ethyl)oxazole-4-carboxamide (19e)** was synthesized from **18e** (90 mg, 0.22 mmol) according to the general procedure J using aqueous hydrazine hydrate 55%; off-yellow solid (33 mg, 46%); R_f = 0.3 (hexanes/AcOEt 5:5); ^{1}H NMR (250MHz, DMSO-d$_6$,) δ: 8.61 (s, 1H, oxazoleH11), 8.51 (s, 1H, oxazoleH10), 8.20 (t, $^{3}J_{H\text{-}H}$ = 5.78 Hz, N*H*), 7.15 – 6.99 (m, 3H, ArH2/H4/H6), 6.21 (s, 2H, O-N*H$_2$*), 4.70 (t, $^{3}J_{H\text{-}H}$ = 5.7 Hz, 1H, H7), 3.49 (dd, $^{3}J_{H\text{-}H}$ = $^{3}J_{H\text{-}H}$ 5.68 Hz, 2H, H8); ^{13}C NMR (63MHz, DMSO-d$_6$) δ: 162.24 (dd, $^{1}J_{C\text{-}F}$ =245.3 Hz, $^{3}J_{C\text{-}F}$ =12.72 Hz, ArC3/C5), 159.81(C=O), 152.28 (oxazoleC11), 145.39 (ArC1), 141.88 (oxazoleC10), 135.38 (oxazoleC9), 109.83 (m, AXX'-system, ArC2/C6), 102.73 (t, $^{2}J_{C\text{-}F}$ = 25.96 Hz, ArC1), 82.71 (C7), 42.40 (C8); Elemental analysis calcd. (%) for $C_{12}H_{11}F_2N_3O_3$: C, 50.89; H, 3.91; N, 14.84; found: C, 51.16; H, 4.09; N, 15.13.

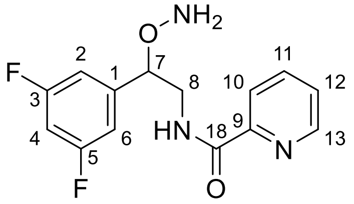

***N*-(2-(aminooxy)-2-(3,5-difluorophenyl)ethyl)picolinamide (19f)** was synthesized from **18f** (70 mg, 0.17 mmol) according to the general procedure J using aqueous methylamine 40%; colorless oil (45 mg, 93%); R_f = 0.3 (hexanes/AcOEt 5:5); ^{1}H NMR (250 MHz, DMSO-d$_6$) δ: 8.74 (t, $^{3}J_{H\text{-}H}$ = 6.0 Hz, 1H, N*H*), 8.65 (dt, $^{3}J_{H\text{-}H}$ = 4.7 Hz, $^{4}J_{H\text{-}H}$ = 1.2 Hz, 1H, pyridineH13), 8.01 – 7.98 (m, 2H, pyridineH10/H11), 7.66 – 7.56 (m, 1H, pyridineH12), 7.12 (tt, $^{3}J_{H\text{-}F}$ = 9.45, $^{4}J_{H\text{-}H}$ =2.38 Hz, 1H, ArH4), 7.04 (dd, $^{3}J_{H\text{-}F}$ = 8.5, $^{4}J_{H\text{-}H}$ =1.9 Hz, 2H, ArH2/H6), 6.25 (s, 2H, O-N*H$_2$*), 4.75 (t, $^{3}J_{H\text{-}H}$ = 5.8 Hz, 1H, H7), 3.58 (dd, $^{3}J_{H\text{-}H}$ =$^{3}J_{H\text{-}H}$ = 5.9 Hz, 2H, H8); ^{13}C NMR (63 MHz, DMSO-d$_6$) δ: 163.70 (C=O), 162.24 (dd, $^{1}J_{C\text{-}F}$ = 245.8, $^{3}J_{C\text{-}F}$ = 13.0 Hz, ArC3/C5), 149.60 (pyridineC9), 148.47 (pyridineC13), 145.44 (t, $^{3}J_{C\text{-}F}$ = 8.6 Hz, ArC4), 137.90 (pyridineC11), 126.65 (pyridineC12), 121.86 (pyridineC10), 109.82 (m, AXX'-system, ArC2/C6), 102.75 (t, $^{2}J_{C\text{-}F}$ =25.9 Hz, ArC4), 82.81 (C7), 42.93 (C8); Elemental analysis calcd. (%) for $C_{14}H_{13}F_2N_3O_2$: C, 57.34; H, 4.47; N, 14.33; found: C, 57.41; H, 4.62; N, 14.42.

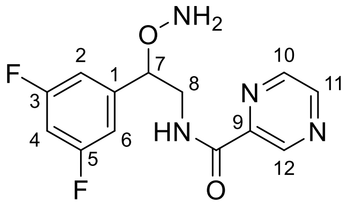

***N*-(2-(aminooxy)-2-(3,5-difluorophenyl)ethyl)pyrazine-2-carboxamide (19g)** was synthesized from **18g** (50.0 mg, 0.12 mmol) according to the general procedure J using aqueous methylamine 40%; white solid

(33 mg, 95%); R_f = 0.2 (hexanes/AcOEt 5:5); ^{1}H NMR (250MHz, DMSO-d$_6$) *δ:* 9.15 (s, 1H, pyrazineH12), 8.87 (s, 2H, N*H* and pyrazineH11), 8.73 (s, 1H, pyrazineH10), 7.19 – 6.96 (m, 3H, ArH2/H4/H6), 6.23 (s, 2H, O-N*H$_2$*), 4.75 (t, $^3J_{H-H}$ = 6 Hz, 1H, H7), 3.58 (t, $^3J_{H-H}$ = 6 Hz, 2H, H8);^{13}C NMR (63MHz, DMSO-d$_6$) *δ*: 162.82 (C=O), 162.26 (dd, $^1J_{C-F}$ = 246 Hz, $^3J_{C-F}$ =12.91 Hz, ArC3/C5), 147.68 (pyrazineC11), 145.35 (t, $^3J_{C-F}$ = 8.63 Hz, ArC1), 144.46 (pyrazineC9), 143.44 (d, *J* = 5.09 Hz, pyrazineC10/12), 109.84 (m, AXX'-system, ArC2/C6), 102.79 (t, $^2J_{C-F}$ =25.83 Hz, ArC4), 82.7 (C7), 42.97 (C8); Elemental analysis calcd. (%) for $C_{13}H_{12}F_2N_4O_2$: C, 53.06; H, 4.11; N, 19.04; found: C, 53.24; H, 4.32; N, 19.32.

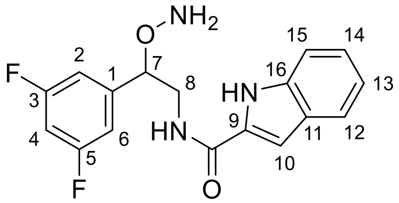

***N*-(2-(aminooxy)-2-(3,5-difluorophenyl)ethyl)-1*H*-indole-2-carboxamide (19i)** was synthesized from **18i** (99 mg, 0.22 mmol) according to the general procedure J using aqueous hydrazine hydrate 55%; salmon-pink solid (41 mg, 58%); R_f = 0.4 (hexanes/AcOEt 6:4); ^{1}H NMR (250MHz, DMSO-d$_6$) *δ*: 11.57 (s, 1H, indoleN*H*), 8.55 (t, $^3J_{H-H}$ = 5.8 Hz, 1H, N*H*), 7.60 (d, $^3J_{H-H}$ = 7.9 Hz, 1H, indoleH12), 7.41 (d, $^3J_{H-H}$ = 8.2 Hz, 1H, indole*H15*), 7.23 – 6.97 (m, 6H, indoleH10/H13/H14, ArH1/H3/H5), 6.22 (s, 2H, O-N*H$_2$*), 4.71 (t, $^3J_{H-H}$ = 5.8 Hz, 1H, H7), 3.54 (dd, $^3J_{H-H}$ =$^3J_{H-H}$ = 5.8, 2H, H8); ^{13}C NMR (63MHz, DMSO-d$_6$) *δ*: 162.28 (dd, $^1J_{C-F}$ = 245.8 Hz, $^3J_{C-F}$ = 13.0 Hz, ArC3/C5), 161.16 (C=O), 145.67 (t, $^3J_{C-F}$ = 8.5 Hz, ArC1), 136.42 (indoleC), 131.51 (indoleC), 127.05 (indoleC), 123.31 (indoleC), 121.51 (indole*C12*), 119.72 (indoleC), 112.30 (indole*C15*), 109.86 (m, AXX'-system, ArC2/C6), 102.74 (t, $^2J_{C-F}$ = 25.8 Hz, ArC4), 102.64 (indoleC), 83.24 (C7), 42.97 (C8); Elemental analysis calcd. (%) for C17H15F2N3O2: C, 61.63; H, 4.56; N, 12.68; found: C, 61.49; H, 4.71; N, 12.53

General procedure for the synthesis of ketoamines 21a, 21b, 21d and 21i (L)

EDC hydrochloride (1.2 eq) was added to a mixture of **20** [2] (1 eq), DIPEA (1 eq) and the corresponding heteroaromatic acid (1.2 eq) at 0 °C in DCM (0.3 M). The reaction was then allowed to reach room temperature and stirred for several hours (2 – 6 h) until completion as confirmed by TLC. Then, the solvent was evaporated under reduced pressure and the crude mixture was purified with FCC using the appropriate ratio of hexanes/AcOEt.

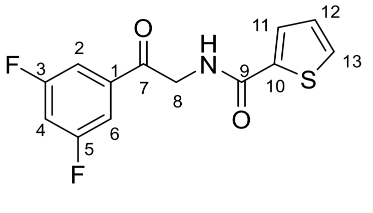

***N*-(2-(3,5-difluorophenyl)-2-oxoethyl)thiophene-2-carboxamide (21a)** was synthesized from **20** (0.5 g, 2.4 mmol) according to the general procedure L. Purified by FCC using hexanes/AcOEt 9:1 to 7:3; white solid (505 mg, 74%); R_f = 0.2 (hexanes/AcOEt 7:3); ^{1}H NMR (500 MHz, DMSO-*d$_6$*) *δ*: 8.93 (t, J_{NH-H8a} = J_{NH-H8b} = 5.5 Hz, 1H, -N*H*), 7.85 – 7.82 (m, 1H, thiopheneH13), 7.80 – 7.78 (m, 1H, thiopheneH11), 7.77 – 7.70 (m, 2H, ArH2/H6), 7.63 (tt, $^3J_{H-F}$ = 8.9 Hz, $^4J_{H-H}$ = 2.1 Hz, 1H, ArH4), 7.20 – 7.17 (m, 1H, thiopheneH12), 4.76 (d, *J* = 5.5 Hz, 2H, H8); ^{13}C NMR (126 MHz, $CDCl_3$) *δ*: 193.46 (C7), 163.10 (dd, $^1J_{C-F}$ = 248.6 Hz, $^3J_{C-F}$ = 12.5 Hz, C3/C5), 161.48 (C9), 139.11 (thiopheneC10), 138.05 (t, $^3J_{C-F}$ = 7.3 Hz, ArC1), 131.03 (thiophenC13), 128.55 (thiopheneC11), 127.94 (thiopheneC12), 111.19 (m, AXX′ system, ArC2/C6), 108.89

(t, $^{2}J_{C\text{-}F}$ = 25.9 Hz, ArC4), 46.52 (C8); Elemental analysis calcd. (%) for $C_{13}H_{9}F_{2}NO_{2}S$: C, 55.51; H, 3.23; N, 4.98 found: C 55.76; H, 3.49; N, 5.13.

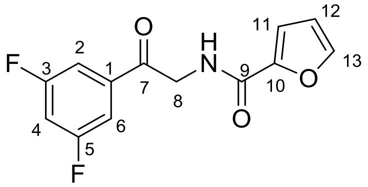

***N*-(2-(3,5-difluorophenyl)-2-oxoethyl)furan-2-carboxamide (21b)** was synthesized from **20** (0.1 g, 0.48 mmol) according to the general procedure L. Purified by FCC using hexanes/AcOEt 8:2 to 4:6; white solid 123 mg, 96%); R_f = 0.3 (hexanes/AcOEt 7:3); ^{1}H NMR (250 MHz, $CDCl_3$) δ: 7.56 – 7.50 (m, 3H, ArH2/H6 and furaneH), 7.29 (brs, -N*H*), 7.18 (d, J = 3.6 Hz, 1H, furaneH), 7.10 (tt, $^{3}J_{H\text{-}F}$ = 8.5 Hz, $^{4}J_{H\text{-}H}$ = 2.2 Hz, 1H, ArH4), 6.53 (dd, J_1 = 3.6 Hz, J_2 = 1.8 Hz, 1H, furaneH), 4.88 (d, J_1 = 4.6 Hz, 2H, H8); ^{13}C NMR (63 MHz, $CDCl_3$) δ: 191.3 (t, $^{4}J_{C\text{-}F}$ = 2.4 Hz, C7), 163.39 (dd, $^{1}J_{C\text{-}F}$ = 252.2 Hz, $^{3}J_{C\text{-}F}$ = 11.8 Hz, ArC3/C5), 158.47 (C9) 147.54 (furaneC10), 144.59 (furaneC13), 137.23 (t, $^{3}J_{C\text{-}F}$ = 7.8 Hz, ArC1), 115.04 (furaneC11), 112.37 (furaneC12), 111.22 (m, AXX′-system, ArC2/C6), 109.73 (t, $^{2}J_{C\text{-}F}$ = 25.2 Hz, ArC4), 46.40 (C8); Elemental analysis calcd. (%) for $C_{13}H_{9}F_{2}NO_{3}$: C, 58.87; H, 3.42; N, 5.28 found: C, 59.11; H, 3.69; N, 5.13.

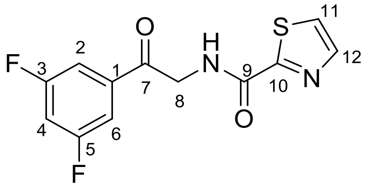

***N*-(2-(3,5-difluorophenyl)-2-oxoethyl)thiazole-2-carboxamide (21d)** was synthesized from **20** (0.27 g, 1.3 mmol) according to the general procedure L. Purified by FCC using hexanes/AcOEt 9:1 to 8:2; pale yellow solid (329 mg, 90%); R_f = 0.4 (hexanes/AcOEt 7:3); ^{1}H NMR (500 MHz, $CDCl_3$) δ: 8.13 (brs, -N*H*), 7.94 (d, J = 3 Hz, 1H, thiazoleH12), 7.62 (d, J = 3 Hz, 1H, thiazoleH11), 7.57 – 7.52 (m, 2H, ArH2/H6), 7.10 (tt, $^{3}J_{H\text{-}F}$ = 8.3 Hz, $^{4}J_{H\text{-}H}$ = 2.4 Hz, 1H, ArH4), 4.91 (d, J = 4.9 Hz, 2H, H8); Elemental analysis calcd. (%) for $C_{12}H_{8}F_{2}N_{2}O_{2}S$: C, 51.06; H, 2.86; N, 9.92 found: C, 51.29; H, 3.11; N, 10.15.

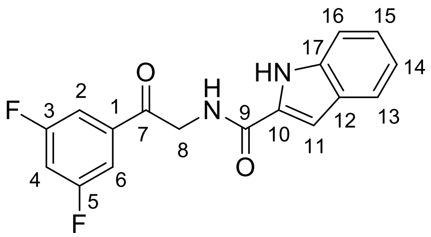

***N*-(2-(3,5-difluorophenyl)-2-oxoethyl)-1*H*-indole-2-carboxamide (21i)** was synthesized from **20** (0.5 g, 2.4 mmol) according to the general procedure L. Purified by recrystallization from ethyl acetate; yellow solid (117 mg, 15%); R_f = 0.4 (hexanes/AcOEt 7:3); *Due to its limited solubility, the compound could be purified only by recrystallization, and acquisition of a ^{13}C NMR spectrum was not feasible. Purity was assessed by ^{1}H NMR only, and the compound was used without further purification*. ^{1}H NMR (500 MHz, DMSO-d$_6$) δ: 11.62 (s, 1H, indoleN*H*), 8.92 (t, $J_{NH\text{-}H8a}$ = $J_{NH\text{-}H8b}$ = 5.5 Hz, 1H, -N*H*), 7.79 – 7.74 (m, 2H, indoleH), 7.67 – 7.61 (m, 2H, ArH2/H6), 7.43 (d, J = 8.3 Hz, 1H, indoleH),7.25 – 7.16 (m, 2H, indoleH), 7.05 (tt, $^{3}J_{H\text{-}F}$ = 7.6 Hz, $^{4}J_{H\text{-}H}$ = 2.4 Hz, 1H, ArH4), 4.81 (d, J = 4.6 Hz, 2H, H8); Elemental analysis calcd. (%) for $C_{17}H_{12}F_{2}N_{2}O_{2}$: C, 64.97; H, 3.85; N, 8.91 found: C, 64.73; H, 4.06; N, 8.69.

General procedure for the synthesis of alcohols 22a, 22b, 22d and 22i (M)

$NaBH_4$ (1.2 eq) was added to an ice-cold solution of the corresponding keto compound (1 eq) in abs. EtOH (0.05M) and stirring was continued at 0 °C for up to 2 hours until reaction was judged complete by TLC. The reaction was then quenched with water and was allowed to reach room temperature and stirred for 30 min. The volatiles were removed under reduced pressure and the crude residue was dissolved in AcOEt and washed with water (3x) and brine (1x), dried over $MgSO_4$, filtered and evaporated to dryness. The crude alcohol was sufficiently pure by NMR and was used for the next step without further purification.

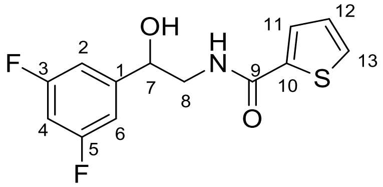

***N*-(2-(3,5-difluorophenyl)-2-hydroxyethyl)thiophene-2-carboxamide (22a)** was synthesized from **21a** (0.505 g, 1.79 mmol) according to the general procedure I; This product was additionally purified by FCC using hexanes/AcOEt 7:3; white solid (369 mg, 73%); R_f = 0.2 (hexanes/AcOEt 7:3); ^{1}H NMR (500 MHz, $CDCl_3$) δ: 7.55 – 7.48 (m, 2H, thiopheneH11/H13), 7.09 (dd, J_1 = 5 Hz, J_2 = 3.9 Hz, 1H, thiopheneH12), 6.99 – 6.91 (m, 2H, ArH2/H6), 6.73 (tt, $^3J_{H\text{-}F}$ = 8.8 Hz, $^4J_{H\text{-}H}$ = 2.3 Hz, 1H, ArH4), 6.43 (brs, 1H, -N*H*), 4.95 (dd, $^3J_{H7\text{-}H8a}$ = 7.8 Hz, $^3J_{H7\text{-}H8b}$ = 2.7 Hz, 1H, H7), 3.87 (ddd, $^2J_{H8b\text{-}H8a}$ = 14.3 Hz, $^3J_{H8b\text{-}NHCO}$ = 6.9 Hz, $^3J_{H8b\text{-}H7}$ = 2.7 Hz, 1H, H8b), 3.71 (brs, 1H, -O*H*), 3.46 (ddd, $^2J_{H8a\text{-}H8b}$ = 14.3 Hz, $^3J_{H8a\text{-}H7}$ = 7.8 Hz, $^3J_{H8a\text{-}NHCO}$ = 5.2 Hz, 1H, H8a); ^{13}C NMR (126 MHz, $CDCl_3$) δ: 163.67 (C9), 163.34 (dd, $^1J_{C\text{-}F}$ = 249.2 Hz, $^3J_{C\text{-}F}$ = 12.3 Hz, ArC3/C5), 146.09 (t, $^3J_{C\text{-}F}$ = 8.3 Hz, ArC1), 137.95 (thiopheneC10), 130.83 (thiopheneC13), 128.93 (thiopheneC11), 127.98 (thiopheneC12), 108.91 (m, AXX'-system, ArC2/C6), 103.34 (t, $^2J_{C\text{-}F}$ = 25.2 Hz, ArC4), 73.22 (C7), 48.04 (C8); Elemental analysis calcd. (%) for $C_{13}H_{11}F_2NO_2S$: C, 55.12; H, 3.91; N, 4.94; found: C, 55.34; H, 4.18; N, 5.12.

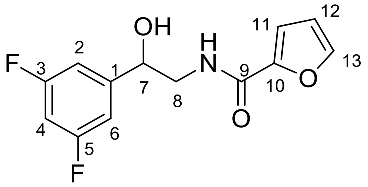

N-(2-(3,5-difluorophenyl)-2-hydroxyethyl)furan-2-carboxamide (22b) was synthesized from **22b** (0.6 g, 2.26 mmol) according to the general procedure I; white solid (0.6 g, quant.); R_f = 0.3 (hexanes/AcOEt 5:5); ^{1}H NMR (300 MHz, $CDCl_3$) δ: 7.48 – 7.44 (m, 1H, furaneH), 7.16 (d, J = 3.6 Hz, 1H, furaneH), 7.01 – 6.89 (m, 2H, ArH2/H6), 6.81 – 6.65 (m, 2H, ArH4 and –N*H*), 6.53 (dd, J_1 = 3.6 Hz, J_2 = 1.7 Hz, 1H, furaneH), 4.94 (dd, $^3J_{H7\text{-}H8a}$ = 7.6 Hz, $^3J_{H7\text{-}H8b}$ = 3 Hz, 1H, H7), 3.85 (ddd, $^2J_{gem}$ = 14.3 Hz, $^3J_{H8b\text{-}NHCO}$ = 7 Hz, $^3J_{H8b\text{-}H7}$ = 3 Hz, 1H, H8b), 3.48 (ddd, $^2J_{gem}$ = 14.3 Hz, $^3J_{H8a\text{-}H7}$ = 7.6 Hz, $^3J_{H8a\text{-}NHCO}$ = 5.4 Hz, 1H, H8a), 1.61 (brs, -O*H*); ^{13}C NMR (63 MHz, $CDCl_3$) δ: 163.31 (dd, $^1J_{C\text{-}F}$ = 248.8 Hz, $^3J_{C\text{-}F}$ = 12.6 Hz, ArC3/C5), 160.09 (C9), 147.36 (furaneC10), 146.04 (t, $^3J_{C\text{-}F}$ = 8.4 Hz, ArC1), 144.57 (furaneC13), 115.32 (furaneC), 112.52 (furaneC), 108.92 (m, AXX'-system, ArC2/C6), 103.32 (t, $^2J_{C\text{-}F}$ = 25.4 Hz, ArC4), 73.34 (t, $^4J_{C\text{-}F}$ = 2 Hz, C7), 47.45 (C8); Elemental analysis calcd. (%) for $C_{13}H_{11}F_2NO_3$: C, 58.43; H, 4.15; N, 5.24; found: C, 58.21; H, 4.39; N, 5.07.

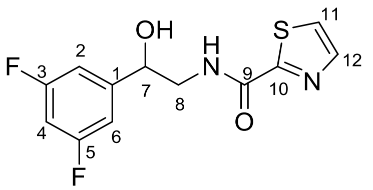

***N*-(2-(3,5-difluorophenyl)-2-hydroxyethyl)thiazole-2-carboxamide (22d)** was synthesized from **21d** (0.2 g, 0.71 mmol) according to the general procedure **I**; white solid (144 mg, 71%); R_f = 0.2 (hexanes/AcOEt 7:3); ^{1}H NMR (500 MHz, $CDCl_3$) *δ*: 7.86 (d, *J* = 3.1 Hz, 1H, thiazoleH12), 7.72 (brs, 1H, -N*H*), 7.61 (d, *J* = 3.1 Hz, 1H, thiazoleH11), 7.00 – 6.94 (m, 2H, ArH2/H6), 6.74 (tt, $^3J_{H\text{-}F}$ = 8.9 Hz, $^4J_{H\text{-}H}$ = 2.3 Hz, 1H, ArH4), 4.97 (dd, $^3J_{H7\text{-}H8a}$ = 8.9 Hz, $^3J_{H7\text{-}H8b}$ = 3.1 Hz, 1H, H7), 3.88 (ddd, ^2J = 14.3 Hz, $^3J_{H8b\text{-}NHCO}$ = 7 Hz, $^3J_{H8b\text{-}H7}$ = 3.1 Hz, 1H, H8b), 3.52 (ddd, ^2J = 14.3 Hz, $^3J_{H8a\text{-}H7}$ = 7.9 Hz, $^3J_{H8a\text{-}NHCO}$ = 5.4 Hz, 1H, H8a), 2.35 (brs, -O*H*); ^{13}C NMR (126 MHz, $CDCl_3$) *δ*: 163.36 (dd, $^1J_{C\text{-}F}$ = 248.6 Hz, $^3J_{C\text{-}F}$ = 13 Hz, ArC3/C5), 163.05 (thiazoleC10), 160.96 (C9), 145.82 (t, $^3J_{C\text{-}F}$ = 8.7 Hz, ArC1), 143.84 (thiazoleC12), 125.18 (thiazoleC11), 108.95 (m, AXX′-system, ArC2/C6), 103.48 (t, $^2J_{C\text{-}F}$ = 25.1 Hz, ArC4), 73.02 (C7), 47.62 (C8); Elemental analysis calcd. (%) for $C_{12}H_{10}F_2N_2O_2S$: C, 50.70; H, 3.55; N, 9.85; found: C, 50.48; H, 3.83; N, 9.64.

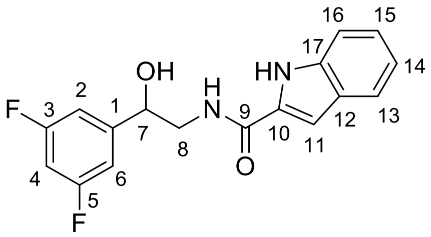

***N*-(2-(3,5-difluorophenyl)-2-hydroxyethyl)-1*H*-indole-2-carboxamide (22i)** was synthesized from **21i** (0.38 g, 1.2 mmol) according to the general procedure L; pale yellow solid (153 mg, 40%); R_f = 0.4 (hexanes/AcOEt 6:4); *Due to its limited solubility, the compound could not be purified, and acquisition of a ^{13}C NMR spectrum was not feasible. Purity was assessed by ^{1}H NMR only, and the compound was used without further purification*. ^{1}H NMR (500 MHz, DMSO-d_6) *δ*: 11.56 (s, 1H, indoleNH), 8.55 (t, *J* – 5.6 Hz, 1H, -N*H*CO-), 7.60 (d, *J* = 8 Hz, 1H, indoleH16), 7.41 (d, *J* 8.3 Hz, 1H, indoleH13), 7.17 (t, *J* = 7.3 Hz, 1H, indoleH), 7.14 – 7.00 (m, 5H, ArH2/H6, ArH4, indoleH x 2), 5.85 (brs, 1H, -O*H*), 4.84 – 4.78 (m, 1H, H7), 3.54 – 3.47 (m, 1H, H8a), 3.46 – 3.40 (m, 1H, H8b); Elemental analysis calcd. (%) for $C_{17}H_{14}F_2N_2O_2$: C, 64.55; H, 4.46; N, 8.86 found: C, 64.37; H, 4.71; N, 8.62.

General procedure for the synthesis of thioesters 24a, 24b, 24d and 24i (N)

The corresponding alcohols were converted to the crude alkyl chlorides (**23a**, **23b**, **23d** and **23i**) according to general procedure F (synthesis of chlorides *via* MsCl or $SOCl_2$) and used directly for the formation of the corresponding thioesters following general procedure G. The intermediates were sufficiently pure as judged by TLC and ^{1}H NMR.

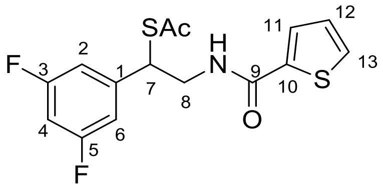

S-(1-(3,5-difluorophenyl)-2-(thiophene-2-carboxamido)ethyl) ethanethioate (24a) was synthesized from **23a** (0.33 g, 1.09 mmol) according to the general procedure G; This product was additionally purified by FCC using hexanes/AcOEt 9:1 to 7:3; white solid (0.162 g, 43%); R_f = 0.3 (hexanes/AcOEt 7:3); ^{1}H NMR (250 MHz, $CDCl_3$) δ: 7.48 (dd, J_1 = 5 Hz, J_2 = 0.9 Hz, 1H, thiopheneH13), 7.44 (dd, J_1 = 3.9 Hz, J_2 = 0.9 Hz, 1H, thiopheneH11), 7.07 (dd, J_1 = 5 Hz, J_2 = 3.9 Hz, 1H, thiopheneH12), 6.94 – 6.86 (m, 2H, ArH2/H6), 6.74 (tt, $^3J_{H\text{-}F}$ = 8.8 Hz, $^4J_{H\text{-}H}$ = 2.3 Hz, 1H, ArH4), 6.35 (brs, 1H, -N*H*), 4.80 (dd, $^3J_{H7\text{-}H8a}$ = 9 Hz, $^3J_{H7\text{-}H8b}$ = 6.8 Hz, 1H, H7), 3.94 – 3.83 (m, 2H, H8), 2.35 (s, 3H, S(CO)C*H₃*); ^{13}C NMR (75 MHz, $CDCl_3$) δ: 195.02 (S(*C*O)CH₃), 163.24 (dd, $^1J_{C\text{-}F}$ = 250.6 Hz, $^3J_{C\text{-}F}$ = 12.8 Hz, ArC3/C5), 161.98 (C9), 142.46 (t, $^3J_{C\text{-}F}$ = 9.5 Hz, ArC1), 138.41 (thiopheneC10), 130.48 (thiopheneC), 128.46 (thiopheneC), 127.87 (thiopheneC), 115.15 (m, AXX'-system, ArC2/C6), 103.85 (t, $^2J_{C\text{-}F}$ = 25.2 Hz, ArC4), 47.31 (t, $^4J_{C\text{-}F}$ = 2 Hz, C7), 44.44 (C8), 29.85 (S(CO)*C*H₃); Elemental analysis calcd. (%) for $C_{15}H_{13}F_2NO_2S_2$: C, 52.77; H, 3.84; N, 4.10; found: C, 52.54; H, 4.11; N, 3.92.

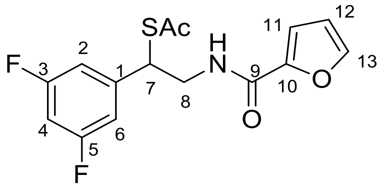

S-(1-(3,5-difluorophenyl)-2-(furan-2-carboxamido)ethyl) ethanethioate (24b) was synthesized from **23b** (0.64 g, 2.24 mmol) according to the general procedure G; Purified by FCC using hexanes/AcOEt 8:2 gradually to 6:4; dark brown oil (0.18 g, 25%); R_f = 0.4 (hexanes/AcOEt 6:4); ^{1}H NMR (500 MHz, $CDCl_3$) δ: 7.45 – 7.41 (m, 1H, furaneH), 7.12 (d, J = 3.4 Hz, 1H, furaneH), 6.94 – 6.87 (m, 2H, ArH2/H6), 6.73 (tt, $^3J_{H\text{-}F}$ = 8.8 Hz, $^4J_{H\text{-}H}$ = 2.2 Hz, 1H, ArH4), 6.52 (brs, 1H, -N*H*), 6.50 (dd, J_1 = 3.4 Hz, J_2 = 1.7 Hz, 1H, furaneH), 4.78 (dd, $J_{H7\text{-}H8a}$ = $J_{H7\text{-}H8b}$ = 7.7 Hz, 1H, H7), 3.92 – 3.83 (m, 2H, H8a/H8b), 2.34 (S, 3H, -SCOC*H₃*); ^{13}C NMR (126 MHz, $CDCl_3$) δ: 194.10 (-S*C*OCH₃), 163.26 (dd, $^1J_{C\text{-}F}$ = 250.6 Hz, $^3J_{C\text{-}F}$ = 13 Hz, ArC3/C5), 158.4 (C9), 147.65 (furaneC10), 144.29 (furaneC13), 142.83 (t, $^3J_{C\text{-}F}$ = 9.3 Hz, ArC1), 114.92 (furaneC11), 112.39 (furaneC12), 111.17 (m, AXX'-system, ArC2/C6), 103.76 (t, $^2J_{C\text{-}F}$ = 25 Hz, ArC4), 47.41 (C7), 43.19 (C8), 30.61 (-SCO*C*H₃); Elemental analysis calcd. (%) for $C_{15}H_{13}F_2NO_3S$: C, 55.38; H, 4.03; N, 4.31 found: C, 55.24; H, 4.27; N, 4.19.

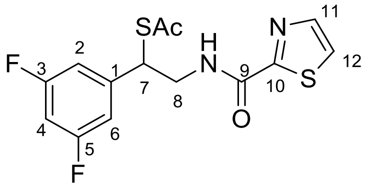

***S*-(1-(3,5-difluorophenyl)-2-(thiazole-2-carboxamido)ethyl) ethanethioate (24d**) was synthesized from **23d** (0.147 g, 0.49 mmol) according to the general procedure G; The product was purified by FCC using hexanes/AcOEt 8:2; orange oil (106 mg, 64%); R_f = 0.4 (hexanes/AcOEt 7:3); ^{1}H NMR (250 MHz, $CDCl_3$) δ: 7.85 (d, J = 3.0 Hz, 1H, thiazoleH12), 7.58 (d, J = 3.0 Hz, 1H, thiazoleH11), 7.46 (brs, 1H, -N*H*), 6.97 – 6.86 (m, 2H, ArH2/H6), 6.73 (tt, $^3J_{H\text{-}F}$ = 8.8 Hz, $^4J_{H\text{-}H}$ = 2.3 Hz, 1H, ArH4), 4.82 (t, $J_{H7\text{-}H8a}$ = $J_{H7\text{-}H8b}$ = 7.5 Hz 1H, H7), 4.03 – 3.77 (m, 2H, H8), 2.34 (s, 3H, –SCOC*H₃*); ^{13}C NMR (63 MHz, $CDCl_3$) δ: 193.61 (-S*C*OCH₃), 163.1 (dd, $^1J_{C\text{-}F}$ = 250.1 Hz, $^3J_{C\text{-}F}$ = 12.6 Hz, ArC3/C5), 162.98 (C9),159.53 (thiazoleC10), 143.60 (thiazoleC12), 142.54 (t, $^3J_{C\text{-}F}$ = 8.8 Hz, ArC1), 124.87 (thiazoleC11), 111.03 (m, AXX′-system, ArC2/C6), 103.65 (t, $^2J_{C\text{-}F}$ = 25 Hz, ArC4), 47.10 (C7), 43.40 (C8), 30.95 (-SCO*C*H₃); Elemental analysis calcd. (%) for $C_{14}H_{12}F_2N_2O_2S_2$: C, 49.11; H, 3.53; N, 8.18; found: C, 48.93; H, 3.75; N, 7.99.

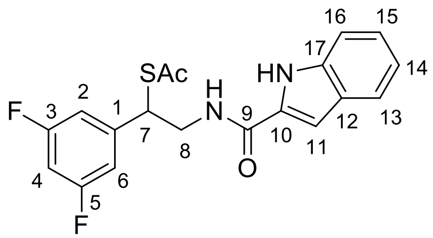

***S*-(1-(3,5-difluorophenyl)-2-(1*H*-indole-2-carboxamido)ethyl) ethanethioate (24i)** was synthesized from **23i** (0.18 g, 0.54 mmol) according to the general procedure G; The product was purified by FCC using hexanes/AcOEt 8:2 to 7:3; light orange solid (122 mg, 61%); R_f = 0.5 (hexanes/AcOEt 7:3); ^{1}H NMR (500 MHz, $CDCl_3$) δ: 9.21 (s, 1H, indoleN*H*), 7.65 (d, J = 8 Hz, 1H, indoleH16), 7.43 (d, J = 8.5 Hz, 1H, indoleH13), 7.30 (t, J = 7.4 Hz, 1H, indoleH), 7.15 (t, J = 7.4 Hz, 1H, indoleH), 6.95 – 6.88 (m, 2H, ArH2/H6), 6.79 (s,1H, indoleH11), 6.75 (tt, $^3J_{H\text{-}F}$ = 8.9 Hz, $^4J_{H\text{-}H}$ = 2.4 Hz, 1H, ArH4), 6.50 (-N*H*). 4.84 (dd, $^3J_{H7\text{-}H8a}$ = 9.5 Hz, $^3J_{H7\text{-}H8b}$ = 6.6 Hz, 1H, H7), 4.02 – 3.95 (m, 1H, H_{8a}), 3.95 – 3.88 (m, 1H, H_{8b}), 2.35 (s, 3H, -SCOCH_3); ^{13}C NMR (126 MHz, $CDCl_3$) δ: 195.02 (-S*C*OCH_3), 163.32 (dd, $^1J_{C\text{-}F}$ = 249.4 Hz, $^3J_{C\text{-}F}$ = 12.9 Hz, ArC3/C5), 161.71 (C9), 142.4 (t, $^3J_{C\text{-}F}$ = 9.2 Hz, ArC1), 136.51 (indoleC), 130.21 (indoleC), 127.79 (indoleC), 124.98 (indoleC), 122.23 (indoleC), 121.0 (indoleC), 112.10 (indoleC11), 111.18 (m, AXX'-system, ArC2/C6), 103.92 (t, $^2J_{C\text{-}F}$ = 25.3 Hz, ArC4), 102.54 (indoleC), 47.39 (C7), 44.26 (C8), 29.88 (-SCO*C*H_3); Elemental analysis calcd. (%) for $C_{19}H_{16}F_2N_2O_2S$: C, 60.95; H, 4.31; N, 7.48; found: C, 61.11; H, 4.46; N, 7.62.

General procedure for the synthesis of thiols 25a, 25b, 25d and 25i

The same general procedure (I) was used as for the 1,′1-diaryl thiol analogues.

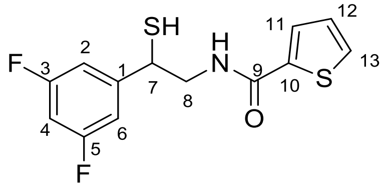

***N*-(2-(3,5-difluorophenyl)-2-mercaptoethyl)thiophene-2-carboxamide (25a)** was synthesized from **24a** (0.162 g, 0.48 mmol) according to the general procedure I; This product was additionally purified by FCC using hexanes/AcOEt 8:2; yellow oil (94 mg, 66%); R_f = 0.4 (hexanes/AcOEt 7:3); ^{1}H NMR (500 MHz, $CDCl_3$) δ: 7.49 (dd, J_1 = 5 Hz, J_2 = 0.9 Hz, 1H, thiopheneH13), 7.47 (dd, J_1 = 3.9 Hz, J_2 = 0.9 Hz, 1H, thiopheneH11), 7.07 (dd, J_1 = 5 Hz, J_2 = 3.9 Hz, 1H, thiopheneH12), 6.94 – 6.88 (m, 2H, ArH2/H6), 6.73 (tt, $^3J_{H\text{-}F}$ = 8.7 Hz, $^4J_{H\text{-}H}$ = 2.2 Hz, 1H, ArH4), 6.41 (brs, 1H, -N*H*), 4.30 – 4.23 (m, 1H, H7), 3.88 – 3.81 (m, 1H, H8a), 3.74 – 3.66 (m, 1H, H8b), 2.04 (d, J = 6.8 Hz, 1H. –S*H*); ^{13}C NMR (126 MHz, $CDCl_3$) δ: 163.32 (dd, $^1J_{C\text{-}F}$ = 249.8 Hz, $^3J_{C\text{-}F}$ = 12.6 Hz, ArC3/C5), 162.08 (C9), 145.34 (t, $^3J_{C\text{-}F}$ = 9.5 Hz, ArC1), 138.26 (thiopheneC10), 130.55 (thiopheneC), 128.60 (thiopheneC), 127.90 (thiopheneC), 110.51 (m, AXX'-system, ArC2/C6), 103.57 (t, $^2J_{C\text{-}F}$ = 25.4 Hz, ArC4), 47.64 (C7), 43.07 (C8); Elemental analysis calcd. (%) for $C_{13}H_{11}F_2NOS_2$: C, 52.16; H, 3.70; N, 4.68; found: C, 51.98; H, 3.96; N, 4.41.

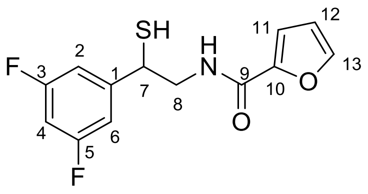

***N*-(2-(3,5-difluorophenyl)-2-mercaptoethyl)furan-2-carboxamide (25b)** was synthesized from **24b** (0.18 g, 0.55 mmol) according to the general procedure **I**; The product was purified by FCC using hexanes/AcOEt 7:3; peachy solid (0.12 g, 77%); R_f = 0.3 (hexanes/AcOEt 7:3). ^{1}H NMR (500 MHz, $CDCl_3$) *δ*: 7.44 – 7.43 (m, 1H, furaneH13), 7.13 (d, *J* = 3.4 Hz, furaneH11), 6.95 – 6.83 (m, 2H, ArH2/H6), 6.73 (tt, $^3J_{H\text{-}F}$ = 8.7 Hz, $^4J_{H\text{-}H}$ = 2.2 Hz, 1H, ArH4), 6.61 (brs, 1H, -N*H*), 6.51 (dd, J_1 = 3.4 Hz, J_2 = 1.6 Hz, 1H, furanH12) 4.29 – 4.17 (m, 1H, H7), 3.92 – 3.65 (m, 2H, H8), 2.05 (d, *J* = 6.5 Hz, 1H. –S*H*); ^{13}C NMR (63 MHz, $CDCl_3$) *δ*: 163.29 (dd, $^1J_{C\text{-}F}$ = 249.3 Hz, $^3J_{C\text{-}F}$ = 12.8 Hz, ArC3/C5), 158.52 (C9), 147.54 (furaneC10), 145.25 (t, $^3J_{C\text{-}F}$ = 9.0 Hz, ArC1), 144.33 (furaneC13), 114.99 (furaneC11), 112.42 (furaneC12), 110.51 (m, AXX'-system, ArC2/C6), 103.57 (t, $^2J_{C\text{-}F}$ = 25.3 Hz, ArC4), 46.80 (C7), 43.06 (C8); Elemental analysis calcd. (%) for $C_{13}H_{11}F_2NO_2S$: C, 55.12; H, 3.91; N, 4.94; found: C, 55.28; H, 4.17; N, 5.11.

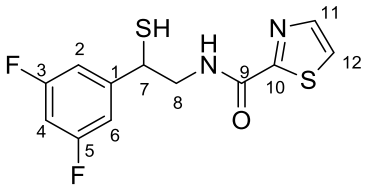

***N*-(2-(3,5-difluorophenyl)-2-mercaptoethyl)thiazole-2-carboxamide (25d)** was synthesized from **24d** (0.106 g, 0.31 mmol) according to the general procedure I; The product was purified by FCC using hexanes/AcOEt 7:3; white solid (49.2 mg, 53%); R_f = 0.4 (hexanes/AcOEt 7:3); ^{1}H NMR (500 MHz, $CDCl_3$) *δ*: 7.85 (d, *J* = 3.0 Hz, 1H, thiazoleH12), 7.59 (d, *J* = 3.0 Hz, 1H, thiazoleH11), 7.55 (brs, 1H, -N*H*), 6.95 – 6.89 (m, 2H, ArH2/H6), 6.74 (tt, $^3J_{H\text{-}F}$ = 8.8 Hz, $^4J_{H\text{-}H}$ = 2.3 Hz, 1H, ArH4), 4.29 – 4.22 (m, 1H, H7), 3.93 – 3.86 (m, 1H, H8a), 3.82 – 3.74 (m, 1H, H8b), 2.08 (d, *J* = 6.6 Hz, 1H, –S*H*); ^{13}C NMR (126 MHz, $CDCl_3$) *δ*: 163.35 (dd, $^1J_{C\text{-}F}$ = 249.6 Hz, $^3J_{C\text{-}F}$ = 12.4 Hz, ArC3/C5), 163.12 (C9), 159.78 (thiazoleC10), 145.10 (t, $^3J_{C\text{-}F}$ = 8.5 Hz, ArC1), 143.79 (thiazoleC12), 125.08 (thiazoleC11), 110.52 (m, AXX'-system, ArC2/C6), 103.69 (t, $^2J_{C\text{-}F}$ = 24.9 Hz, ArC4), 47.27 (C7), 43.03 (C8); Elemental analysis calcd. (%) for $C_{12}H_{10}F_2N_2OS_2$: C, 47.99; H, 3.36; N, 9.33; found: C, 47.86; H, 3.57; N, 9.17.

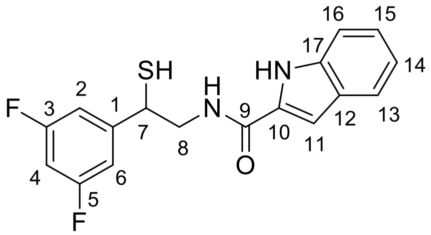

***N*-(2-(3,5-difluorophenyl)-2-mercaptoethyl)-1*H*-indole-2-carboxamide (25i)** was synthesized from **24i** (0.122 g, 0.33 mmol) according to the general procedure I; The product was purified by FCC using hexanes/AcOEt 7:3; pale yellow solid (61.8 mg, 57%); R_f = 0.4 (hexanes/AcOEt 7:3); ^{1}H NMR (500 MHz, $CDCl_3$) *δ*: 9.44 (s, 1H, indoleN*H*), 7.64 (d, *J* = 8.1 Hz, 1H, indoleH16), 7.44 (d, *J* = 8.1 Hz, 1H, indoleH13), 7.30 (t, *J* = 7.2 Hz, 1H, indoleH), 7.15 (t, *J* = 7.2 Hz, 1H, indoleH), 6.99 – 6.86 (m, 2H, ArH2/H6), 6.81 (s,1H, indoleH11), 6.75 (tt, $^3J_{H\text{-}F}$ = 9 Hz, $^4J_{H\text{-}H}$ = 2.4 Hz, 1H, ArH4), 6.51 (-N*H*). 4.17 – 4.08 (m, 1H, H7), 3.95 – 3.87 (m, 1H, H8a), 3.82 – 3.73 (m, 1H, H8b), 2.06 (1H, -S*H*); ^{13}C NMR (126 MHz, $CDCl_3$) *δ*: 163.20 (dd, $^1J_{C\text{-}F}$ =

250.5 Hz, $^3J_{C\text{-}F}$ = 13.1 Hz, ArC3/C5), 161.70 (C9), 145.13 (t, $^3J_{C\text{-}F}$ = 8.2 Hz, ArC1), 136.59 (indoleC), 130.06 (indoleC), 127.68 (indoleC), 125.05 (indoleC), 122.19 (indoleC), 121.04 (indoleC), 112.14 (indoleC), 110.49 (m, AXX'-system, ArC2/C6), 103.65 (t, $^2J_{C\text{-}F}$ = 25.3 Hz, ArC4), 102.68 (indoleC11), 47.23 (C7), 43.18 (C8); Elemental analysis calcd. (%) for $C_{17}H_{14}F_2N_2OS$: C, 61.43; H, 4.25; N, 8.43; found: C, 61.27; H, 4.41; N, 8.27.

General procedure for the synthesis of *O*-Substituted *N*-Hydroxyphthalimides 27n and 32q (P)

To a solution of *N*-hydroxyphthalimide (1 equiv.) in anhydrous DMF (1.02 M), NaH 60% w/w (1.25 equiv.) was added at 0 °C. The mixture was stirred at rt for 30 min. Thereafter, the appropriate halogenide (1.5 equiv.) was added and the reaction was stirred at rt overnight. Then, water was added and a solid precipitate formed. The precipitate was filtered under vacuum and washed with water and a solution of n-pentane/Et_2O 7:3. The solid was dried over P_2O_5, to afford the desired product.

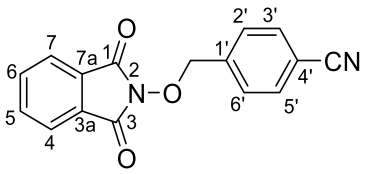

4-(((1,3-dioxoisoindolin-2-yl)oxy)methyl)benzonitrile (**27n**) was synthesized from 4-(bromomethyl)benzonitrile according to the general procedure P. Pink solid (695.3 mg, 99 %); ^{1}H NMR (400 MHz, $CDCl_3$) *δ*: 7.85 – 7.80 (m, 2H, Ar), 7.80 – 7.74 (m, 2H, Ar), 7.72 – 7.64 (m, 4H, Ar- Pthal), 5.26 (s, 2H, OCH_2); ^{13}C NMR (101 MHz, $CDCl_3$) δ 163.51 (C_1, C_3), 139.11 ($C_{1'}$), 134.84 ($C_{3'}$, $C_{5'}$), 132.51 (C_5, C_6), 130.05 (C_{3a}, C_{7a}) 129.30 ($C_{2'}$, $C_{6'}$), 123.85 (C_4, C_7), 118.58 (C-N), 113.28 ($C_{4'}$), 78.81 (CH_2). Elemental analysis calcd. (%) for $C_{16}H_{10}N_2O_3$: C, 69.06; H, 3.62; N, 10.07; Found: C, 69.32; H, 3.94; N, 10.29.

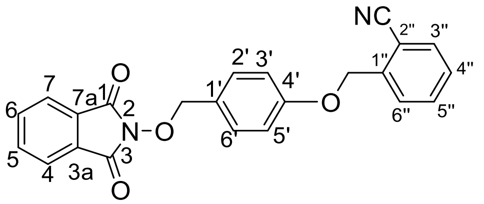

2-((4-(((1,3-dioxoisoindolin-2-yl)oxy)methyl)phenoxy)methyl)benzonitrile (32q) was synthesized from 2-((4-(chloromethyl)phenoxy)methyl)benzonitrile (**30q**) according to the general procedure P. Off-white solid (171.4mg, 46 %); R_f = 0.63 (DCM); ^{1}H NMR (600 MHz, DMSO) *δ*: 7.94 – 7.90 (m, 1H, Ar), 7.86 (s, 4H, Ar- Pthal), 7.78 – 7.72 (m, 2H, Ar), 7.58 (ddd, J = 7.7, 6.8, 2.0 Hz, 1H, Ar), 7.49 – 7.45 (m, 2H, Ar), 7.11 – 7.06 (m, 2H, Ar), 5.27 (s, 2H, OCH_2), 5.11 (s, 2H, OCH_2); ^{13}C NMR (101 MHz, DMSO) *δ*: 163.16 (C_1, C_3), 158.65 (C4'), 139.85 (C1"), 134.81 (C5"), 133.46 ($C_{3''}$), 133.34 (C_5, C_6), 131.53 (C_{3a}, C_{7a}), 129.71 ($C_{2'}$, $C_{6'}$), 129.16 ($C_{1'}$), 128.53 ($C_{4''}$), 126.98 ($C_{6''}$), 123.26 (C_4, C_7), 117.20 (C- N), 114.72 ($C_{3'}$, $C_{5'}$), 111.35 ($C_{2''}$), 78.81 (OCH_2), 67.57 (OCH_2). Elemental analysis calcd. (%) for $C_{23}H_{16}N_2O_4$: C, 71.87; H, 4.20; N, 7.29. Found: C, 71.99; H, 4.43; N, 7.51.

General procedure for the synthesis of *O*-Substituted Hydroxylamines 28n and 33q (Q)

To a solution of the appropriate *N*-hydroxyphthalimide (1 equiv.) in CH_2Cl_2 (0.51 M), hydrazine monohydrate 64% w/w (2 equiv.) is added, and the reaction is stirred at rt for 3 h. The white precipitate formed is filtered, washed with DCM, and the filtrate is concentrated to afford the corresponding hydroxylamine.

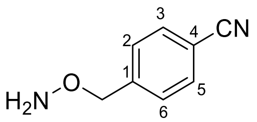

4-((aminooxy)methyl)benzonitrile (28n) was synthesized from the compound **26n** (263.7 mg, 0.95 mmol) according to the general procedure Q. Purified by CC; off-yellow oil (139 mg, 99 %);^{1}H NMR (400 MHz, DMSO-d$_6$) δ 7.82 - 7.80 (m, 2H, Ar), 7.52 - 7.50 (m, 2H, Ar), 6.20 (s, 2H, NH_2), 4.65 (s, 2H, OCH_2); ^{13}C NMR (101 MHz, D_2O, Hydrochloride salt) *δ*: 141.4 (C_1), 135.9 (C_5, C_3), 132.4 (C_6, C_2), 122.2 (C_4), 114.9 (C-N), 78.8 (OCH_2). Elemental analysis calcd. (%) for $C_8H_8N_2O$: C, 64.85; H, 5.44; N, 18.91; found: C, 64.99; H, 5.23; N, 18.68.

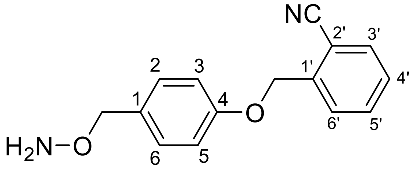

***O*-((4-((aminooxy)methyl)phenoxy)methyl)benzonitrile (33q)** was synthesized from the previous compound **31q** according to the general procedure Q. Purified by CC; colorless oil (117mg, 99%); R_f = 0.11 (DCM); ^{1}H NMR (400 MHz, DMSO-d$_6$) *δ*: 7.94 – 7.88 (m, 1H, Ar), 7.75 (dd, *J* = 6.7, 1.5 Hz, 2H, Ar), 7.57 (ddd, *J* = 7.7, 6.5, 2.2 Hz, 1H, Ar), 7.30 – 7.24 (m, 2H, Ar), 7.04 – 6.99 (m, 2H, Ar), 5.97 (s, 2H, NH_2), 5.24 (s, 2H, OCH_2), 4.49 (s, 2H, OCH_2); ^{13}C NMR (126 MHz, DMSO-d$_6$) *δ*: 157.55 (C_4), 136.13 ($C_{1'}$), 134.56 ($C_{2'}$), 129.93 (C_2, C_6), 129.69 (C_1), 129.28 ($C_{6'}$), 128.05 ($C_{3'}$), 127.60 ($C_{4'}$), 125.34 ($C_{5'}$), 113.99 (C_3, C_5), 112.2 (C-N), 76.19 (OCH_2), 67.49 (OCH_2). Elemental analysis calcd. (%) for $C_{15}H_{14}N_2O_2$: C, 70.85; H, 5.55; N, 11.02; found: C, 70.57; H, 5.85; N, 11.38.

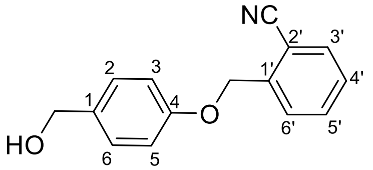

.

2-((4-(Hydroxymethyl)phenoxy)methyl)benzonitrile (30q) To a solution of 4-(hydroxymethyl)phenol (**29**) (1 equiv.) in anhydrous acetonitrile (0.44 M), Cs_2CO_3 (2 equiv.) was added under cooling, and the mixture was stirred at room temperature for 30 min. Then, 2-(bromomethyl)benzonitrile (1 equiv.) was added, and the reaction was left under argon atmosphere and stirring at 82 °C for 20 h (overnight). The reaction mixture was filtered and washed with acetonitrile, and the filtrate was collected and concentrated to afford **30q** as a white solid (578 mg, 99%). The product was used in the next step without further purification; R_f =0.13 (hexane/AcOEt 2:1); ^{1}H NMR (400 MHz, DMSO-d$_6$) *δ*: 7.93 – 7.87 (m, 1H, Ar), 7.77 – 7.70 (m, 2H, Ar), 7.60 – 7.53 (m, 1H, Ar), 7.29 – 7.23 (m, 2H, Ar), 7.03 – 6.97 (m, 2H, Ar), 5.23 (s, 2H, OCH_2), 4.43 (s, 2H, OCH_2); ^{13}C NMR (75 MHz, DMSO-d$_6$) *δ*: 157.9 (C_4), 143.9 ($C_{1'}$), 133.5 (C_1), 132.4 ($C_{3'}$), 133.2 ($C_{5'}$), 129.3 (C_2, C_6), 128.3 ($C_{4'}$), 127.8 ($C_{6'}$), 115.8 (C- N), 114.5 (C_3, C_5), 111.4 ($C_{2'}$), 67.5 (OCH_2), 64.7 (OCH_2); Elemental analysis calcd. (%) for $C_{15}H_{13}NO_2$: C, 75.30; H, 5.48; N, 5.85. Found: C, 75.54; H, 5.19; N, 5.68.

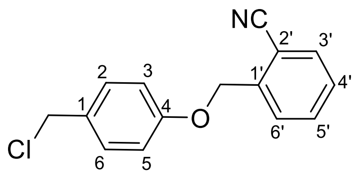

4-((4-(Chloromethyl)phenoxy)methyl)benzonitrile (31q)

To a solution of **30q** (1 equiv.) in thionyl chloride (30 equiv., acting as both solvent and chlorinating reagent) was heated to reflux at 76 °C for 1 h. The reaction mixture was concentrated to afford a beige solid, which was washed with hexane and collected by filtration. The product was used in the next step without further purification (508 mg, 82%); Beige solid (508mg, 82%); R_f = 0.6 (CH_2Cl_2/AcOEt 1:1); ^1H NMR (400 MHz, DMSO-d_6) δ: 7.92 (d, J = 7.7 Hz, 1H, Ar), 7.79 – 7.70 (m, 2H, Ar), 7.61 – 7.54 (m, 1H, Ar), 7.42 – 7.37 (m, 2H, Ar), 7.08 – 7.03 (m, 2H, Ar), 5.26 (s, 2H, OCH_2), 4.73 (s, 2H, CH_2Cl); ^{13}C NMR (75 MHz, DMSO-d_6) δ: 159 (C_4), 143.9 ($C_{1'}$), 132.4 ($C_{3'}$), 133.2 ($C_{5'}$), 130.1 (C_1), 129.6 (C_2, C_6), 128.3 ($C_{4'}$), 127.8 ($C_{6'}$), 115.8 (C-N), 114.5 (C_3, C_5), 111.4 ($C_{2'}$), 67.5 (OCH_2), 46.2 (CH_2Cl); Elemental analysis calcd. (%) for $C_{15}H_{12}ClNO$: C, 69.91; H, 4.69; N, 5.44. Found: C, 69.64; H, 4.51; N, 5.77.

Screening-Level Inhibition Profiles and Concentration–Response Curves

A. Enzymatic Data

This part presents representative three-point inhibition profiles (0.1, 1, and 10 µM) obtained during primary screening. These data were used for initial structure–activity relationship (SAR) assessment. Full concentration–response curves were generated only for selected compounds demonstrating promising activity in the three-point screen and were used for IC_{50} determination.

Classes A & D

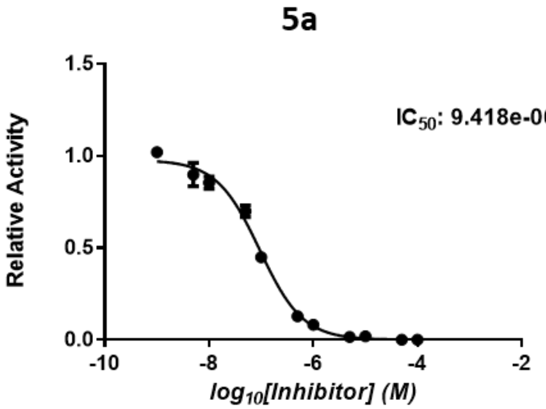

5a
IC50: 9.418e-008 M
Relative Activity
log10[Inhibitor] (M)

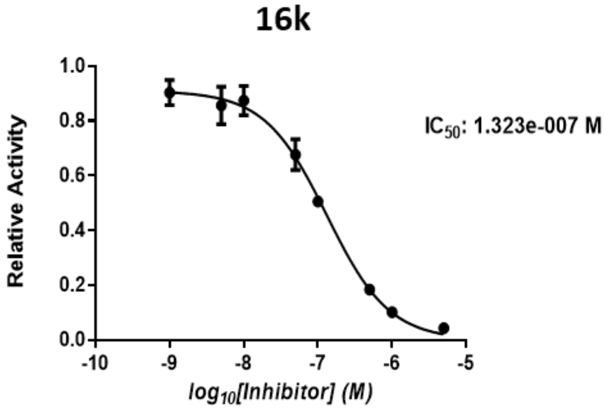

16k
IC50: 1.323e-007 M
Relative Activity
log10[Inhibitor] (M)

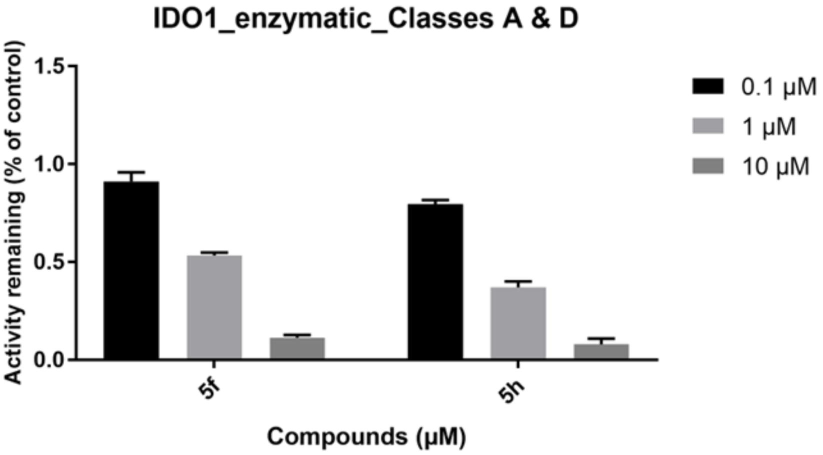

IDO1_enzymatic_Classes A & D
Activity remaining (% of control)
0.1 μM
1 μM
10 μM
5f
5h
Compounds (μM)

CLASS B

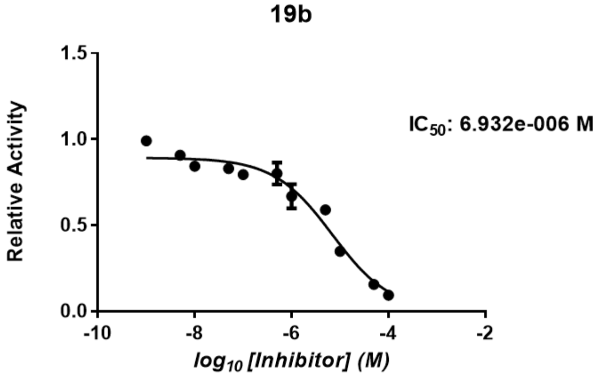

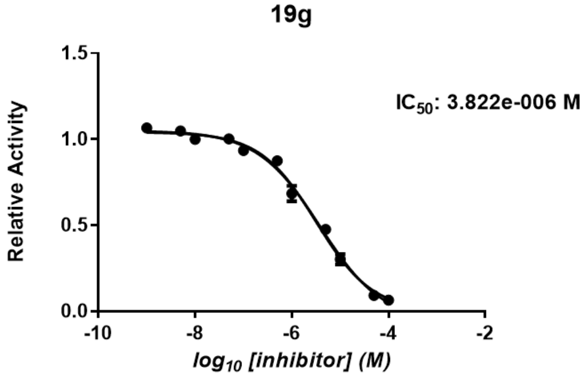

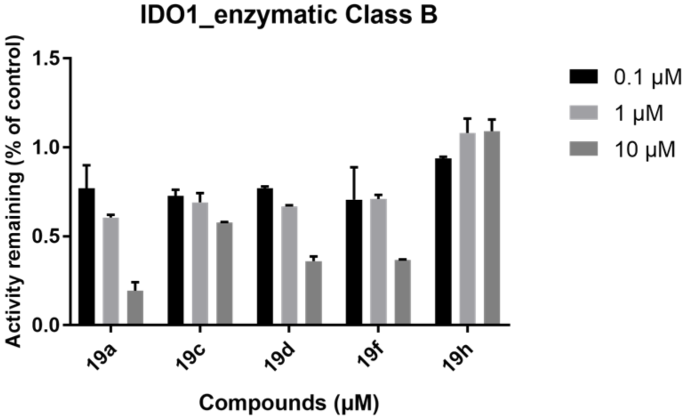

CLASS C

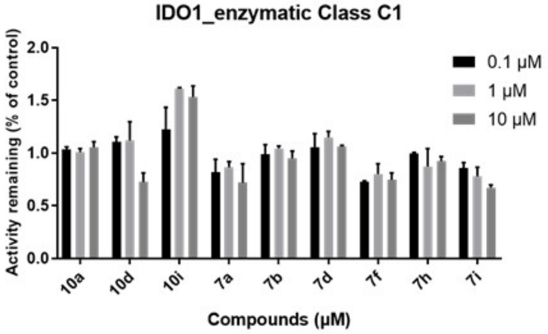

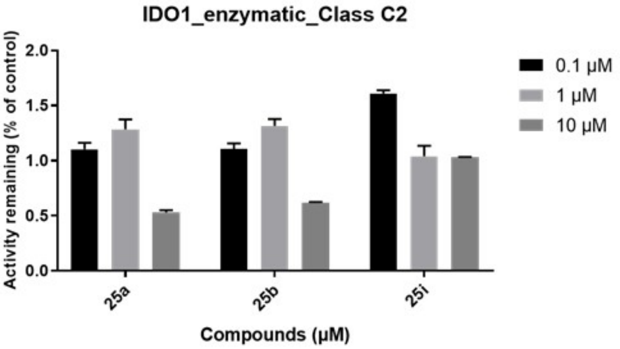

Class E

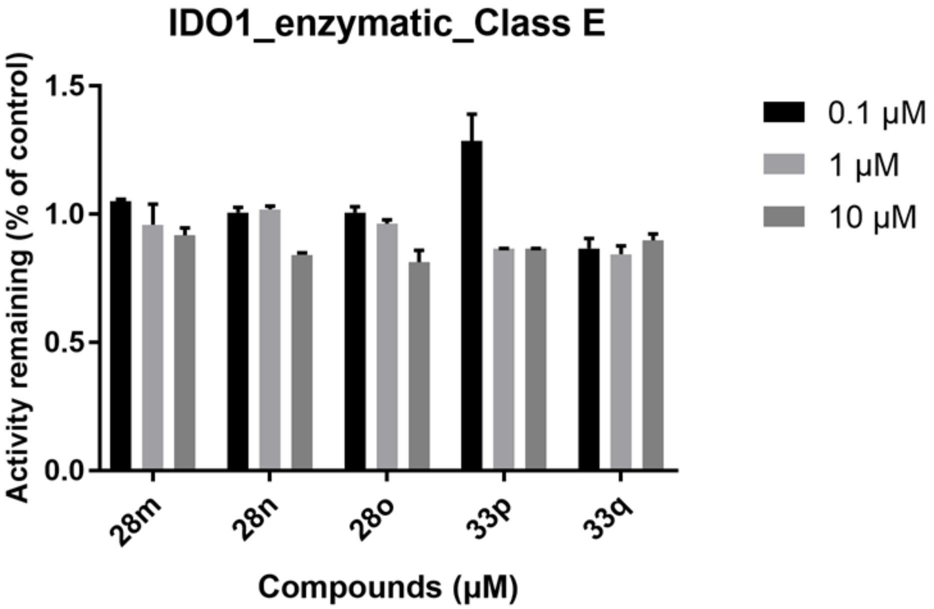

Figure S1. Enzymatic inhibition of recombinant human IDO1 by additional diaryl hydroxylamine derivatives. Compounds are grouped into classes A–E as defined in the main manuscript; class C is further subdivided into C-1 (oximes and short thiols) and C-2 (long thiols). IDO1 activity was assessed under initial-rate conditions by quantifying kynurenine formation using the p-dimethylaminobenzaldehyde (p-DMAB) colorimetric assay. For selected compounds, full concentration–response curves were generated using serial dilutions and used for IC_{50} determination by nonlinear regression (four-parameter logistic model). In parallel, the broader compound set was evaluated as part of initial SAR screening at three fixed concentrations (0.1, 1, and 10 μM), and results are presented as bar plots showing relative activity remaining compared to vehicle-treated controls. Data are normalized to vehicle control (control = 1) and expressed as fraction of control. Data represent independent experiments performed in technical replicates.

CLASSES A & D

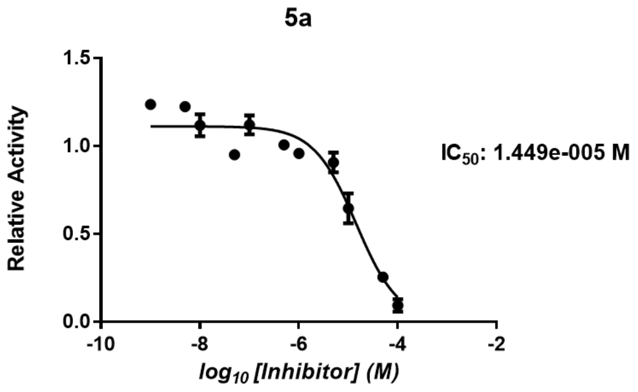

5a
Relative Activity
IC50: 1.449e-005 M
log10 [Inhibitor] (M)

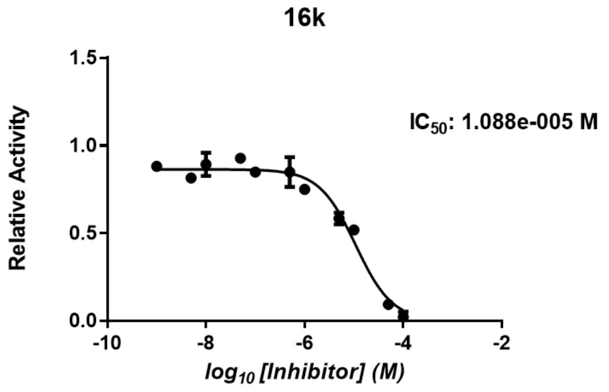

16k
Relative Activity
IC50: 1.088e-005 M
log10 [Inhibitor] (M)

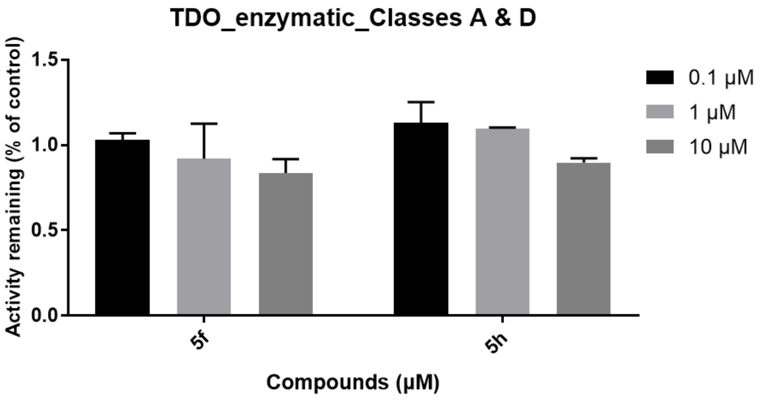

TDO_enzymatic_Classes A & D
Activity remaining (% of control)
0.1 μM
1 μM
10 μM
5f
5h
Compounds (μM)

CLASS B

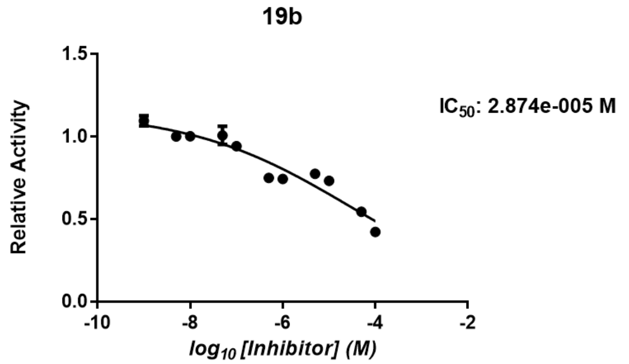

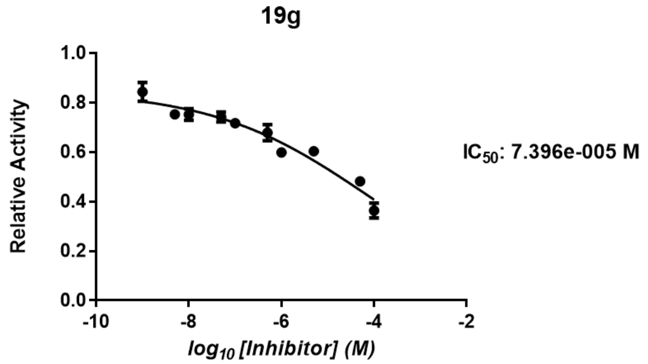

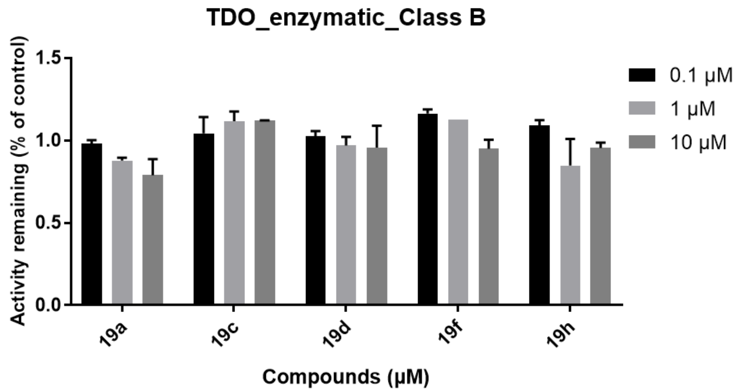

CLASS C

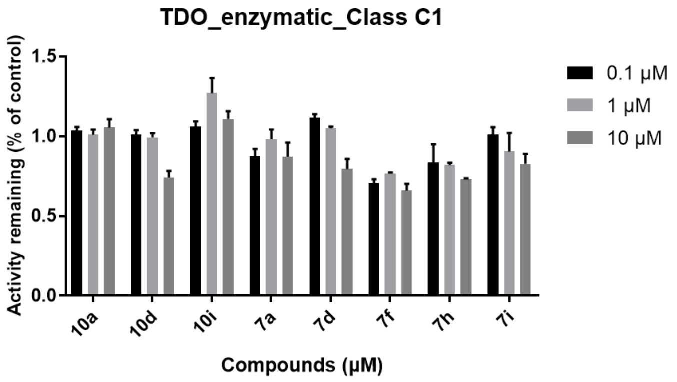

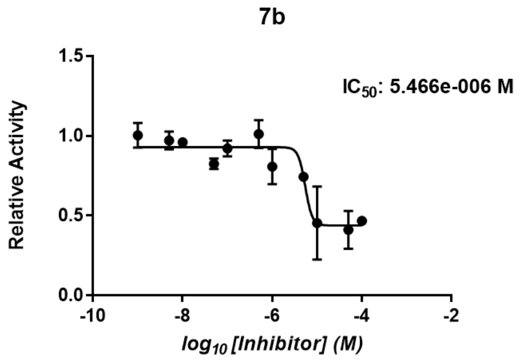

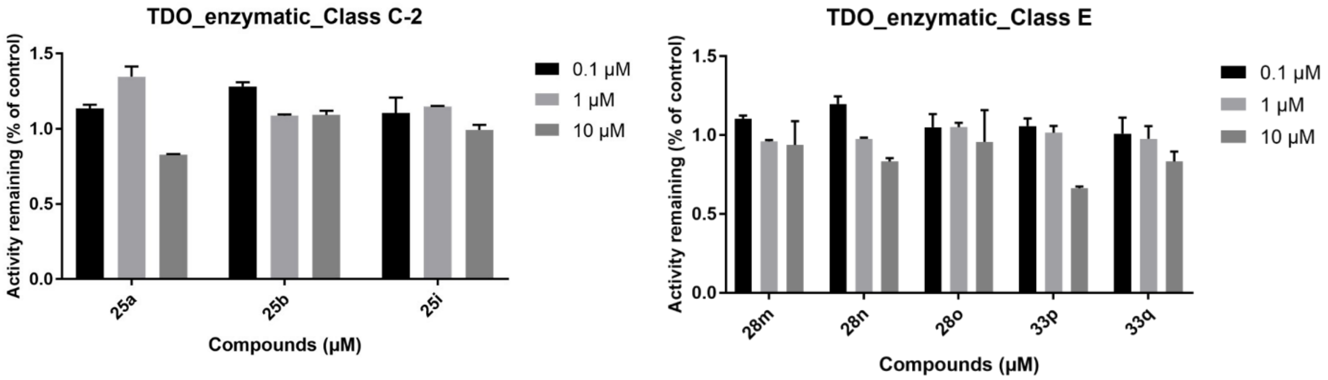

Figure S2. Enzymatic inhibition of recombinant human TDO by additional diaryl hydroxylamine derivatives: Compounds are grouped into classes A–E as defined in the main manuscript; class C is further subdivided into C-1 (oximes and short thiols) and C-2 (long thiols). Enzymatic inhibition of recombinant human TDO was assessed under initial-rate conditions by quantifying kynurenine formation using the p-dimethylaminobenzaldehyde (p-DMAB) colorimetric assay. For selected compounds, full concentration–response curves were generated using serial dilutions and used for IC_{50} determination by nonlinear regression (four-parameter logistic model). In parallel, additional compounds listed in Table 1 were evaluated as part of initial SAR screening at three fixed concentrations (0.1, 1, and 10 µM), and results are presented in bar plots as relative activity remaining compared to vehicle-treated controls. Experimental conditions are as described for Figure S1. Data are normalized to vehicle control (control = 1) and expressed as fraction of control. Data represent independent experiments performed in technical replicates.

B. Cellular Data

CLASSES A & D

5a

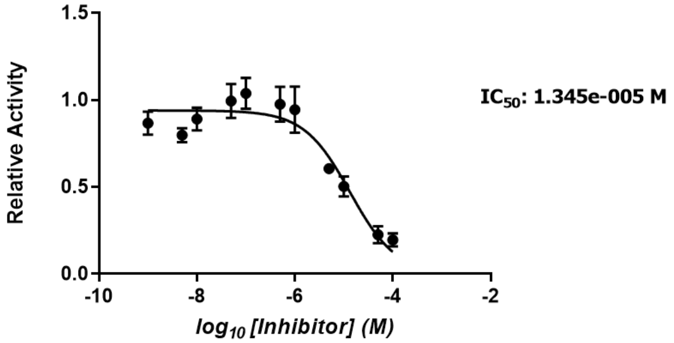

5f

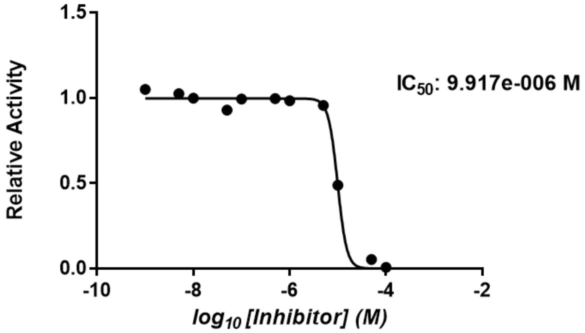

5h

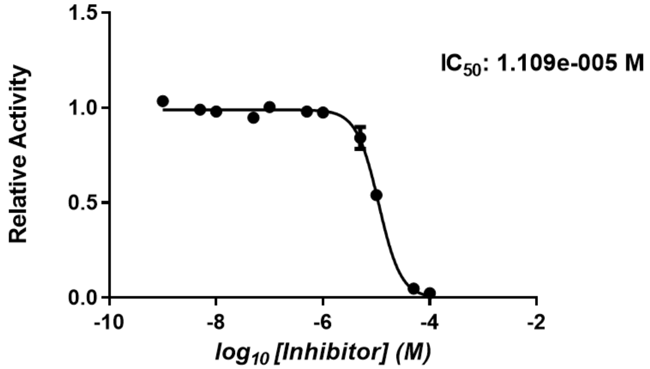

16k

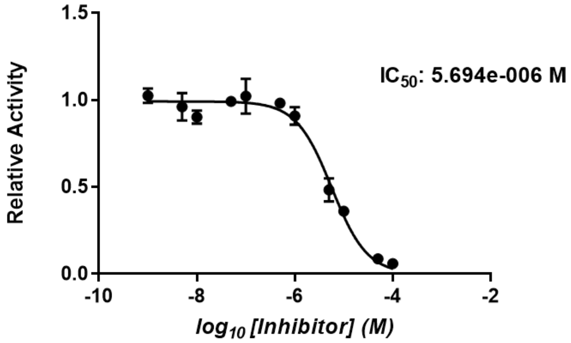

CLASS B

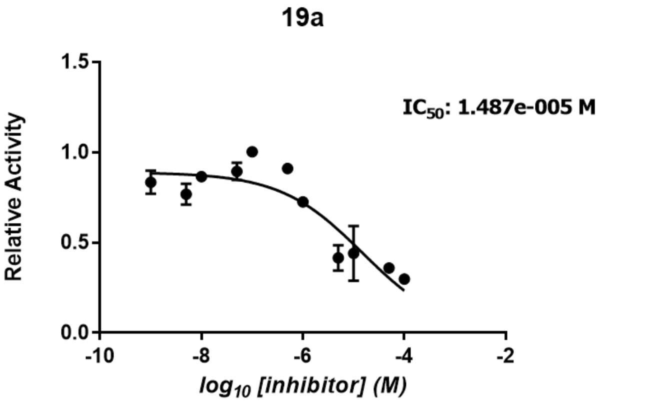

19a
IC$_{50}$: 1.487e-005 M
Relative Activity
log$_{10}$ [inhibitor] (M)
1.5
1.0
0.5
0.0
-10
-8
-6
-4
-2

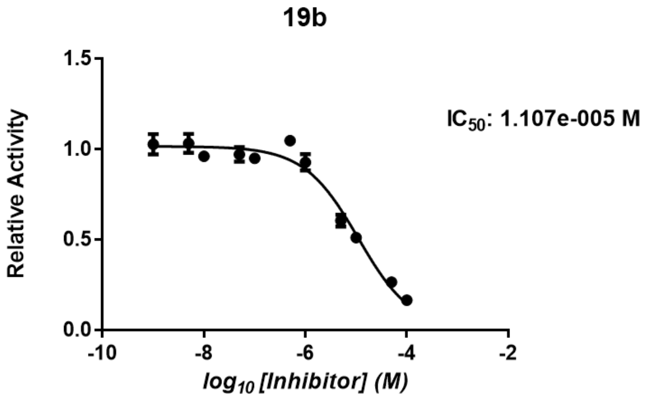

19b
IC$_{50}$: 1.107e-005 M
Relative Activity
log$_{10}$ [Inhibitor] (M)
1.5
1.0
0.5
0.0
-10
-8
-6
-4
-2

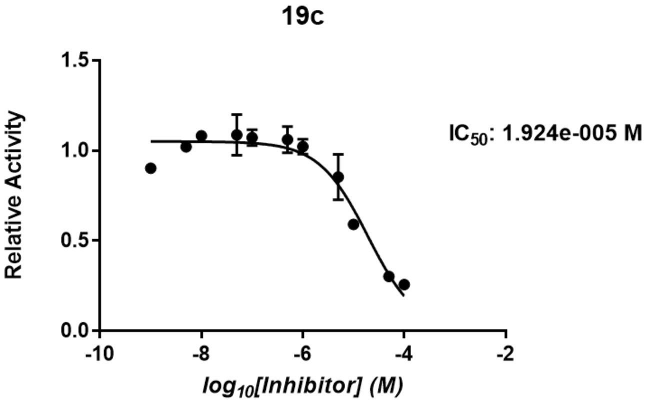

19c
IC$_{50}$: 1.924e-005 M
Relative Activity
log$_{10}$[Inhibitor] (M)
1.5
1.0
0.5
0.0
-10
-8
-6
-4
-2

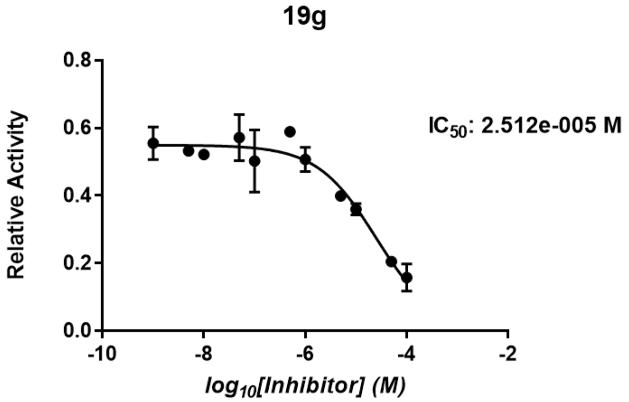

19g
IC$_{50}$: 2.512e-005 M
Relative Activity
log$_{10}$[Inhibitor] (M)
0.8
0.6
0.4
0.2
0.0
-10
-8
-6
-4
-2

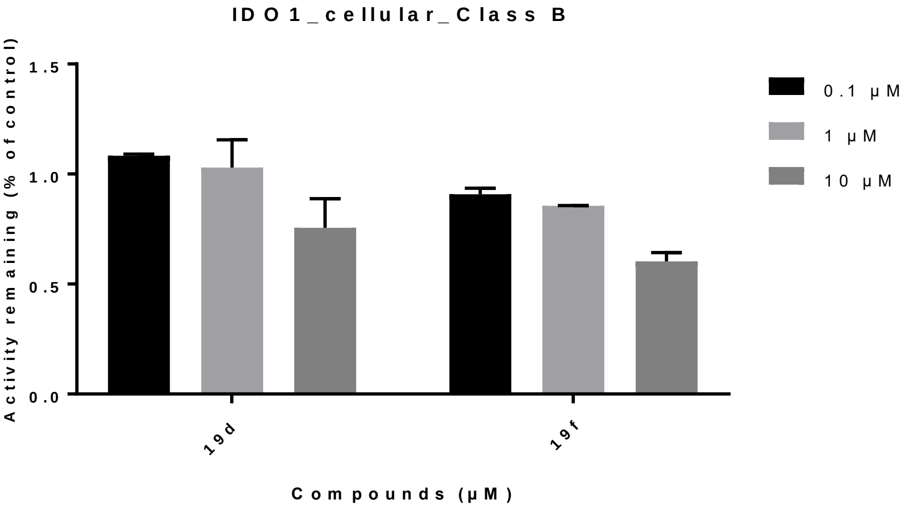

IDO1_cellular_Class B
Activity remaining (% of control)
1.5
1.0
0.5
0.0
0.1 µM
1 µM
10 µM
19d
19f
Compounds (µM)

CLASS C

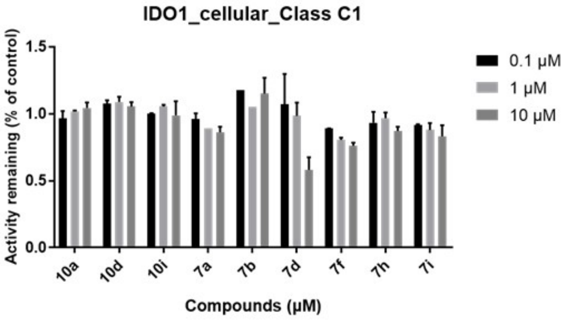

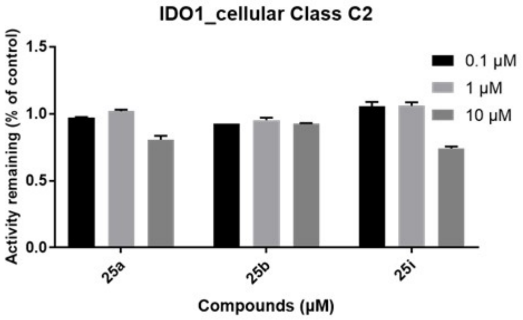

CLASS E

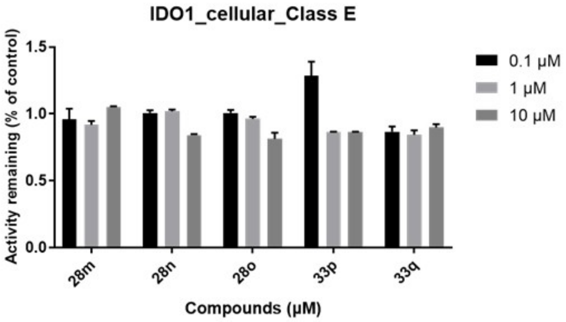

Figure S3: Cellular inhibition of endogenous IDO1 activity in IFNγ-stimulated HeLa cells. Compounds are grouped into classes A–E as defined in the main manuscript; class C is further subdivided into C-1 (oximes and short thiols) and C-2 (long thiols). Endogenous IDO1 activity was evaluated by quantifying kynurenine accumulation in conditioned medium after 48 h of treatment using the p-dimethylaminobenzaldehyde (p-DMAB) derivatization assay. For selected compounds, full cellular concentration–response curves were generated using serial dilutions and used for IC_{50} determination by nonlinear regression (four-parameter logistic model). In parallel, initial SAR screening across the broader compound set was performed at three fixed concentrations (0.1, 1, and 10 μM) and is presented as bar plots for comparative profiling. Data are normalized to vehicle control (control = 1) and expressed as fraction of control. Data represent technical replicates from independent experiments.

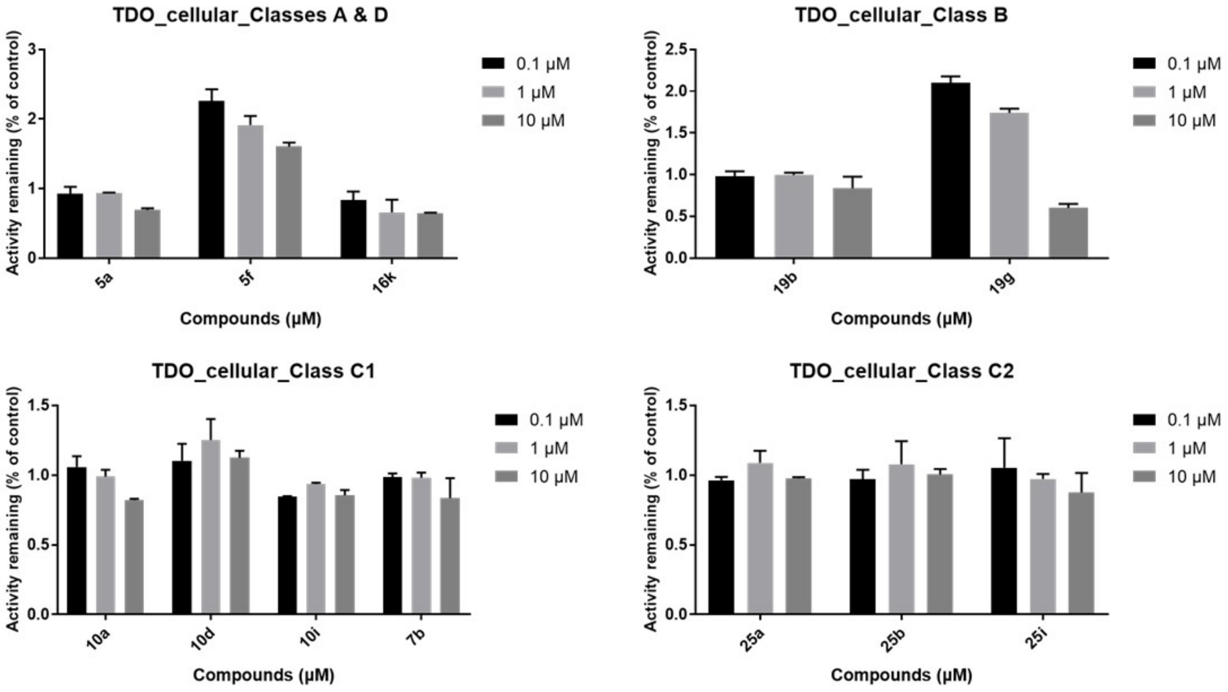

Figure S4: Cellular inhibition of endogenous TDO activity in A172 glioblastoma cells: Compounds are grouped into classes A–E as defined in the main manuscript; class C is further subdivided into C-1 (oximes and short thiols) and C-2 (long thiols). Endogenous TDO activity was evaluated by quantifying kynurenine accumulation in conditioned medium after 48 h of treatment using the p-dimethylaminobenzaldehyde (p-DMAB) derivatization assay. Compounds were evaluated at three fixed concentrations (0.1, 1, and 10 µM) as part of initial SAR screening and are presented as bar plots for comparative profiling. Experimental conditions were identical to those described in Figure S3. Data represent technical replicates from independent experiments. Data are normalized to vehicle control (control = 1) and expressed as fraction of control.

^{1}H and ^{13}C NMR spectra

Figure NMR1. ^{1}H spectrum of **5a** in $CDCl_3$

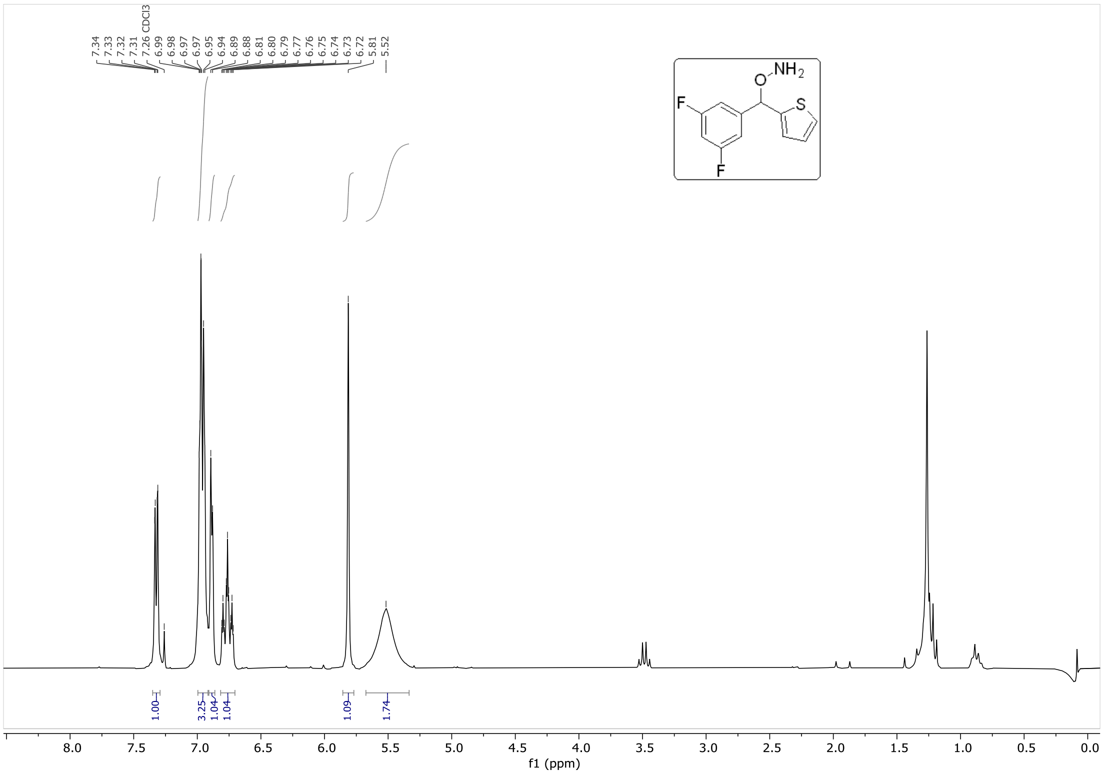

Figure NMR2. ^{13}C spectrum of **5a** in $CDCl_3$

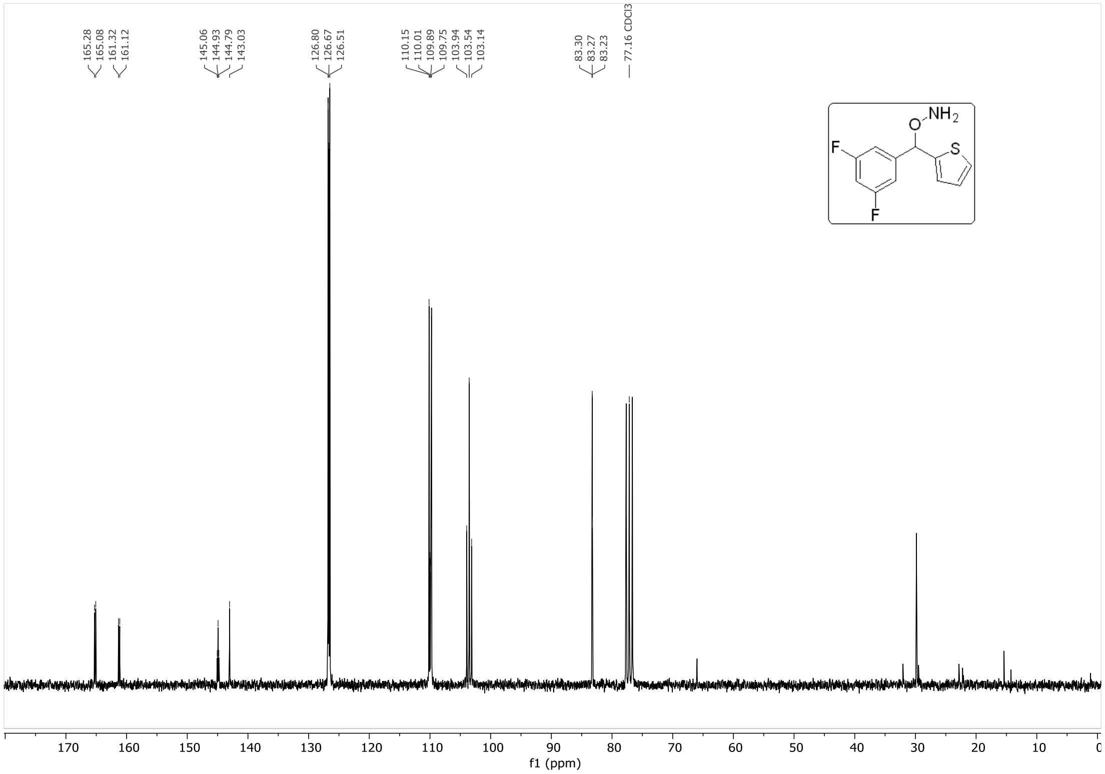

Figure NMR3. ^{1}H spectrum of **5d** in $CDCl_3$

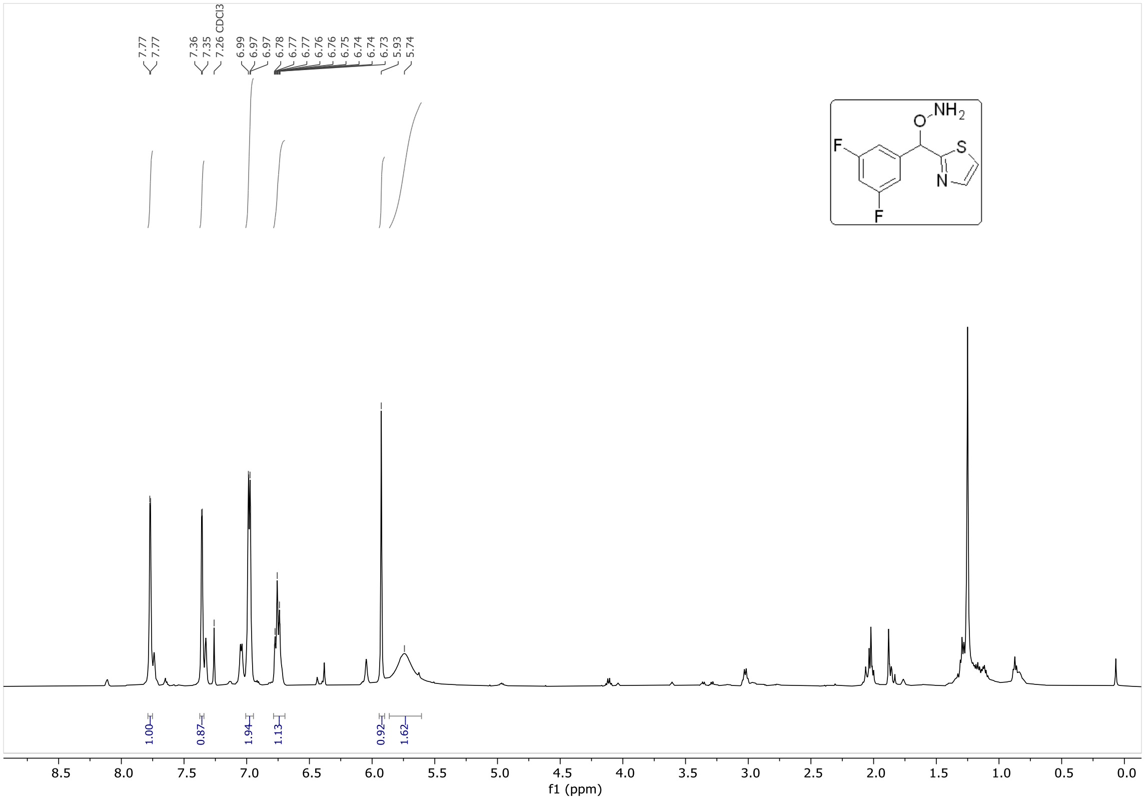

Figure NMR4. ^{13}C spectrum of **5d** in $CDCl_3$

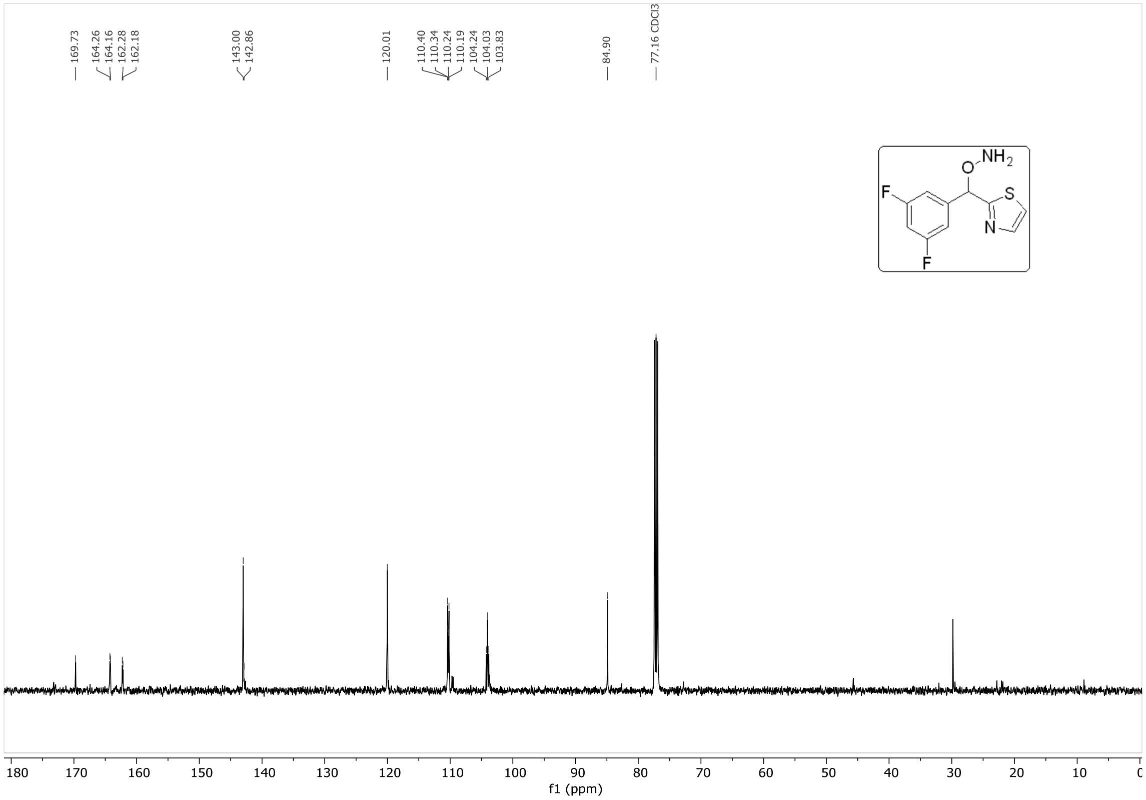

Figure NMR5. ^{1}H spectrum of **5f** in $CDCl_3$

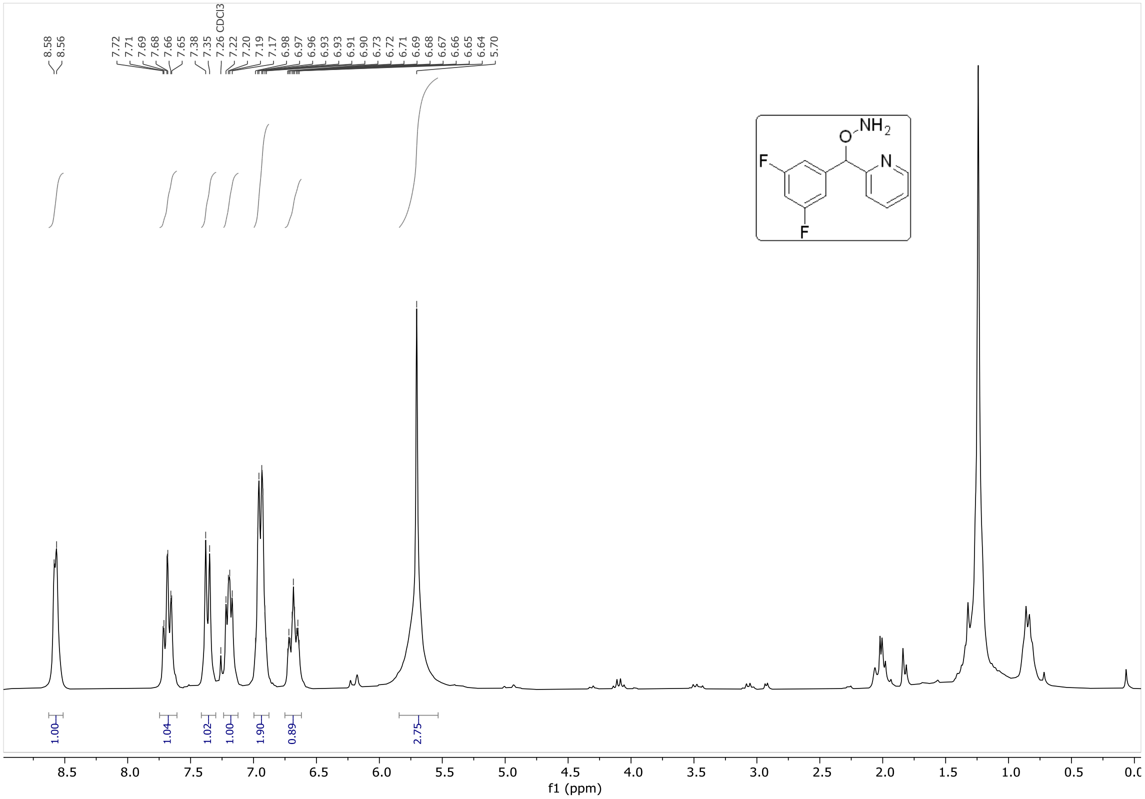

Figure NMR6. ^{13}C spectrum of **5f** in $CDCl_3$

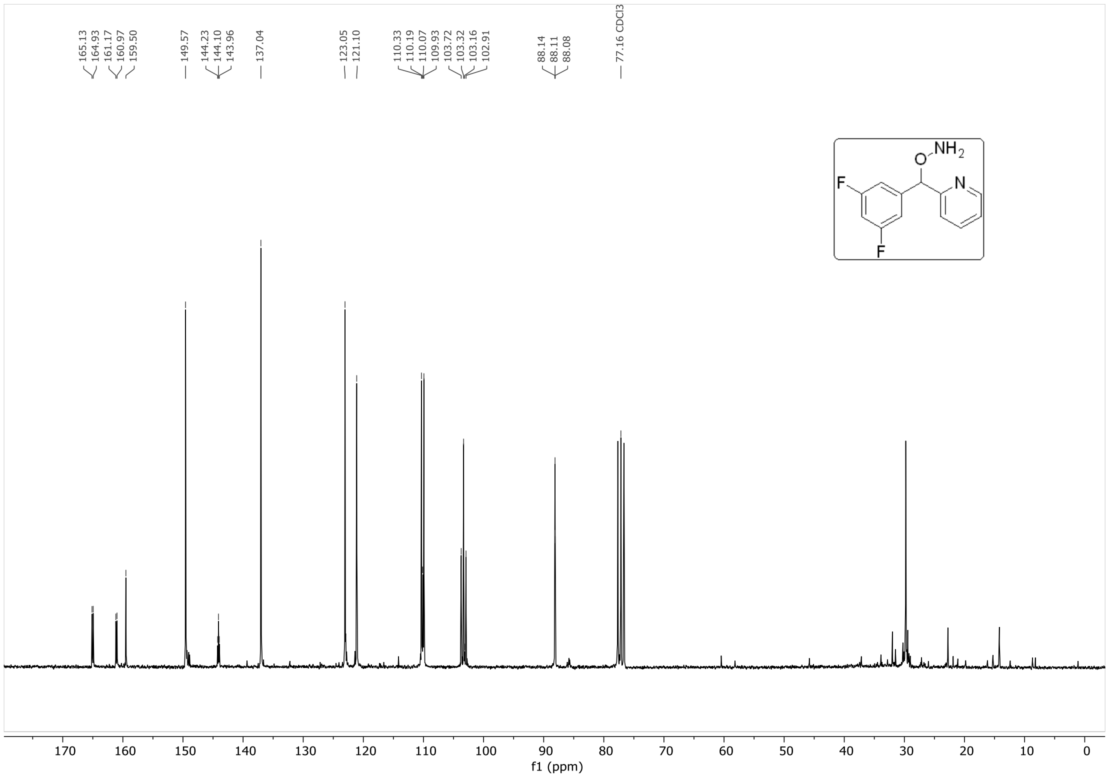

Figure NMR7. ^{1}H spectrum of **5h** in $CDCl_3$

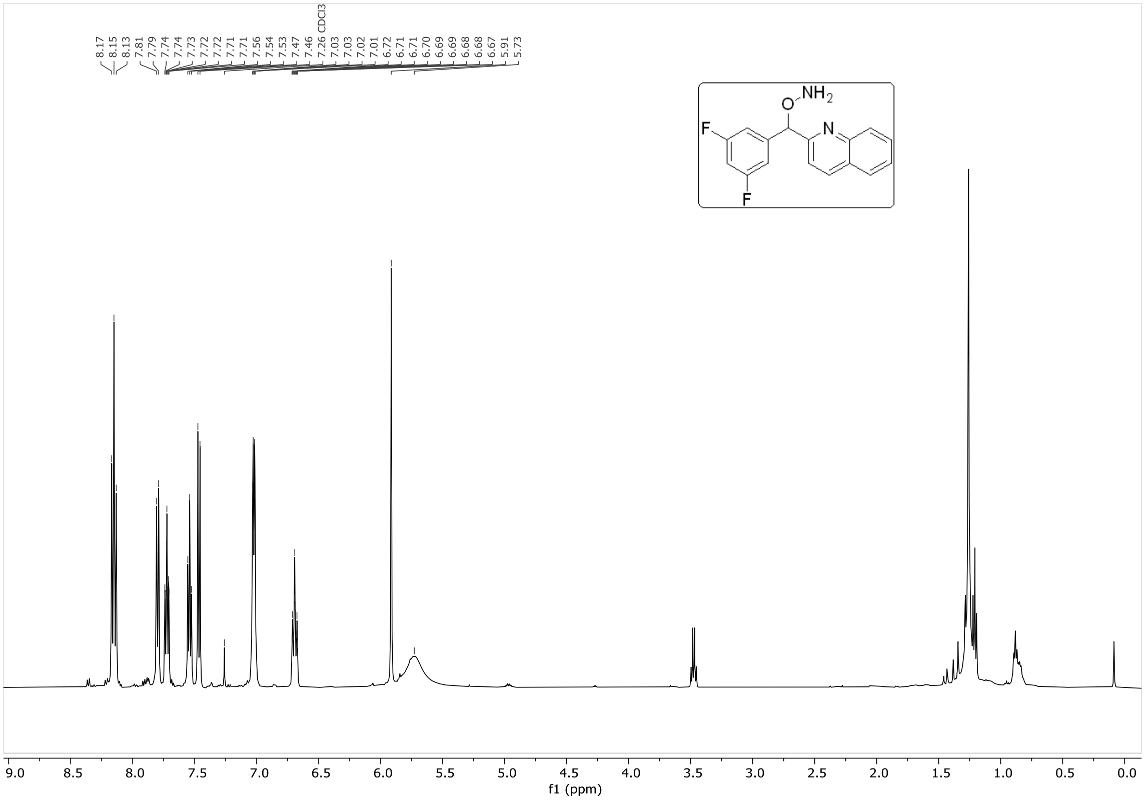

Figure NMR8. ^{13}C spectrum of **5h** in $CDCl_3$

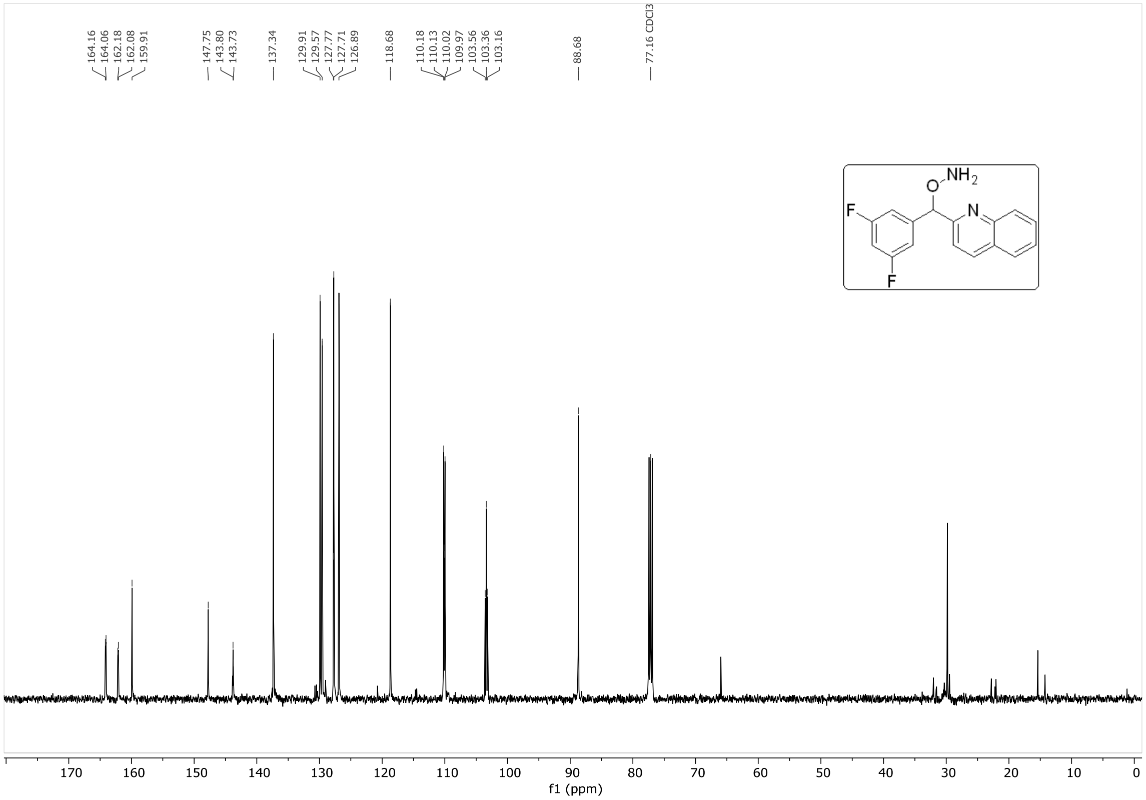

Figure NMR9. ^{1}H spectrum of **16j** in D_2O

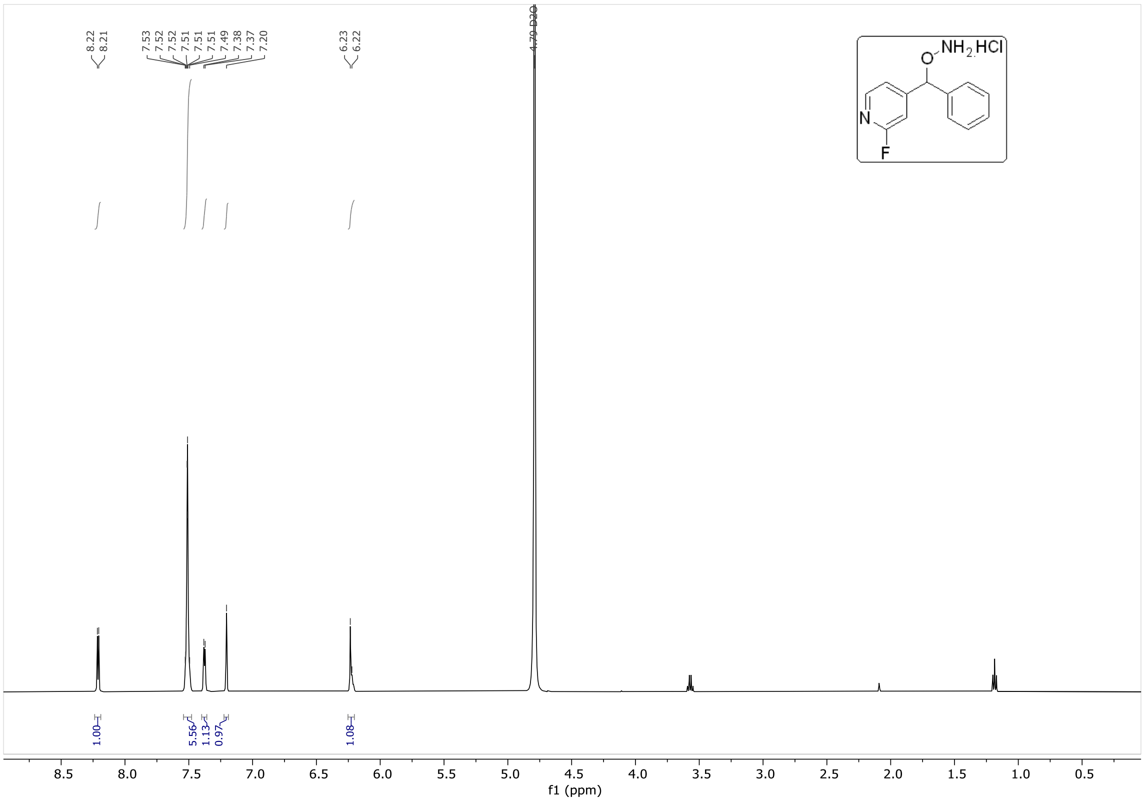

Figure NMR10. ^{13}C spectrum of **16j** in D_2O

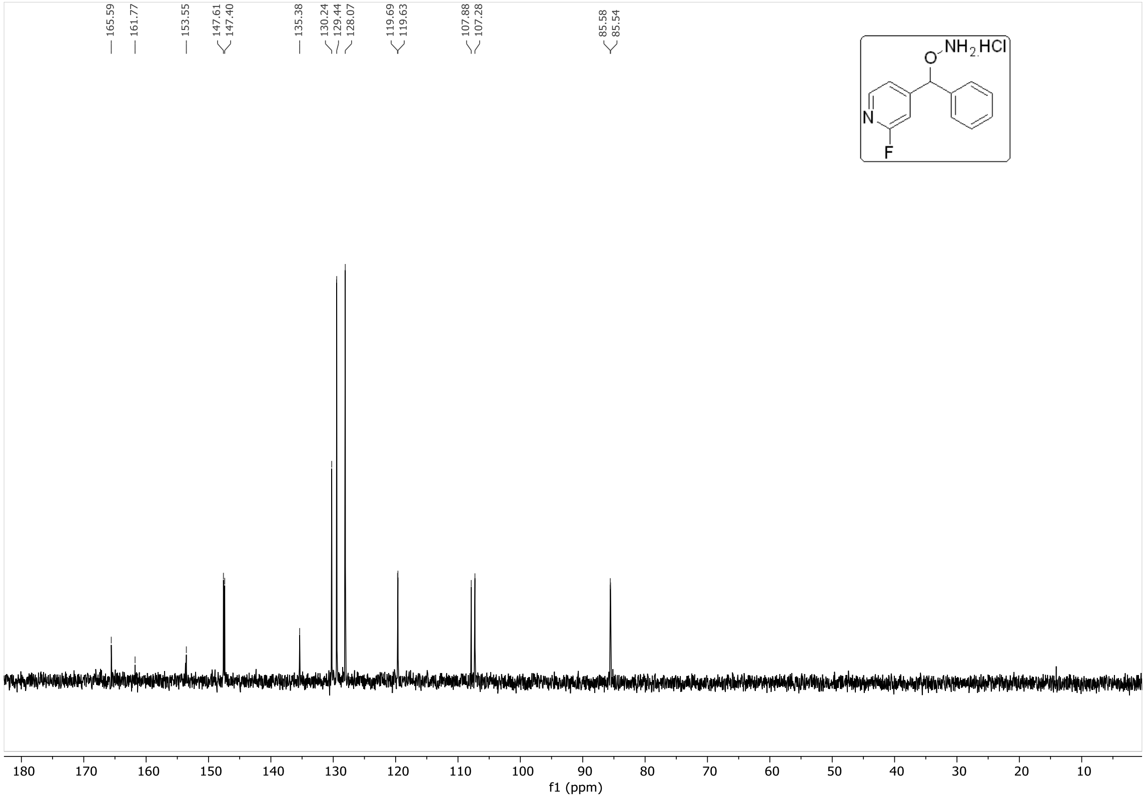

Figure NMR11. ^{1}H spectrum of **16k** in MeOD

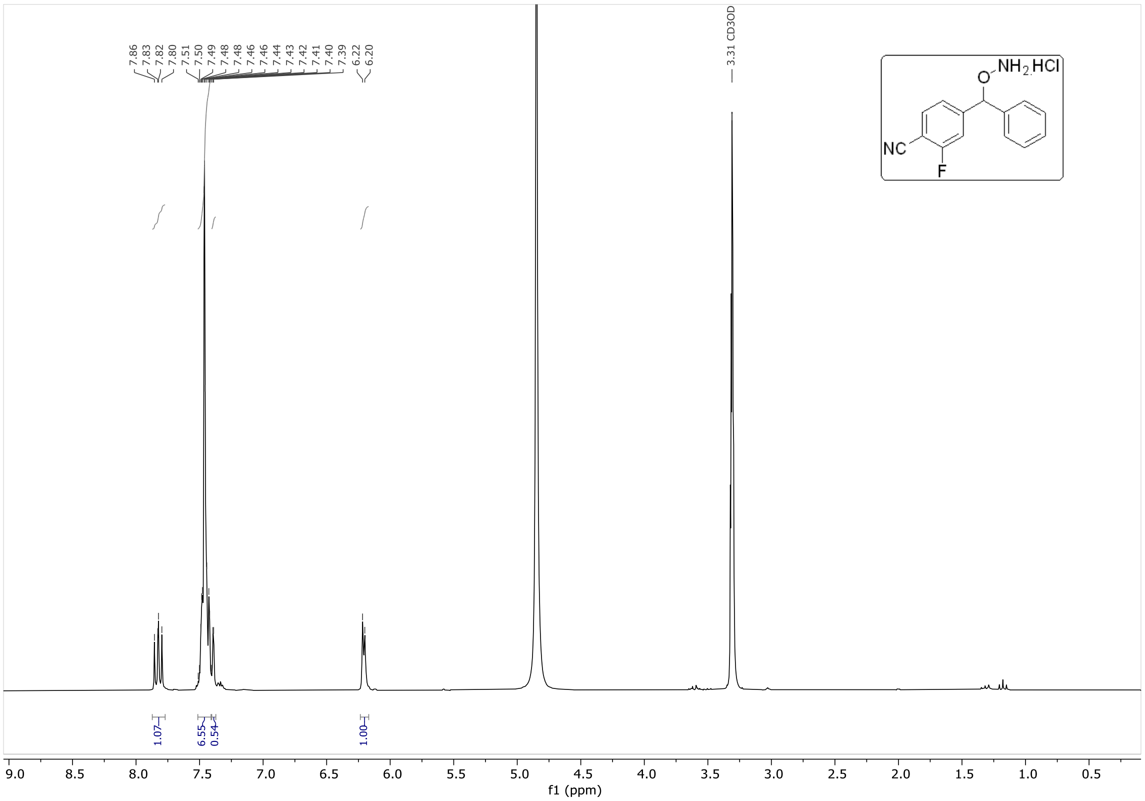

Figure NMR12. ^{13}C spectrum of **16k** in MeOD

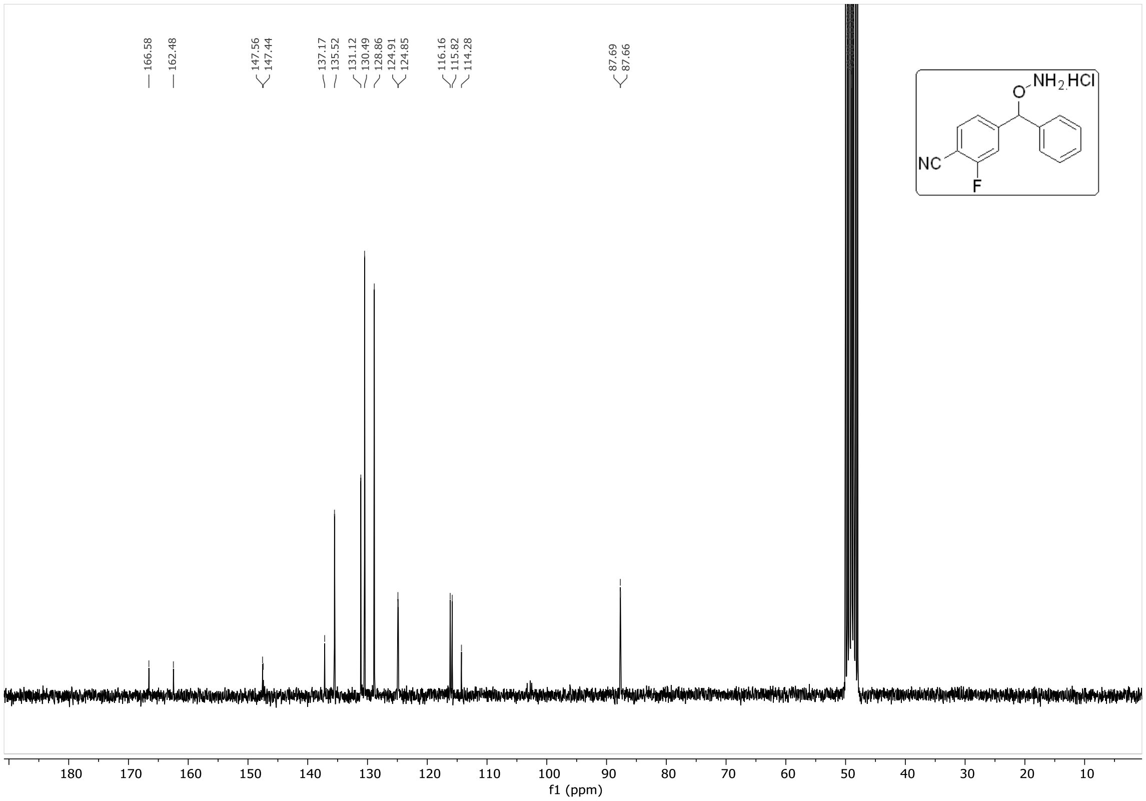

Figure NMR13. ^{1}H spectrum of **7a** in $CDCl_3$

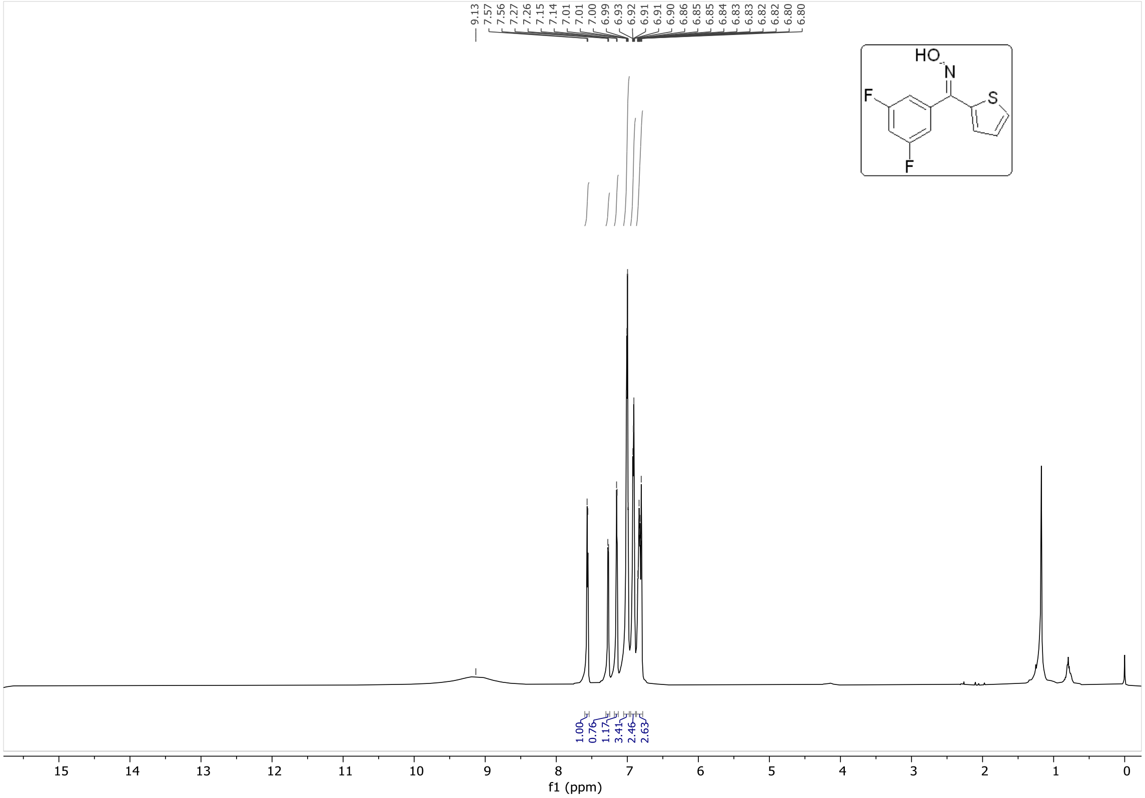

Figure NMR14. ^{13}C spectrum of **7a** in $CDCl_3$

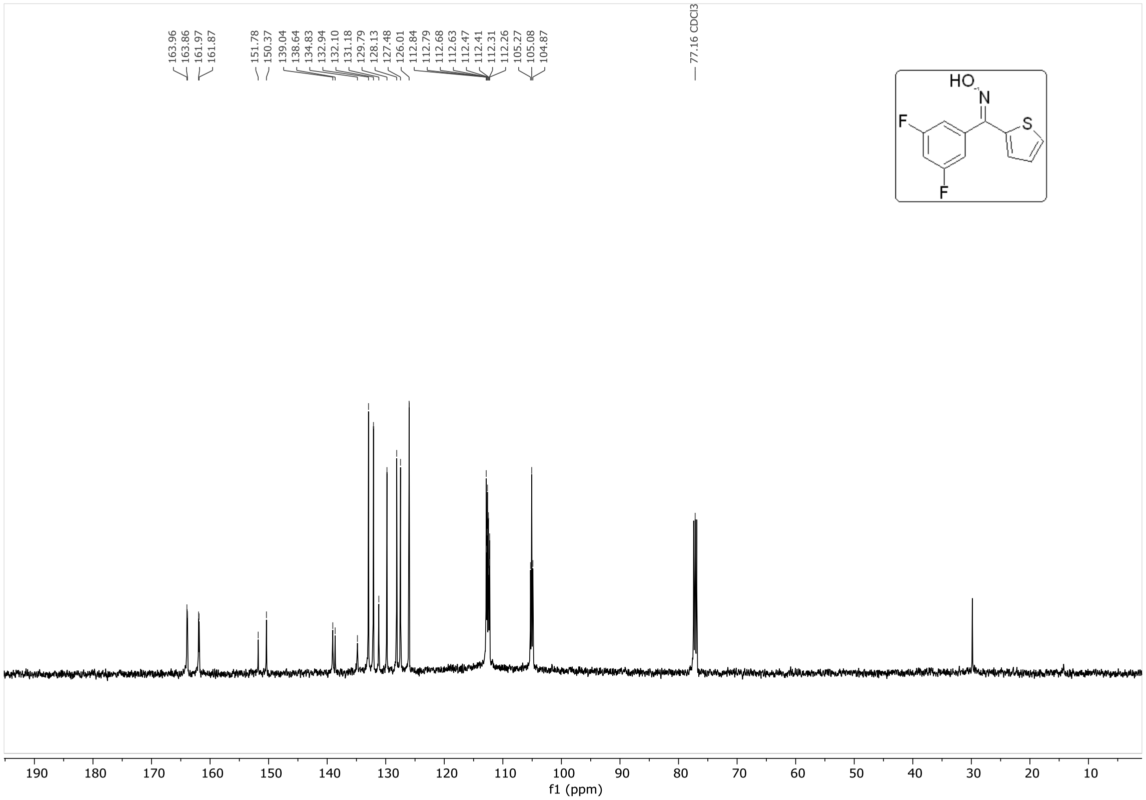

Figure NMR15. ^{1}H spectrum of **7b** in $CDCl_3$

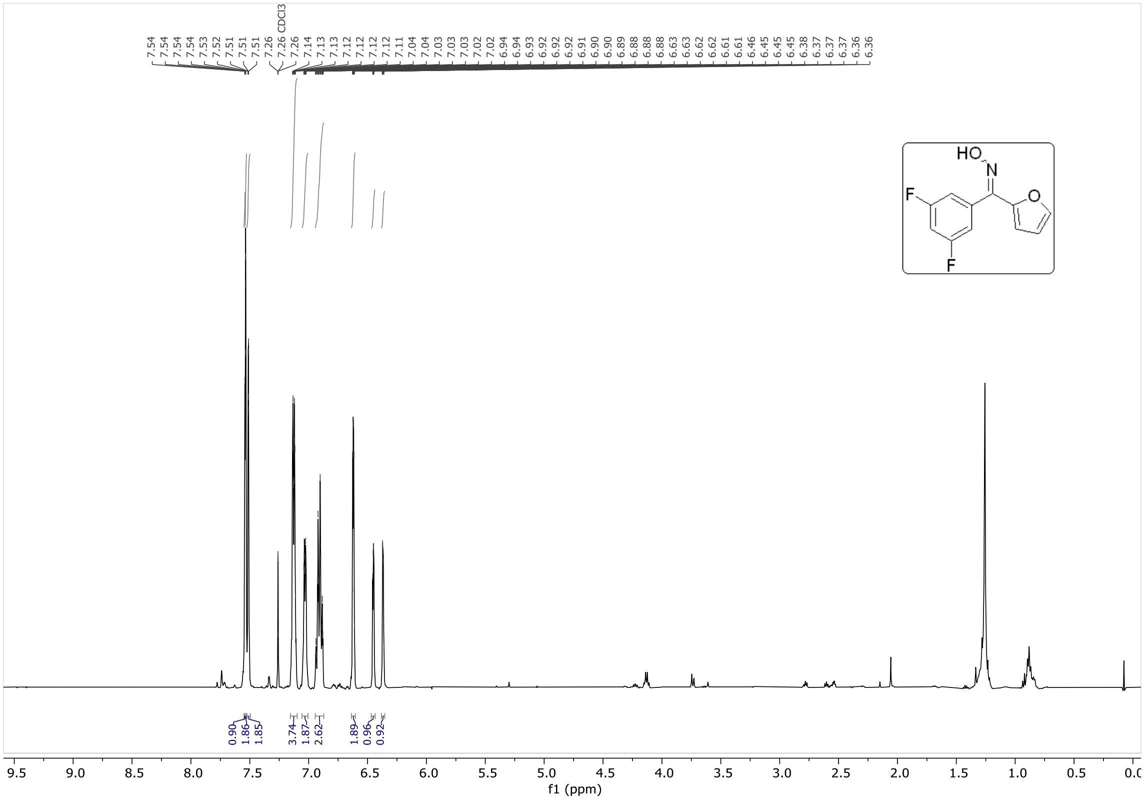

Figure NMR16. ^{13}C spectrum of **7b** in $CDCl_3$

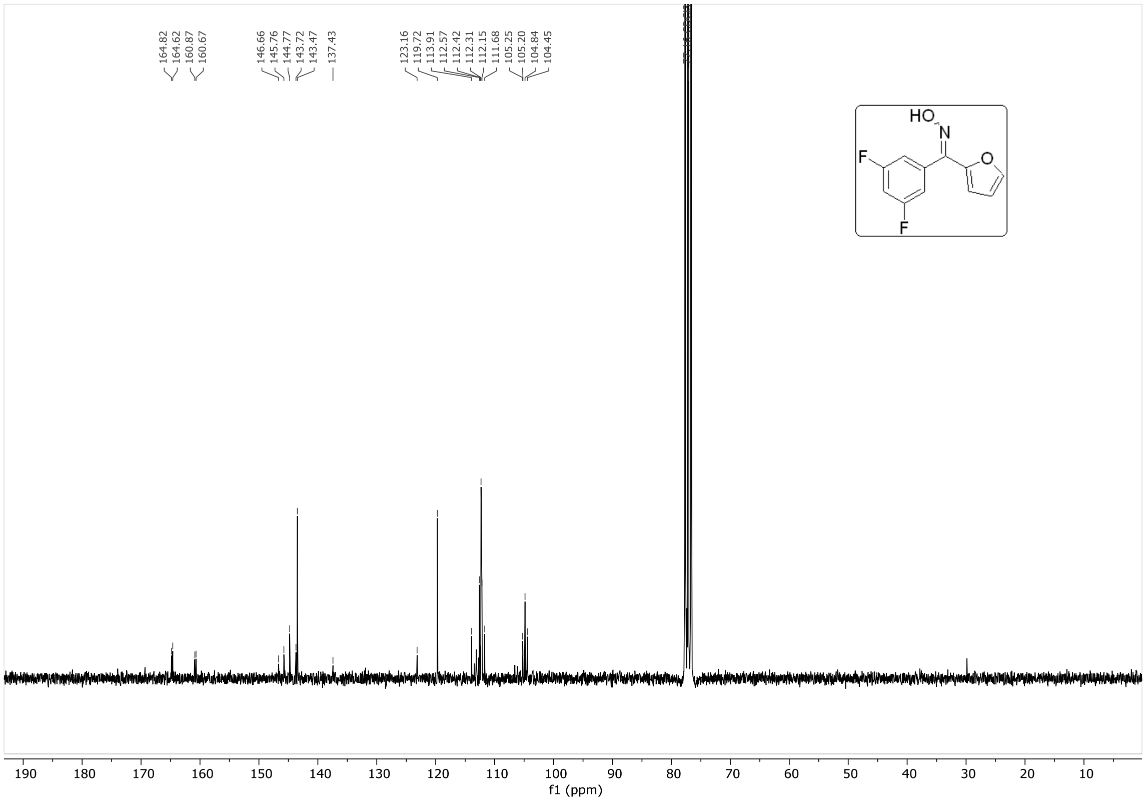

Figure NMR17. ^{1}H spectrum of **7d** in $CDCl_3$

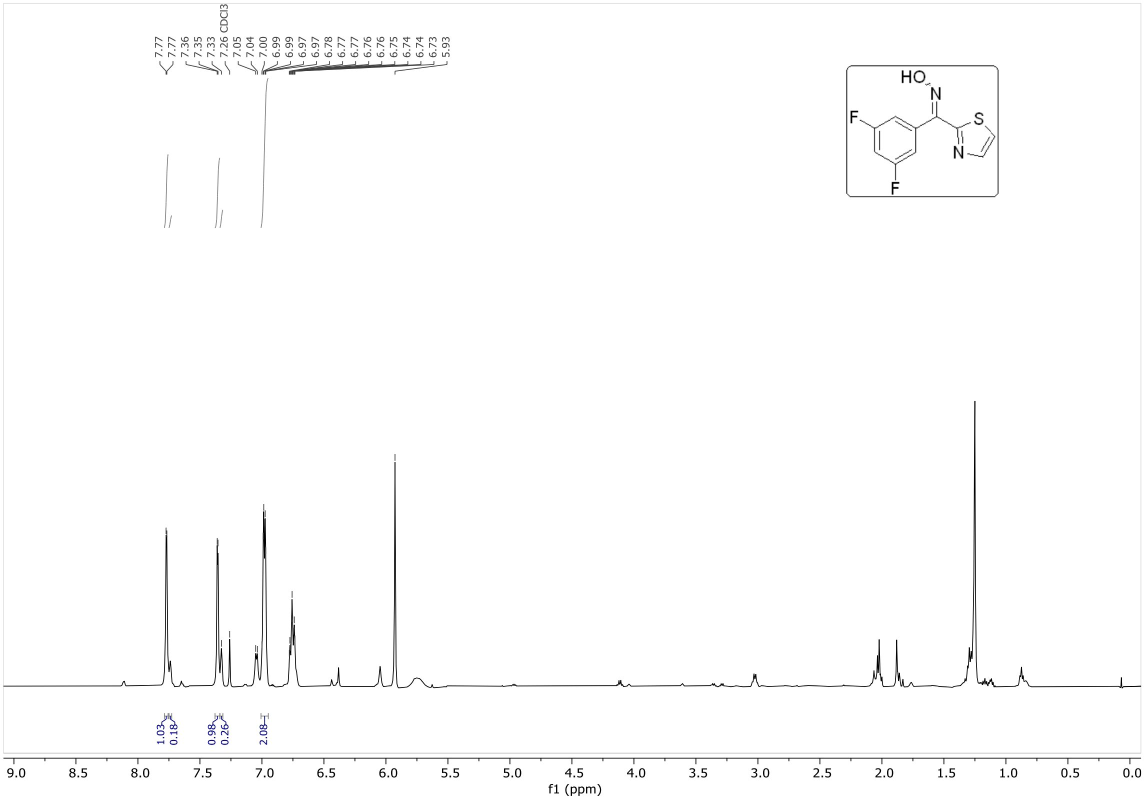

Figure NMR18. ^{13}C spectrum of **7d** in $CDCl_3$

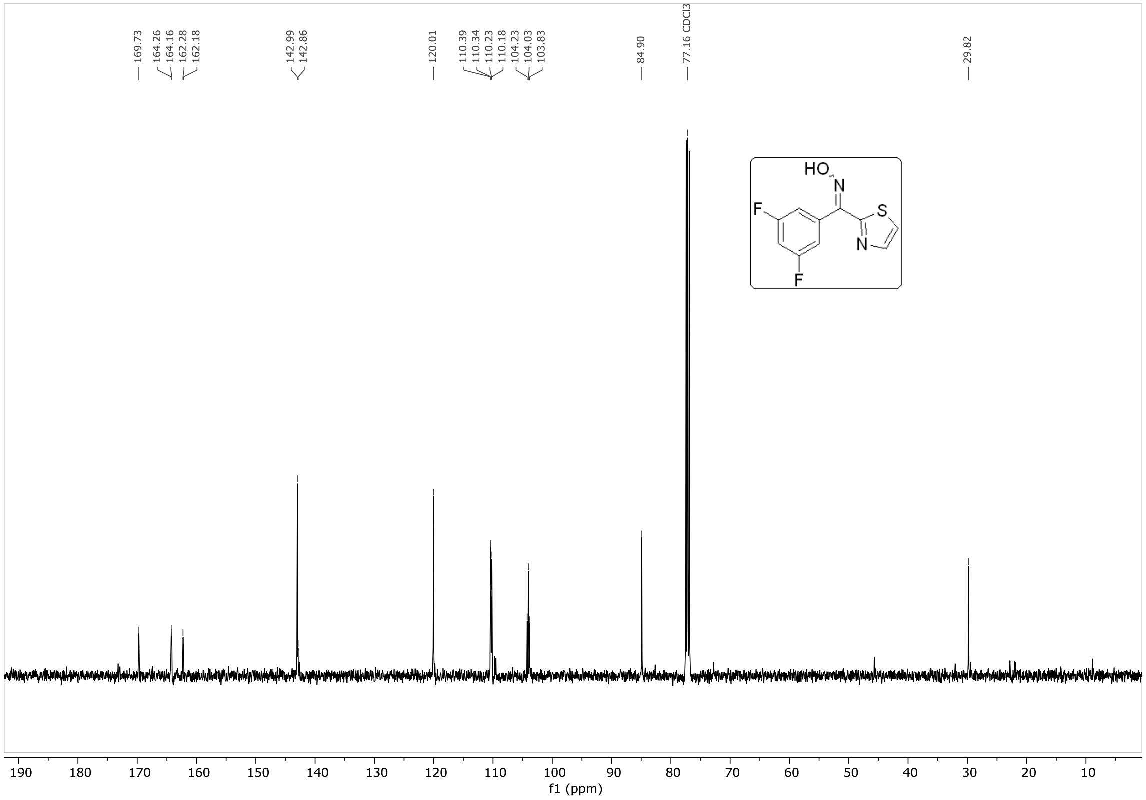

Figure NMR19. ^{1}H spectrum of **7f** in $CDCl_3$

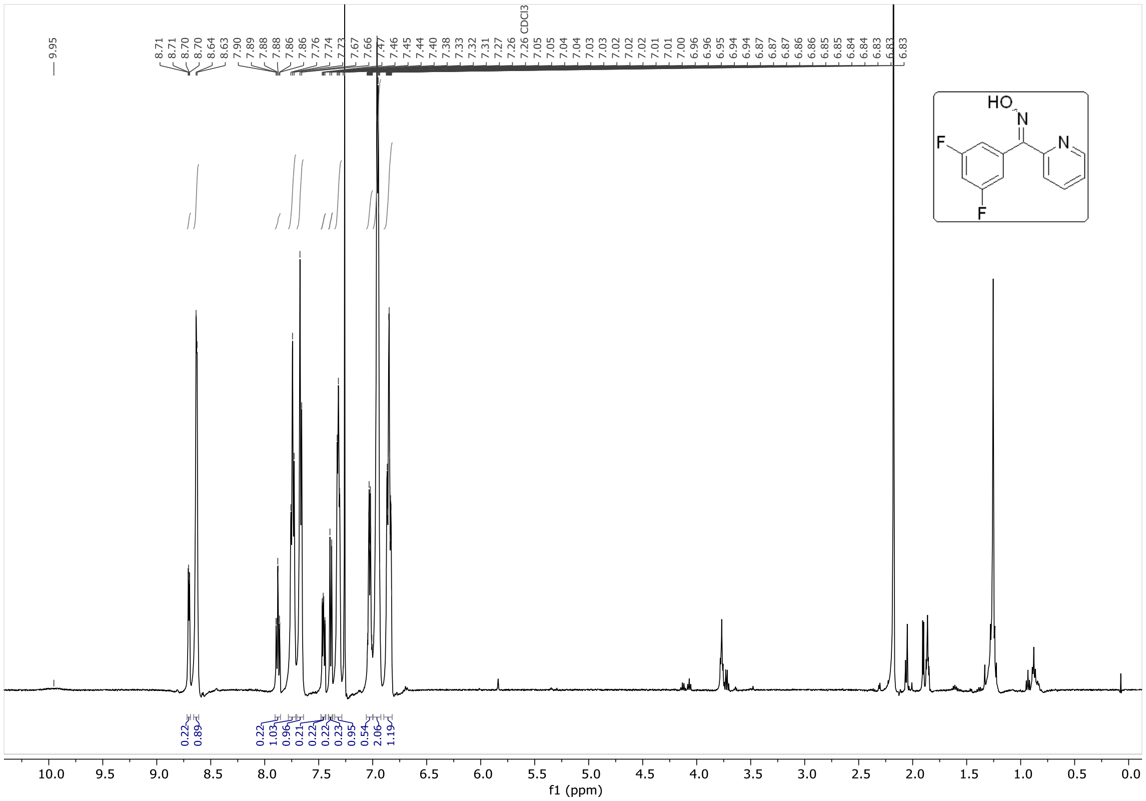

Figure NMR20. ^{13}C spectrum of **7f** in $CDCl_3$

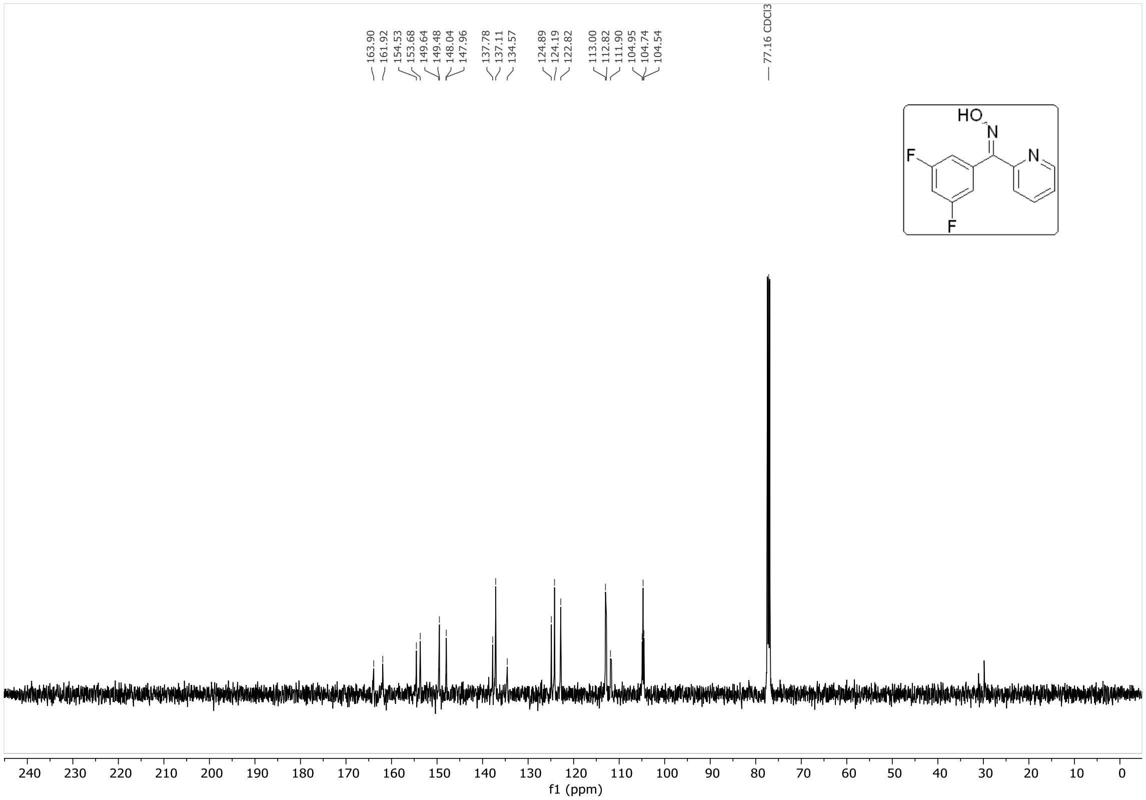

Figure NMR21. ^{1}H spectrum of **7h** in $CDCl_3$

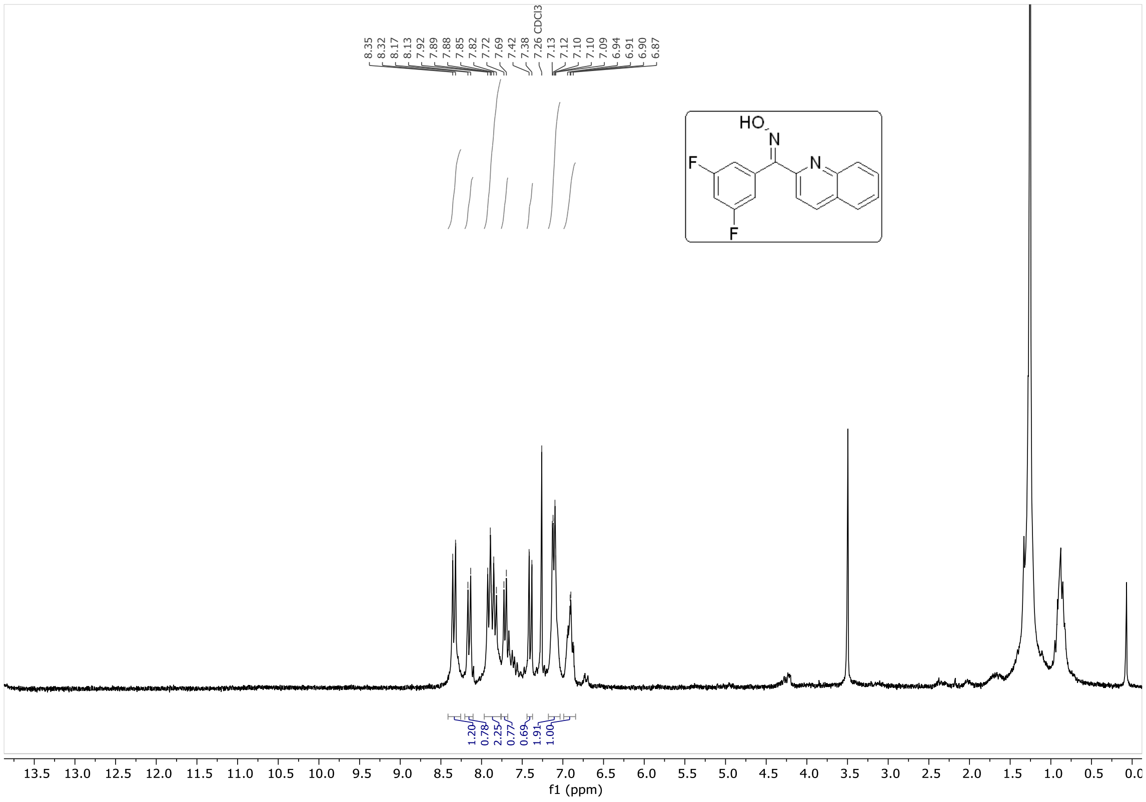

Figure NMR22. ^{13}C spectrum of **7h** in $CDCl_3$

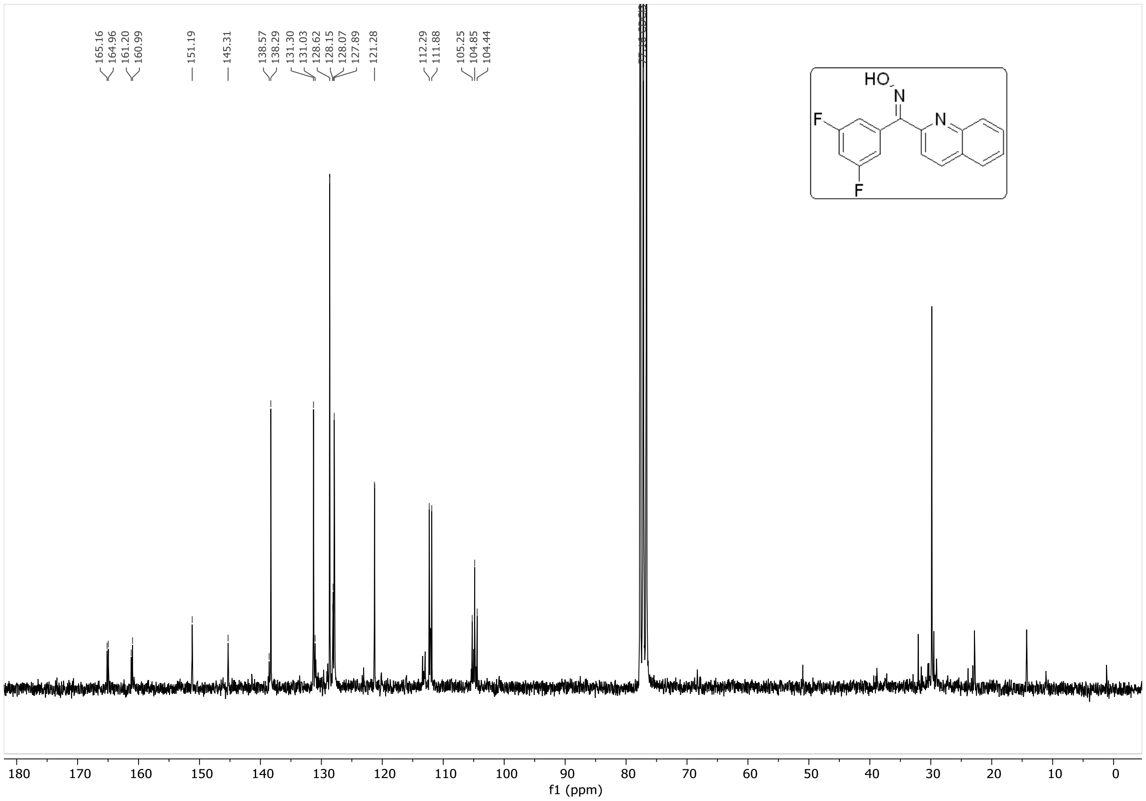

Figure NMR23. ^{1}H spectrum of **7i** in $CDCl_3$

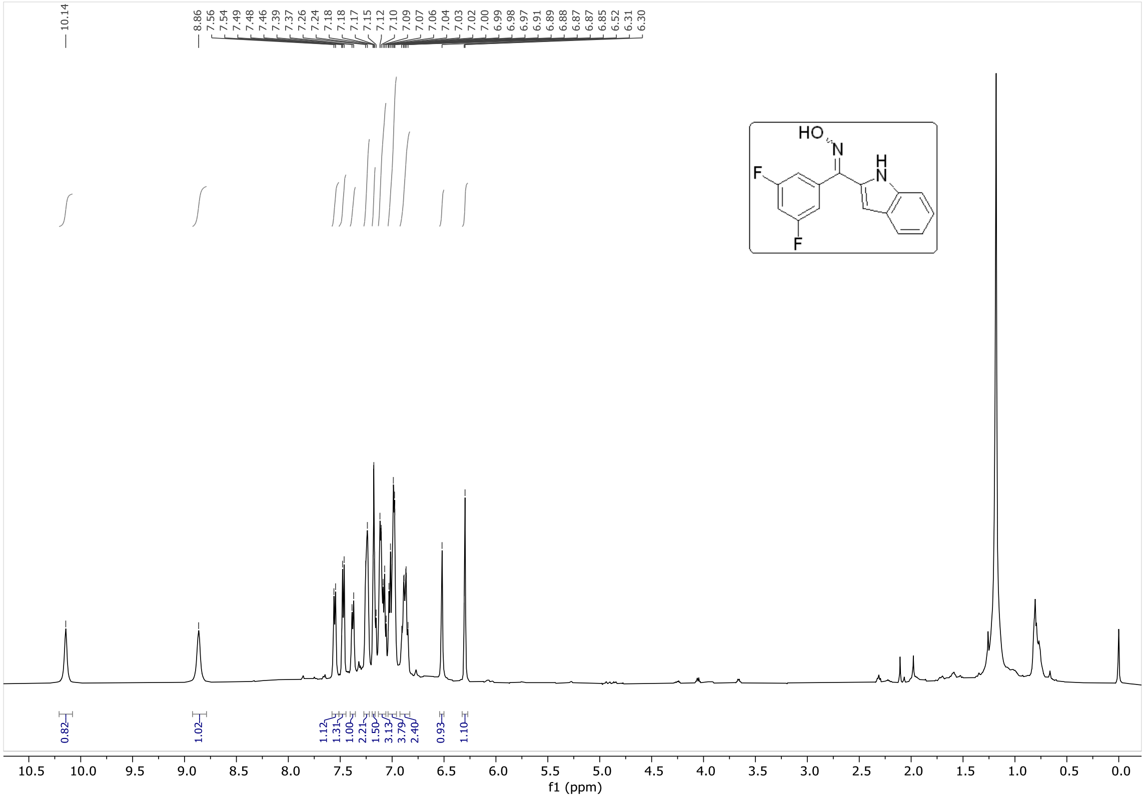

Figure NMR24. ^{13}C spectrum of **7i** in $CDCl_3$

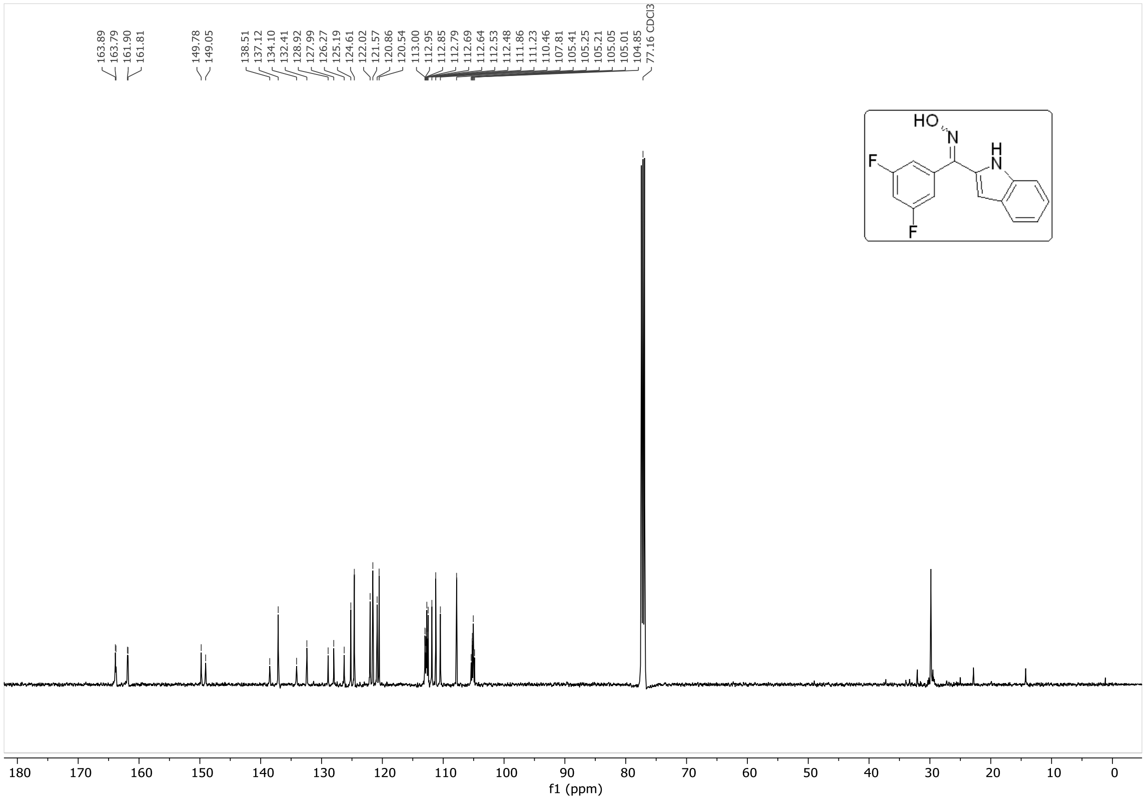

Figure NMR25. ^{1}H spectrum of **10a** in $CDCl_3$

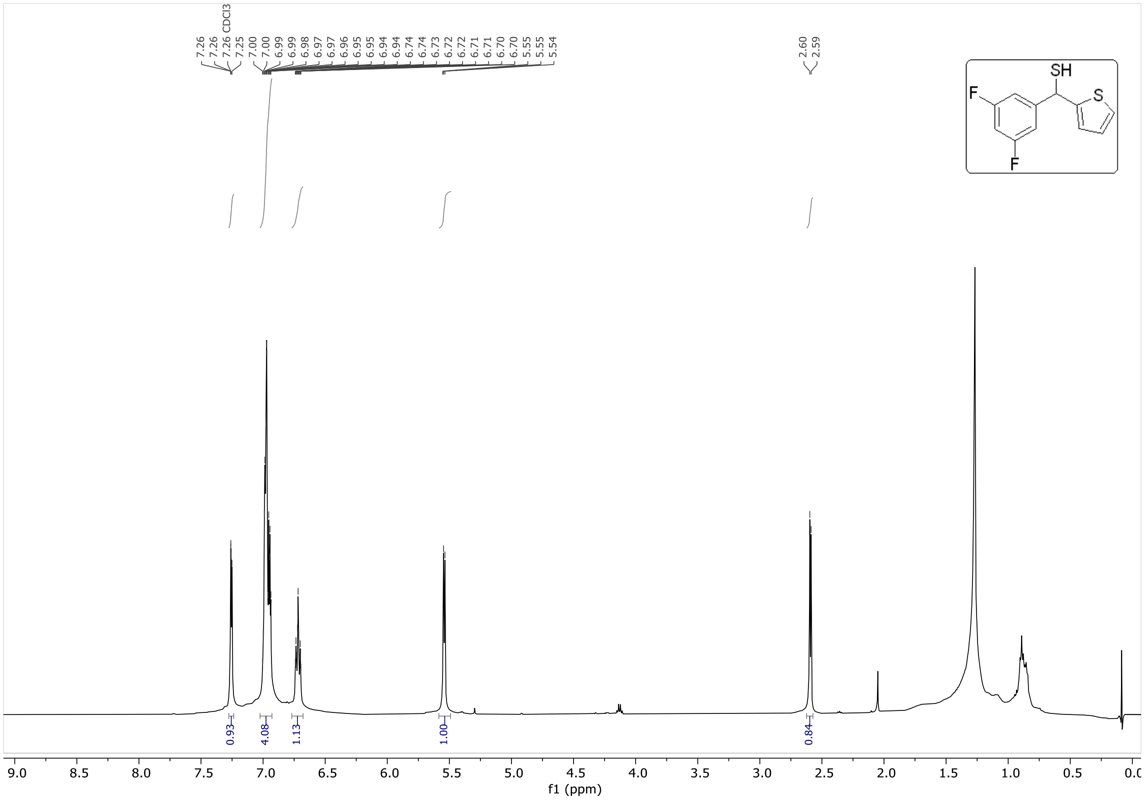

Figure NMR26. ^{13}C spectrum of **10a** in $CDCl_3$

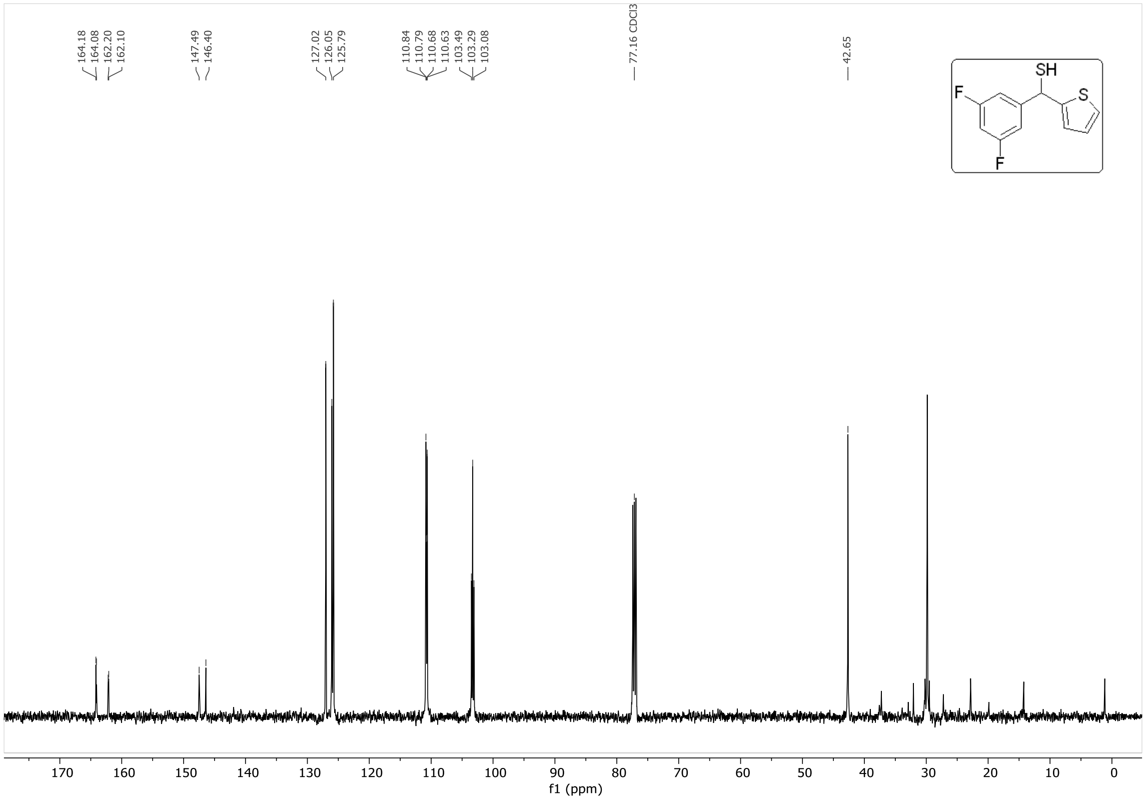

Figure NMR27. ^{1}H spectrum of **10b** in $CDCl_3$

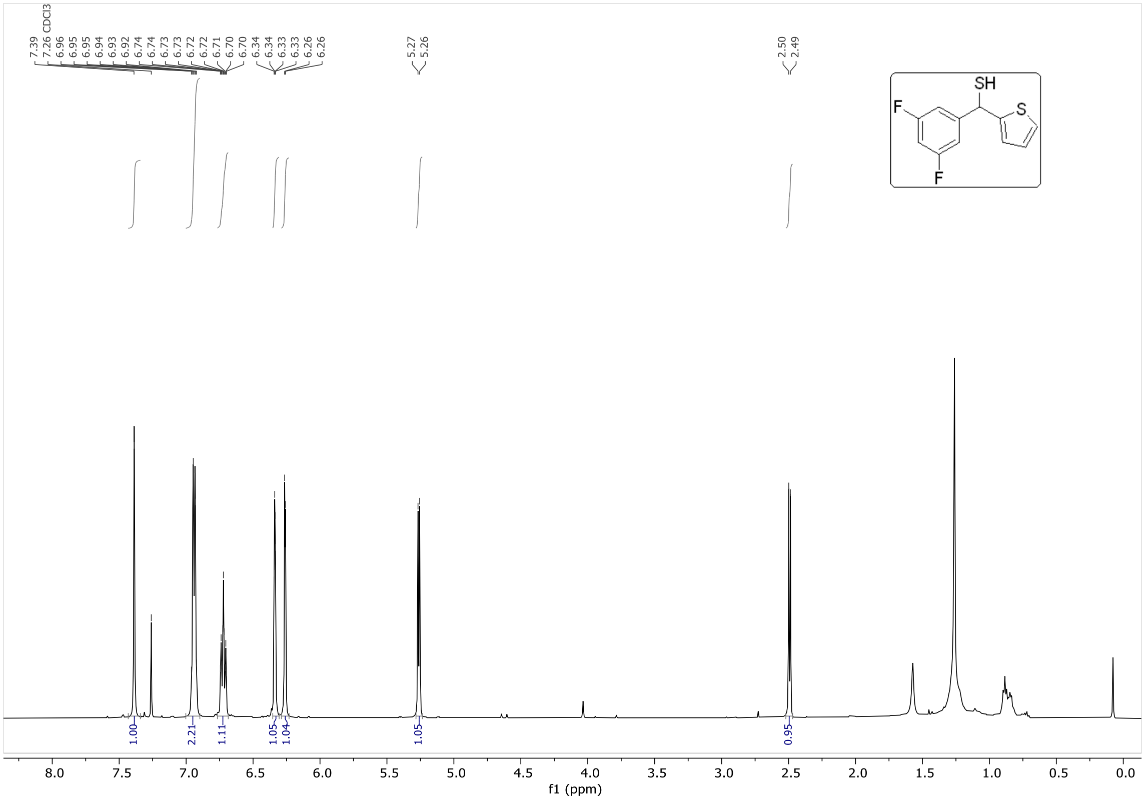

Figure NMR28. ^{13}C spectrum of **10b** in $CDCl_3$

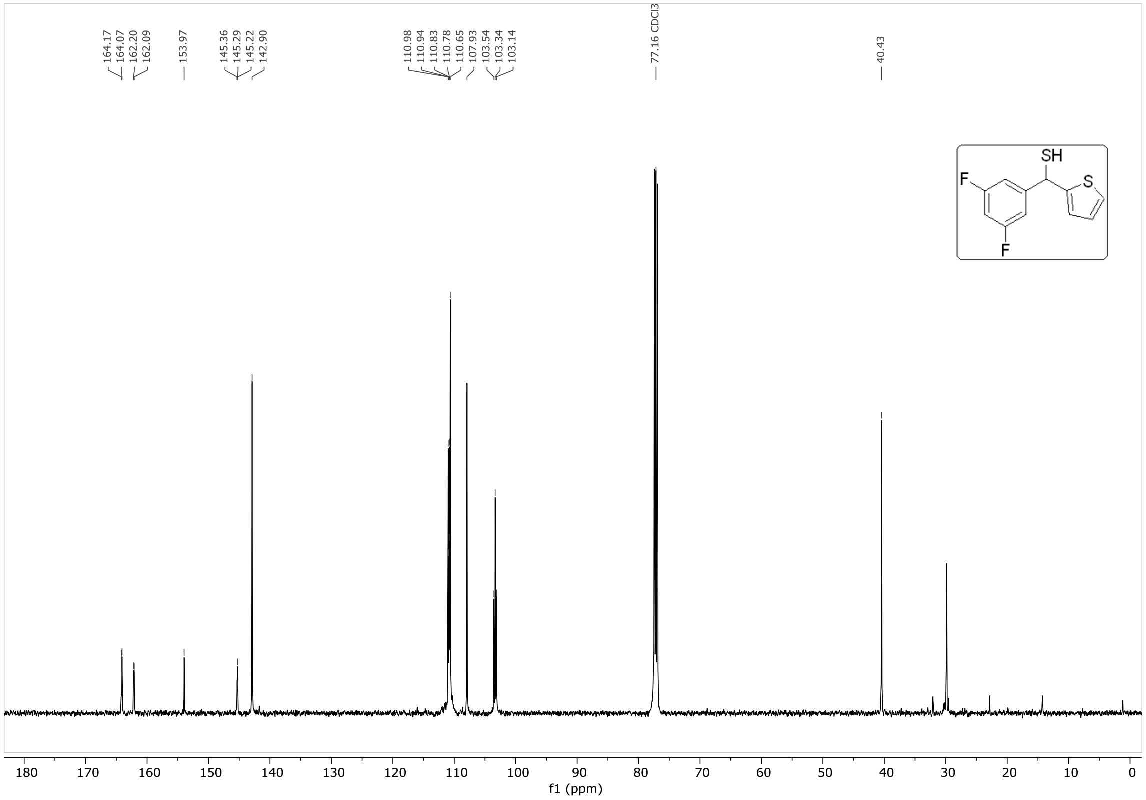

Figure NMR29. ^{1}H spectrum of **10d** in $CDCl_3$

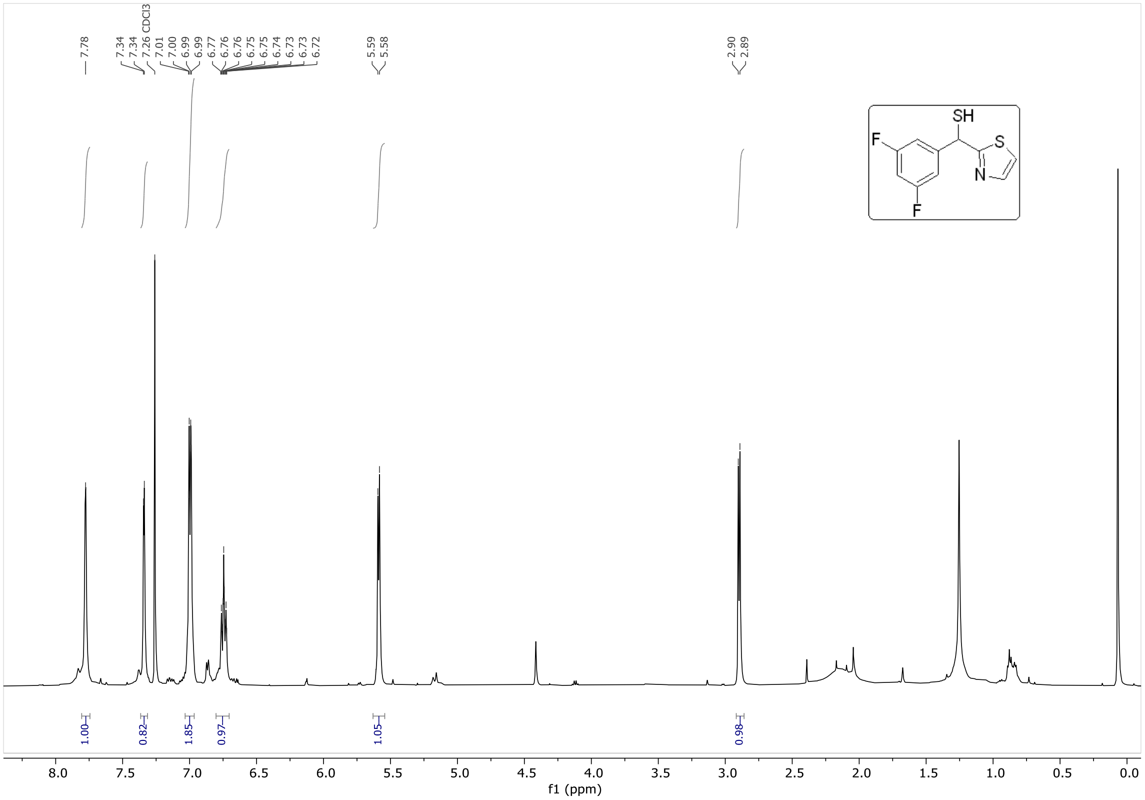

Figure NMR30. ^{13}C spectrum of **10d** in $CDCl_3$

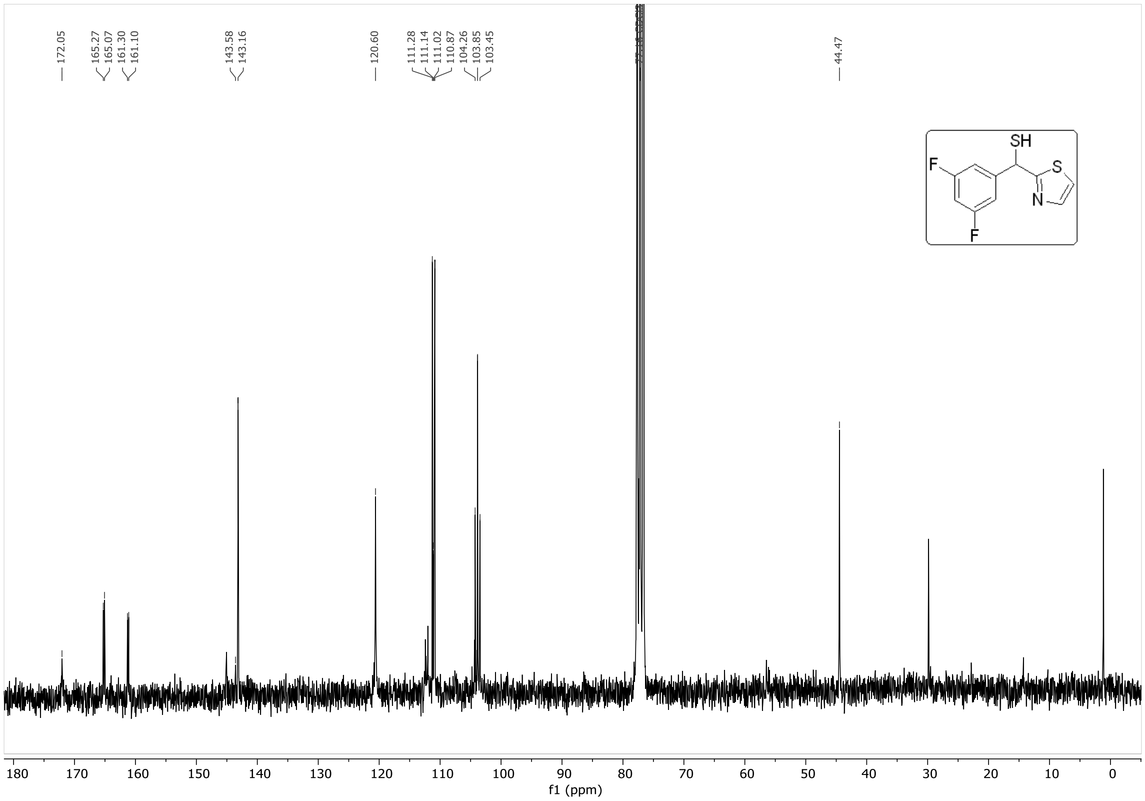

Figure NMR31. ^{1}H spectrum of **10f** in $CDCl_3$

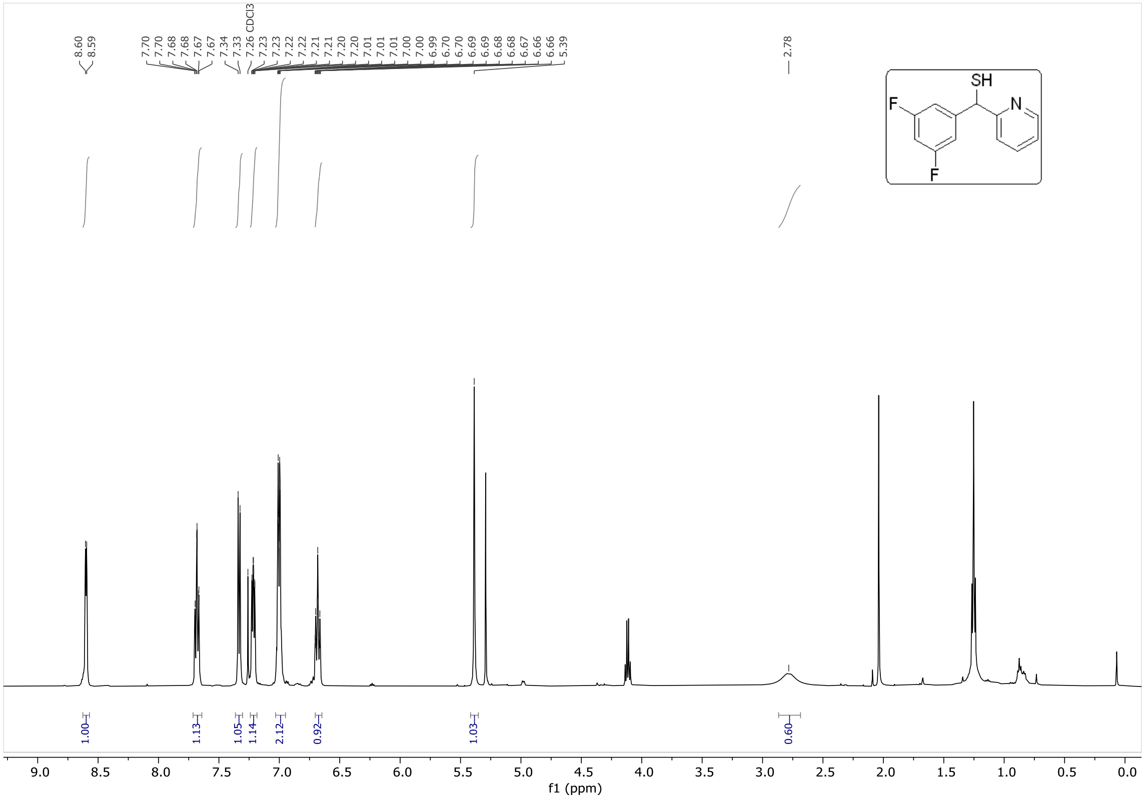

Figure NMR32. ^{13}C spectrum of **10f** in $CDCl_3$

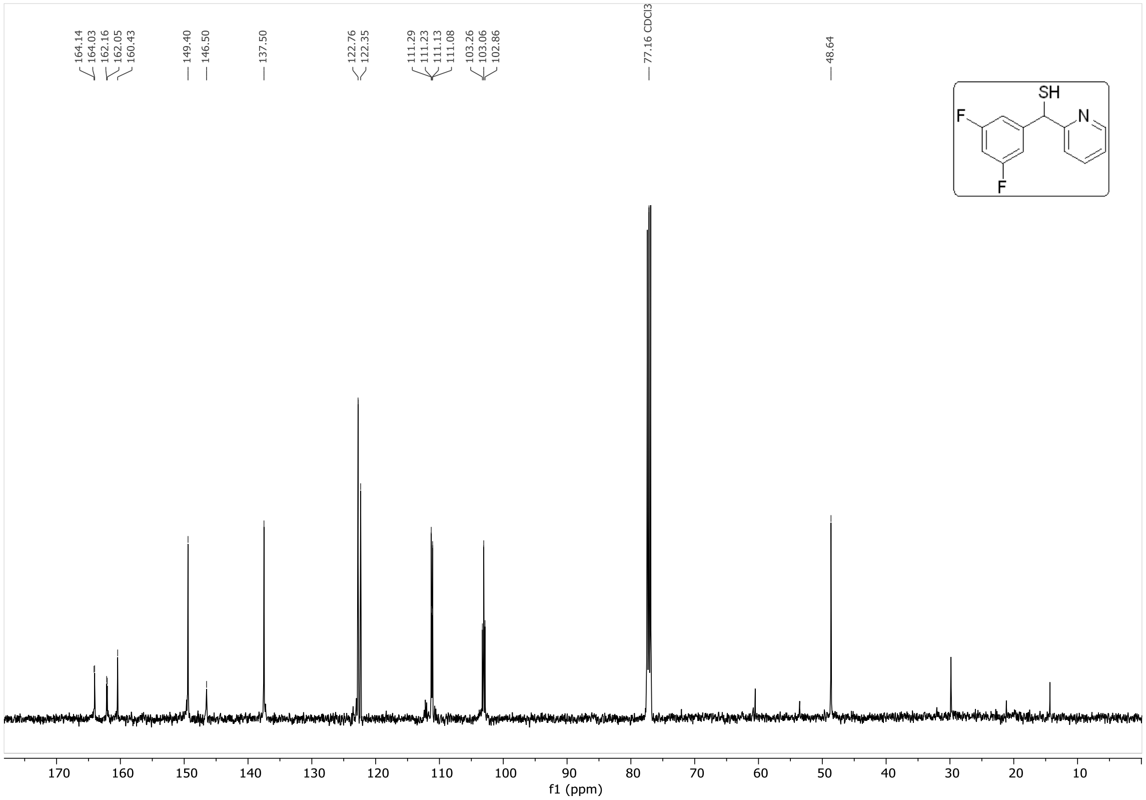

Figure NMR33. ^{1}H spectrum of **10i** in $CDCl_3$

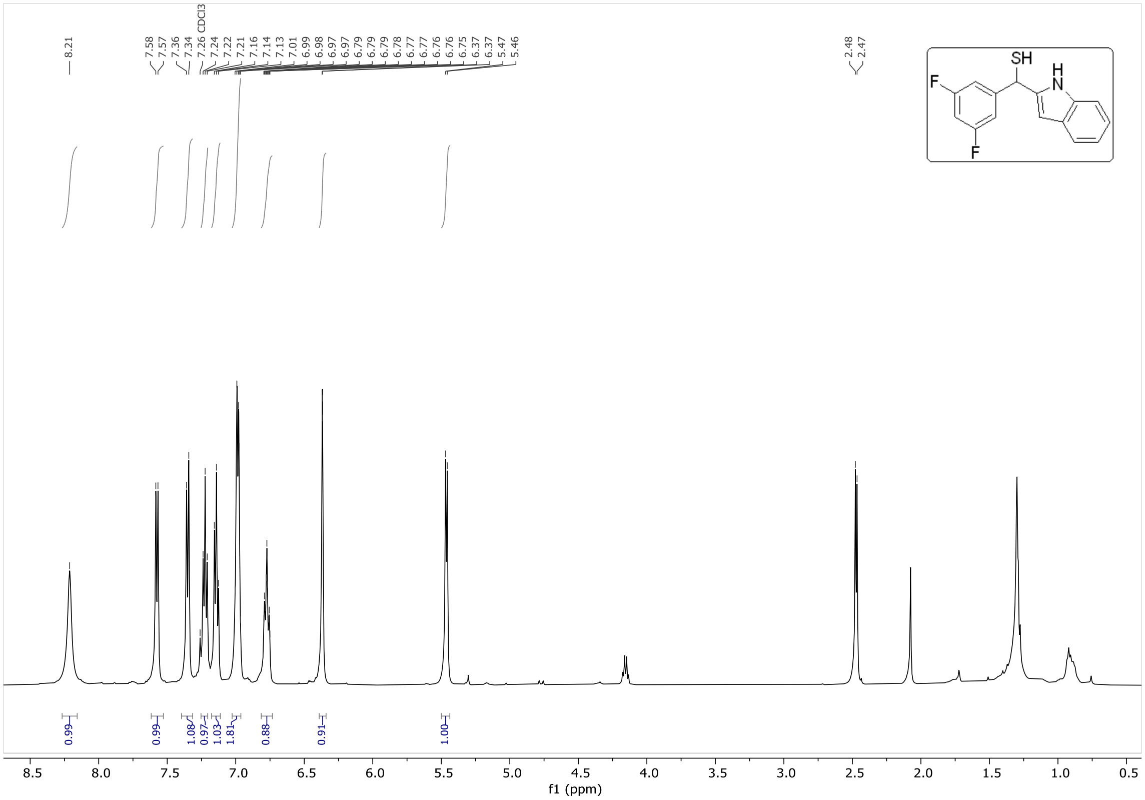

Figure NMR34. ^{13}C spectrum of **10i** in $CDCl_3$

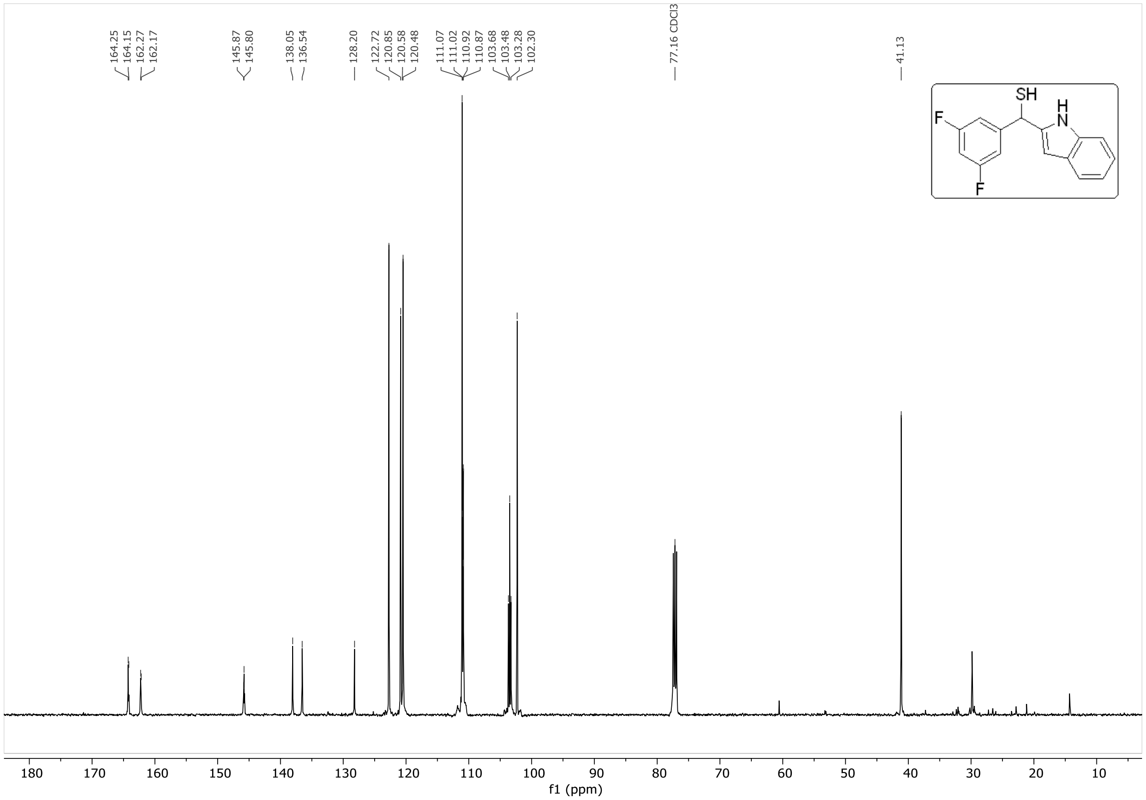

Figure NMR35. ^{1}H spectrum of **19a** in DMSO-d_6

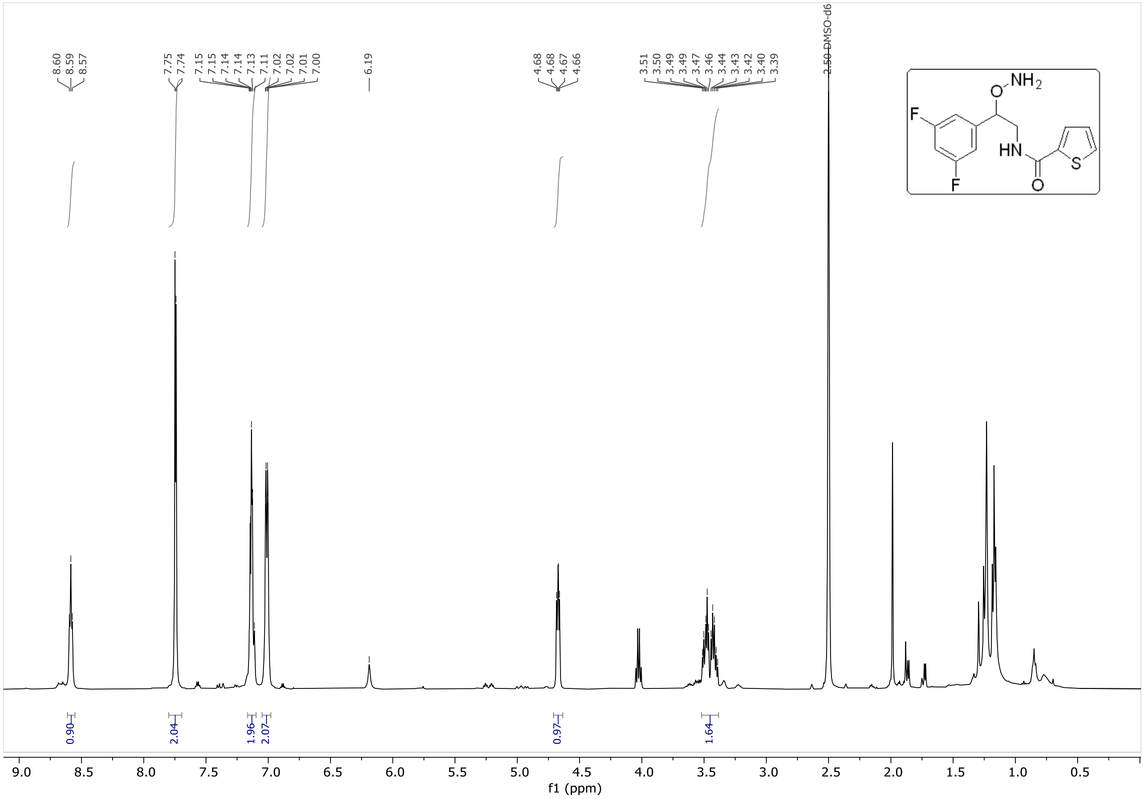

Figure NMR36. ^{13}C spectrum of **19a** in DMSO-d_6

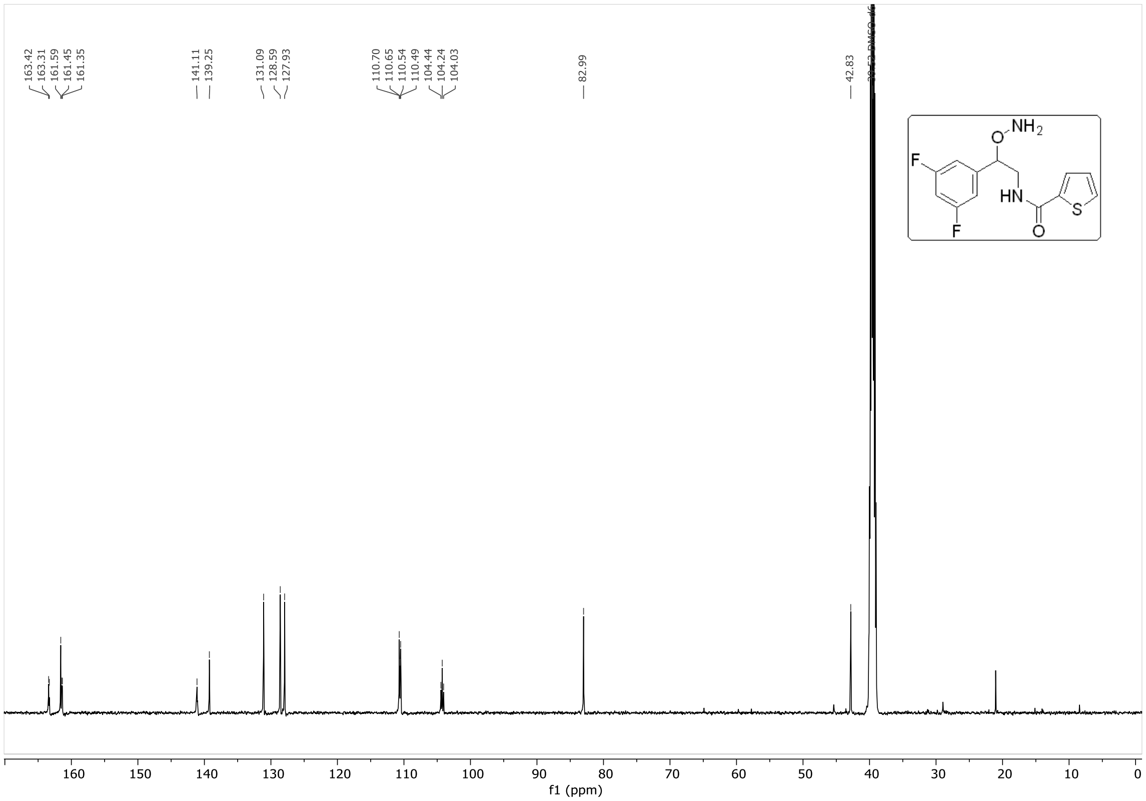

Figure NMR37. ^{1}H spectrum of **19c** in DMSO-d$_6$

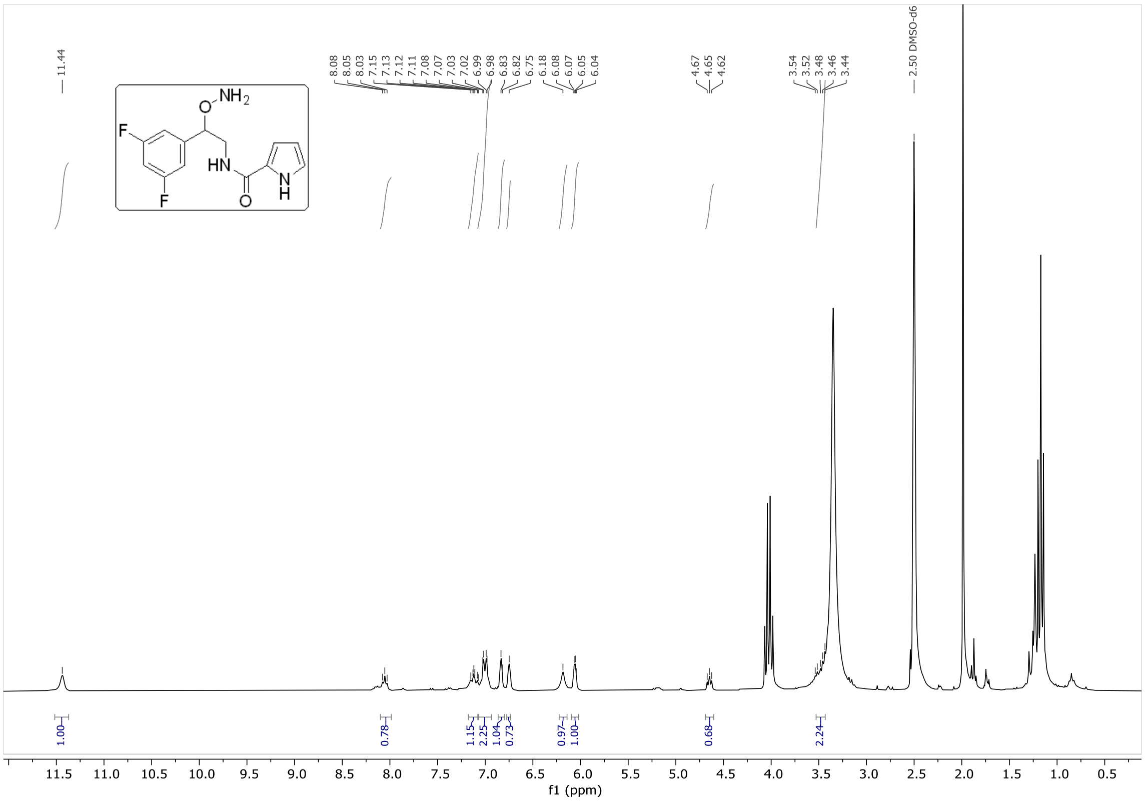

Figure NMR38. ^{13}C spectrum of **19c** in DMSO-d$_6$

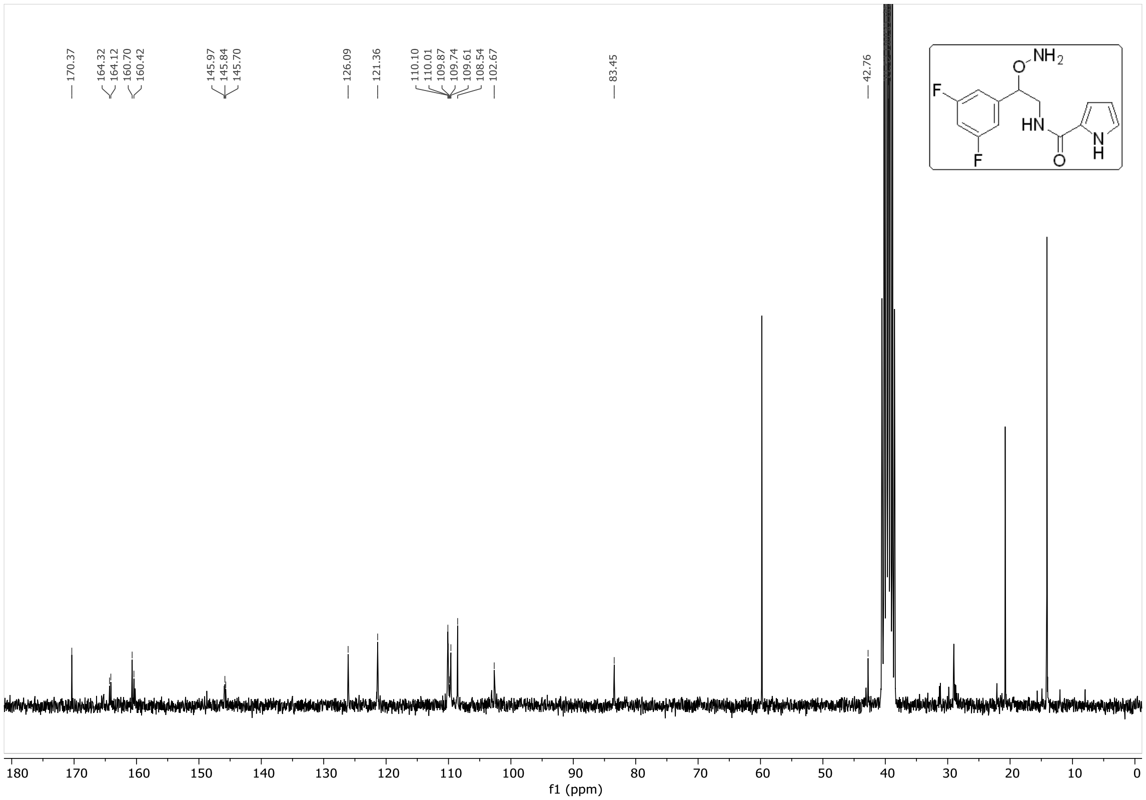

Figure NMR39. ^{1}H spectrum of **19e** in DMSO-d_6

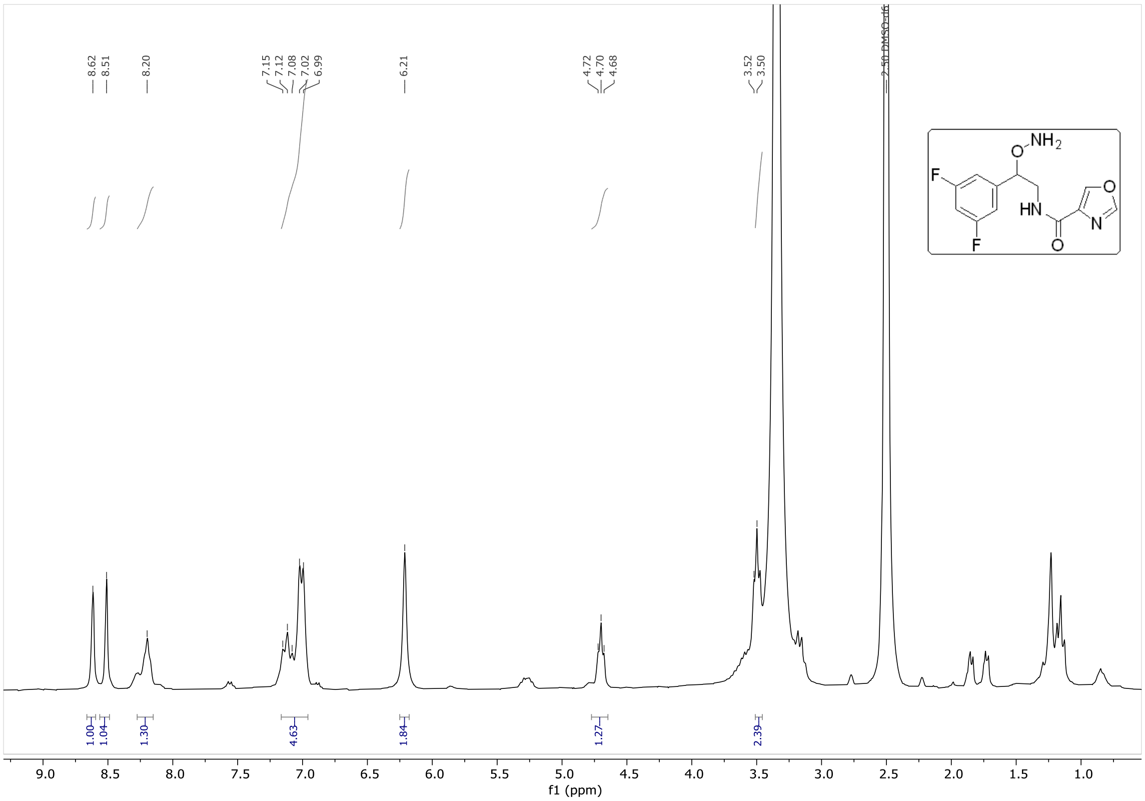

Figure NMR40. ^{13}C spectrum of **19e** in DMSO-d_6

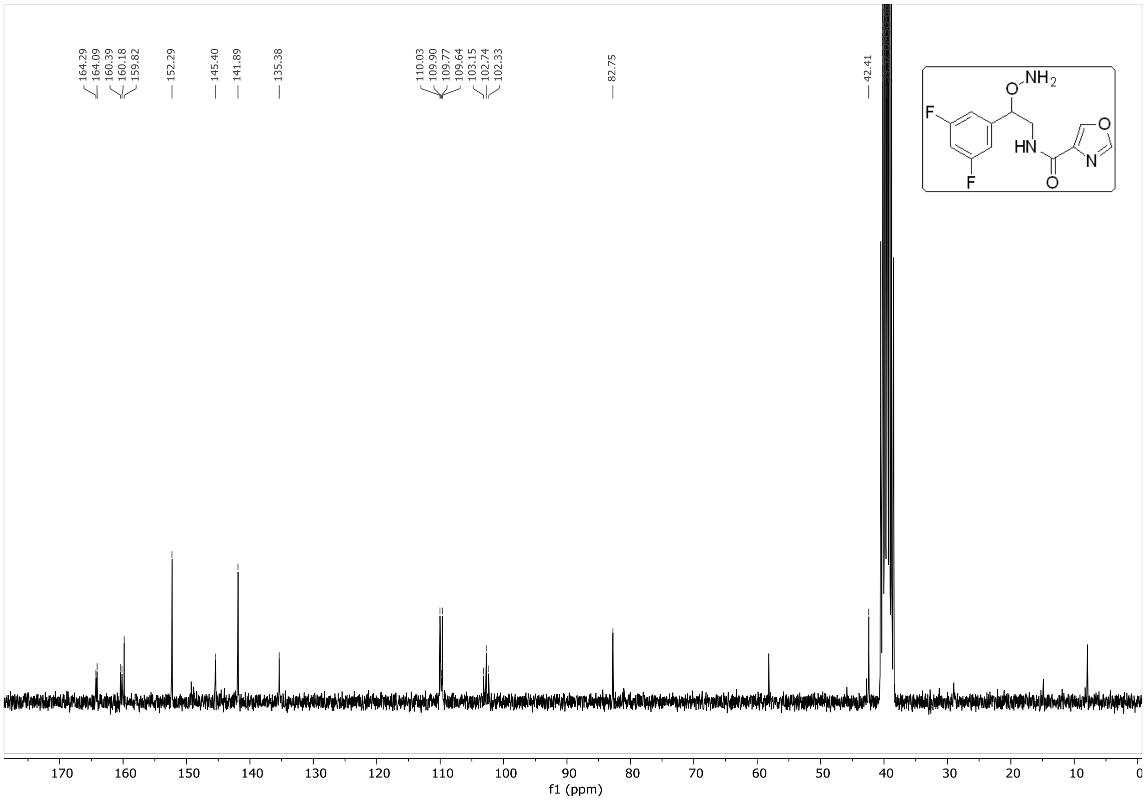

Figure NMR41. ^{1}H spectrum of **19f** in DMSO-d$_6$

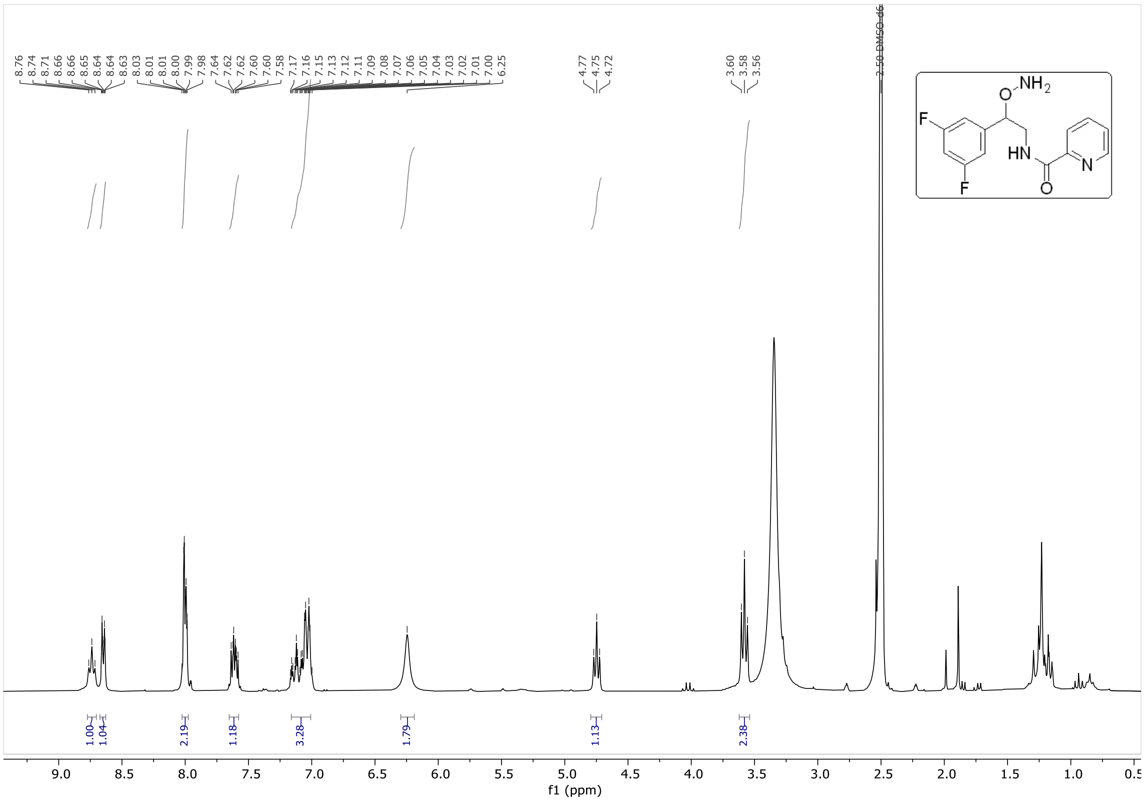

Figure NMR42. ^{13}C spectrum of **19f** in DMSO-d$_6$

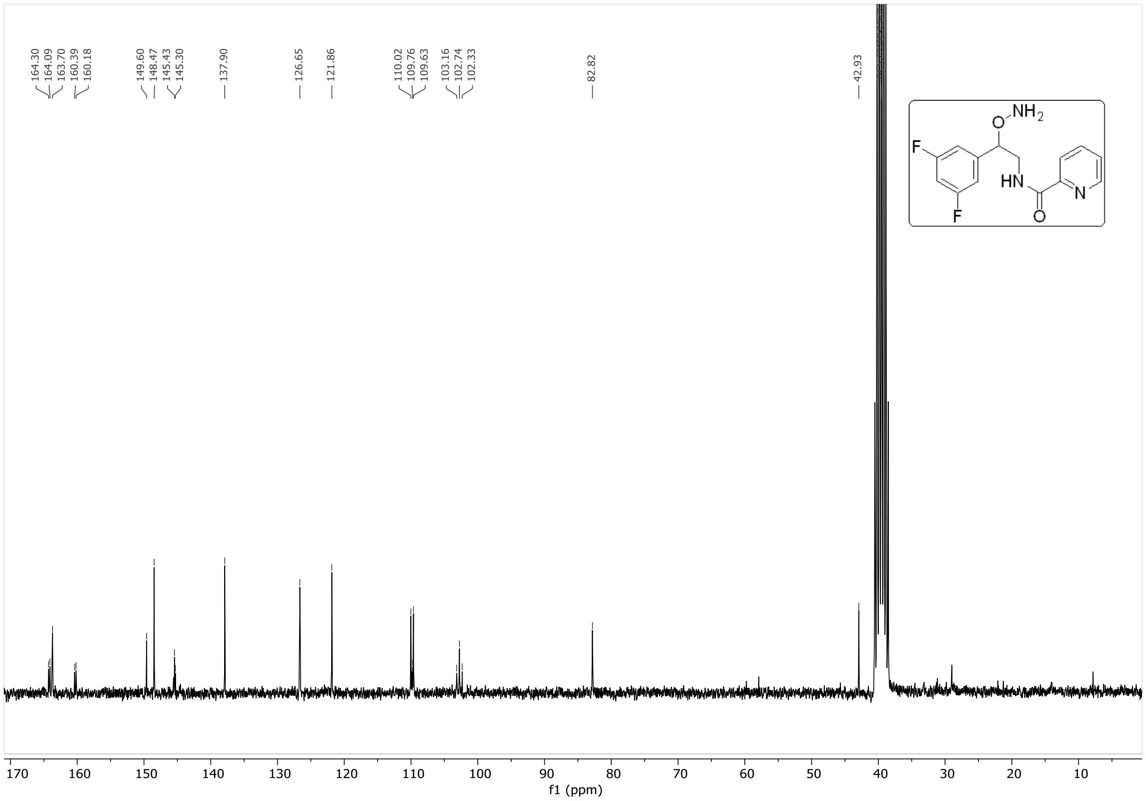

Figure NMR41. ^{1}H spectrum of **19g** in DMSO-d_6

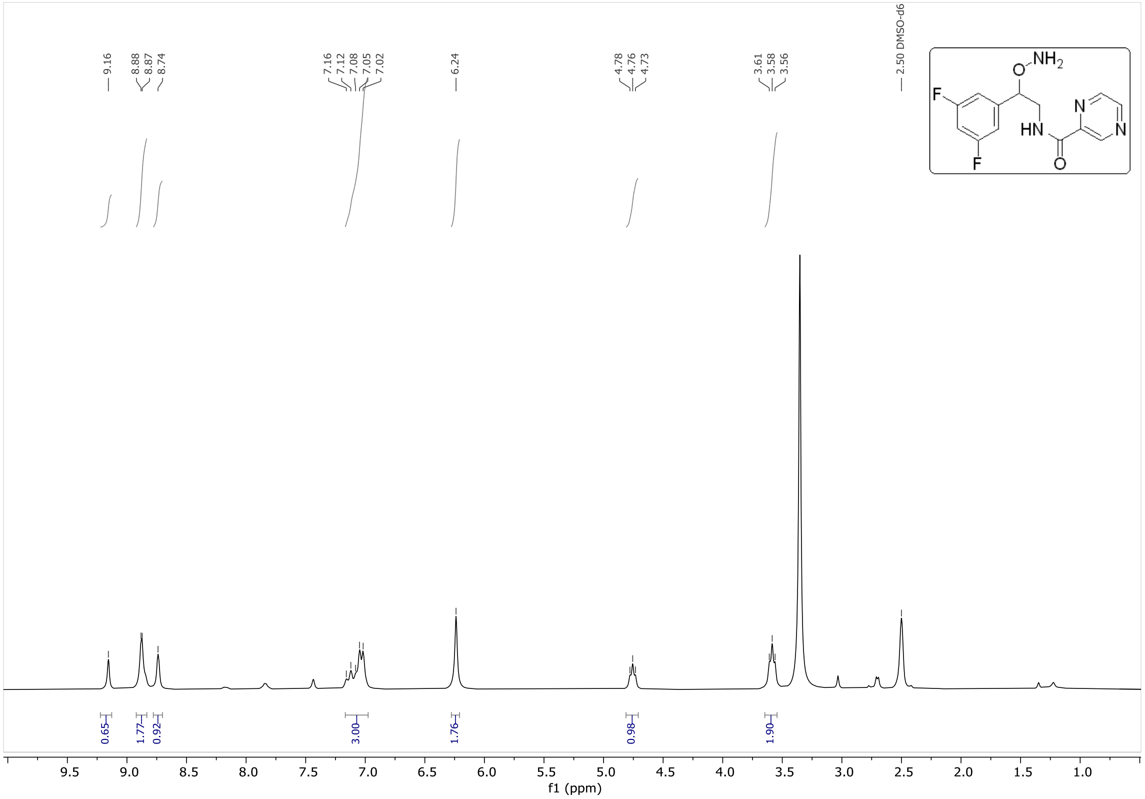

Figure NMR42. ^{13}C spectrum of **19g** in DMSO-d_6

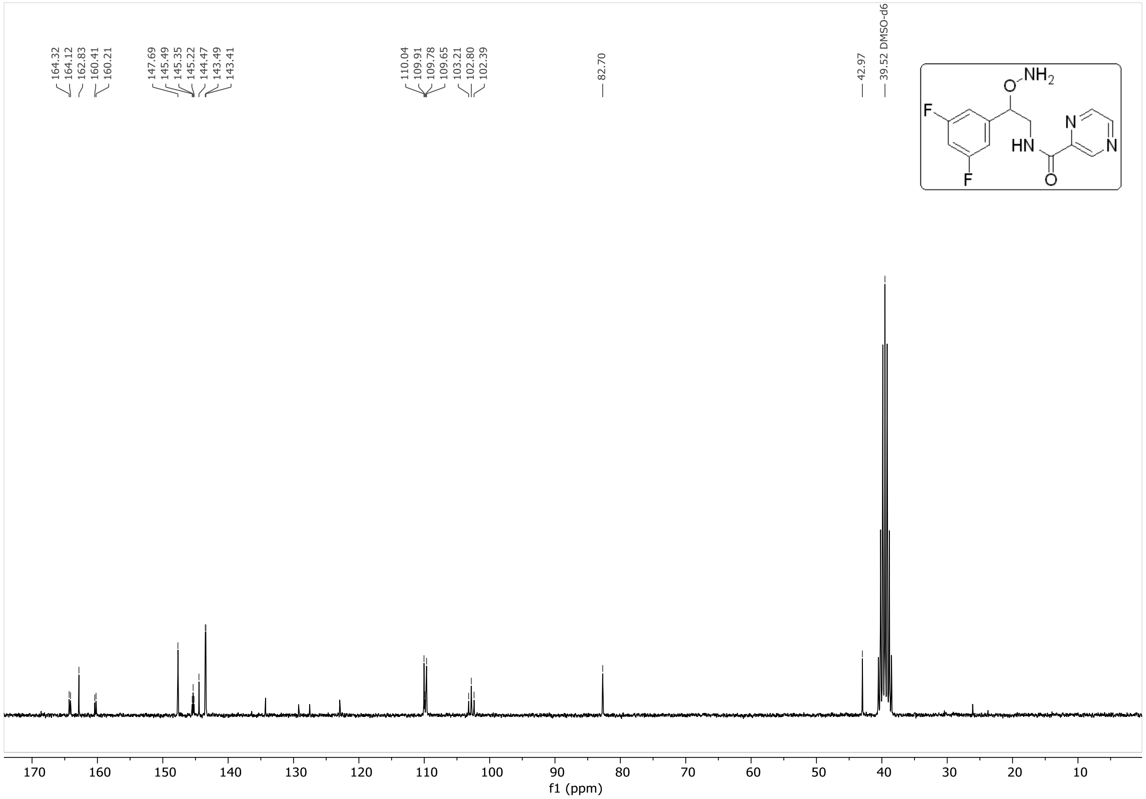

Figure NMR41. ^{1}H spectrum of **19i** in DMSO-d6

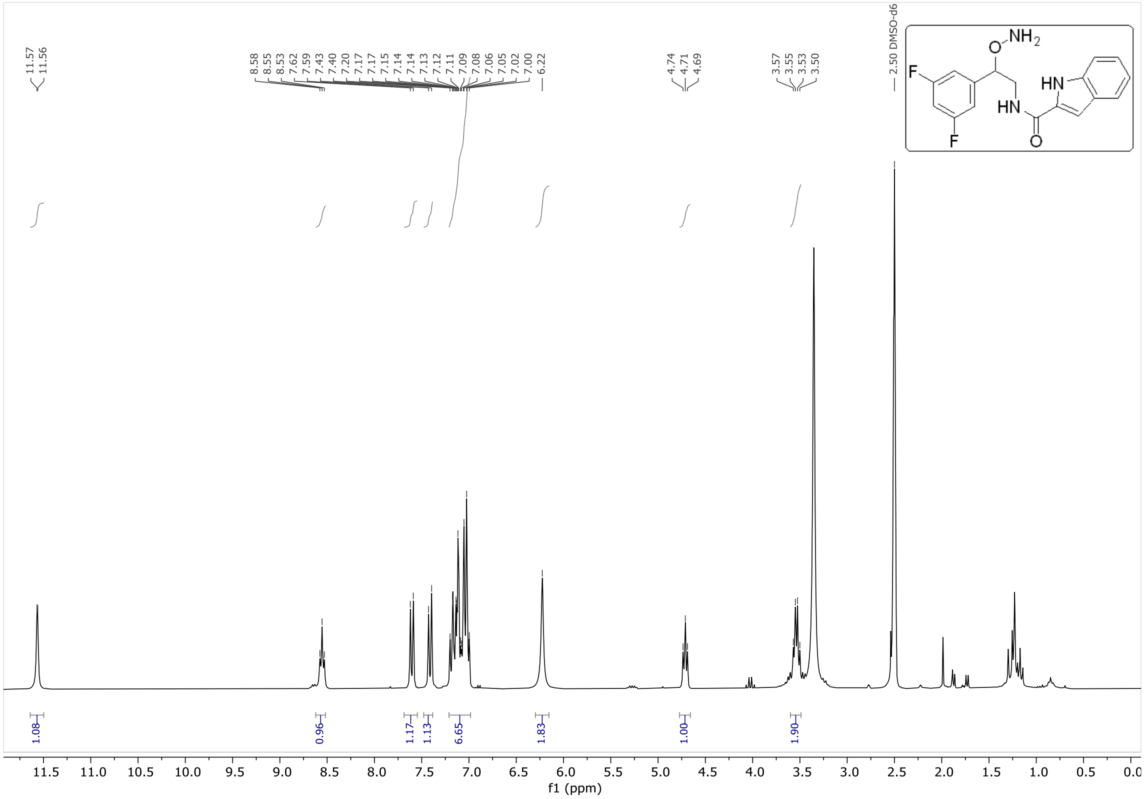

Figure NMR42. ^{13}C spectrum of **19i** in DMSO-d6

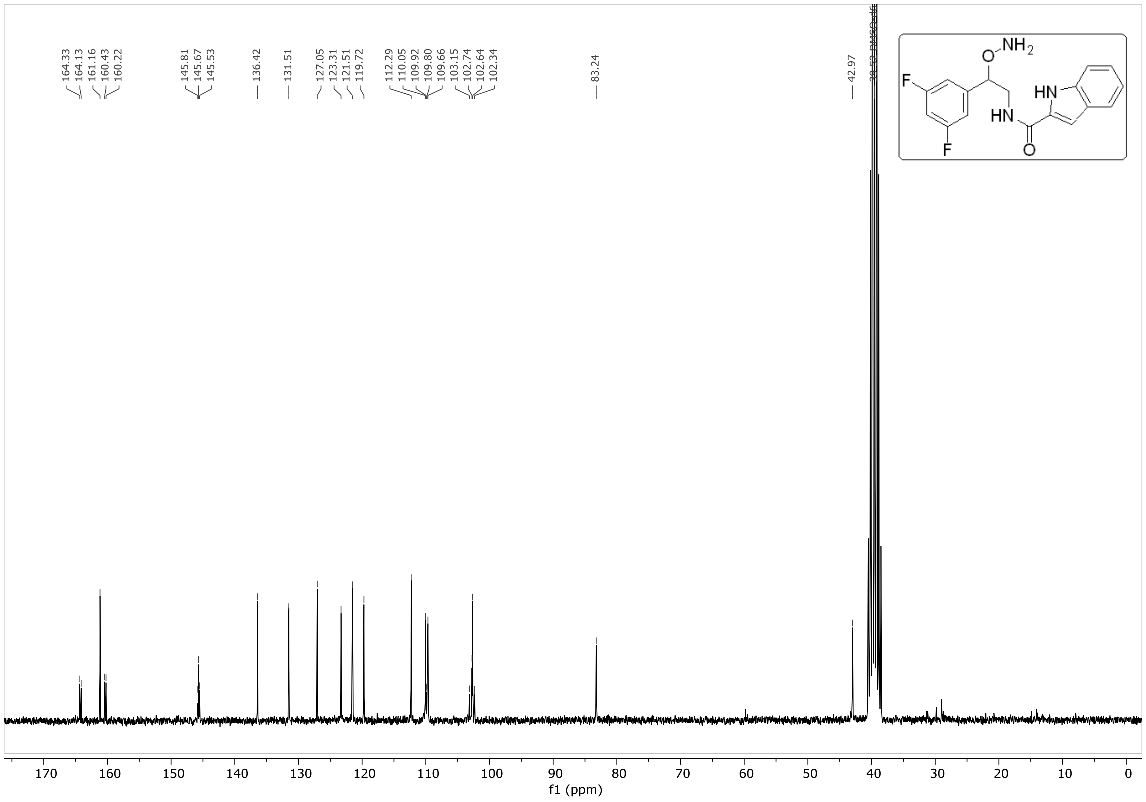

Figure NMR43. ^{1}H spectrum of **25a** in $CDCl_3$

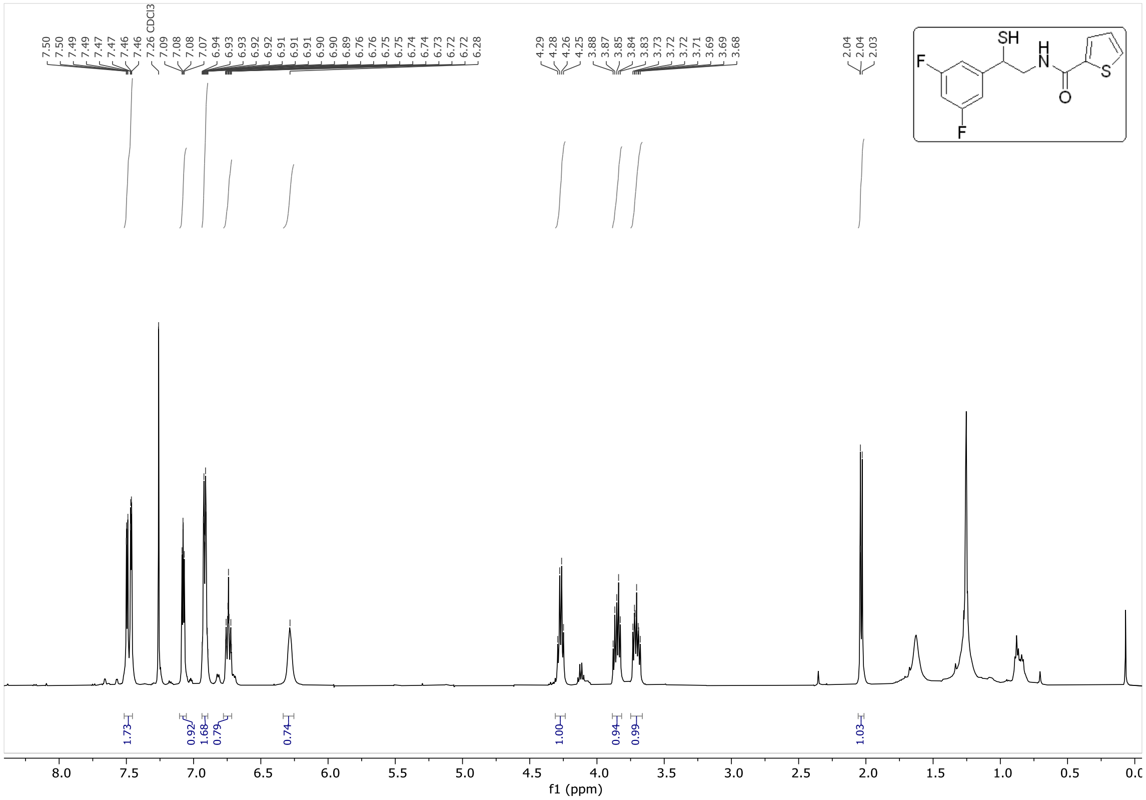

Figure NMR44. ^{13}C spectrum of **25a** in $CDCl_3$

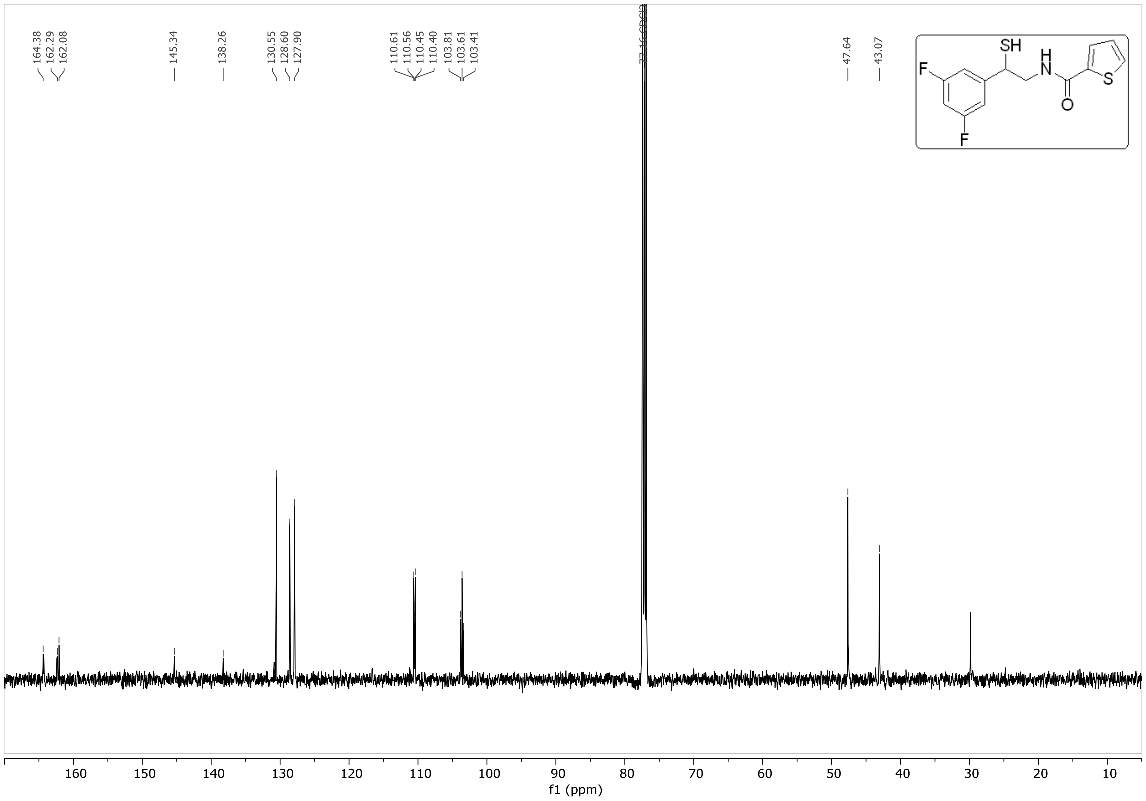

Figure NMR45. ^{1}H spectrum of **25b** in $CDCl_3$

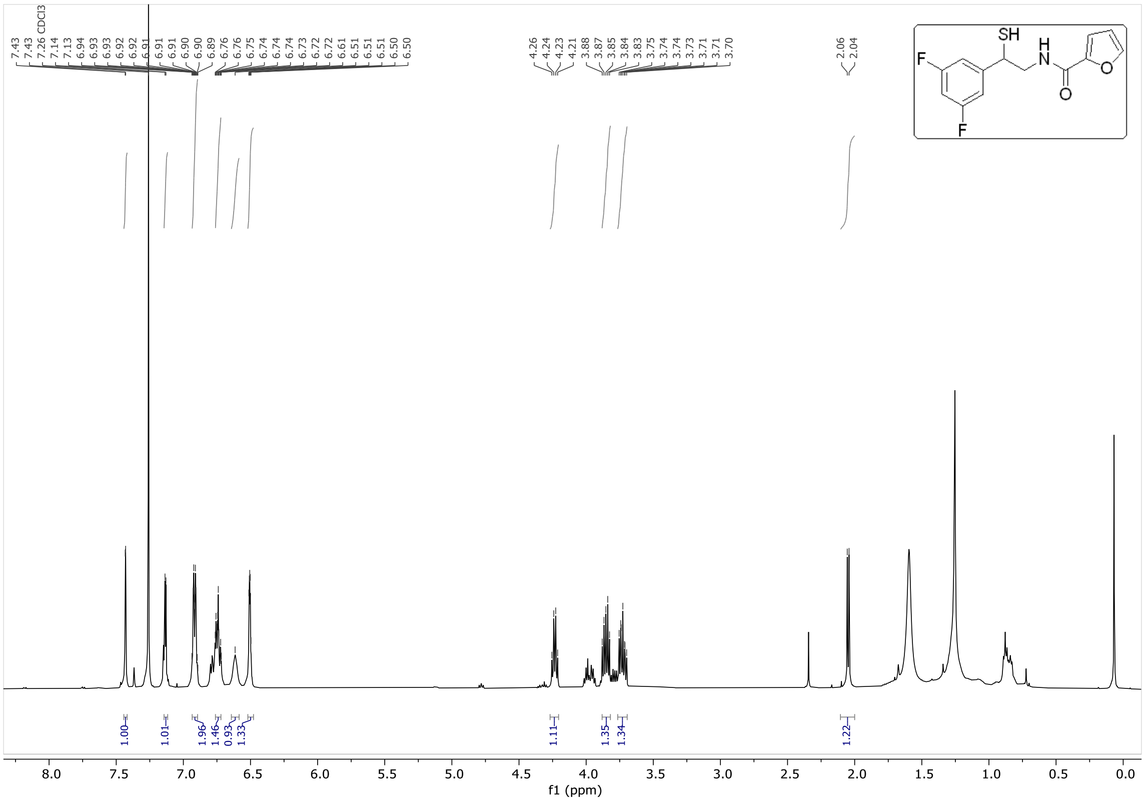

Figure NMR46. ^{13}C spectrum of **25b** in $CDCl_3$

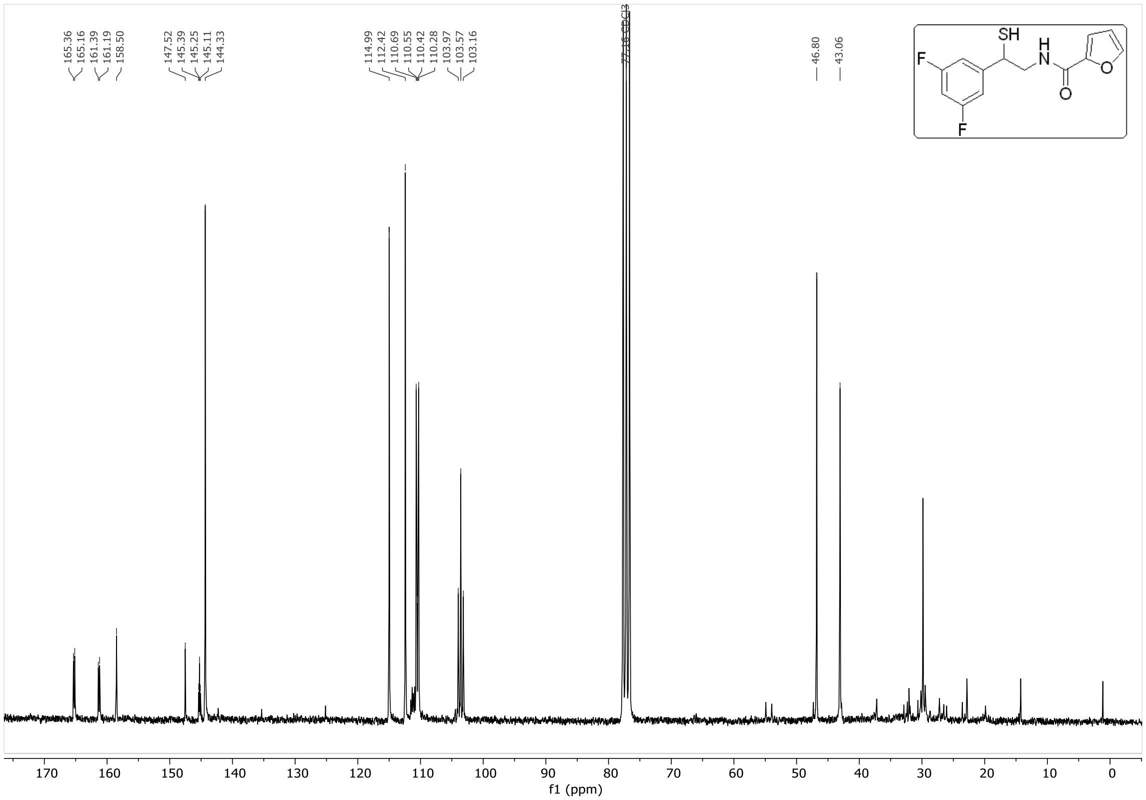

Figure NMR47. ^{1}H spectrum of **25d** in $CDCl_3$

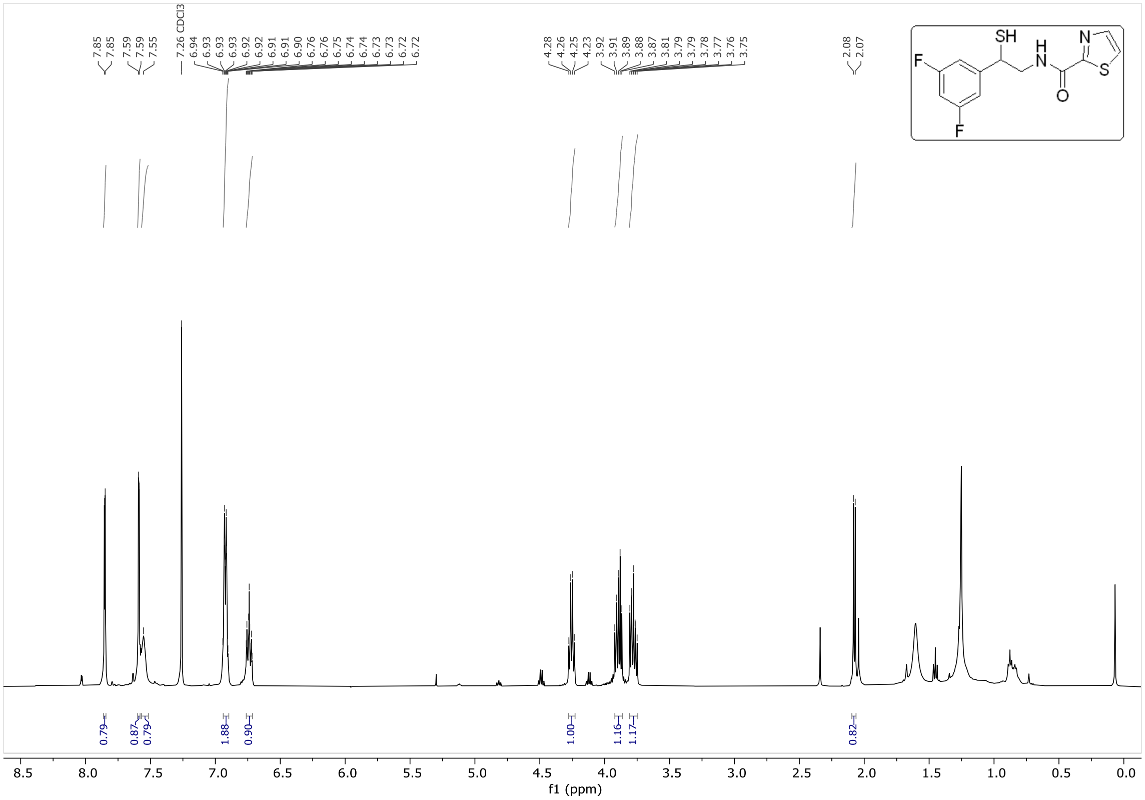

Figure NMR48. ^{13}C spectrum of **25d** in $CDCl_3$

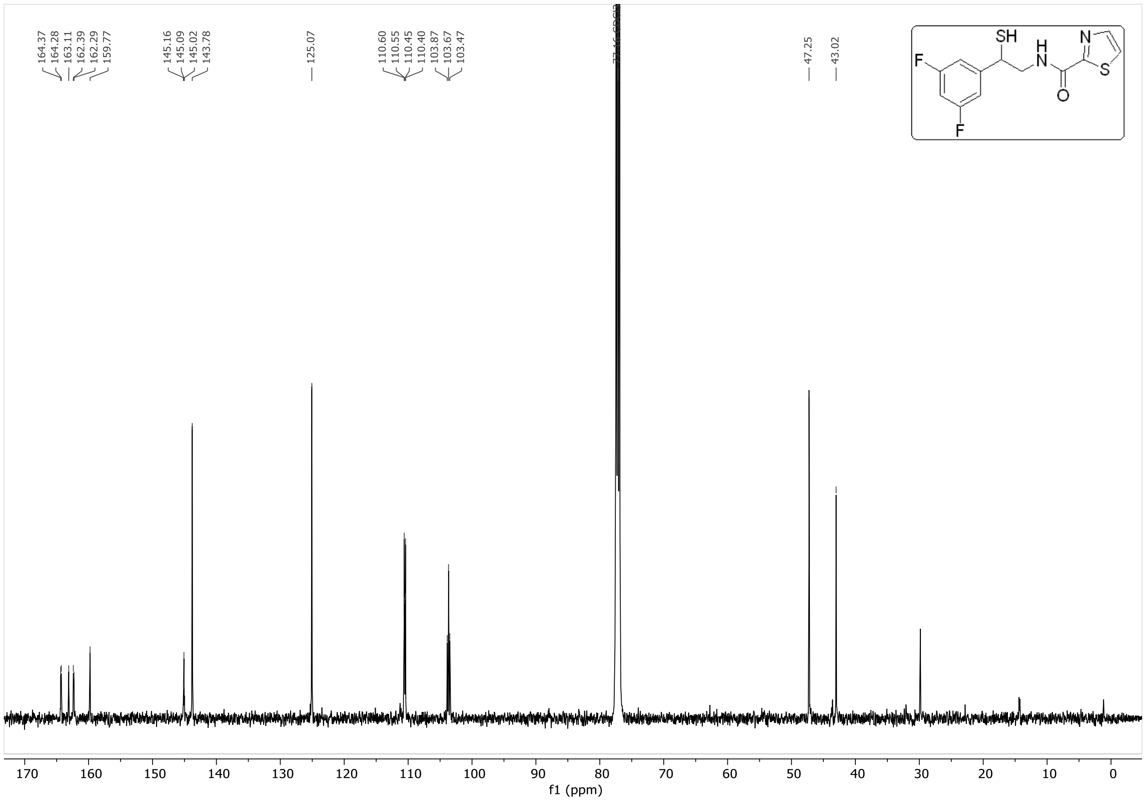

Figure NMR49. ^{1}H spectrum of **25i** in $CDCl_3$

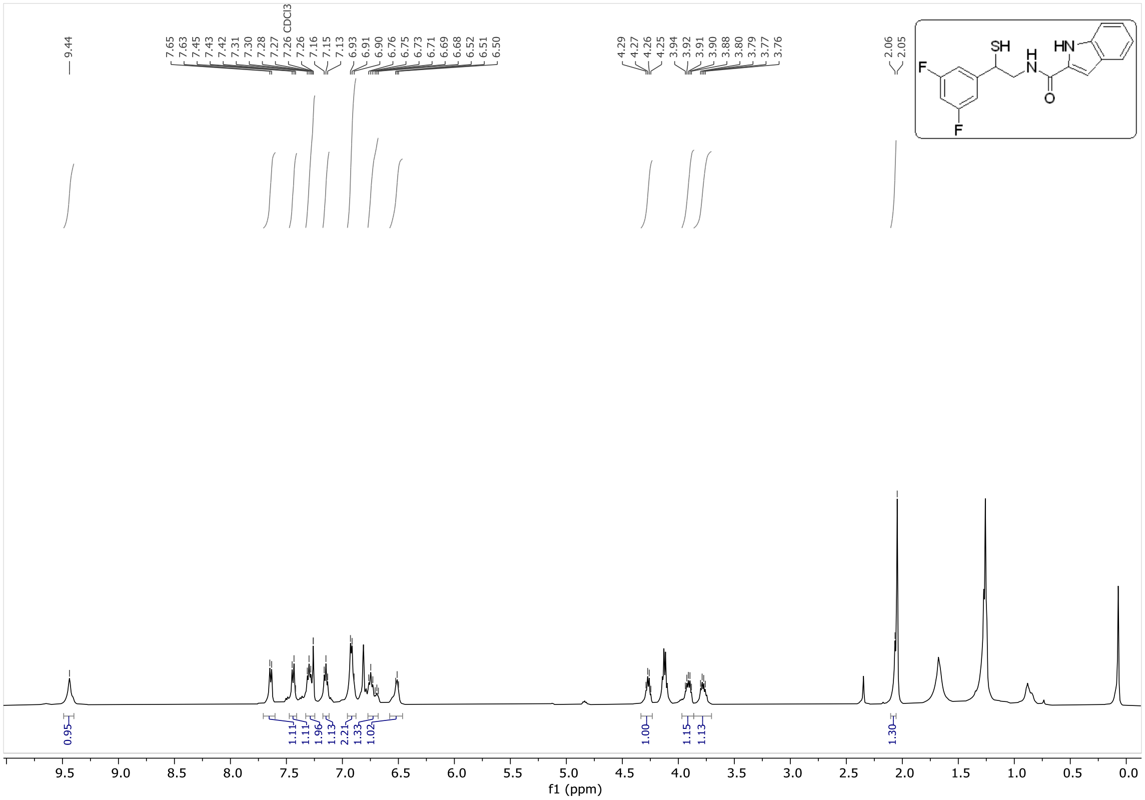

Figure NMR50. ^{13}C spectrum of **25i** in $CDCl_3$

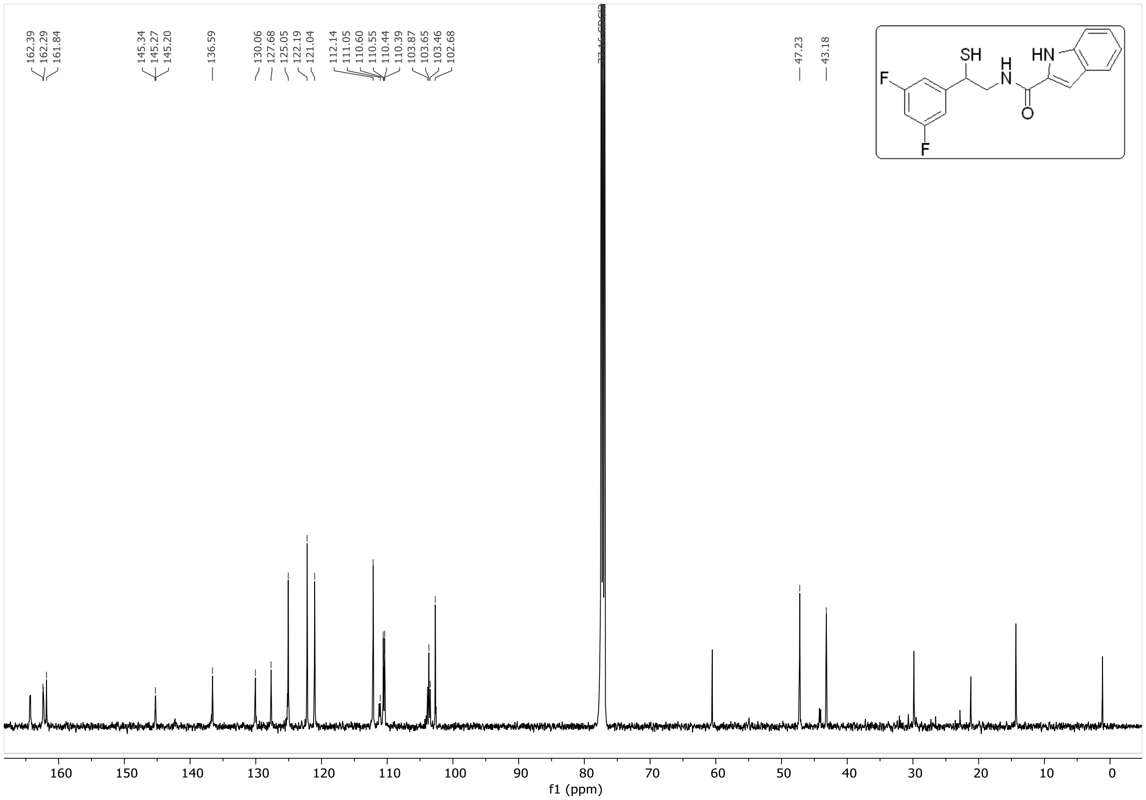

Figure NMR51. ^{1}H spectrum of **28n** in DMSO

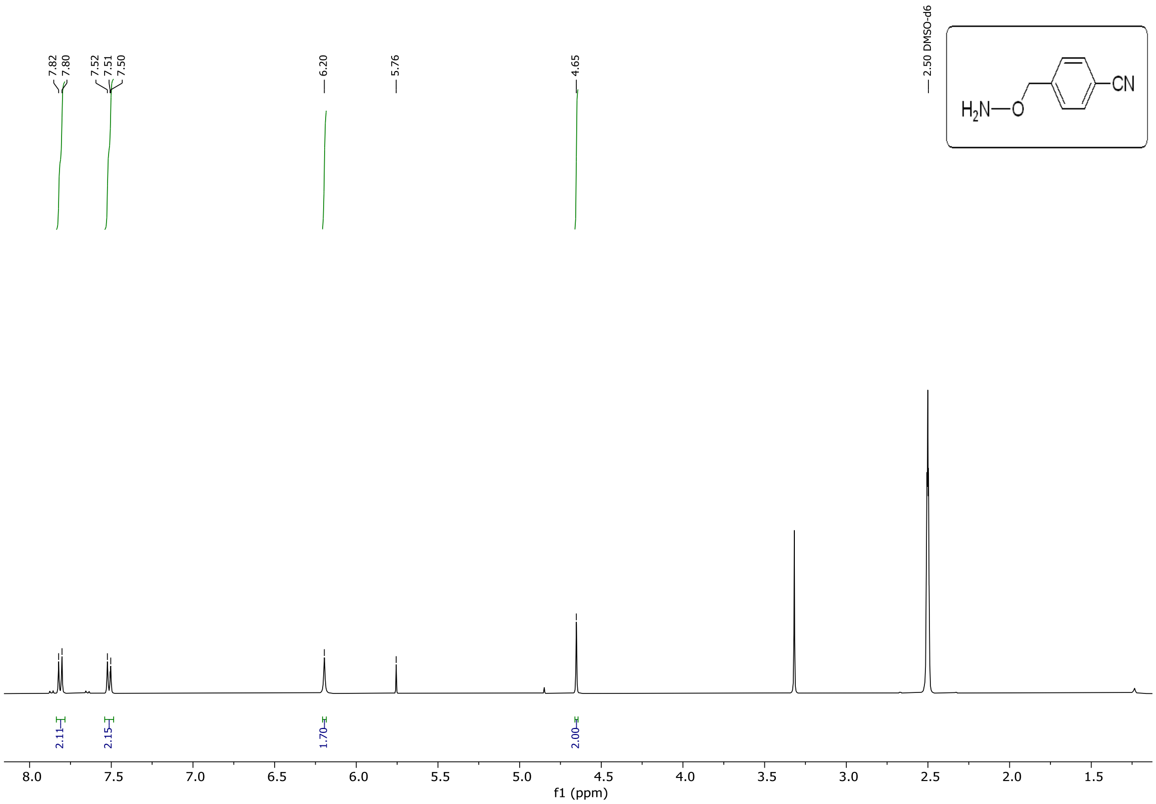

Figure NMR52. ^{13}C spectrum of **28n** in D_2O

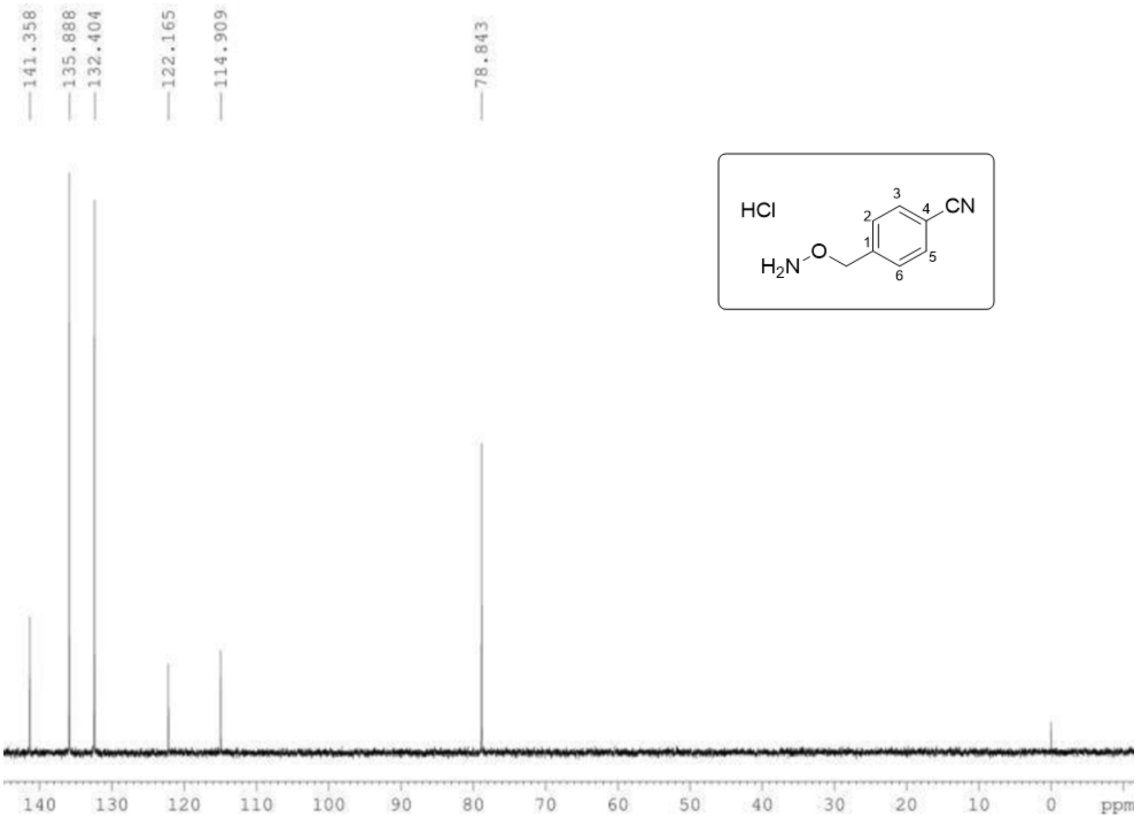

Figure NMR53. ^{1}H spectrum of **33q** in DMSO

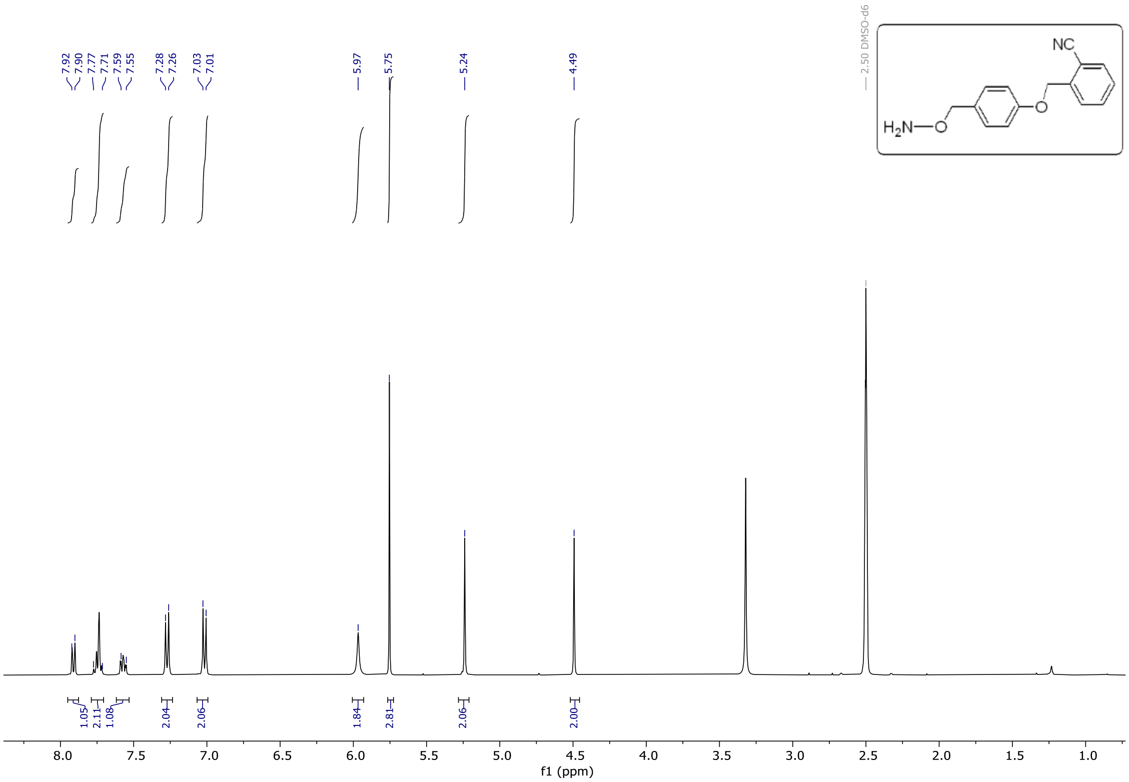

References

[1] D. Moianos, M. Makri, G. M. Prifti, A. Chiotellis, A. Pappas, M. E. Woodson, R. Tajwar, J. E. Tavis, G. Zoidis ''N-Hydroxypiridinedione: A Privileged Heterocycle for Targeting the HBV RNase H'', *Molecules* **2024**, *29*(12).

[2] M. E. Woodson, H. F. Walden, M. A. Mottaleb, M. Makri, G. M. Prifti, D. Moianos, V. Pardali, G. Zoidis, J. E. Tavis ''Efficacy and in vitro pharmacological assessment of novel N-hydroxypyridinediones as hepatitis B virus ribonuclease H inhibitors'', *Antimicrob Agents Chemother* **2025**, *69*(1), e0145524.

[3] Y. Liu, Y. Huang, H. Song, Y. Liu, Q. Wang ''Regio- and chemoselective N-1 acylation of indoles: Pd-catalyzed domino cyclization to afford 1,2-fused tricyclic indole scaffolds'', *Chemistry* **2015**, *21*(14), 5337-5340.
